# Changes in the *Staphylococcus aureus* Transcriptome during Early Adaptation to the Lung

**DOI:** 10.1371/journal.pone.0041329

**Published:** 2012-08-02

**Authors:** Donald O. Chaffin, Destry Taylor, Shawn J. Skerrett, Craig E. Rubens

**Affiliations:** 1 Seattle Children’s Hospital Research Institute, Seattle, Washington, United States of America; 2 University of Washington, Seattle, Washington, United States of America; Cairo University, Egypt

## Abstract

*Staphylococcus aureus* is a common inhabitant of the human nasopharynx. It is also a cause of life-threatening illness, producing a potent array of virulence factors that enable survival in normally sterile sites. The transformation of *S. aureus* from commensal to pathogen is poorly understood. We analyzed *S. aureus* gene expression during adaptation to the lung using a mouse model of *S. aureus* pneumonia. Bacteria were isolated by bronchoalveolar lavage after residence in vivo for up to 6 hours. *S. aureus* in vivo RNA transcription was compared by microarray to that of shake flask grown stationary phase and early exponential phase cells. Compared to in vitro conditions, the in vivo transcriptome was dramatically altered within 30 minutes. Expression of central metabolic pathways changed significantly in response to the lung environment. Gluconeogenesis (*fbs*, *pckA*) was down regulated, as was TCA cycle and fermentation pathway gene expression. Genes associated with amino acid synthesis, RNA translation and nitrate respiration were upregulated, indicative of a highly active metabolic state during the first 6 hours in the lung. Virulence factors regulated by *agr* were down regulated in vivo and in early exponential phase compared to stationary phase cells. Over time in vivo, expression of *ahpCF*, involved in H_2_O_2_ scavenging, and *uspA*, which encodes a universal stress regulator, increased. Transcription of leukotoxic α and β-type phenol-soluble modulins *psmα1-4* and *psmβ1-2* increased 13 and 8-fold respectively; *hld* mRNA, encoding δ-hemolysin, was increased 9-fold. These were the only toxins to be significantly upregulated in vivo. These data provide the first complete survey of the *S. aureus* transcriptome response to the mammalian airway. The results present intriguing contrasts with previous work in other in vitro and in vivo models and provide novel insights into the adaptive and temporal response of *S. aureus* early in the pathogenesis of pneumonia.

## Introduction

*Staphylococcus aureus* is a Gram-positive coccus that exists as normal human flora on skin and nasal passages and colonizes over half of the healthy population [1]. *S. aureus* is a leading cause of hospital and community-acquired respiratory tract infections worldwide and colonization is a major risk factor for developing invasive *S. aureus* disease [2]. The rise of methicillin resistant *S. aureus* (MRSA) and vancomycin resistant *S. aureus* (VRSA) strains brings urgency to understanding the mechanisms that enable *S. aureus* to colonize and in some cases transition to invasive disease [2].

A large measure of the success of *S. aureus* is undoubtedly due to its singular ability to adapt to different environments. Outside of the host, *S. aureus* can endure desiccation and starvation, enabling survival on a wide variety of surfaces [3], [4]. Consequently, *S. aureus* can be difficult to eradicate from hospital settings and outbreaks of staphylococcal infection can result from these reservoirs [5], [6]. *S. aureus* is also the major cause of food borne illness in the developed world due to its ability to survive the acid stress and the low temperatures of many food-processing procedures [7].

In the mammalian host, *S. aureus* survives in a variety of commensal niches, residing on the skin [8], [9], in the nares [10] and the gastro-intestinal and reproductive tracts [11]. In addition, *S. aureus* can invade normally sterile sites. It is a frequent cause of superficial infections that can progress to life threatening illness. Within host cells, *S. aureus* can transition to a small colony variant (SCV) phenotype leading to long-term persistence and recurrent infections [12], [13], [14].

Survival in such a wide variety of environments requires regulation of gene transcription in a manner targeted to the new niche. These adaptive responses can be triggered by changes in nutritional status, quorum sensing, temperature and/or pH [15], [16]. The regulatory networks responsive to environmental cues are being deciphered. For example, the *S. aureus* response to environmental stimuli such as oxidative and disulfhydryl stress [17], response to H_2_O_2_ [18], heat shock, cold shock, stringent and SOS responses [19], pH [15], and cell-wall-active antibiotics [20], [21]) have been explored using proteomics and DNA microarrarys. Other studies have looked at the effect of gene deletions (*clpP* [22], SaeRS regulation [23]) and have examined gene expression during biofilm vs. planktonic growth [24].

Most research in the field, however, has been carried out utilizing in vitro systems, and the *S. aureus* response to the mammalian host in vivo is relatively unexplored. The *S. aureus* gene complement essential for in vivo survival has been studied using IVET (in vivo expression technology) models [25]) and in vivo microarray studies have examined *S. aureus* transcription during internalization into human epithelial cells [26] and “caged” *S. aureus* in a rabbit sub-cutaneous abscess model [27]. These studies have demonstrated that in vivo virulence regulation can differ from than that seen in vitro and there is an increasing appreciation of the difficulty of reproducing the in vivo behavior of *S. aureus* using in vitro models [28], [29].

We have developed a murine pneumonia model [30] to determine the changes in the host lung airway proteome during the development of *S. aureus* pneumonia. We have extended these studies to delineate modifications to the *S. aureus* transcriptome during adaptation to the mouse lung environment. This is the first study to examine the complete *S. aureus* transcriptome in a host lung environment and the temporal evolution of staphylococcal gene expression during the initial stages in the development of pneumonia.

## Materials and Methods

### Ethics Statement

Animal studies were conducted in compliance with the National Research Council Guide for the Care and Use of Laboratory Animals. The mouse experiments were specifically approved by the University of Washington Institutional Animal Care and Use Committee, under protocol number 2671-08. Mice were anesthetized by exposure to 2–4% isoflurane prior to use in procedures and all efforts were made to minimize suffering.

### Strains and Growth Conditions

*S. aureus* strain JP1, a serotype-8 human blood isolate [31], was used for all experiments. For mouse inoculations, 100 µl of *S. aureus* from frozen stocks were inoculated into 10 ml Luria-Bertani broth (LB, Becton Dickinson, NJ) in loosely capped 50-ml Erlenmeyer flasks. The cultures were incubated for 6 hours with shaking (180 rpm) at 37°C. After Gram staining for purity, cultures were diluted 1∶100 into 200 ml of fresh LB (4∶1 air space to medium ratio) and incubated 16 to 18 hours at 37°C with shaking (180 rpm). One ml of culture was transferred to 2 ml of RNA Protect (Qiagen, Valencia, CA), vortexed, incubated at 23°C (RT) for 5 minutes and then frozen at –80°C. The remaining cells were harvested by centrifugation at 10,000×g at RT for 5 minutes, washed 2× with RT endotoxin-free PBS and resuspended in 15 ml endotoxin-free PBS. The culture was diluted to 1.2×10^10^ cfu/ml with PBS and used immediately for inoculation. For samples isolated from growth in fresh LB, 10 ml LB was inoculated from a single colony from overnight growth on blood agar and grown for 16.5 hours at 37°C with shaking at 200 rpm. One ml was immediately transferred to 2 ml RNA Protect. Two 2-ml aliquots of the culture were washed 2× with 37°C PBS and then resuspended in 2 ml pre-warmed (37°C) LB. The aliquots were combined and then incubated at 37°C in a 16×150 mm glass tube set at an angle of 58° with shaking at 200 rpm in air. After 30 minutes, a 1 ml sample was transferred to 2 ml RNA Protect and processed as above.

### Mouse Pneumonia Model

Intranasal inoculation and bronchoalveolar lavage (BAL) were carried out as previously described [30]. Briefly, C57BL/6 mice (Jackson Laboratories, Bar Harbor, ME) were anesthetized by exposure to 2–4% isoflurane and 50 µl of PBS containing 6×10^8^ *S. aureus* CFU was instilled intranasally. After recovery for 30′, 120′ or 360′ minutes the mice were euthanized with pentobarbital (10 mice/time point), exsanguinated by cardiac puncture and both lungs lavaged with PBS, (37°C, 1 ml, then 0.8 ml for each of 3 additional passes). A 50µl aliquot of pooled BAL fluid from each mouse was removed for quantitative culture and the remainder was added to 6.4 ml RNA Protect. The samples were vigorously vortexed, then allowed to stand at RT for 5 minutes and then frozen at –80°C.

### Isolation of Total RNA

The samples were thawed; samples from each time point were combined and then centrifuged at 10,000×g for 15 minutes at RT. The supernatants were removed, the pellets resuspended in 600 µl Qiagen RLT buffer and transferred to 2 ml screw-cap microcentrifuge tubes containing 0.5 ml 200 µm diameter acid-washed glass beads (Sigma-Aldrich, St. Louis, MO), held on ice. The samples were processed for 2 cycles in a FastPrep 120 cell disruptor (MP Biomedicals, Irvine, CA) for 30 sec. at a power setting of 6.5 at 4°C with cooling on ice for 2 minutes between cycles. Following disruption, the samples were centrifuged at 10,000×g for 10 minutes at 4°C, and 300 µl of the supernatant was transferred to 250 µl absolute ethanol, mixed by inversion and transferred to a Qiagen RNA Easy mini column. RNA was isolated as recommended by the manufacturer using an on-column DNase treatment. RNA was eluted in a final volume of 40 µl RNase-free dH_2_O and stored at –80°C. The RNA was quantitated by UV spectrophotometry and evaluated for quality by capillary electrophoresis (Agilent, Santa Clara, CA).

### RNA Labeling and Microarray

Labeling of RNA was carried out using a MessageAmp II – Bacteria kit (Ambion, Life Technologies Corporation, Carlsbad, CA) using the manufacturer’s recommendations with the exception that the biotin-16-UTP (Roche Applied Science, Indianapolis, IN) was increased to 3.08 mM (41% of total UTP in the reaction). The method used for amplification and labeling of mRNA has been shown to yield excellent reproducibility and linearity [32]. The labeled RNA was fragmented, hybridized to Saur2a gene chips [33] (array GPL4047, Affymetrix, Santa Clara, CA) and the probe intensities determined at the Center for Expression Arrays, Seattle, using the manufacturer’s recommended conditions. For the in vivo data, at least 3 biologically independent experiments (from lavage to RNA isolation, labeling and hybridization) were carried out. A control from the inoculating culture was included for each experiment. For determining gene expression during growth in fresh LB, two independently labeled and hybridized to microarrays were done, each from a unique pool of three independent RNA preparations. Controls from the inoculating culture (stationary phase cells) were concurrently prepared in the same fashion. Probe intensity values were normalized to the median value for each microarray using GeneSpring GX version 7.3.1 software (Agilent). Significant differences in the probe set intensities between conditions were determined by ANOVA utilizing parametric testing with variances assumed equal, the criterion for significance set at p<0.05. The Benjamini and Hochberg False Discovery Rate test using Student-Newman-Keuls post hoc tests was used to correct for multiple comparisons. The results were further restricted by requiring at least a 2-fold change in expression between conditions for biological significance. Tables S5 and S6 list the Affymetrix GPL4047 gene chip probes significantly upregulated or downregulated, respectively, in cells harvested after 30′ in vivo compared to stationary phase cells. The microarray data presented here have been deposited in a MIAME compliant form in the Gene Expression Omnibus (GEO) database [34] under accession number GSE30544 (http://www.ncbi.nlm.nih.gov/geo/query/acc.cgi?acc=GSE30544).

### Chromosomal DNA Labeling and Microarray

Chromosomal DNA from *S. aureus* JP1 was prepared from 2 ml of cells grown overnight in LB. Fragmentation of the DNA and hybridization to the Saur2a GeneChip arrays was performed as described [33]. Three independent chromosomal DNA preparations were prepared and hybridized to 3 independent arrays. A gene in strain JP1 was considered present if called present on all three arrays. The set of genes called absent was crosschecked against the set of genes called present from the RNA hybridizations to eliminate false negatives.

### DNA Sequencing

The Multi Locus Sequence Type (MLST) type of strain JP1 was determined from PCR amplicons of internal fragments of 7 “house-keeping” genes generated and sequenced as described [35] using the BigDye Terminator version 3.1 sequencing kit (Applied Biosystems, Life Technologies, Carlsbad, CA). 28 genes associated with virulence were amplified using high fidelity Taq DNA polymerase and DNA primers (table S1) annealing ∼500 bp to either side of the gene coding region. The amplicons were sequenced in both directions using the BigDye sequencing kit and the sequences assembled using Sequencher version 4.2.2 (GeneCodes Corp., Ann Arbor, MI). Completed sequences were compared against the sequenced *S. aureus* genomes using nBLAST [36] http://www.ncbi.nlm.nih.gov/blast/Blast.cgi?PAGE=Nucleotides&PROGRAM=blastn with NCBI Genomes (chromosome) set to *Staphylococcus aureus* (taxid:1280).

### Phenotypic Characterization

AA auxotrophy was determined in chemically defined AA “drop-out” media as described [37], [38]. Determination of α, β, and δ toxin production was ascertained by overnight incubation on rabbit (α-toxin) or sheep-blood (β and δ-toxin) triptic soy agar plates at 37°C in air [39]. Strains RN4220 and Newman were used as controls for β-toxin and α and δ-toxin, respectively. Polysaccharide intercellular adhesin (PIA) production was determined by color on Congo red agar as described [40].

## Results

The goal of this study was to identify genes that are differentially expressed during the course of early infection of the lung. To this end, we analyzed changes in the *S. aureus* transcriptome by microarray during the first 6 hours after introduction into the airway and compared them to transcriptional profiles from stationary and early exponential phase conditions in laboratory media.

### Characterization of JP1

*S. aureus* strain JP1 was used in this study. It is a serotype 8, methicillin susceptible *S. aureus* (MSSA) human blood isolate [30] which produces α and δ-toxins, but does not express β-toxin. JP1 is auxotrophic for arginine, proline, and threonine (data not shown). Since JP1 has not been sequenced, it was characterized by MLST typing [35], genomic microarray [33], [41] and sequencing of selected regulatory and virulence loci. MLST analysis established strain JP1 as belonging to sequence type (ST) 6 [42], a sparsely occupied ST from a diverse range of geographic locales. JP1 is a double locus variant (DLV) of ST5 and is therefore associated with the much larger CC5 [42], [43] whose members include genome reference strains N315 and Mu50 [44].

*S. aureus* strains harboring different alleles of regulatory genes can exhibit discordant patterns of virulence gene expression [45], [46]. To ensure that differences in transcription were not affected by mutations in regulatory genes, 12 regions containing twenty-eight regulatory and virulence loci of JP1 were sequenced. With the exception of *spa*, which diverges rapidly and has been used for characterizing short time-frame *S. aureus* evolution [47], there were few nucleotide differences in coding regions and no changes that resulted in gaps or non-synonymous substitutions in the protein translations (table S2). Of note, *rsbU* of JP1 did not have the 11-bp deletion allele of RN6390 [48] or the Leu18 to Pro18 *saeS* variant found in strain Newman that results in hyper expression of class I *sae* activated genes (i.e. *coa*, *fnbA*, *eap*, *fib*, and *sae*) [23]. The 5′ regulatory regions were also highly conserved. Consequently, for these loci, it is unlikely that virulence regulation would be altered in strain JP1 due to gene deletion or inactivation. Sequencing of the *agr* locus in strain JP1 identified it as belonging to the Agr type I family [49] which also includes *S. aureus* reference genome strains COL [50], Newman [51], USA300_TCH1516 [52], and NCTC_8325 [53]. Remarkably, the entire sequenced *agr* locus was identical on the nucleotide level to USA300-TCH959, a genome reference strain from the human microbiome project (GB NZ_GG697987.1, bases 152642–156859). Strain JP1 was determined to be *spa* type t304, which is widely disseminated in Europe and found as well in the Middle East, Africa, Iceland and North America (http://spaserver2.ridom.de/frequencies.shtml).

To make a complete inventory of the probe sets on the saur2a gene chip that had complements in the JP1 genome, chromosomal JP1 DNA was isolated, labeled, fragmented and hybridized to saur2a microarrays. The saur2a arrays contain 7,792* S. aureus* qualifiers corresponding to 4,380 ORFS, 3,343 probe sets mapping to all intergenic regions of *S. aureus* N315 greater than 50 bp in length and 69 hybridization control probe sets [33]. Six *S. aureus* genomes are represented on the chips, COL, N315, MSSA476, MRSA252, Mu3, and MW2. Three independent trials of chromosomal hybridizations were carried out. Genes on the array not found in JP1 are given in table S3. Notably, most absences could be attributed to mobile genetic elements (MGEs) that are not present in JP1, such as the SSCmec cassette. Interestingly, fragmentary or chimeric versions of several MGEs were found in JP1, indicating the high level of genetic plasticity in these regions. A brief inventory is given in table S4. A phylogenetic tree based on genes present is presented in figure S1 and indicates that of the current reference genome strains, MW2 [54] and MSSA476 [55] are the nearest genetic neighbors to JP1. This result was consistent with the data derived from MLST as can be seen from figure S2 which shows a neighbor joining tree produced from concatenated MLST loci from the 15 extant genome reference strains. Both trees place JP1 between the MLST ST8 strains (NCTC8325, USA300-TCH1516, USA300-FPR3757 and Newman) and MLST ST5 strains (Mu50, Mu3, ED98 and N315). The distances on the tree derived from the microarrays indicate that JP1 is only slightly closer to the cluster containing MLST ST8 strains than that of the ST5 strains, whereas the MLST data indicates that JP1 is slightly closer to the ST5 strains (0.0031 vs. 0.0039 map units). These differences may indicate disparities between the slower evolution of the “core” genome, followed by MLST, and the rapid acquisition and or loss of mobile genetic elements reflected in the microarrays.

### Global Transcriptome Response to the Host Airway Over Time

We hypothesized that upon entry into the airway, the staphylococci would change their gene expression profile in response to the new environment and that adaptive responses would also change over time. Total RNA from BAL fluid was isolated, labeled and hybridized to saur2a gene chips. Samples were obtained after 30, 120 and 360 minutes post intranasal challenge along with a sample of the stationary phase (14–16 hr growth in LB) culture used for inoculation. At least three independent biological replicates were obtained for all samples. As a baseline comparison with actively growing cells, two independent hybridizations were carried out with RNA harvested after growth for 30 minutes in fresh LB, pre-warmed to 37°C.

**Figure 1:**
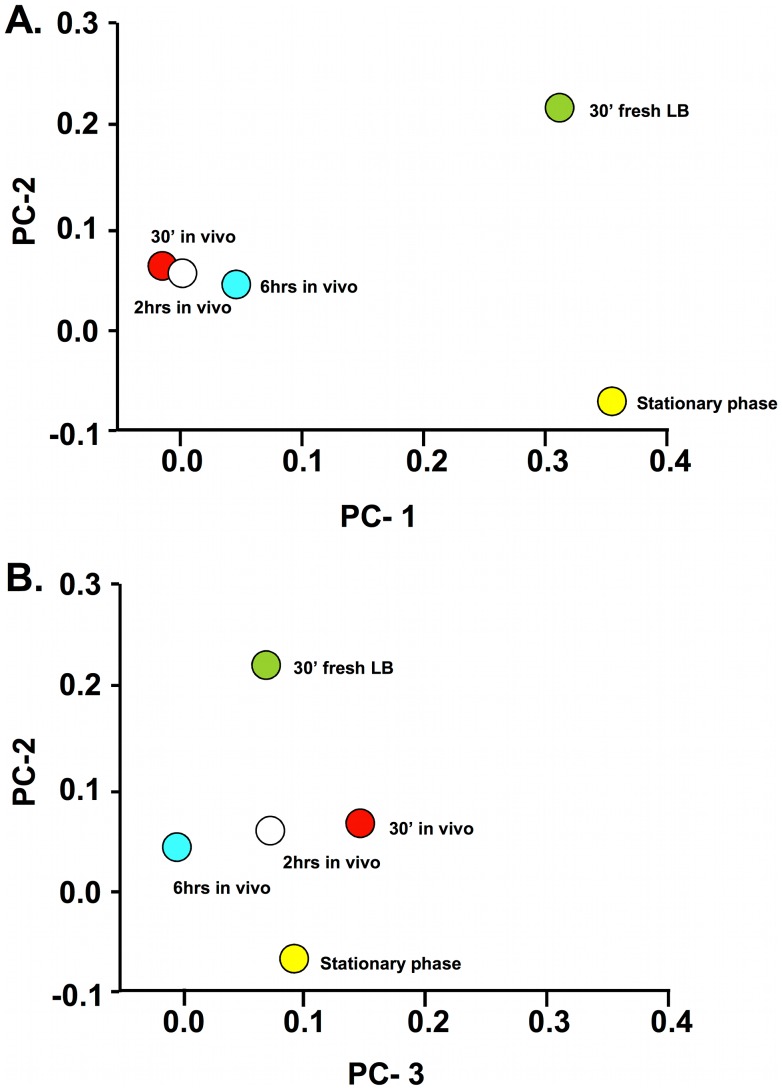
Principle component analysis based on growth condition from Saur2A GeneChip mRNA hybridization data. Mean centering and scaling was used to transform the resulting eigenvectors into indices of explained variability. The axis scale indicates fraction of the total variance explained by the individual principle component plotted on that axis. Each point on the graph is labeled with its particular growth condition. Panel A: Principle component 1 vs. principle component 2. Panel B: Principle component 2 vs. principle component 3.

Principle component analysis was used to examine correlations between the *S. aureus* gene expression patterns from the various growth conditions. The eigenvectors describing the first three principle components accounted for 91.3% of the variance in the dataset. As seen in figure 1, panel A, the first two components separate the in vivo from the in vitro samples (components 1 and 2) and the in vitro samples from each other (component 2). Component 1 and 2 were able to account for 55.4% and 24.6% of the total sample variance, respectively. Only a small portion of the variance explained by the first two components is represented by differences within the in vivo samples, however, which clustered with each other on the plot. Of note, nevertheless, was a trend in the in vivo samples to move over time toward the state of the stationary phase cells. The third principle component, accounting for 11.4% of the overall variance and plotted against component 2 in panel B, accounted for the majority of the variance between the in vivo samples, but little of the variance introduced by the in vitro samples. Thus, categorizing the expression patterns of the cells by the different growth conditions in this study, accounts for most of the variance in the data set and demonstrates that the in vivo conditions provide a distinct growth environment compared to laboratory media and that this environment evolves temporally in a manner that is not akin to either in vitro condition (figure 1, panel B).

**Figure 2:**
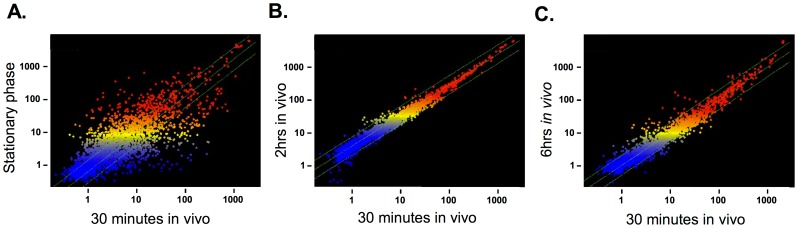
Changes in gene expression between experimental conditions. The scatter plots shown compare gene expression between cells isolated from stationary phase growth and those recovered after 30′ residence in the mouse lung (panel A), after 30′ vs. 120′ in the lung (panel B) in the lung and after 30′ vs. 6 hours in the lung (panel C). Each point represents the expression of a single transcript. The axis denominated by the relative probe intensity values. Probes with the same intensities for each condition compared fall on the diagonal line. Two fold change lines are on either side of the diagonal.

**Figure 3:**
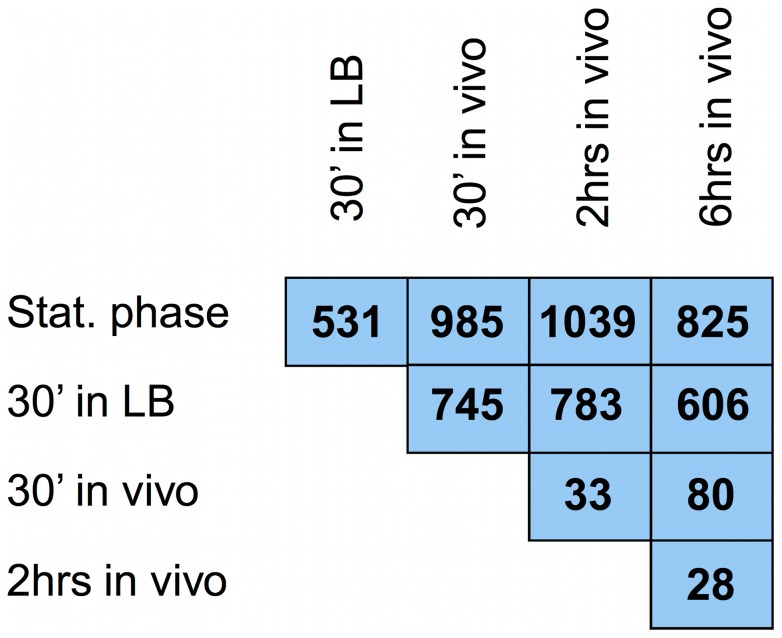
Matrix of significant changes in gene expression between conditions. Gene qualifiers on the microarray were considered significantly different between growth conditions if they were assigned a p<0.05 by ANOVA and also exhibited a fold change difference of greater than or equal to 2.

Scatter plots were used to visualize changes in expression of 2911 *S. aureus* genes (figure 2). Panel A compares stationary phase transcription to that after 30′ in vivo, whereas panels B and C contrast 30′ in vivo with transcription after 120′ in vivo and 6 hours in vivo, respectively. The scatter plots indicate that *S. aureus* adapts within 30 minutes to the mouse lung with extensive changes in their transcriptome. After initial adaptation few additional changes occurred within the first 2 hours. After 6 hours in the lung a modest number of additional changes in expression were seen, indicating further adaptation or apparent changes to the environment. ANOVA, corrected for multiple comparisons, was used to identify probe sets exhibiting significant changes between conditions. Probe sets with a p<0.05 having also a >2 fold change in expression between conditions were considered significant (tables 5 and 6). A matrix of significant changes in gene expression between conditions is shown in figure 3. The greatest number of changes was seen between stationary phase cells and cells retrieved from the lungs at 2 hours. Over 1,000 transcripts were significantly different between the two conditions. The other in vivo conditions produced similar numbers of changes compared to stationary phase cells (985 and 825 for 30′ and 6 hour samples, respectively).

**Figure 4:**
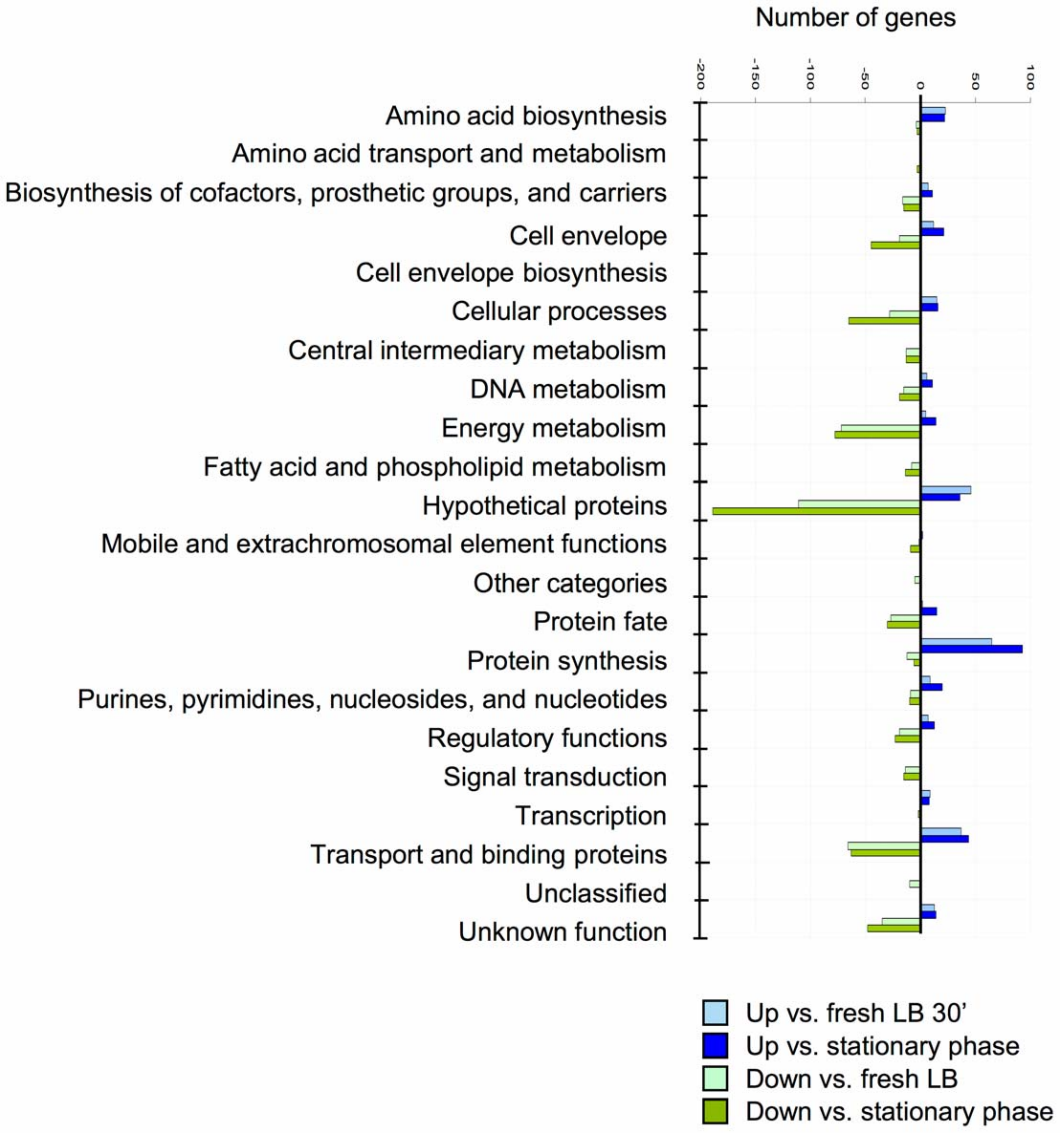
Alterations in transcription grouped according to CVI CMR main role categories. Genes with changes in transcription after 30′ in vivo (compared to cells from stationary phase or growth in fresh LB) were categorized by their main role as assigned by the Craig J. Vintner Comprehensive Microbial Resource. A 2-fold or greater difference in transcription and significance p<0.05 ANOVA was required for inclusion.

To better understand the significance of the changes seen in transcription profiles, transcripts were grouped by TIGR Main Role Category and their expression patterns compared between 30′ in vivo conditions and the stationary phase growth conditions of the initial inoculum. Numerous changes in metabolic state and virulence expression were seen between in vitro and in vivo conditions within 30 minutes (figure 4). Compared to growth in laboratory media, a dramatic increase in amino acid (AA), nucleotide and protein synthesis was observed from the in vivo samples, indicating a high metabolic state in vivo. The large number of pathways activated for de-novo synthesis of metabolites suggested either limited nutrients in vivo or a preference for utilization of a particular carbon source for synthesis and energy needs. To understand the evolving state of the bacteria as they transitioned from growth in laboratory media to survival and growth in the lung airway during the establishment of early pneumonia, we systematically examined, as detailed below, the *S. aureus* transcriptional responses for individual genes within the groups established by the TIGR categories.

### Protein Synthesis

**Figure 5:**
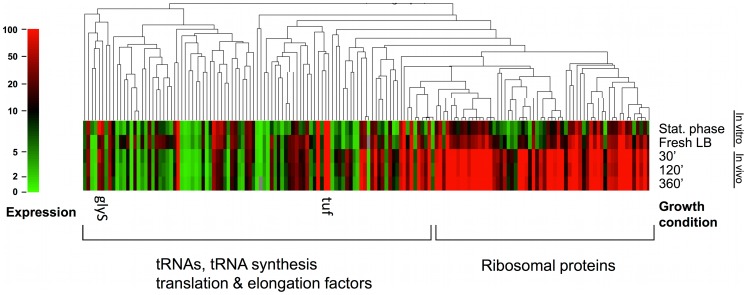
Gene tree with corresponding heat map of genes belonging to COG group J (translation). Genes were organized by average linkage clustering with distances determined by their pair-wise Pearson correlation coefficients. The scale on the left indicates transcriptional activity; classification into ribosomal protein synthesis compared to tRNAs, tRNA synthesis translation & elongation factors is depicted by brackets on the right. Columns in the heat map are labeled at the top with their corresponding growth conditions. Glycine tRNA *glyS* which had highest expression in stationary phase and translation factor *tuf* which was highly expressed under all conditions are labeled on the figure.

Bacteria adjust the relative proportions of RNA, DNA and protein to maximize their growth rate under the particular nutritional conditions in which they find themselves. As the growth rate increases, the ratio of RNA to DNA and protein increases, primarily due to the increase in ribosomal RNAs (rRNA) and the transcription of ribosomal protein mRNAs. In the *S. aureus* mouse pneumonia model we found an immediate upregulation of genes involved in protein synthesis upon entry into the host airway (figure 5). The largest number of differentially transcribed genes was found to be in the subcategory of synthesis and modification ribosomal proteins. Of 61 genes in this category, 54 were upregulated after 30′ in vivo compared to stationary phase cells (88.5%). On average these genes were increased 8 fold. Comparing in vivo cells to those from fresh LB, a subset of the group above (36 genes) were upregulated by an average of 4 fold. Thus, although there was a general activation of ribosomal protein synthesis for fresh LB derived cells, the effect was less than seen in cells derived from the mouse lung at the same time point. Genes that were not significantly different between conditions included ribosomal proteins L9, L33 S21, L7Ae, the putative ribosomal-protein alanine-acetyltransferase SACOL2039 and ribosomal protein L11 methyltransferase gene *prmA*.

Synthesis of tRNA and genes involved in modifications of tRNAs were likewise upregulated in the in vivo samples. Transcription of 33 genes out of 49 in the category were increased compared to stationary phase cells (67%). Twenty-four of these genes were also upregulated after thirty minutes in vivo compared to the fresh LB samples; the higher transcription levels were maintained throughout the 6-hour in vivo time course. The overall high levels of ribosomal protein mRNA and tRNA synthesis in the in vivo samples compared to the stationary phase and fresh LB conditions is consistent with a high growth rate in the host although it is not possible to infer the actual growth rate from the transcriptional data.

### Amino Acid Biosynthesis

*S. aureus* JP1 RNA hybridized with 72 probes for genes identified by TIGR as being involved in AA biosynthesis. Of these, 36 genes (50%) were upregulated after 30′ in vivo compared to the stationary phase and early exponential phase samples. Each of the 6 families of AA biosynthetic pathways were represented in the total (synthesis from pyruvate, oxaloacetate, ribose 5-phosphate, α-ketoglutarate, erythrose 4-phosphate/phosphoenolpyruvate and 3-phosphoglycerate). The highest upregulated genes were *gltBD* (69 fold) coding for glutamine: 2-oxoglutarate amidotransferase (GOGAT), a key enzyme in nitrogen assimilation which synthesizes glutamate from α-ketoglutarate.

The *hisGDSBHAFI* operon for histidine biosynthesis (SACOL2696-2704) was upregulated up to 57 fold after 30′ in vivo compared to in vitro conditions. Expression of a number of genes within the *his* operon were below the level of detection under the in vitro conditions. The *trp* operon (*trpEGDCFBA*) was up also regulated after entry into the lung; most significantly for *trpB* and *trpA*, 16.4 and 23.4 fold respectively, compared to stationary phase expression. The *ilv*/*leu* operon (SACOL2042-2050) that codes for isoleucine, valine and leucine synthesis and produces precursors for alanine were upregulated by as much as 40 fold vs. stationary phase conditions and 60 fold for cells from fresh LB. Lysine and methionine biosynthesis operons were also upregulated 2 to 6 fold in vivo. Five biosynthetic amino acid genes (7% of the total) were down regulated at 30′ in vivo compared to in vitro conditions. SACOL0873 encoding AroD was two fold lower vs. stationary phase cells. The *aro* genes encode production of chorismate, a precursor for tyrosine, tryptophan, phenylalanine and tetrahydrofolate biosynthesis. Another gene that was lower at 30′ in vivo compared to in vitro conditions was SAR2109 (*dapE*), however, this gene maybe miss-annotated since it is more similar to a protease than an aspartate biosynthetic gene. The only anabolic AA synthesis gene expressed at high levels in stationary phase cells was *glnA*, coding for glutamine synthase, a member of the GS/GOGAT (glutamate synthase/oxaloacetate - glutamine amidotransferase) system responsible for nitrogen assimilation. It appeared to be constitutively transcribed since it was equally expressed under all conditions tested. The dramatic increase in de-novo AA biosynthetic pathways is consistent with the increase seen in ribosomal protein transcription and indicates the need to fuel protein synthesis and, potentially, a response to a limited access to exogenous AAs.

### Amino Acid Catalysis

Eighteen genes of 34 (53%) in the TIGR Amino Acid Catalysis category were significantly down regulated at 30′ in vivo compared to stationary phase cells. The greatest change was seen in *rocA* (33 fold lower) encoding Δ-1-pyrroline-5-carboxylate dehydrogenase, a key enzyme in proline and arginine catabolism since it produces glutamate, which can be fed into the TCA cycle. The rest of the arginine degradation pathway was also expressed at a lower level compared to stationary phase cells. Proline catabolism (*putA*), histidine degradation (*hutH*, *hutU*, *hutI* and *hutG*) and tryptophan degradation (*ipdC*), were decreased after 30′ in vivo vs. stationary phase cells.

Several AA dehydrogenases catalyzing the first step in AA degradation (deamination to produce ketoacids) were also decreased in vivo. These included *ilvA* (10.8 fold) and *ald1* which were reduced 40.7 and 14.9 fold after 120′ in the mouse lung vs. stationary phase and fresh LB conditions, respectively. Transcripts for *ald1* were not detected in the other in vivo conditions. Also lower were *ald2*, (7.2, 6.0 and 3.9 fold lower after 30′, 120′ and 360′ in vivo) and the D-alanine aminotransferase encoded by *dat*, which was 3 to 4 fold lower in vivo than in either of the in vitro conditions and like *ald2*, operates on a number of amino acids but specializes in D-isomers.

Finally, transcription of *gluD*, encoding glutamate dehydrogenase, was decreased 8.2, 7.9 and 3.9 fold lower after 30′, 120′ and 360′ in vivo compared to stationary phase cells. Exhibiting one of the highest expression levels for stationary phase cell transcripts, *gluD* is involved in the deamination of glutamate, the point at which numerous amino acid catabolic pathways converge, and as such plays a key regulatory role in balancing the flow of nitrogen and carbon though the cell. Most AA catabolic genes in *S. aureus* are under carbon catabolite repression CCR, ensuring that the most favorable carbon source available will be used for the metabolic needs of the cell [56]. The strong repression of most pathways of AA catabolism in vivo suggests the presence of carbohydrate utilizable for growth by *S. aureus* in the lung environment.

### Glycolysis/gluconeogenesis

**Figure 6:**
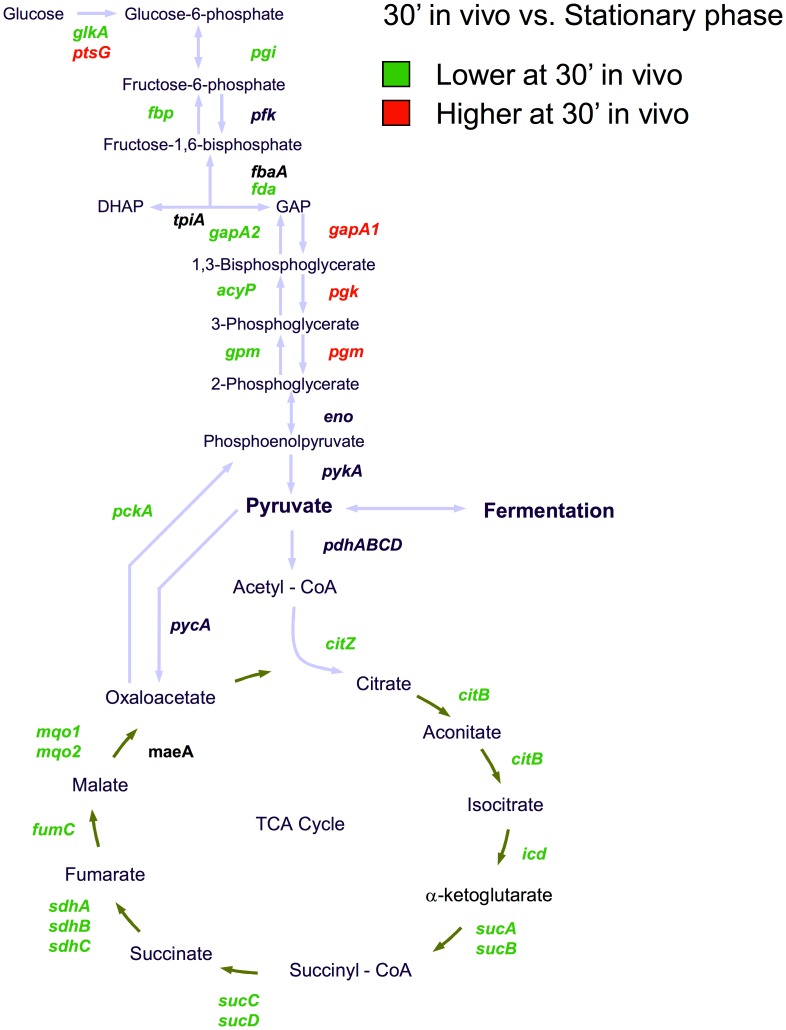
Expression of central metabolic pathways. Genes involved in glycolysis, gluconeogenesis, and the TCA cycle with their respective transcriptional activities are shown. Genes with higher transcription after 30′ in the mouse airway compared to stationary phase cells are labeled in red, those with lower transcription are labeled green and no change is indicated with black.

Compared to both in vitro conditions, glycolysis was upregulated and gluconeogenesis down regulated in vivo, indicating that glucose is the preferred carbon source initially in the airway (figure 6). This was in keeping with the apparent catabolite repression seen for amino acid utilization pathways above. In *S. aureus*, readily metabolizable carbohydrates are converted to pyruvate through glycolysis. When carbohydrate is unavailable, the flow of carbon is reversed since glucose 6-phosphate must be now synthesized for production of nucleotides, erythrose 4-phosphate family amino acids and NADPH via the pentose phosphate cycle.

Three important glycolytic genes that direct the flow of carbon through the cell, *gapA1*, *pgk*, *pgm*, were 4.3 to 4.8, 2.7 to 3.1 and 2.2 to 2.8 fold increased in vivo compared to stationary phase cells, respectively. These genes are co-transcribed from the *gapA1* operon along with *tpiA*, which was also upregulated, but did not reach the threshold for significance. There are two glyceraldehyde 3-phosphate (GAP) dehydrogenases in *S. aureus*, *gapA1* and *gapA2* (*gapB*). In *B. subtilis*, *gapA1* homologue has been shown to be the glycolytic gene, whereas *gapA2* functions in a gluconeogenic capacity [57]. In *S. aureus*, it has been recently shown that *gapA1* and *gapA2* are both regulated by the glycolytic regulator GapR, but are inversely transcribed in response to glucose (*gapA1* is upregulated whereas *gapA2* is downregulated). Additionally, both genes were demonstrated to be important for *S. aureus* virulence [58]. The *gapR* gene is found immediately 5′ of *gapA1* and is a homologue of the *B. subtilis* central glycolytic genes repressor CggR which controls the *B. subtilis gap* operon. Fructose-1,6-bisphosphate (FBP) as well as a number of other glycolysis intermediates upstream of GAP can bind to CggR to relieve repression of the *gap* operon [59]. We found transcription of *gapR* increased in vivo compared to stationary phase cells (7.0, 5.8 and 4.0 fold for 30′, 120′ and 6 hours in vivo) and cells from fresh LB (3.7, 3.1 and 2.1 fold for 30′, 120′ and 6 hours in vivo, respectively) suggesting increased levels of FBP in vivo.

While we found *gapA1* expression upregulated in vivo compared to stationary phase cells, the neo-glucogenic gene *gapA2* was expressed 27, 10 and 6 fold lower after a residence of 30′, 120′ and 6 hours in the alveolar space. Likewise and in contrast to upregulation of glycolysis in vivo, the key genes for gluconeogenesis, phosphoenolpyruvate carboxykinase (PEPCK, encoded by *pckA*) and *fbp*, which encodes fructose-bisphosphatase, were both down regulated in vivo (figure 6). While *fbp* expression did not differ between the fresh LB and stationary phase cells, it was 5 to 11 fold lower for cells from the lung lavage. This consistent with gluconeogenic conditions in the in vitro cells and glycolytic conditions in the in vivo cells.

*S. aureus* contains numerous alternative carbohydrate uptake systems but only 3 genes for carbohydrate transport (10%) were upregulated in vivo. Transcription of the primary glucose PTS uptake system, *ptsG* (SACOL2552) was increased in fresh LB 4.9 fold compared to stationary phase, but increased by 7.3, 9.8 and 16.6 fold after 30′, 120′ and 6 hours in the lung, respectively. Also showing a similar pattern of expression were *glcA* (SACOL0175) and a putative glucose uptake permease (SACOL2246). That these were the only sugar uptake systems significantly increased in vivo is consistent with glucose as the primary carbohydrate utilized in vivo. In contrast, numerous alternative carbohydrate uptake systems were activated in the fresh LB derived cells (trehalose, galactose, fructose, ribose, N-acetylglucosamine) indicating de-repression as expected for growth in LB without glucose. These data also indicate that *S. aureus* exhibits a marked preference for glucose in deference to all the other sugars that can be taken up and catabolized, at least in this airway infection model.

### Fermentation

**Figure 7:**
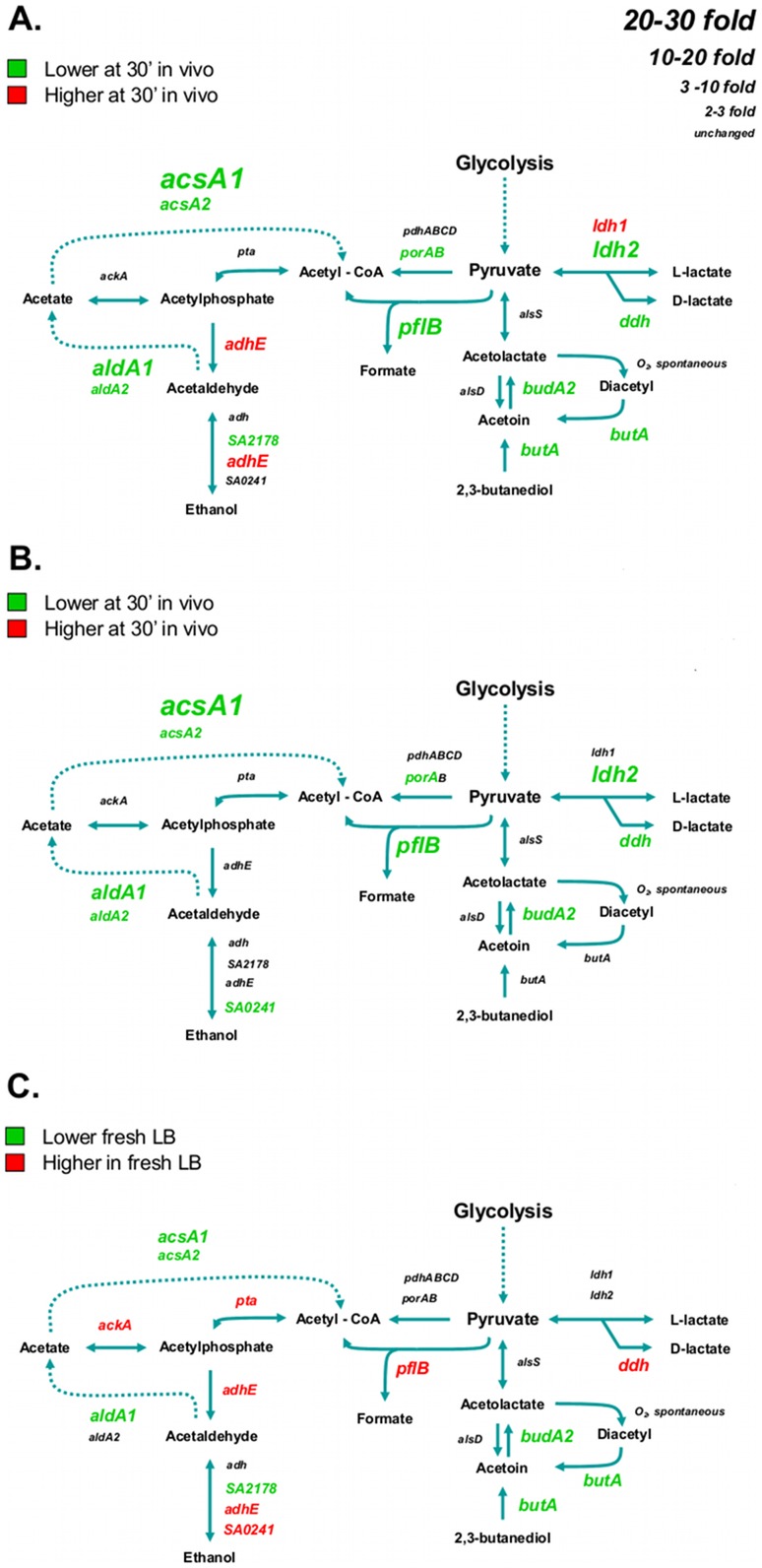
*S. aureus* mixed acid fermentation pathways and the transcriptional activities of the respective genes under in vitro and in vivo growth conditions. Upregulated genes are labeled in red whereas down regulated genes are labeled in green. The size of the label indicates the degree of change between conditions as indicated by the key in the upper right hand corner. Panel A: Stationary phase cells vs. 30′ in the mouse airway. Panel B: Cells grown 30′ in fresh LB vs. 30′ in the mouse airway. Panel C: Stationary phase cells vs. cells grown 30′ in fresh LB.

A general reduction in transcription of genes involved in fermentation was seen in vivo compared to stationary phase (figure 7A) and fresh LB derived cells (figure 7B). The only exceptions were modestly higher expression of alcohol dehydrogenase *adhE* (SACOL0135) and lactate dehydrogenase 1 *ldh1* (SACOL0222) compared to stationary phase cells (figure 7A). The largest decreases in expression compared to stationary phase were seen in *acsA1* (SACOL1783, acetyl-CoA synthetase) and *aldA1* (SACOL0154, aldehyde dehydrogenase) coding for enzymes that recycle the acetaldehyde and acetate produced during exponential phase growth. *acsA1* accounted for the largest decrease in expression compared to fresh LB conditions as well (figure 7B). Pyruvate-formate lyase, *pflB*, (SACOL0204), reported to be preferentially expressed in stationary phase compared to exponential phase in glucose containing media [60], was down regulated 12.1 fold in cells isolated after 30′ in vivo compared to stationary phase cells. In contrast, *pflB* was expressed 6.5 fold higher in fresh LB compared to stationary phase cells (figure 7C). Therefore *pflB* expression was a remarkable 78.5 fold lower when comparing the 30′ in vivo cells to those from fresh LB. Also striking differences were seen between the in vivo and in vitro conditions in decreased expression of *ldh2*, *ddh*, *budA2* and *butA.* It is intriguing that in contrast to the increase in *ldh1* (*lctE*) expression, large decreases were seen *ldh2* in vivo. Expression of *ldh1* is controlled by the oxygen responsive regulators SrrAB and NreB and the redox regulator Rex which senses NAD^+^/NADH balance and is upregulated by by CcpA in the presence of a carbohydrate carbon source [61]. It can also upregulated by NO• stress initiated by the host iNOS system, however, we did not see the concomitant increase in *hmp* expression and observed a decrease in expression of *ddh* which is also upregulated in response to NO•. These observations would discount the role of NO• in the in vivo response here. *ldh2* is not co-transcribed with *ldh1* but is in the same operon as *budA2* involved in recycling 2,3-butanediol via catabolic acetolactate synthesis. BudA2 is distinct from the *alsDS* encoded acetolactate synthesis that results in acetoin and 2,3-butanediol production and is upregulated by glucose, CcpA [56] and by acetate via CidR [62]. The initial step in 2,3-butanediol/diacetyl catabolism is carried out by the *butA* gene product under regulatory control of CodY which is responsive to the cell’s energy levels. Transcription of *ldh2* is largely insensitive to oxygen tension or the presence of NO [63] and unlike *ldh1* is unregulated by redox potential. The coupling of *ldh2* to *budA2* indicates that *ldh2* is activated under conditions of metabolite recycling such as stationary phase growth. Thus downregulation of *acsA1*, *aldA1*, *pflB*, *budA2*, *butA* and *ldh2* are all linked to the sensing of a nutrient environment in the host distinct from that of the metabolite rich milieu of stationary phase cells.

### Tricarboxylic Acid Cycle

The TCA cycle provides biosynthetic intermediates, produces NADH and FADH for reduction in the electron transport system, and ATP to drive cellular processes. TCA cycle gene expression was repressed in vivo for all genes except *mqo2*, compared to stationary phase cells (figure 6). The largest decreases were for *sucAB* (10.8 and 12.2 fold), *fumC* (9.5 fold), *sdhABC* (7.0, 5.0 and 7.5 fold), and *mqo1* (5.8 fold). The remaining genes were 2 to 3.5 fold lower under these conditions. There was a slight increase in transcription of the oxidative fork of the TCA cycle (*citZ*, *citB* and *icd*, 2.5, 2.8 and 2.7 fold, respectively) after 6 hours in vivo compared to the 30′ sample. In contrast to the in vivo conditions, TCA cycle gene expression was unchanged for the fresh LB conditions compared to stationary phase except for *citZ*, *icd* and *mqo1* which were marginally lower in expression. Many of the TCA cycle genes in *S. aureus* are under control of CcpA and are repressed in the presence of rapidly metabolizable carbohydrate. Under these conditions with aerobic growth, pyruvate is diverted from the TCA cycle toward acetate production with the generation of ATP by substrate level phosphorylation. Under anaerobic conditions, pyruvate is diverted to lactate. After available carbohydrate has been consumed, acetate is catabolized via the TCA cycle to produce energy and biosynthetic precursors [64]. This requires the upregulation of TCA cycle genes and the anaplerotic catabolism of AA’s to refresh TCA cycle intermediates [65]. Therefore, the sequence of transcriptional events in vivo is suggestive of initial growth on carbohydrate with a shift toward catabolism of alternative substrates after 6 hours.

### Respiration

Like other facultative anaerobes *S. aureus* satisfies the bulk of its energy needs through fermentation. It contains, however, a complete electron transport system and can carry out both aerobic and anaerobic respiration. The respiratory chain consists of NADH dehydrogenase(s) (complex I) and succinate dehydrogenase (complex II) which transfer electrons to the lipophillic isoprenoid electron carrier menaquinone [66]. Menaquinone passes the electrons on to the terminal cytochromes of complex IV where O_2_ is reduced to H_2_O. A proton motive force is generated at complex I, II and IV which drives the F_o_F_1_ ATP synthase. *S. aureus* can also respire anaerobically using a nitrate reductase complex that reduces NO_3_^−^ to NO_2_^−^.

In concert with the decrease in TCA cycle activity, 10 of 21 genes (48%) assigned by TIGR to the electron transport system were down regulated after 30′ in vivo compared to stationary phase conditions. Complex I NADH dehydrogenases *mnhABCDEFG* (SACOL0949–SACOL0955) and *mnhABCDEFG_2_* (SACOL0680–SACOL0687) were down regulated in the in vivo and fresh LB samples compared to stationary phase cells. The NAD-dependent formate dehydrogenase gene, *fdh* (SACOL0162), however, was transcribed 2.2 fold higher in the 30′ in vivo samples than stationary phase and 2.6 fold higher than fresh LB cells, possibly indicating micro-aerobic conditions.

Three of the four subunits of the cytochrome aa3 quinol oxidase complex (cytochrome *bo* oxidase, *qoxABCD*), the terminal oxidase that transfers 4e^−^ to O_2_ to produce H_2_O, were decreased by 3.5 to 2.1 fold in vivo compared to in vitro conditions. *S. aureus* has a branched ETS where cytochrome *aa3* complex is expressed under aerobic conditions, whilst the cytochrome bd menaquinol oxidase *cydAB*, is expressed under microaerobic conditions. Interestingly, *cydB* was significantly upregulated 2.8 to 2.2 fold in the in vivo samples and fresh LB cells, compared to the stationary phase conditions.

Several thioredoxins were lower in expression after 30′ in the lung: SACOL0875, SACOL0881, (each 3.2 fold), SACOL1794 (2.2 fold), and thioredoxin reductase *trxB* (SACOL879, 2.5 fold). SACOL2534, a hypothetical protein similar to NADH dehydrogenase, and NAD(P)H-flavin oxidoreductases SACOL0941 and SACOL0453 were also lower by 2.2 and 2.9 fold, respectively. Ferridoxin (*fer*, SACOL1525), however, was upregulated 5.0 and 4.2 fold after 30′ in vivo compared to cells from stationary phase and fresh LB, respectively.

The respiratory nitrate reductase operon, *narGHJI* (SACOL2395-2392) was upregulated in vivo as much as 2.7 fold in the 30′ sample compared to stationary phase cells, but this was not statistically different. The nitrate transporter, *narK*, however, controlled by the same oxygen sensitive 2-component response regulator, *nreBC* [67], was significantly upregulated 4.7 to 3.4 fold compared to the in vitro conditions.

The F_0_F_1_-type ATP synthase, *atpBEFHAGDC*, was unchanged compared to stationary phase cells, was down regulated 2.2 to 3.0 fold in comparison to early exponential phase cells from LB. To maintain the redox balance of the cell, NADH and FADH produced by the TCA cycle must be oxidized using the ETS. However, went the TCA cycle is repressed, as is the case here in the mouse lung, the need for ETS expression decreases. Therefore, transcriptional profile observed in vivo for the ETS genes parallels that see for the TCA cycle under the same conditions.

### Biosynthesis of Cofactors

From a total of 77 genes associated with of cofactor biosynthesis, prosthetic groups, and carriers, 11 genes were upregulated and 12 down regulated after being in the lung for 30′ compared to stationary phase conditions. Compared to cells from fresh LB, 8 genes were upregulated and 15 were downregulated. Three genes associated with cobalamin (vitamin B_12_) synthesis, *cobW*, *cobQ* and SACOL0491 were upregulated in vivo. The uroporphyrin-III C-methyltransferase gene *sirB* (*nasF*/*cobA*, SACOL2396), involved in siroheme synthesis, was 3.0 to 3.6 fold higher after 30′ in vivo than in either of the in vitro conditions. Siroheme is a co-factor of nitrite reductase, which reduces nitrite produced during nitrate respiration. The oxygen sensitive response regulator NreBC controls *sirB* expression [67]. Transcription of genes for heme production were not significantly different between stationary phase cells and those from in vivo conditions, but were significantly lower after 30′ in vivo compared to cells from fresh LB for *hemLBI* (3.0 and 3.7 fold) and *hemGHE* (2.1 to 2.3 fold).

Folylpolyglutamate synthase *folC*, encoding involved in the terminal steps of tetrahydrofolate synthesis was upregulated at 30′ in vivo compared to stationary phase and fresh LB derived cells, however, two other genes for folate synthesis, *dfrA* (*folA*) and *folD* were down regulated after 30′ in vivo compared to stationary phase cells and *folP* was 2.2 to 4.0 fold lower in vivo compared to fresh LB conditions.

NAD synthesis genes *nadC* and *nadE* (SACOL1975-1974) were down regulated in vivo 2.5 to 4.8 fold compared to in vitro conditions. The inorganic polyphosphate/ATP-NAD kinase gene, *ppnK*, was also lower. Significant differences in expression were not found for riboflavin, FMN, and FAD biosynthesis. Pyridoxine biosynthesis enzyme *pdxS* had significantly reduced transcription and Thiamine biosynthesis ATP pyrophosphatase *thiL* was modestly upregulated in vivo and in fresh LB vs. stationary phase cells.

Genes for pantothenate synthesis (SACOL2448, *panBCD* and SACOL2615-2613) were not significantly different between in vivo and stationary phase samples but were downregulated 2.1 to 4.4 fold in both in vivo and stationary phase samples compared to cells from fresh LB. Pantothenate kinase gene *coaA* was increased, however, between 4.6 and 3.8 fold after 30′ in vivo compared to in vitro grown cells. CoaA carries out the first step in CoASH synthesis after the production of pantothenate [68]. Since *S. aureus* lacks glutathione production [69], CoASH is the primary intracellular thiol responsible for ameliorating the effects of reactive oxygen species such as peroxides and epoxides. Changes in transcription of cofactor synthesis genes could reflect differences between vitamin availability between the host and laboratory media. LB is rich in B complex vitamins, particularly niacin, pantothenate and riboflavin. Based on our array data, *S. aureus* is able to meet its needs for riboflavin, pyridoxine and pantothenate without increased de-novo synthesis compared to growth in LB. In vivo, however, it appears several other B vitamins (cobalamin, folate, and thiamine) are less available, or the cell’s needs for these cofactors are increased. Thiamine pyrophosphate is a cofactor for pyruvate dehydrogenase, 2-oxoglutarate dehydrogenase and transketolase, enzymes involved in carbohydrate metabolism. Folate is required for DNA synthesis, as is Cobalamin, which is also necessary for fatty acid synthesis. Increased in vivo transcription of the biosynthetic pathways for these vitamins may reflect changes in the energy source and higher apparent growth rate in vivo.

### Transport Systems

A large number of changes in expression were seen between conditions for genes involved in transport of molecules in and out of the cell. Of 310 genes in the category, 144 genes or 46.4% exhibited significant differences in transcription between one or more condition. In comparing stationary phase, fresh LB and in vivo conditions, 39 genes varied between all conditions.

#### Amino acid transport

Sixty-one genes are assigned to amino acid transport by TIGR. Of those 21% were down regulated and 23% were upregulated in vivo vs. cells from stationary phase conditions. Comparing early exponential phase cells to in vivo cells, genes that were upregulated in stationary phase were equally upregulated in the exponential phase cells. Those that were down regulated in vivo were 4 to 10 fold upregulated in fresh LB compared to stationary phase cells. Significantly increased in vivo were SACOL1018, a sodium:alanine symporter, and SACOL2619 and SACOL0630, two cation/H^+^:amino acid symporters of unknown specificity. Several operons coding for branched-chain amino acid (BCAA) transport and responsible for transport of leucine, isoleucine and valine were upregulated in vivo. Also upregulated were the choline-glycine betaine osmoprotectant transporter genes *cudT* (SACOL2632) and *opuD* (SACOL1384) and *opp-1ABCDF*, SACOL2476-71) an oligopeptide permease complex. Interestingly, oligopeptide uptake has been linked with regulation of virulence determinates in a number of species including *S. aureus* and the *opp* operon has been shown to be necessary for in vivo survival [25].

Amino acid transport genes that were repressed in vivo included *opuC* operon (*opuCABCD*, SACOL2453-50) encoding yet another ABC-type choline-glycine betaine osmoprotectant transport system and the nearby D-serine/D-alanine/glycine transporter (SACOL2441). Also lower in expression were the high affinity Na+/proline symporter *putP* (SACOL1963), lysine-specific permease *lysP* (SACOL1728) and SACOL1476, an amino acid permease that is co-transcribed with *ilvA*, the catabolic threonine dehydratase.

It is significant that BCAA import is upregulated in the host. The BCAA isoleucine acts as an important regulatory molecule that helps sense nutrient and energy levels through direct interaction with CodY [70]. When BCAA levels are high, CodY represses transcription of over 100 targets including genes involved in AA biosynthesis and uptake. When BCAA’s are low, repression is relieved. CodY also acts as an activator of virulence components both by direct action upon, and independently of, Agr. Since CodY regulates the uptake of BCAAs, upregulation of BCAA transporters in vivo may indicate low intra-cellular levels of BCAAs and a de-repressive state for CodY with implications for virulence factor production.

#### Carbohydrate uptake

Alternative carbohydrate uptake systems were expressed at significantly lower levels after 30′ in vivo compared to in vitro conditions. Trehalose, glycerol-3-phosphate, fructose and ascorbate-specific transport systems (*treP*, *glpT*, SACOL2663 and *ulaA*) had up to 47.0 fold lower transcription in vivo. Genes with high expression in in vitro but lowest expression in vivo also included SACOL-2521, a protein, similar to glucarate transporters and sucrose-specific PTS transporter protein SACOL0178. Transcription of Na^+^/dicarboxylate symporter, SACOL1979, was lower by 7.3 and 2.4 fold after 30′ in vivo compared to stationary phase and fresh LB conditions.

#### Nucleosides, purines and pyrimidines transport

Four of six genes for transport of nucleosides, purines and pyrimidines were altered between in vivo and in vitro conditions. Genes with lower expression included *pbuX* (18.5 fold). Xanthine/uracil permease family protein SACOL2242, however, had 9.6 fold increased expression compared to the stationary phase samples. Similarly, *pyrP* (SACOL1211), another xanthine/uracil permease, was upregulated 7.5 fold vs. cells from stationary phase. Expression of *pyrP* was not detected in the fresh LB samples.

#### Cation and anion transport

Several cation transport genes were down regulated in vivo compared to in vivo conditions. These included NRAMP family Mn2^+^/Fe2^+^ transporter SACOL1114 (11.2 fold lower compared to fresh LB), molybdenum ABC transporter *modABC* and Co^2+^/Zn^2+^/Cd^2+^ efflux system component SACOL0155; all were lower in expression after 30′ in vivo than for stationary phase cells. Also lower compared to cells from fresh LB was ABC-type cobalt transport system ATPase component *cbiO* (SACOL1085) (3.0 to 3.3 fold lower in vivo). In contrast to the systems above, cation transport gene SACOL0501, a Na^+^-dependent transporter, was transcribed from 8.7 to 64.4 fold higher in vivo compared to stationary phase. Putative cationic transporter SACOL2375 was also upregulated 2.0 to 3.6 fold compared to stationary phase and fresh LB cells. Significant differences in anion transport were not seen between in vivo and stationary phase cells but the ABC-type nitrate/sulfonate/bicarbonate transport system, permease component *susC* (SACOL0159) was strongly downregulated in vivo (10.2 to 18.9 fold) compared to growth in fresh LB.

#### Iron uptake systems

*S. aureus* produces as many as eight systems for iron importation. Expressed at a lower level in vivo compared to both in vitro conditions were Fe3+-hydroxamate transport system SACOL0665-0666, Mn2+/Zn2+ transport system *sitABC* (SACOL0690-0688) (divergently transcribed from the iron dependent repressor gene *sirR* (SACOL0691)), iron transport system SACOL0665-66, and the *isdA*, *isdC*, *isdF* and *isdG* genes of the heme dependant *isd* iron uptake system (SACOL1138-1146). This last system was expressed at low levels under all the conditions tested.

In contrast, the *hts* heme transport system composed of SACOL2167-2165 was upregulated in-vivo and the Fe^3+^-hydroxamate transport system gene *fhuD*, SACOL2277, had 4.3 fold increased expression compared to stationary phase cells. ABC-type Mn2+/Zn2+ transport system permease component *znuB* (SACOL1612) was expressed 6.2 fold higher after 30′ in vivo and the ATP binding component *znuC* (SACOL1613) 3.0 fold higher compared to stationary phase conditions. This operon is divergently transcribed from ferric uptake regulator homolog *furA*, which was transcribed 2.4 fold higher in vivo than in stationary phase cells. Expression of ferrichrome transport permease *fhuG* (SACOL706) was unchanged compared to stationary phase cells but had higher expression (3.5 fold) compared to cells from fresh LB. Iron acquisition is necessary for survival in the host, and the levels of free iron in vivo are extremely low (10^−18^ M). The multiple iron up-take systems in *S. aureus* may reflect the need for systems specialized for specific host compartments, given that they are differentially regulated. Further studies are needed to address these issues.

#### Drug transport

A number of drug/toxin efflux systems were upregulated in vivo. The greatest increase was seen in toxin export gene *msbA* (SACOL1924, 7.3 fold) and the trans-membrane efflux pump gene, *sbtA* (4 and 10 fold higher in vivo after 30′ compared to stationary phase or fresh LB samples). Also significantly higher were putative drug transporters SACOL1809, SACOL2257 and proton/drug antiporter efflux pump SACOL0261. SACOL2157, encoding an EmrB/QacA subfamily drug resistance transporter was 3.1 fold higher in vivo compared from cells from fresh LB. Arsenic pump homolog *arsB* was higher in vivo compared to stationary phase samples. Teichoic acid translocation permease gene *tagG* (SACOL0695) was transcribed 2.1 to 2.7 fold higher in vivo that in stationary phase.

Expressed at lower levels in vivo vs. stationary phase cells were multi-drug transport system gene, *abcA*, quinolone resistance drug transporter gene *norA* (SACOL0754) and putative drug transporters SACOL1475 and SACOL0163. SACOL0163 and *abcA* were also downregulated 6.6 and 2.6 fold in vivo compared to fresh LB grown cells. The multi-drug transport system encoded by SACOL1994 and 1996 was lower compared to in vitro conditions. Putative integral membrane drug efflux protein SACOL2449 and the ABC-type multi-drug transport system encoded by SACOL1994 and 1996 were 3 fold lower in vivo compared to cells from fresh LB.

Interestingly, the ABC-type antimicrobial peptide transport system encoded by SACOL0305–0306, was downregulated as much as 7.1 fold in vivo and in cells from fresh LB (6.6 fold) compared to stationary phase cells. SACOL2644, an ABC-type antimicrobial peptide transport system ATPase was 2.0 to 3.0 fold lower in vivo vs. in vitro conditions.

### Stress

#### Adaptations to atypical conditions

Twenty-three genes are assigned to this category by TIGR; 14 (61%) of these were significantly changed between one or more of the conditions in our study. These genes are responsive to a variety of stresses including heat stress, cold stress, osmotic shock, starvation and general stress conditions. The two alleles of the GTP pyrophosphokinase gene *relA* were down regulated in vivo. Universal stress protein *uspA* (SACOL1759) and its paralogue SACOL1753 were also both decreased upon entry into the mouse airway.

Cold shock proteins encoded by *cspC* (SACOL0861) and *cspB* (SACOL2731) were induced in vivo. The reason for induction of cold shock proteins from the in vivo samples is not clear, but may indicate a response to other factors than temperature. In *E*. *coli*, CspB is thought to act as an RNA chaperone and promotes an increase in mRNA half-lives. In *S. aureus*, *cspB* was induced by cold shock but also induced under stringent growth conditions [19]. Induction of *cspC*, which shares 81% identity with *cspB*, was not reported. A third cold shock protein, *cspD*, which is 77% homologous to *cspB,* was expressed high but invariant levels under all conditions we examined. Our data indicate that cold shock response genes may be important for increased mRNA stability under the unique stresses encountered in the host environment. Also induced in vivo after 30′, but not at either 2 or 6 hrs in vivo, was *betA*, SACOL2627, choline dehydrogenase involved in osmoprotectant glycine betaine synthesis. Four general stress proteins genes were repressed in transitioning from stationary phase: *asp23*, general stress protein 39, *ydaD* and N315-SA2170, a hypothetical protein similar to general stress protein 26. Interestingly, all four genes were even more strongly repressed (as much as 40 fold) when the cells were transitioned to fresh LB.

Changing nutritional conditions can also be a source of considerable stress. Based on the expression of stringent response associated genes, in vivo conditions seem to provide a more congenial environment for *S. aureus* than stationary phase in vitro conditions. Only 31 of 157 genes of the stringent response regulon [19] were upregulated in vivo compared to stationary phase cells. 16 of these were associated with amino acid biosynthesis. In contrast, 52 genes in the regulon were down regulated in vivo after 30′ compared to stationary phase cells. Thirteen of these genes had regulatory functions including activities involved in stress response (*ydaD*, SACOL-1753, *uspA*, *relA*) and pathogenesis (*agrA*, *sarR*), four genes were associated with amino acid transport and catabolism, and 6 genes had virulence related activities (lipases *geh* and *lip*, V8 protease operon *sspABC* and drug efflux pump *norA*). Forty five genes induced under stringent conditions were expressed at higher levels in fresh LB compared to growth in vivo; 25 of these were in common with the genes upregulated in stationary phase discussed above. The remaining genes were largely involved in transport and catabolism functions and energy production. In comparison, only 27 stringent response genes were expressed at higher levels in vivo compared to LB and eighteen of these were associated with de novo amino acid biosynthesis. Of 21 stringent response genes upregulated in fresh LB compared to stationary phase cells, only 5 were also upregulated in vivo vs. stationary phase cells. Four of these were related to amino acid biosynthesis (*metI*, *metB*, *gcvPA* and *ileS*). Thus the in vivo response correlated to stringent conditions consists primarily of induction of AA biosynthetic functions and repression of generalized stress responses and virulence expression. These results indicate that *S. aureus* is well adapted to the host environment found in this pneumonia model and is surprisingly free of indications of overt stress.

#### Oxidative stress

*SA* employs several systems to protect against oxidative damage. *S. aureus* produces both cytoplasmic (*sodA*) and membrane bound (*sodX*) super oxide dismutases (SODs), which convert O_2_^−^ to H_2_O_2_. An extracellular catalase encoded by *katA* can then break down H_2_O_2_ to H_2_O. Staphyloxanthine, a carotenoid pigment, acts as an anti-oxidant and the alkyl hydroperoxide reductase system (*aphCF*) also plays a role in detoxifying H_2_O_2_ and NO. *S. aureus* can produce two glutathione peroxidases (*gpxA1,* SACOL-1325 and *gpxA2,* SACOL-2641) that use glutathione oxidation to reduce H_2_O_2_ to H_2_O. The primary response to nitrosive stress includes induction of the flavohaemoglobin Hmp, proteins associated with the SrrAB regulated anoxic/anaerobic regulon and Fur regulated iron homeostasis systems [63], [71]. Not all of these responses are adaptive. Increases in Fur regulated [Fe_4_-S_4_] cluster synthesis/repair is a major contributor to Fenton chemistry under oxygen stress conditions and can be detrimental to the cell.

In the mouse airway, we found expression of *sodA2* (SACOL-1610), catalase (*katA*) and the alkyl hydroperoxide reductase system (*aphCF*) to all be lower (30′ and 120′ minutes in vivo) compared to stationary phase cells. Expression of *sodA2* and *katA* were lower in fresh LB than stationary phase cells as well, but *aphCF* levels were unchanged. After 6 hours in vivo, however, *aphCF* transcription had returned to stationary phase levels. Glutathione peroxidase *gpxA1* was down regulated in vivo, *gpxA2* was unchanged and SACOL0876, encoding a putative arsenate reductase had lower transcription in vivo than in vitro. The function of this gene is unknown, but the paralogous *spx* (SACOL-1002) is a global regulator coordinating DNA repair in response to disulfide stress [72]. Thioredoxin-disulfide reductase (*trxB*, SACOL-0829) was transcribed 2.2 and 2.6 fold lower after 30′ and 120′ in vivo, respectively, compared to stationary phase cells. Expression of *hmp* (SACOL-0220) was unchanged between in vivo and stationary phase conditions, but exhibited up to 12.8 fold higher transcription levels after growth in fresh media. Overall, there was a reduced activation of oxidative stress response genes in vivo that seen in vitro. Staphyloxanthine, the golden pigment from which *S. aureus* derives its name, is a potent anti-oxidant that protects *S. aureus* from killing by host reactive oxygen species (ROS). Staphyloxanthine synthesis encoded by *crtOPQMN* was dramatically reduced in vivo (5 to 22 fold depending on the individual gene and time point). Transcription of the *crt* operon is controlled by the global virulence regulator σ^b^ [73]. The lack of an upregulation in vivo of markers of oxidative and/or nitrosive stress is surprising, since this is a major means of killing used by MΦ and neutrophils that are present in the airway in this infection model [30]. The results indicate that the *S. aureus* are somehow able to circumvent or avoid killing by these cells via oxidative burst.

*S. aureus* are acid tolerant [74] and are equipped with several mechanisms to deal with acidic environments such as that found in the Mφ phagolyososome. These include production of NH_4_^+^ by urease, expulsion of H^+^ via proton efflux pumps, compensatory adjustments in redox potentials and a switch to non-acidic fermentation products. The stationary phase conditions were alkaline at a pH of 8.6. Modest levels of transcription were seen from the urease operon (*ureABCEFGDI*) and did not differ between stationary phase and in vivo conditions. Transcription of proton exporting NADH dehydrogenase *nuoF* (*ndhF*, SACOL0494) and the F_1_F_0_ ATPase (*atpCDGAHFEB*, SACOL2095-2101) was also not significantly different between conditions (p>0.05).

Under acid stress the pentose phosphate pathway (PPP) is upregulated to compensate for the increased reduction of NADH and NADPH. Glu-6-P dehydrogenase (*zwf*) and phosphogluconate dehydrogenase (*gnd*) control carbon flow through the PPP and are upregulated under acid stress [7]. In the mouse airway, these genes had lower expression than in stationary phase growth. As regards genes involved in neutral end-point metabolite production, fermentation pathways were generally lower in vivo than in vitro although there was a slight increase in *adh* transcription. These results parallel those seen above for oxidative stress and are consistent with either avoidance of phagocytosis or defeat of the early stages of phagolysosome maturation which include phagolysosome acidification. It is interesting to note that the *ure* operon has been shown to be highly upregulated in an in vitro biofilm model compared to stationary phase planktonic growth and emphasizes the conditional nature of *S. aureus* responses to different growth environments.

#### Virulence factors

**Figure 8:**
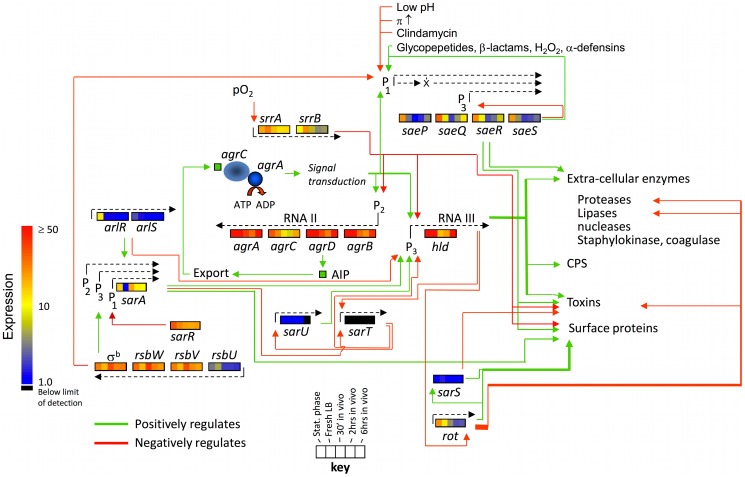
Expression of virulence regulators. Schema of the *S. aureus* virulence regulatory network based on the current understanding of the interrelationships between regulators. Transcriptional changes seen in strain JP1 during adaptation to the mouse airway are shown for each gene. Green and red lines indicate positive and negative regulation, respectively, between interacting regulators. The color scale on the left indicates transcriptional activity for each gene; a key for the gene expression by growth condition is shown on the bottom center of the figure. Transcriptional units are shown as dashed lines above or below the operons originating at their respective promoters. Where operons have multiple promoters, the regulator acting at the specific promoter is diagrammed. Osmotic pressure is indicated by the symbol π. The RNAII and III transcripts are shown as dashed lines above their respective operons.

Virulence is controlled in *S. aureus* by a complex system of interacting regulatory networks that are responsive to changes in both the intracellular and extracellular environment but converge on the *agr* (accessory gene regulator) system comprised of the divergently transcribed *agrBDCA* and *RNAIII* operons. Expression of *agr* is induced by secretion of a quorum sensing auto-inducing peptide (AIP). *Agr* regulates toxins, extracellular proteases and capsule and is expressed in late-log and post exponential phase and repressed in early exponential phase [75] (figure 8). We saw dramatic changes in *agr* expression between the different in vitro and in vivo conditions in this study. Expression of *agrA, agrB, agrC, agrD* and RNAIII was highest in stationary phase cells, lowest at 30′ in vivo (30.5 fold for RNAIII (*hld*) to 4.6 fold for *agrC*).

**Figure 9:**
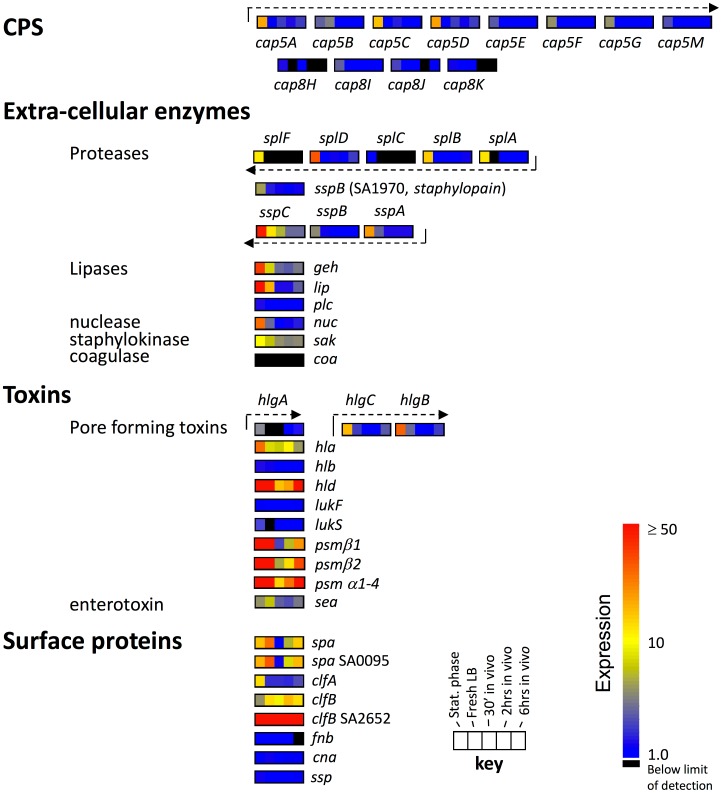
Expression of *S. aureus* virulence factors in strain JP1 during growth in laboratory media and the mouse airway. The color scale on the right indicates transcriptional activity for each gene and corresponds to the key for growth conditions at the bottom right of the figure. Transcriptional units are shown as dashed lines above their respective operons.

Of 46 genes identified as *agr* regulated, 41 had significantly altered expression under one or more of the conditions examined (figure 9). Genes without significant differences were *sak*, three alleles of *clfB* (N315-SA2423, SAR2709 and MW2551), *aur*, *cna*, and *fnb*. A decrease in expression for most genes was seen with the shift from stationary phase to in vivo conditions. The geometric mean expression for 50 *agr* controlled transcripts was 6.2, 6.4 and 4.0 fold lower for growth in after 30′, 120′ and 360′ in vivo, respectively, compared to stationary phase. Transcripts that were significantly lower after 30′ in vivo included cytotoxins *psmβ1* & *2*, (46 and 29 fold lower), *psmα1, 2, 3* & *4* (9 fold lower), *hld* (8 fold lower) and the secreted enzymes *lip* (9 fold lower) and *nuc*, (4 fold lower). Protease, superantigen and capsule gene expression were significantly lower for both conditions compared to stationary phase. This is consistent with the decrease in *agr*-regulated transcription seen after 30′ in vivo. Expression of cytotoxins *lukF* and *hlb* were also significantly lower at 30′ in vivo (2.1 and 2.4 fold, respectively vs. stationary phase).

A decrease in expression for most *agr*-controlled genes was also seen with the shift from stationary phase to fresh media but for most transcripts, the in vivo expression was lower than that in fresh LB. The geometric mean expression for 50 *agr* controlled transcripts was 2.9 fold lower for fresh LB compared to stationary phase cells. Interestingly, the *psm*’s were not decreased in early exponential compared to stationary phase, suggesting either Agr independent regulation or other regulatory factors in play. Protease, superantigen and capsule gene expression were not different between fresh media and 30′ in vivo with the exceptions of *sea*, *sspB*, *ssp* and *plc*. Expression of *sea*, *sspB*, *ssp* and *plc* was not significantly different, however, between early exponential and stationary phase. Additionally, cytotoxin *lukF* and *hlb* expression was not significantly different between cells from fresh LB and stationary phase cultures, unlike their in vivo expression profiles described above.

While most virulence related transcripts obtained their lowest expression after 30′ in vivo and maintained this level over the subsequent 6 hrs, a select set of transcripts (*psm*’s, *hld*, *lip*, *spa, nuc*), steadily increased from 30′ to 360′ in vivo after the initial drop in expression following introduction of the *S. aureus* into the airway. Despite the prevailing idea that *spa* expression is inversely related to that of toxins and other secreted virulence factors [75], [76], the expression pattern of *spa* was highly correlated with that of the phenol soluble modulins *psmα1-4* and *psmβ1* and *2* (Pearson correlation of 0.897, 0.846, and 0.845). This implies that these select genes might be controlled by a common regulatory network that is specifically responsive to the host environment or particular host factors.

#### Expression of modulators of agr expression and their respective regulons

σ^b^ is a *S. aureus* alternative sigma factor and a master switch that potentates cellular responses to numerous stresses [77]. These adaptive responses can in turn modulate important virulence traits such as resistance to antibiotics [78], [79], extra-cellular protease production and biofilm formation [80]. Many of these traits are mediated through σ^b^ regulatory effects on *agr* and specifically RNAIII. In the mouse lung, σ^b^ transcription was not significantly different from stationary phase cells. A similar pattern was seen for the cognate σ^b^ anti-sigma factor, *rsbW*, and the anti-anti-sigma factor *rsbV*, which are co-transcribed with σ^b^. Compared to early exponential phase cells, the *rsb* operon was induced 3 to 4 fold after 30 minutes in vivo (figure 8). σ^b^ has been shown to positively regulate staphylococcal accessory regulator A (*sarA*) which regulates target genes through direct DNA binding; *sarA* is in turn repressed by *sarR*. Expression of *sarA* in this study was well correlated with expression of σ^b^, *rsbW* and *rsbV* (Pearson correlation of 0.964, 0.872, and 0.841) but not with *sarR* (r^2^ =  −0.162) or with *agr* (r^2^ =  −0.128, *agrA*) which it positively regulates in in vitro studies. Transcription of the O_2_ sensing 2-component regulatory system, *srrAB*, which negatively regulates *agr*, toxin and surface protein production, was also not significantly different between in vivo and stationary phase conditions, but was induced 3 fold in fresh LB. In contrast to *srrAB*, the *saePQRS* operon, which has a positive regulatory effect on production of extra-cellular enzymes [81], adherence proteins [82] and toxins [83], [84], was strongly repressed upon introduction into the airway (up to 20 fold, figure 8). The pattern of expression closely followed that of *agr*, which also exerts similar pattern of regulation on these virulence factors and is an up-stream activator of *sae* [85], [86]. Cells from fresh LB also demonstrated *sae* repression, but at one half to one quarter the extent seen in the in vivo samples. The stand-alone regulator, *rot* (Regulator Of Toxins), exhibited highest expression in the stationary phase cells and demonstrated increasingly lower expression at 30′, 120′ and 6 hours in vivo. Expression of *rot* was also lower in the fresh LB samples, but was significantly less repressed than in the in vivo samples. RNAIII has been shown to be a repressor of *rot*, although highest *rot* and RNAIII transcription were both seen during stationary phase conditions.

Transcription of *sarU*, *sarT, sarS, alrRS* was extremely low or undetectable for all conditions, with the exception of *alrR*, which was expressed under stationary phase conditions (figure 8). *sarU* and *sarT* are absent from many *S. aureus* but are present in *S. aureus* JP1 and appear to be functional based on DNA sequencing. Due to their lack of expression, *sarU*, *sarT, sarS* are unlikely to play a major regulatory role in the early adaptation of strain JP1 to the host airway.

### Temporal Changes in Transcription During Residence in the Airway

In contrast to the large number of genes with altered transcription between laboratory media and initial exposure to the lung, 92% fewer differences in transcription were seen in comparing the 6-hour to 30′ exposure to the airway environment (76 vs. 985 probe sets, figure 3). Fifty-one genes were upregulated and 25 genes were down regulated between the 30′ and 6 hour time points. For upregulated transcripts, the changes were confined to a few major genes categories. The TIGR main role category with the largest number of changes was that of Energy Metabolism (12 genes). These included key control points for AA catabolism (*rocA*, *gluD*), TCA cycle (*citB*, *citZ, icd*), fermentation (*aldA*, *acs*), and glycolysis/gluconeogenesis (*gapA2*, *pckA*) suggesting a transition to additional/alternative energy sources after 6 hours in the airway. The second most populous category (10 transcripts) was that of cellular processes. Half of these genes were in the toxin production and resistance sub-category (*psmα1, 2, 3*, and *4*, *psmβ1* and *psmβ2*, *hld*, three alleles of the *mapW* protein (MW1880), pathogenesis gene *spa* (two alleles), detoxification genes *ahpF* and *C* and *gpxA* and general stress proteins 20 U (*dps*) and universal stress protein *uspA*). Of interest, three of four genes in the *sae* operon (*saePQR*) were upregulated (4.3, 3.6 and 2.2 fold, respectively) after 6 hours in the lung. SaeRS is a two-component response regulator that is responsive to a number of phagocytosis related stimuli including pH, oxidative stress and α-defensins [85]. The third largest category comprised transport and binding proteins and was largely composed of various carbohydrate transport systems. These included the glucose-specific PTS IIABC transporter component *ptsG*, fructose/(mannose)-specific PTS IIABC component SACOL2663, maltose/maltodextrin ABC permease *malK* and glycerol-3-phosphate transporter gene, *glpT*. Also upregulated were the phosphate transporter *ptsS*, sodium:solute symporter family gene SACOL0311 and putative transport gene SACOL2521. Like the genes involved in energy metabolism above, points to relief of carbon-catabolite repression of operons encoding alternative carbohydrate uptake systems.

Genes down regulated at 6 hours were also confined to a few TIGR categories. Amino acid biosynthesis genes comprised 40% of the down regulated transcripts; aromatic, glutamate and histidine AA biosynthesis families were represented. Several genes related to stress responses were also down regulated- *rsbV* and *rsbW*, the anti-anti-σ^b^ and anti-σ^b^ factors, *smpB*, which facilitates targeting for degradation proteins on stalled ribosomes and *spoVG*, a regulator of CPS production and methicillin/glycopeptide resistance in *S. aureus* [87] and a homologue of the *B*. *subtilis* spore formation regulator *spoVG*. Control of *spoVG* is σ^b^ dependant [88], in turn *rsbV* and *W* control σ^b^ activity thru post-translational interactions. These genes were highly correlated in their patterns of expression across all conditions tested. Also decreased in expression after 6 hours in vivo were genes associated with fermentation (*fdh*, *adhE*), transport (SACOL0946, *sbt*) and nucleotide salvage pathways (*pyrR*, SACOL0225) and *rmsC* which encodes a protein that facilitates in ribosomal subunit assembly. The transcriptional changes seen at the six hour time point indicate a decrease in available carbohydrate for energy and a concurrent switch to a more aggressive stance by the *S. aureus* as shown by the increase in toxin production. The increased virulence factor production is consistent with the upregulation of the *agr* operon at this time, but it is interesting that this coincides with the need to exploit additional energy sources, as demonstrated by the upregulation of alternative carbohydrate up-take systems and pathways for AA catabolism. It is reasonable to expect that virulence factor production and metabolic processes should be linked, since tissue damage resulting from toxins can provide nutrients once preferred energy sources are expended.

## Discussion

Over the past ten years microarray technology has been extensively used to study the *S. aureus* transcriptome. These studies have examined the effects of specific regulatory and metabolic gene deletions as well as the transcriptional changes induced by a wide variety of environmental and growth conditions. *S. aureus* have been shown to sense and adapt to a wide variety of stimuli, including pH, p0_2_, temperature and nutrient levels. Many of these factors have been shown to have direct effects on *S. aureus* virulence. Few studies, however, have examined *S. aureus* transcription in the setting of the host environment. For the most part, knowledge of the environment found in various host niches is largely guesswork, as is the physiological state of the *S. aureus* that might be residing in them. It is clear from IVET and STM (signature tagged mutagenesis) studies that *S. aureus* must adapt to survive within, and transition between, different host compartments.

The aim of this study was to document the changes in *Staphylococcus aureus* gene expression induced by conditions in the lower murine airway and, additionally, examine temporal changes in gene expression during the development of the early stages of airway infection. The results presented here reveal not only what the *S. aureus* are “thinking” as they adapt to the host, but also give clues to the environment in which they find themselves. The *S. aureus* act, in effect, as super sensitive “biosensors”, reporting from the host on a plethora of parameters. Based on the *S. aureus* transcriptional responses reported here, the circumstances in the lower airway are radically different from those of laboratory conditions, with expression of as many as 1000 transcripts significantly altered between in vitro and in vivo growth. Growth conditions were also seen to change over time in the lung, although not as dramatically, with 80 transcripts changed between growth for 30′ and 6 hours in vivo.

The lower airway appears to be a high growth environment for *S. aureus*. Ribosomal protein and tRNA synthesis, which are markers for bacterial growth, were highly upregulated, more so than cells incubated in fresh LB. Growth was primarily fueled by carbohydrate, most likely glucose. This was indicated by upregulation of glycolysis and downregulation of alternative carbohydrate uptake systems, pathways for gluconeogenesis and amino acid catabolism, and lower transcription of the TCA cycle and ETS. These changes are characteristic of glucose mediated carbon catabolite repression. The upregulation of AA biosynthetic pathways in vivo indicates an environment low in available AA relative to the protein synthesis needs of the *S. aureus*. This was also indicated by upregulation of BCAA import, which is a key marker of intracellular isoleucine availability. The disposition of the central metabolic pathways in the host is important since their regulation is mediated by global regulators (CcpA and CodY) that also exert control over virulence networks. The TCA and ETS systems are also major targets of oxidative killing, and their downregulation due to CcpA and CodY repression could be advantageous in light of MΦ and neutrophil killing mechanisms.

The lack of changes within stress responsive gene networks after the *S. aureus* were resident in the lungs indicated that they did not, initially, sense conditions of lower pH, oxidative, or nitrosive stress. This was a surprising result since these stresses are commonly applied by the host as defense mechanisms used in response to infection. After 6 hours of in vivo growth, however, *ahpCF*, involved in H_2_O_2_ scavenging, had 3.5-fold higher expression. The universal stress regulator gene *uspA* was also elevated. These responses may indicate an increase in exogenous derived oxidative stress, although by 6 hours, perhaps due to insufficient carbohydrate, there is an increase in AA catabolism with a concomitant increase in TCA cycle activity which can generate endogenous oxygen radicals. Certain virulence genes were also upregulated at 6 hours, which may indicate a response to the AIP quorum sensing peptide. The *agr* operon was upregulated as was transcription of the *agr* regulated leukotoxic α and β-type phenol-soluble modulins *psmα1-4* and *psmβ1-2* (13 and 8-fold respectively); *hld* mRNA (part of the RNAIII transcript), encoding δ-hemolysin, was increased 9-fold. Surprisingly, these were the only toxins to be significantly upregulated in vivo, despite *agr* induction.

The changes seen during adaptation to the host airway were different that those found in other in vitro and in vivo models such as growth in batch culture [75] and internalization by PMNs maintained in cell culture [89]. *S. aureus* instilled in the airway rapidly became metabolically active, similar to when they are shifted from stationary phase to fresh media. This was unlike intracellular models utilizing PMNs, where little or no growth was seen over the first 2 hours. The activation of *S. aureus* metabolic pathways in PMNs was also different. Gluconeogenesis (*fbp*, *gapB*), TCA cycle (*citB*, *citC*, *citZ*, *sucCD*, *sdhABC*), fermentation (*aldA*, *acsA*) were upregulated in neutrophils but were down regulated in the lung. These data indicate that *S. aureus* senses and adapts to a significantly different nutritional environment between these conditions. Our results also differ from the metabolic responses of *Streptococcus pyogenes* isolated from murine subcutaneous “caged” cultures [90]. Fewer transcriptional changes between in vitro and in vivo conditions (266) were seen in this study and correspondingly, there were comparatively smaller differences in metabolic processes. Of interest, upregulation of protein synthesis, the switch from gluconeogenesis to glycolysis, increased de-novo AA biosynthesis and downregulation of fermentation were not seen, suggestive a slow growth, AA rich environment similar to the in vitro post exponential phase culture control. There were, however, a large number of changes associated with utilization of carbohydrates other than glucose between in vitro and in vivo conditions. As seen in the present study, trehalose and cellobiose PTS systems, related to uptake of yeast related carbohydrates, were downregulated in vivo, but unlike the *S. aureus* response to the murine lung, mannose, glycerol, N-acetylglucosamine, N-acetylgalactosamine, N-acetylneuraminate, and fructo-oligosaccharide uptake systems were activated. These results point to the wide differences between niches within the host and the need for multiple adaptive strategies for microbial survival in vivo.

In the murine airway, production of virulence factors such as proteases, lipases and toxins were decreased in a manner similar to that seen after the transition from stationary phase to early exponential phase in batch culture [75], [76]. Other factors, however, such as surface proteins and adhesins, which are typically upregulated in early to mid exponential phase in batch culture, were either down regulated or unchanged after transfer to the lung. The only exceptions were *ssaA* and other genes of the WalK/WalR regulon, which are involved in cell wall remodeling and turnover [91]. This was despite the initial downregulation in the airway of *agr,* the principle regulatory agonist for surface protein and adhesin expression. In contrast to the initial adaptation to the mouse lung, surface virulence factors, most notably *spa*, were upregulated in our fresh media controls. After 6 hours in the lung *spa* was strongly upregulated despite concurrent upregulation of RNAIII, which in batch cultures represses *spa* expression [76]. Similar to *spa*, transcription of *sasA*, encoding a fibrinogen binding protein, was lower in the lung while upregulated in fresh media. Also transcribed lower in the airway was the fibrinogen binding protein gene *fib*. Conversely, the *cap* operon, encoding capsule biosynthesis, and which has been shown to be under positive regulation by both *agr* [92] and *sae* [93], was initially down regulated in the mouse airway but failed to be upregulated after six hours in the lung.

Unlike both batch culture and the mouse airway transcriptome presented here, *S. aureus* has been shown to concurrently upregulate both toxin *and* surface protein expression immediately after being taken up by leukocytes [89]. In these experiments, toxin and surface proteins expression was independent of *agr* since *agr* and RNAIII transcription was not significantly different between cells from PMNs and the batch culture controls. Cells isolated from PMNs also exhibited a strong upregulation of genes associated with oxidative stress, a response not seen in this study as *S. aureus* habituated to the murine airway. The oxidative stress response seen in the PMN model is consistent with the ability of these cells to deliver a robust oxidative burst compared to other cells such as macrophages and non-professional phagocytes. However, even at the 6-hour time point in the murine airway, only mild oxidative stress (upregulation of *ahpCF*) was seen despite a considerable neutrophil infiltration. The lack of a marked stress response in *S. aureus* recovered from the airway may be related to the large proportion (>80%) of cells associated with alveolar macrophages (data not shown), although the *S. aureus* used for these studies included both intracellular and extracellular staphylococci from the bronchoalveolar space. A recent study found that *S. aureus* can persist and replicate in macrophages phagosomes for up to 5 days. It was speculated that macrophages could act as a reservoir where *S. aureus* could avoid immune recognition and killing [94]. Our results suggest that *S. aureus* may be protected early in the establishment of pneumonia by being taken up by alveolar macrophages, where they are unavailable for phagocytosis and destruction by incoming PMNs. Differences between *S. aureus* in vivo and in vitro virulence factor expression have been reported for a rabbit endocarditis model [95], device related infections [84], and staph recovered from the cystic fibrosis lung [96]. These data, along with the data presented here, indicate that *S. aureus* tailors its spectrum of virulence factors to fit the unique environments it encounters in the host.

The nutritional environment found in the murine lung may influence the selective activation of virulence factors. As discussed above, the primary energy source in vivo seemed to be carbohydrate, based on repression of gluconeogenesis, the TCA cycle and amino acid catabolism, all known to be controlled by carbohydrate mediated catabolite repression. In turn, catabolite repression has been shown to modulate virulence gene expression in *S. aureus* [56], [61] and other Gram-positive organisms such as *B. subtilis*, *L. monocytogenes*, *S. pyogenes* [97], [98] and *S. agalactiae*. Additionally, other studies have demonstrated a connection between sensing of intracellular AA and GTP pools through CodY and expression of virulence regulators *agr, RNAIII* and σ^b^ and production of capsule, adhesins, toxins, extracellular proteases, and pigment [70], [99], [100]. We saw large changes in CodY controlled operons (BCAA uptake, leu, lle and val biosynthesis) for *S. aureus* resident in host airway which suggests that CodY may also be active in modulating virulence responses in vivo.

In sum, we found that the *S. aureus* transcriptome is rapidly and radically altered in transitioning from in vitro conditions to the mouse lung. The adaptation of *S. aureus* to the alveolar space involved changes in numerous nutrient acquisition and utilization pathways indicating that this host compartment presents a significantly different growth environment to the *S. aureus* than that of LB. Virulence components were initially down regulated in vivo, perhaps due to low *agr* induction in the initial absence of the auto inducing peptide. Upregulation of *agr* and *sae* was seen by 6 hrs in vivo along with induction of several virulence factors. Not all virulence components, however, were equally activated. The leukotoxins *psmα1-4*, *psmβ1* & *2* and δ-toxin, *hld* were highly expressed while α-toxin and γ-toxin remained down regulated. Staphylococcal protein A (*spa*) was activated at 6 hrs in vivo despite high levels of RNAIII and low levels of *rot*, which have been shown to repress and activate *spa* in vitro, respectively. In comparison to other models that have been used to explore *S. aureus* gene expression, the mouse airway induced unique patterns of transcription.

These data provide novel insights into *S. aureus* adaptation to the airway during initial colonization and initiation of infection, and emphasize the importance of in vivo models in elucidating *S. aureus* virulence mechanisms. It also represents the first complete survey of the *S. aureus* transcriptome response to the host airway. The results present intriguing contrasts with previous work in other in vitro and in vivo models and offer the sole data set for the temporal response of *S. aureus* to the lung environment early in the establishment of pneumonia.

This work was presented, in part, at the 109^th^ general meeting of the American Society for Microbiology in Philadelphia, PA, May 17–21, 2009 (Poster B-201).

## Supporting Information

Figure S1 **Neighbor-joining tree constructed from Saur2A GeneChip chromosomal DNA hybridizations using Genedata Expression Analyst Pro v3.1.11.** Aquamarine terminal nodes indicate CDC *S. aureus* isolates, whereas magenta colored terminal nodes specify sequenced genome reference strains. Three independent biological replicate hybridizations of JP1 chromosomal DNA are represented in gold. Branches encompassing the USA300 and USA400 lineages are designated.
(PDF)

Figure S2 **Phylogenetic tree based on concatenated MLST DNA sequences of 15** ***S*** **.** ***aureus*** **genomic reference strains (indicated by magenta) and strain JP1 (gold) computed using the Multiple Sequence Comparison by Log-Expectation (MUSCLE) algorithm.** The MLST type for each strain is given adjacent to the strain name assigned to each leaf node.
(PDF)

Table S1 Table A: DNA sequences of PCR primers used in the study. Table B: DNA sequences of sequencing primers used in the study.
(PDF)

Table S2 Table of nucleotide differences between S. aureus JP1 and the genetically closest reference strain. The JP1 allele is given in the 1st column, column 2 contains the nearest reference strain, columns 3 and 4 give the start and stop of the region sequenced mapped onto the corresponding reference strain genome, column 6 indicates the number of mismatches and/or gaps between JP1 and the reference strain, column 7 gives the location of the mismatch within the JP1 sequence, column 8 indicates if the mismatch is within the coding of the allele and column 9 indicates if the mismatch creates a synonymous or non- synonymous AA substitution.
(PDF)

Table S3 Table of non-hybridizing Affymetrix GPL4047 gene chip probes. Columns 1 through 12 give the Affymetrix probe identifier, the *S. aureus* strain COL, N315, MSRA, MSSA, Mu50, and MW2 gene designations, the gene name, the GenBank identifier, the GenBank description, the gene function, the TIGR main role category and TIGR subcategory role, respectively.
(PDF)

Table S4 Disposition of Pathogenicity Islands and Mobile Genetic Elements in *S*. *aureus* strain JP1. Column 1, genome locus; column 2, notes on the presence and arrangement of the genetic element within S. aureus strain JP1.
(PDF)

Table S5 Table of Affymetrix GPL4047 gene chip probes upregulated in cells harvested after 30′ in vivo compared to stationary phase cells. Columns 1 through 16 give the Affymetrix probe identifier, the log2 probe intensities for the stationary phase, early log phase, 30′, 120, and 360′ in vivo samples, the *S. aureus* strain COL, N315, MSRA, MSSA, Mu50, and MW2 gene designations, the gene name, the gene product function, the TIGR main role category and TIGR subcategory role, respectively. The following 4 columns give the fold change difference from stationary phase for the early log phase (LB), 30′ (30′ in vivo), 120′ (120′ in vivo) and 360′ (360′ in vivo) derived cells. The values are color coded with green indicating significantly downregulated and dark gold indicating significantly upregulated. White indicates no significant difference.
(PDF)

Table S6 Table of Affymetrix GPL4047 gene chip probes downregulated in cells harvested after 30′ in vivo compared to stationary phase cells. Columns 1 through 16 give the Affymetrix probe identifier, the log2 probe intensities for the stationary phase, early log phase, 30′, 120, and 360′ in vivo samples, the *S. aureus* strain COL, N315, MSRA, MSSA, Mu50, and MW2 gene designations, the gene name, the gene product function, the TIGR main role category and TIGR subcategory role, respectively. The following 4 columns give the fold change difference from stationary phase for the early log phase (LB), 30′ (30′ in vivo), 120′ (120′ in vivo) and 360′ (360′ in vivo) derived cells. The values are color coded with green indicating significantly downregulated and dark gold indicating significantly upregulated. White indicates no significant difference.
(PDF)

## References

[1] Wertheim HF, Vos MC, Ott A, van Belkum A, Voss A, et al (2004) Risk and outcome of nosocomial Staphylococcus aureus bacteraemia in nasal carriers versus non-carriers. Lancet 364: 703–705. 15325835 10.1016/S0140-6736(04)16897-9

[2] Chambers HF, Deleo FR (2009) Waves of resistance: Staphylococcus aureus in the antibiotic era. Nat Rev Microbiol 7: 629–641. 19680247 10.1038/nrmicro2200 PMC2871281

[3] Oie S, Kamiya A (1996) Survival of methicillin-resistant Staphylococcus aureus (MRSA) on naturally contaminated dry mops. J Hosp Infect 34: 145–149. 8910757 10.1016/s0195-6701(96)90140-1

[4] O’Connell NH, Humphreys H (2000) Intensive care unit design and environmental factors in the acquisition of infection. J Hosp Infect 45: 255–262. 10981659 10.1053/jhin.2000.0768

[5] Crossley K, Landesman B, Zaske D (1979) An outbreak of infections caused by strains of Staphylococcus aureus resistant to methicillin and aminoglycosides. II. Epidemiologic studies. J Infect Dis 139: 280–287. 255553 10.1093/infdis/139.3.280

[6] Kumari DN, Haji TC, Keer V, Hawkey PM, Duncanson V, et al (1998) Ventilation grilles as a potential source of methicillin-resistant Staphylococcus aureus causing an outbreak in an orthopaedic ward at a district general hospital. J Hosp Infect 39: 127–133. 9651857 10.1016/s0195-6701(98)90326-7

[7] Bore E, Langsrud S, Langsrud O, Rode TM, Holck A (2007) Acid-shock responses in Staphylococcus aureus investigated by global gene expression analysis. Microbiology 153: 2289–2303. 17600073 10.1099/mic.0.2007/005942-0

[8] Miller M, Cook HA, Furuya EY, Bhat M, Lee MH, et al (2009) Staphylococcus aureus in the community: colonization versus infection. PLoS ONE 4: e6708. 19693269 10.1371/journal.pone.0006708 PMC2724739

[9] Boyce JM, Havill NL, Maria B (2005) Frequency and possible infection control implications of gastrointestinal colonization with methicillin-resistant Staphylococcus aureus. J Clin Microbiol 43: 5992–5995. 16333087 10.1128/JCM.43.12.5992-5995.2005 PMC1317179

[10] Ten Broeke-Smits NJ, Kummer JA, Bleys RL, Fluit AC, Boel CH (2010) Hair follicles as a niche of Staphylococcus aureus in the nose; is a more effective decolonisation strategy needed? J Hosp Infect 76: 211–214. 20864209 10.1016/j.jhin.2010.07.011

[11] Andrews WW, Schelonka R, Waites K, Stamm A, Cliver SP, et al (2008) Genital tract methicillin-resistant Staphylococcus aureus: risk of vertical transmission in pregnant women. Obstet Gynecol 111: 113–118. 18165399 10.1097/01.AOG.0000298344.04916.11

[12] Chatterjee I, Herrmann M, Proctor RA, Peters G, Kahl BC (2007) Enhanced Post-Stationary-Phase Survival of a Clinical Thymidine-Dependent Small-Colony Variant of Staphylococcus aureus Results from Lack of a Functional Tricarboxylic Acid Cycle. J Bacteriol 189: 2936–2940. 17259321 10.1128/JB.01444-06 PMC1855812

[13] Kahl BC, Belling G, Becker P, Chatterjee I, Wardecki K, et al (2005) Thymidine-dependent Staphylococcus aureus small-colony variants are associated with extensive alterations in regulator and virulence gene expression profiles. Infect Immun 73: 4119–4126. 15972501 10.1128/IAI.73.7.4119-4126.2005 PMC1168585

[14] Seggewiss J, Becker K, Kotte O, Eisenacher M, Yazdi MRK, et al (2006) Reporter Metabolite Analysis of Transcriptional Profiles of a Staphylococcus aureus Strain with Normal Phenotype and Its Isogenic hemB Mutant Displaying the Small-Colony-Variant Phenotype. J Bacteriol 188: 7765–7777. 16980462 10.1128/JB.00774-06 PMC1636313

[15] Weinrick B, Dunman PM, McAleese F, Murphy E, Projan SJ, et al (2004) Effect of mild acid on gene expression in Staphylococcus aureus. J Bacteriol 186: 8407–8423. 15576791 10.1128/JB.186.24.8407-8423.2004 PMC532443

[16] Palazzolo-Ballance AM, Reniere ML, Braughton KR, Sturdevant DE, Otto M, et al (2008) Neutrophil microbicides induce a pathogen survival response in community-associated methicillin-resistant Staphylococcus aureus. J Immunol 180: 500–509. 18097052 10.4049/jimmunol.180.1.500

[17] Wolf C, Hochgrafe F, Kusch H, Albrecht D, Hecker M, et al (2008) Proteomic analysis of antioxidant strategies of Staphylococcus aureus: diverse responses to different oxidants. Proteomics 8: 3139–3153. 18604844 10.1002/pmic.200701062

[18] Chang W, Small DA, Toghrol F, Bentley WE (2006) Global Transcriptome Analysis of Staphylococcus aureus Response to Hydrogen Peroxide. J Bacteriol 188: 1648–1659. 16452450 10.1128/JB.188.4.1648-1659.2006 PMC1367260

[19] Anderson KL, Roberts C, Disz T, Vonstein V, Hwang K, et al (2006) Characterization of the Staphylococcus aureus heat shock, cold shock, stringent, and SOS responses and their effects on log-phase mRNA turnover. J Bacteriol 188: 6739–6756. 16980476 10.1128/JB.00609-06 PMC1595530

[20] Utaida S, Dunman PM, Macapagal D, Murphy E, Projan SJ, et al (2003) Genome-wide transcriptional profiling of the response of Staphylococcus aureus to cell-wall-active antibiotics reveals a cell-wall-stress stimulon. Microbiology 149: 2719–2732. 14523105 10.1099/mic.0.26426-0

[21] McAleese F, Wu SW, Sieradzki K, Dunman P, Murphy E, et al (2006) Overexpression of genes of the cell wall stimulon in clinical isolates of Staphylococcus aureus exhibiting vancomycin-intermediate- S. aureus-type resistance to vancomycin. J Bacteriol 188: 1120–1133. 16428416 10.1128/JB.188.3.1120-1133.2006 PMC1347359

[22] Michel A, Agerer F, Hauck CR, Herrmann M, Ullrich J, et al (2006) Global regulatory impact of ClpP protease of Staphylococcus aureus on regulons involved in virulence, oxidative stress response, autolysis, and DNA repair. J Bacteriol 188: 5783–5796. 16885446 10.1128/JB.00074-06 PMC1540084

[23] Rogasch K, Ruhmling V, Pane-Farre J, Hoper D, Weinberg C, et al (2006) Influence of the two-component system SaeRS on global gene expression in two different Staphylococcus aureus strains. J Bacteriol 188: 7742–7758. 17079681 10.1128/JB.00555-06 PMC1636327

[24] Resch A, Rosenstein R, Nerz C, Gotz F (2005) Differential gene expression profiling of Staphylococcus aureus cultivated under biofilm and planktonic conditions. Appl Environ Microbiol 71: 2663–2676. 15870358 10.1128/AEM.71.5.2663-2676.2005 PMC1087559

[25] Coulter SN, Schwan WR, Ng EY, Langhorne MH, Ritchie HD, et al (1998) Staphylococcus aureus genetic loci impacting growth and survival in multiple infection environments. Mol Microbiol 30: 393–404. 9791183 10.1046/j.1365-2958.1998.01075.x

[26] Garzoni C, Francois P, Huyghe A, Couzinet S, Tapparel C, et al (2007) A global view of Staphylococcus aureus whole genome expression upon internalization in human epithelial cells. BMC Genomics 8: 171. 17570841 10.1186/1471-2164-8-171 PMC1924023

[27] Yarwood JM, McCormick JK, Paustian ML, Kapur V, Schlievert PM (2002) Repression of the Staphylococcus aureus accessory gene regulator in serum and in vivo. J Bacteriol 184: 1095–1101. 11807070 10.1128/jb.184.4.1095-1101.2002 PMC134826

[28] Pragman AA, Schlievert PM (2004) Virulence regulation in Staphylococcus aureus: the need for in vivo analysis of virulence factor regulation. FEMS Immunol Med Microbiol 42: 147–154. 15364098 10.1016/j.femsim.2004.05.005

[29] Ray B, Ballal A, Manna AC (2009) Transcriptional variation of regulatory and virulence genes due to different media in Staphylococcus aureus. Microb Pathog 47: 94–100. 19450677 10.1016/j.micpath.2009.05.001

[30] Ventura CL, Higdon R, Kolker E, Skerrett SJ, Rubens CE (2008) Host airway proteins interact with Staphylococcus aureus during early pneumonia. Infect Immun 76: 888–898. 18195024 10.1128/IAI.01301-07 PMC2258841

[31] Skerrett SJ, Liggitt HD, Hajjar AM, Wilson CB (2004) Cutting Edge: Myeloid Differentiation Factor 88 Is Essential for Pulmonary Host Defense against Pseudomonas aeruginosa but Not Staphylococcus aureus. J Immunol 172: 3377–3381. 15004134 10.4049/jimmunol.172.6.3377

[32] Francois P, Garzoni C, Bento M, Schrenzel J (2007) Comparison of amplification methods for transcriptomic analyses of low abundance prokaryotic RNA sources. J Microbiol Methods 68: 385–391. 17112614 10.1016/j.mimet.2006.09.022

[33] Dunman PM, Mounts W, McAleese F, Immermann F, Macapagal D, et al (2004) Uses of Staphylococcus aureus GeneChips in genotyping and genetic composition analysis. J Clin Microbiol 42: 4275–4283. 15365023 10.1128/JCM.42.9.4275-4283.2004 PMC516287

[34] Barrett T, Troup DB, Wilhite SE, Ledoux P, Evangelista C, et al (2011) NCBI GEO: archive for functional genomics data sets–10 years on. Nucleic Acids Res 39: D1005–1010. 21097893 10.1093/nar/gkq1184 PMC3013736

[35] Enright MC, Day NP, Davies CE, Peacock SJ, Spratt BG (2000) Multilocus sequence typing for characterization of methicillin-resistant and methicillin-susceptible clones of Staphylococcus aureus. J Clin Microbiol 38: 1008–1015. 10698988 10.1128/jcm.38.3.1008-1015.2000 PMC86325

[36] Altschul SF, Gish W, Miller W, Myers EW, Lipman DJ (1990) Basic local alignment search tool. J Mol Biol 215: 403–410. 2231712 10.1016/S0022-2836(05)80360-2

[37] Emmett M, Kloos WE (1975) Amino acid requirements of staphylococci isolated from human skin. Can J Microbiol 21: 729–733. 1125861 10.1139/m75-107

[38] Pattee PA, Neveln DS (1975) Transformation analysis of three linkage groups in Staphylococcus aureus. J Bacteriol 124: 201–211. 1176430 10.1128/jb.124.1.201-211.1975 PMC235883

[39] Adhikari RP, Arvidson S, Novick RP (2007) A nonsense mutation in agrA accounts for the defect in agr expression and the avirulence of Staphylococcus aureus 8325-4 traP:kan. Infect Immun 75: 4534–4540. 17606604 10.1128/IAI.00679-07 PMC1951176

[40] Ziebuhr W, Heilmann C, Gotz F, Meyer P, Wilms K, et al (1997) Detection of the intercellular adhesion gene cluster (ica) and phase variation in Staphylococcus epidermidis blood culture strains and mucosal isolates. Infect Immun 65: 890–896. 9038293 10.1128/iai.65.3.890-896.1997 PMC175065

[41] Cassat JE, Dunman PM, McAleese F, Murphy E, Projan SJ, et al (2005) Comparative genomics of Staphylococcus aureus musculoskeletal isolates. J Bacteriol 187: 576–592. 15629929 10.1128/JB.187.2.576-592.2005 PMC543526

[42] Enright MC, Robinson DA, Randle G, Feil EJ, Grundmann H, et al (2002) The evolutionary history of methicillin-resistant Staphylococcus aureus (MRSA). Proc Natl Acad Sci U S A 99: 7687–7692. 12032344 10.1073/pnas.122108599 PMC124322

[43] Suzuki M, Matsumoto M, Takahashi M, Hayakawa Y, Minagawa H (2009) Identification of the clonal complexes of Staphylococcus aureus strains by determination of the conservation patterns of small genomic islets. J Appl Microbiol 107: 1367–1374. 19426274 10.1111/j.1365-2672.2009.04321.x

[44] Kuroda M, Ohta T, Uchiyama I, Baba T, Yuzawa H, et al (2001) Whole genome sequencing of meticillin-resistant Staphylococcus aureus. Lancet 357: 1225–1240. 11418146 10.1016/s0140-6736(00)04403-2

[45] Bischoff M, Entenza JM, Giachino P (2001) Influence of a functional sigB operon on the global regulators sar and agr in Staphylococcus aureus. J Bacteriol 183: 5171–5179. 11489871 10.1128/JB.183.17.5171-5179.2001 PMC95394

[46] Giachino P, Engelmann S, Bischoff M (2001) Sigma(B) activity depends on RsbU in Staphylococcus aureus. J Bacteriol 183: 1843–1852. 11222581 10.1128/JB.183.6.1843-1852.2001 PMC95078

[47] Shopsin B, Gomez M, Montgomery SO, Smith DH, Waddington M, et al (1999) Evaluation of Protein A Gene Polymorphic Region DNA Sequencing for Typing of Staphylococcus aureus Strains. J Clin Microbiol 37: 3556–3563. 10523551 10.1128/jcm.37.11.3556-3563.1999 PMC85690

[48] Kullik I, Giachino P, Fuchs T (1998) Deletion of the Alternative Sigma Factor sigma B in Staphylococcus aureus Reveals Its Function as a Global Regulator of Virulence Genes. J Bacteriol 180: 4814–4820. 9733682 10.1128/jb.180.18.4814-4820.1998 PMC107504

[49] Ji G, Beavis R, Novick RP (1997) Bacterial interference caused by autoinducing peptide variants. Science 276: 2027–2030. 9197262 10.1126/science.276.5321.2027

[50] Gill SR, Fouts DE, Archer GL, Mongodin EF, Deboy RT, et al (2005) Insights on evolution of virulence and resistance from the complete genome analysis of an early methicillin-resistant Staphylococcus aureus strain and a biofilm-producing methicillin-resistant Staphylococcus epidermidis strain. J Bacteriol 187: 2426–2438. 15774886 10.1128/JB.187.7.2426-2438.2005 PMC1065214

[51] Baba T, Bae T, Schneewind O, Takeuchi F, Hiramatsu K (2008) Genome Sequence of Staphylococcus aureus Strain Newman and Comparative Analysis of Staphylococcal Genomes: Polymorphism and Evolution of Two Major Pathogenicity Islands. J Bacteriol 190: 300–310. 17951380 10.1128/JB.01000-07 PMC2223734

[52] Diep BA, Gill SR, Chang RF, Phan TH, Chen JH, et al (2006) Complete genome sequence of USA300, an epidemic clone of community-acquired meticillin-resistant Staphylococcus aureus. Lancet 367: 731–739. 16517273 10.1016/S0140-6736(06)68231-7

[53] Gillaspy AF, Worrell V, Orvis J, Roe BA, Dyer DW, et al (2006) The *Staphylococcus aureus* NCTC8325 Genome. In: Fischetti V, Novick R, Ferretti J, Portnoy D, Rood J, editors. Gram Positive Pathogens. Washington, DC: ASM Press. 381–412.

[54] Baba T, Takeuchi F, Kuroda M, Yuzawa H, Aoki K, et al (2002) Genome and virulence determinants of high virulence community-acquired MRSA. Lancet 359: 1819–1827. 12044378 10.1016/s0140-6736(02)08713-5

[55] Holden MT, Feil EJ, Lindsay JA, Peacock SJ, Day NP, et al (2004) Complete genomes of two clinical Staphylococcus aureus strains: evidence for the rapid evolution of virulence and drug resistance. Proc Natl Acad Sci U S A 101: 9786–9791. 15213324 10.1073/pnas.0402521101 PMC470752

[56] Seidl K, Muller S, Francois P, Kriebitzsch C, Schrenzel J, et al (2009) Effect of a glucose impulse on the CcpA regulon in Staphylococcus aureus. BMC Microbiology 9: 95. 19450265 10.1186/1471-2180-9-95 PMC2697999

[57] Fillinger S, Boschi-Muller S, Azza S, Dervyn E, Branlant G, et al (2000) Two glyceraldehyde-3-phosphate dehydrogenases with opposite physiological roles in a nonphotosynthetic bacterium. J Biol Chem 275: 14031–14037. 10799476 10.1074/jbc.275.19.14031

[58] Purves J, Cockayne A, Moody PC, Morrissey JA (2010) Comparison of the regulation, metabolic functions, and roles in virulence of the glyceraldehyde-3-phosphate dehydrogenase homologues gapA and gapB in Staphylococcus aureus. Infect Immun 78: 5223–5232. 20876289 10.1128/IAI.00762-10 PMC2981307

[59] Rezacova P, Kozisek M, Moy SF, Sieglova I, Joachimiak A, et al (2008) Crystal structures of the effector-binding domain of repressor Central glycolytic gene Regulator from Bacillus subtilis reveal ligand-induced structural changes upon binding of several glycolytic intermediates. Mol Microbiol 69: 895–910. 18554327 10.1111/j.1365-2958.2008.06318.x PMC2764557

[60] ten Broeke-Smits NJ, Pronk TE, Jongerius I, Bruning O, Wittink FR, et al (2010) Operon structure of Staphylococcus aureus. Nucleic Acids Res 38: 3263–3274. 20150412 10.1093/nar/gkq058 PMC2879529

[61] Seidl K, Stucki M, Ruegg M, Goerke C, Wolz C, et al (2006) Staphylococcus aureus CcpA Affects Virulence Determinant Production and Antibiotic Resistance. Antimicrob Agents Chemother 50: 1183–1194. 16569828 10.1128/AAC.50.4.1183-1194.2006 PMC1426959

[62] Yang SJ, Dunman PM, Projan SJ, Bayles KW (2006) Characterization of the Staphylococcus aureus CidR regulon: elucidation of a novel role for acetoin metabolism in cell death and lysis. Mol Microbiol 60: 458–468. 16573694 10.1111/j.1365-2958.2006.05105.x

[63] Richardson AR, Libby SJ, Fang FC (2008) A nitric oxide-inducible lactate dehydrogenase enables Staphylococcus aureus to resist innate immunity. Science 319: 1672–1676. 18356528 10.1126/science.1155207

[64] Somerville GA, Chaussee MS, Morgan CI, Fitzgerald JR, Dorward DW, et al (2002) Staphylococcus aureus aconitase inactivation unexpectedly inhibits post-exponential-phase growth and enhances stationary-phase survival. Infect Immun 70: 6373–6382. 12379717 10.1128/IAI.70.11.6373-6382.2002 PMC130419

[65] Zhu Y, Xiong YQ, Sadykov MR, Fey PD, Lei MG, et al (2009) Tricarboxylic acid cycle-dependent attenuation of Staphylococcus aureus in vivo virulence by selective inhibition of amino acid transport. Infect Immun 77: 4256–4264. 19667045 10.1128/IAI.00195-09 PMC2747957

[66] Heinemann M, Kummel A, Ruinatscha R, Panke S (2005) In silico genome-scale reconstruction and validation of the Staphylococcus aureus metabolic network. Biotechnol Bioeng 92: 850–864. 16155945 10.1002/bit.20663

[67] Schlag S, Fuchs S, Nerz C, Gaupp R, Engelmann S, et al (2008) Characterization of the oxygen-responsive NreABC regulon of Staphylococcus aureus. J Bacteriol 190: 7847–7858. 18820014 10.1128/JB.00905-08 PMC2583599

[68] Choudhry AE, Mandichak TL, Broskey JP, Egolf RW, Kinsland C, et al (2003) Inhibitors of pantothenate kinase: novel antibiotics for staphylococcal infections. Antimicrob Agents Chemother 47: 2051–2055. 12760898 10.1128/AAC.47.6.2051-2055.2003 PMC155856

[69] Newton GL, Arnold K, Price MS, Sherrill C, Delcardayre SB, et al (1996) Distribution of thiols in microorganisms: mycothiol is a major thiol in most actinomycetes. J Bacteriol 178: 1990–1995. 8606174 10.1128/jb.178.7.1990-1995.1996 PMC177895

[70] Pohl K, Francois P, Stenz L, Schlink F, Geiger T, et al (2009) CodY in Staphylococcus aureus: a Regulatory Link between Metabolism and Virulence Gene Expression. J Bacteriol 191: 2953–2963. 19251851 10.1128/JB.01492-08 PMC2681790

[71] Richardson AR, Dunman PM, Fang FC (2006) The nitrosative stress response of Staphylococcus aureus is required for resistance to innate immunity. Mol Microbiol 61: 927–939. 16859493 10.1111/j.1365-2958.2006.05290.x

[72] Pamp SJ, Frees D, Engelmann S, Hecker M, Ingmer H (2006) Spx is a global effector impacting stress tolerance and biofilm formation in Staphylococcus aureus. J Bacteriol 188: 4861–4870. 16788195 10.1128/JB.00194-06 PMC1483011

[73] Pelz A, Wieland KP, Putzbach K, Hentschel P, Albert K, et al (2005) Structure and biosynthesis of staphyloxanthin from Staphylococcus aureus. J Biol Chem 280: 32493–32498. 16020541 10.1074/jbc.M505070200

[74] Cotter PD, Hill C (2003) Surviving the acid test: responses of gram-positive bacteria to low pH. Microbiol Mol Biol Rev 67: 429–453, table of contents. 10.1128/MMBR.67.3.429-453.2003 PMC193868 12966143

[75] Cheung AL, Bayer AS, Zhang G, Gresham H, Xiong YQ (2004) Regulation of virulence determinants in vitro and in vivo in Staphylococcus aureus. FEMS Immunol Med Microbiol 40: 1–9. 14734180 10.1016/S0928-8244(03)00309-2

[76] Vandenesch F, Kornblum J, Novick RP (1991) A temporal signal, independent of agr, is required for hla but not spa transcription in Staphylococcus aureus. J Bacteriol 173: 6313–6320. 1717437 10.1128/jb.173.20.6313-6320.1991 PMC208961

[77] van Schaik W, Abee T (2005) The role of sigmaB in the stress response of Gram-positive bacteria – targets for food preservation and safety. Curr Opin Biotechnol 16: 218–224. 15831390 10.1016/j.copbio.2005.01.008

[78] Sieradzki K, Tomasz A (1998) Suppression of glycopeptide resistance in a highly teicoplanin-resistant mutant of Staphylococcus aureus by transposon inactivation of genes involved in cell wall synthesis. Microb Drug Resist 4: 159–168. 9818967 10.1089/mdr.1998.4.159

[79] Singh VK, Schmidt JL, Jayaswal RK, Wilkinson BJ (2003) Impact of sigB mutation on Staphylococcus aureus oxacillin and vancomycin resistance varies with parental background and method of assessment. Int J Antimicrob Agents 21: 256–261. 12636988 10.1016/s0924-8579(02)00359-x

[80] Lauderdale KJ, Boles BR, Cheung AL, Horswill AR (2009) Interconnections between Sigma B, agr, and Proteolytic Activity in Staphylococcus aureus Biofilm Maturation. Infect Immun 77: 1623–1635. 19188357 10.1128/IAI.01036-08 PMC2663138

[81] Giraudo AT, Cheung AL, Nagel R (1997) The sae locus of Staphylococcus aureus controls exoprotein synthesis at the transcriptional level. Arch Microbiol 168: 53–58. 9211714 10.1007/s002030050469

[82] Liang X, Yu C, Sun J, Liu H, Landwehr C, et al (2006) Inactivation of a Two-Component Signal Transduction System, SaeRS, Eliminates Adherence and Attenuates Virulence of Staphylococcus aureus. Infect Immun 74: 4655–4665. 16861653 10.1128/IAI.00322-06 PMC1539584

[83] Goerke C, Fluckiger U, Steinhuber A, Bisanzio V, Ulrich M, et al (2005) Role of Staphylococcus aureus global regulators sae and sigmaB in virulence gene expression during device-related infection. Infect Immun 73: 3415–3421. 15908369 10.1128/IAI.73.6.3415-3421.2005 PMC1111833

[84] Goerke C, Fluckiger U, Steinhuber A, Zimmerli W, Wolz C (2001) Impact of the regulatory loci agr, sarA and sae of Staphylococcus aureus on the induction of alpha-toxin during device-related infection resolved by direct quantitative transcript analysis. Mol Microbiol 40: 1439–1447. 11442841 10.1046/j.1365-2958.2001.02494.x

[85] Geiger T, Goerke C, Mainiero M, Kraus D, Wolz C (2008) The Virulence Regulator Sae of Staphylococcus aureus: Promoter Activities and Response to Phagocytosis-Related Signals. J Bacteriol 190: 3419–3428. 18344360 10.1128/JB.01927-07 PMC2395011

[86] Novick RP, Jiang D (2003) The staphylococcal saeRS system coordinates environmental signals with agr quorum sensing. Microbiology 149: 2709–2717. 14523104 10.1099/mic.0.26575-0

[87] Schulthess B, Meier S, Homerova D, Goerke C, Wolz C, et al (2009) Functional characterization of the sigmaB-dependent yabJ-spoVG operon in Staphylococcus aureus: role in methicillin and glycopeptide resistance. Antimicrob Agents Chemother 53: 1832–1839. 19223635 10.1128/AAC.01255-08 PMC2681525

[88] Meier S, Goerke C, Wolz C, Seidl K, Homerova D, et al (2007) {sigma}B and the {sigma}B-Dependent arlRS and yabJ-spoVG Loci Affect Capsule Formation in Staphylococcus aureus. Infect Immun 75: 4562–4571. 17635871 10.1128/IAI.00392-07 PMC1951174

[89] Voyich JM, Braughton KR, Sturdevant DE, Whitney AR, Said-Salim B, et al (2005) Insights into mechanisms used by Staphylococcus aureus to avoid destruction by human neutrophils. J Immunol 175: 3907–3919. 16148137 10.4049/jimmunol.175.6.3907

[90] Aziz RK, Kansal R, Aronow BJ, Taylor WL, Rowe SL, et al (2010) Microevolution of group A streptococci in vivo: capturing regulatory networks engaged in sociomicrobiology, niche adaptation, and hypervirulence. PLoS ONE 5: e9798. 20418946 10.1371/journal.pone.0009798 PMC2854683

[91] Dubrac S, Boneca IG, Poupel O, Msadek T (2007) New insights into the WalK/WalR (YycG/YycF) essential signal transduction pathway reveal a major role in controlling cell wall metabolism and biofilm formation in Staphylococcus aureus. J Bacteriol 189: 8257–8269. 17827301 10.1128/JB.00645-07 PMC2168699

[92] van Wamel W, Xiong YQ, Bayer AS, Yeaman MR, Nast CC, et al (2002) Regulation of Staphylococcus aureus type 5 capsular polysaccharides by agr and sarA in vitro and in an experimental endocarditis model. Microb Pathog 33: 73–79. 12202106 10.1006/mpat.2002.0513

[93] Steinhuber A, Goerke C, Bayer MG, Doring G, Wolz C (2003) Molecular architecture of the regulatory Locus sae of Staphylococcus aureus and its impact on expression of virulence factors. J Bacteriol 185: 6278–6286. 14563862 10.1128/JB.185.21.6278-6286.2003 PMC219404

[94] Kubica M, Guzik K, Koziel J, Zarebski M, Richter W, et al (2008) A potential new pathway for Staphylococcus aureus dissemination: the silent survival of S. aureus phagocytosed by human monocyte-derived macrophages. PLoS ONE 3: e1409. 18183290 10.1371/journal.pone.0001409 PMC2169301

[95] Xiong YQ, Willard J, Yeaman MR, Cheung AL, Bayer AS (2006) Regulation of Staphylococcus aureus alpha-toxin gene (hla) expression by agr, sarA, and sae in vitro and in experimental infective endocarditis. J Infect Dis 194: 1267–1275. 17041853 10.1086/508210

[96] Goerke C, Wolz C (2004) Regulatory and genomic plasticity of Staphylococcus aureus during persistent colonization and infection. Int J Med Microbiol 294: 195–202. 15493830 10.1016/j.ijmm.2004.06.013

[97] Shelburne SA, Olsen RJ, Suber B, Sahasrabhojane P, Sumby P, et al (2010) A Combination of Independent Transcriptional Regulators Shapes Bacterial Virulence Gene Expression during Infection. PLoS Pathog 6: e1000817. 20333240 10.1371/journal.ppat.1000817 PMC2841617

[98] Shelburne SA, Davenport MT, Keith DB, Musser JM (2008) The role of complex carbohydrate catabolism in the pathogenesis of invasive streptococci. Trends in Microbiology 16: 318–325. 18508271 10.1016/j.tim.2008.04.002 PMC2975494

[99] Majerczyk CD, Dunman PM, Luong TT, Lee CY, Sadykov MR, et al (2010) Direct Targets of CodY in Staphylococcus aureus. J Bacteriol 192: 2861–2877. 20363936 10.1128/JB.00220-10 PMC2876493

[100] Shaw LN, Aish J, Davenport JE, Brown MC, Lithgow JK, et al (2006) Investigations into sigmaB-modulated regulatory pathways governing extracellular virulence determinant production in Staphylococcus aureus. J Bacteriol 188: 6070–6080. 16923874 10.1128/JB.00551-06 PMC1595368

