# Supplementary figures and images for: Changes in the Staphylococcus aureus Transcriptome during Early Adaptation to the Lung
Source: PLoS One. 2012 Aug 2;7(8):e41329. doi: 10.1371/journal.pone.0041329 (PMC3410880; doi:10.1371/journal.pone.0041329)

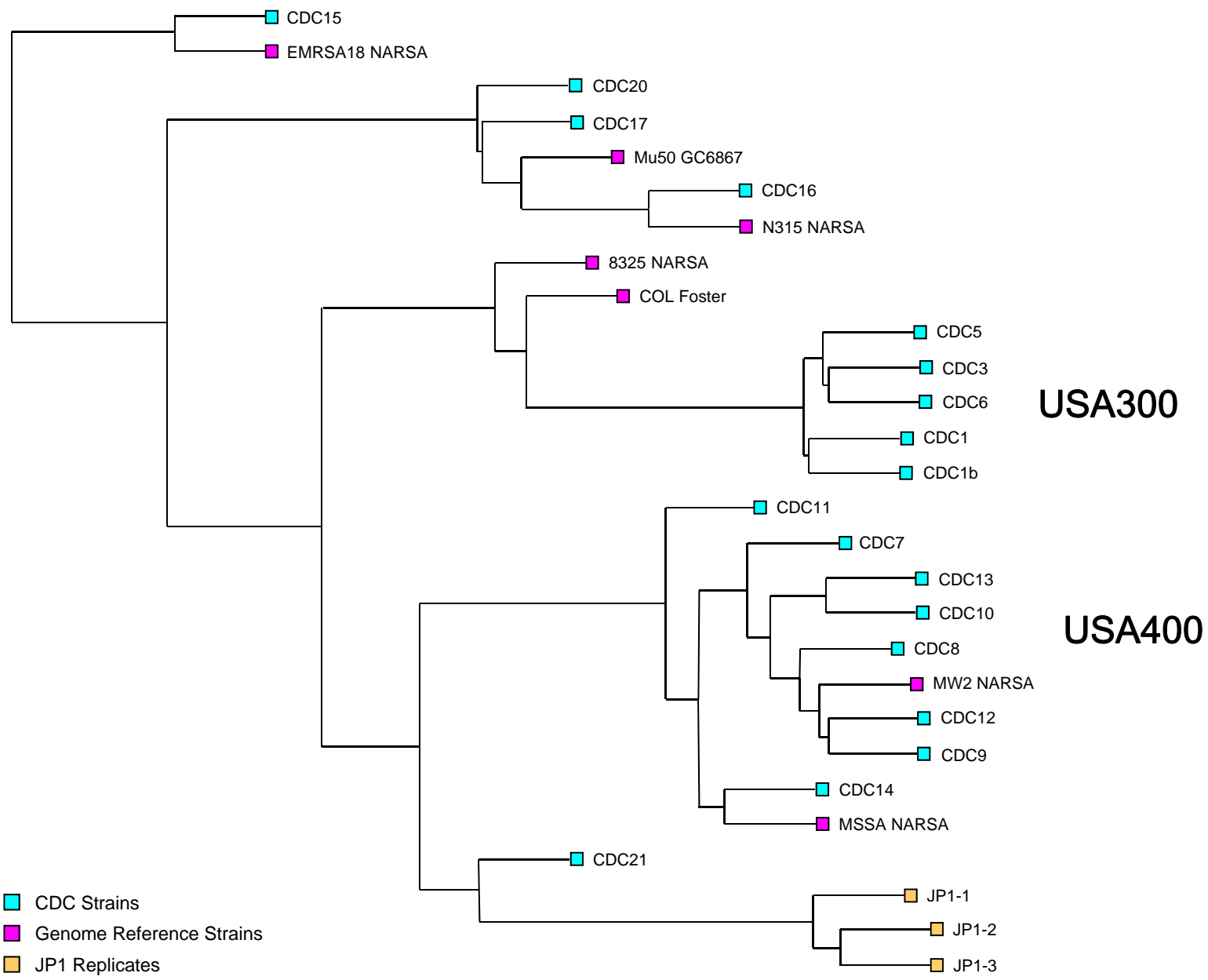

Supplement: Figure S1 — Neighbor-joining tree constructed from Saur2A GeneChip chromosomal DNA hybridizations using Genedata Expression Analyst Pro v3.1.11. Aquamarine terminal nodes indicate CDC S. aureus isolates, whereas magenta colored terminal nodes specify sequenced genome reference strains. Three independent biological replicate hybridizations of JP1 chromosomal DNA are represented in gold. Branches encompassing the USA300 and USA400 lineages are designated. (PDF) [file pone.0041329.s001.pdf]

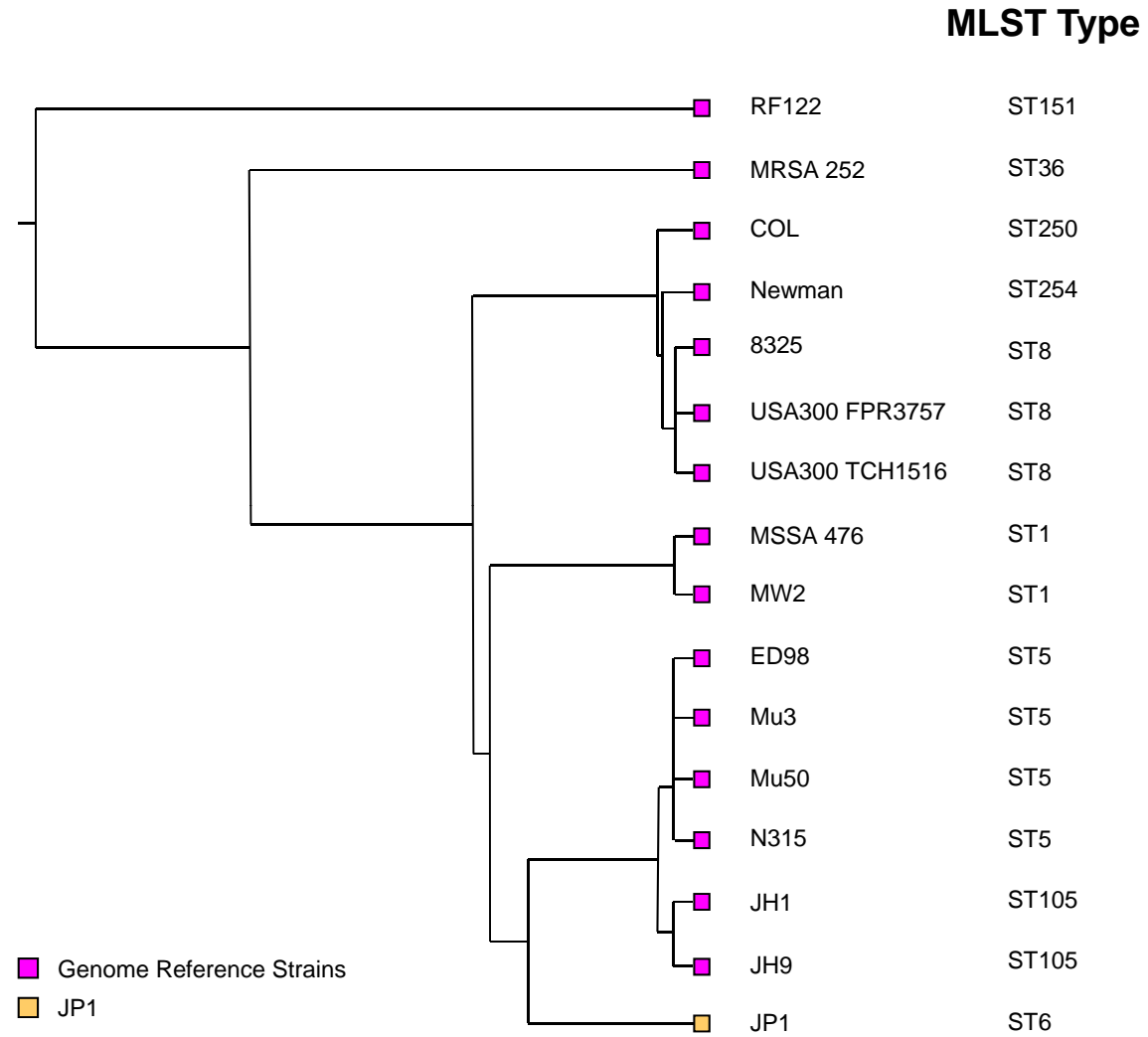

Supplement: Figure S2 — Phylogenetic tree based on concatenated MLST DNA sequences of 15 S . aureus genomic reference strains (indicated by magenta) and strain JP1 (gold) computed using the Multiple Sequence Comparison by Log-Expectation (MUSCLE) algorithm. The MLST type for each strain is given adjacent to the strain name assigned to each leaf node. (PDF) [file pone.0041329.s002.pdf]
