# Supplementary material for: Changes in the Staphylococcus aureus Transcriptome during Early Adaptation to the Lung
Source: PLoS One. 2012 Aug 2;7(8):e41329. doi: 10.1371/journal.pone.0041329 (PMC3410880; doi:10.1371/journal.pone.0041329)
Supplement: Table S1 — Table A: DNA sequences of PCR primers used in the study. Table B: DNA sequences of sequencing primers used in the study. (PDF) [file pone.0041329.s003.pdf]

Supplemental Table 1a:

## PCR PRIMERS

| Operon/ gene | Primer Type | Strand | Primer Name | Sequence (5'-3') | Prod. Size |
|---|---|---|---|---|---|
| sarS | PCR | + | sarS-L | AAAGTGGGGCTTTGAATGTG | 1753 |
| sarS | PCR | - | sarS-R | TGGGCGTACCTTATTCTTGC | |
| rot | PCR | + | rot-L | CATGCTCCATTCATTTGTGC | 1501 |
| rot | PCR | - | rot-R | ATTTAGCCTCATTCGGTTTG | |
| rsb | PCR | + | rsb-L | TCGCGACATTTATGTGGATAC | 3673 |
| rsb | PCR | - | rsb-R | GCTGAATGCAGTAGCTCACC | |
| sarR | PCR | + | sarR-L | TTGCCTACACATGGTTTTGC | 1348 |
| sarR | PCR | - | sarR-R | ATTTTGTGAGCAAGCCATCC | |
| sarUT | PCR | + | sarUT-L | TGCTTCTGCCTCTTGTTGAG | 2424 |
| sarUT | PCR | - | sarUT-R | AAGGTACGGCACCATCAAAC | |
| agr | PCR | + | agr-L | AGATATCCTGCTCGCAGTGG | 4729 |
| agr | PCR | - | agr-R | AAAATTGCGCCATAGGATTG | |
| srr | PCR | + | srr-L | CGATGCTTCTTCGCAAATTC | 3476 |
| srr | PCR | - | srr-R | ACTGTATGCGCTTTCCTGTG | |
| sae | PCR | + | sae-L | TTCCGGGATATTTTTCAACG | 3974 |
| sae | PCR | - | sae-R | TGCCCTCCTCTTATTTTGACTC | |
| sarA | PCR | + | sarA -L | CGAACATTCAGGACATGCAC | 1375 |
| sarA | PCR | - | sarA -R | AAACATTTAATTGCGCTAAATCG | |
| spa (SA0095) | PCR | + | spaSA0095-L | TTGAGGCGTTTCAGAAGTTG | 2479 |
| spa (SA0095) | PCR | - | spaSA0095-R | AAACCTCAGCACATTCAAAGC | |
| psmβ1&2 | PCR | + | psmβ1&2 -L | TCCAGCTGAGCTACCAGGAC | 1326 |
| psmβ1&2 | PCR | - | psmβ1&2 -R | GTGAAACGCCCATTCTTCTG | |
| psmα1-4 | PCR | + | psmα1-4 -L | ATTAGCAGAACGCCAAGACG | 2586 |
| psmα1-4 | PCR | - | psmα1-4 -R | TGTCATACCCCAGCAGAGTG | |

Supplemental Table 1b:

## SEQUENCING PRIMERS

| Operon | Primer Type | Strand | Primer Name | Sequence (5'-3') |
|---|---|---|---|---|
| sarS | Sequencing | + | sarS-L1 | CGTTCTGCAATTTTCTCTCG |
| sarS | Sequencing | - | sarS-R1 | CAGATGATACCAAAAGATAG |
| rot | Sequencing | + | rot-L1 | ATCCCAACAATCCCGAAAC |
| rot | Sequencing | - | rot-R1 | GCGATTGCAAGTAGAGCAAC |
| rsb | Sequencing | + | rsb-L1 | TCGAATCTATTTATGGCACC |
| rsb | Sequencing | + | rsb-L2 | GCTTCACTAACTGCAATCTTGG |
| rsb | Sequencing | + | rsb-L3 | CGAGTTCCATCTTGTCTCATAGG |
| rsb | Sequencing | + | rsb-L4 | TCGTGTTTGTGAACTGATTCC |
| rsb | Sequencing | + | rsb-L5 | CCACGATTAGTTGCCTCCTC |
| rsb | Sequencing | - | rsb-R1 | GCGGCACAAAAAGTAAGTGG |
| rsb | Sequencing | - | rsb-R2 | CATAAACATATGCACCCACAAG |
| rsb | Sequencing | - | rsb-R3 | ACGGAGGTCGAATAACATGC |
| rsb | Sequencing | - | rsb-R4 | GGCGAAAGAGTCGAAATCAG |
| rsb | Sequencing | - | rsb-R5 | GCAATGGAAATGGGACAAAG |
| rsb | Sequencing | - | rsb-R6 | CGAAGTTAAAGTCGGTGGAG |
| sarR | Sequencing | + | sarR-L1 | CCTCTGATGCACATCTTGTTG |
| sarR | Sequencing | - | sarR-R1 | GCTAAGTGCTCAGAGTTCAAACC |
| sarUT | Sequencing | + | sarUT-L1 | TGCTCTTGCTTTTCAAATCATTC |

| | | | | |
|---|---|---|---|---|
| sarUT | Sequencing | + | sarUT-L2 | TGAACGCCGTATTTTTGTTTC |
| sarUT | Sequencing | + | sarUT-L3 | AGAACGATCCCTTGCAGATG |
| sarUT | Sequencing | - | sarUT-R1 | AAAATACGGCGTTCATCTGC |
| sarUT | Sequencing | - | sarUT-R2 | AAGGGATGAAGACTTGATGAATG |
| sarUT | Sequencing | - | sarUT-R3 | TTCAATGCTATTGAGTTGATGG |
| srr | Sequencing | + | srr-L1 | AACGCTGCAATAGGCTGAAC |
| srr | Sequencing | + | srr-L2 | TCCATAATATCATTCGCCATC |
| srr | Sequencing | + | srr-L3 | TTCTGTGCTTTTGTTGGATAGC |
| srr | Sequencing | + | srr-L4 | TATACCCAACGCCCCAGAC |
| srr | Sequencing | - | srr-R1 | GAGCCGGCTAAATAGTGTCG |
| srr | Sequencing | - | srr-R2 | CGTTTAAGAGACCAAGCTACACG |
| srr | Sequencing | - | srr-R3 | CCATTGTCCTTGATGAATCG |
| sarA | Sequencing | + | sarA-L1 | CATCTTGCTCGATACATTTGC |
| sarA | Sequencing | - | sarA-R1 | AGGAAGATAGCCGCATAACG |
| spa | Sequencing | + | spa-L1 | GGCTTGTTATTGTCTTCCTC |
| spa | Sequencing | + | spa-L2 | CATTTGCAGCAGGTGTTACG |
| spa | Sequencing | + | spa-L3 | TTTTAGCTTCTGACAATAGG |
| spa | Sequencing | - | spa-R1 | TGCTAACCTATTGTCAGAAG |
| spa | Sequencing | - | spa-R2 | CAAACGGCACTACTGCTGAC |
| psmβ1&2 | Sequencing | + | psmβ-L1 | CGCAAATACTGTGCAAGCTG |
| psmβ1&2 | Sequencing | - | psmβ-R1 | TTTGTTTATCCGCACAACATC |
| psmα1-4 | Sequencing | + | psmα-L1 | CAAAGCCACCATCCCTATTG |
| psmα1-4 | Sequencing | + | psmα-L2 | AATGGCCCCCTTCAAATAAG |
| psmα1-4 | Sequencing | + | psmα-L3 | AGGTCTCGTCTAGGCAAAGC |
| psmα1-4 | Sequencing | + | psmα-L4 | GGCTTTTTCGGTATTCATGG |
| psmα1-4 | Sequencing | - | psmα-R1 | TGCTTTGCCTAGACGAGACC |
| psmα1-4 | Sequencing | - | psmα-R2 | TTCACATGGGTATCATTGC |
| psmα1-4 | Sequencing | - | psmα-R3 | TTGTGGCGTGTTTTATGTTTG |
| psmα1-4 | Sequencing | - | psmα-R4 | TCAATTTCTTGCGCATTGAC |
| sae | Sequencing | + | sae-L1 | CTGGGGGATATGTTTTACCTG |
| sae | Sequencing | + | sae-L2 | CACCATTATCGGCTCCTTTC |
| sae | Sequencing | + | sae-L3 | CGTAAAACGAGTTCCCTTGG |
| sae | Sequencing | + | sae-L4 | ATTGCAATCTCTCCGAGTGG |
| sae | Sequencing | + | sae-L5 | GGGGCTGTGAAATCATACG |
| sae | Sequencing | + | sae-L6 | TCATGCTAACTCCTCATTTCTTC |
| sae | Sequencing | - | sae-R1 | CCCACTTACTGATCGTGGATG |
| sae | Sequencing | - | sae-R2 | GATGCTAATACCGTGAATGTCC |
| sae | Sequencing | - | sae-R3 | TTCTGAAGGACTACGTGATGG |
| sae | Sequencing | - | sae-R4 | AGCTAATGAACTCGCGCAAC |
| sae | Sequencing | - | sae-R5 | CCATGAGCTCAAACACTTCC |
| agr | Sequencing | + | agr-L1 | CACAAATAAACTCGGATGAAGC |
| agr | Sequencing | + | agr-L2 | CATTCGCGTTGCATTTATTG |
| agr | Sequencing | + | agr-L4 | AAAAGAAGCCCATTCCTGTG |
| agr | Sequencing | + | agr-L5 | CATTGGTAACATCGCAGCTTATAG |
| agr | Sequencing | + | agr-L7 | TGTGCCATTGAAATCACTCC |
| agr | Sequencing | + | agr-L8 | AGGGGCTCACGACCATAC |
| agr | Sequencing | - | agr R1 | GAGGGCAATTTCCATAGGC |
| agr | Sequencing | - | agr-R2 | TTTAGCTTGCTCAAGCACCTC |
| agr | Sequencing | - | agr-R3 | GCTTCATCCGAGTTTATTTGTG |
| agr | Sequencing | - | agr-R4 | TTGAATGAATTGGGCAAATG |
