# Supplementary material for: Changes in the Staphylococcus aureus Transcriptome during Early Adaptation to the Lung
Source: PLoS One. 2012 Aug 2;7(8):e41329. doi: 10.1371/journal.pone.0041329 (PMC3410880; doi:10.1371/journal.pone.0041329)
Supplement: Table S2 — Table of nucleotide differences between S. aureus JP1 and the genetically closest reference strain. The JP1 allele is given in the 1st column, column 2 contains the nearest reference strain, columns 3 and 4 give the start and stop of the region sequenced mapped onto the corresponding reference strain genome, column 6 indicates the number of mismatches and/or gaps between JP1 and the reference strain, column 7 gives the location of the mismatch within the JP1 sequence, column 8 indicates if the mismatch is within the coding of the allele and column 9 indicates if the mismatch creates a synonymous or non- synonymous AA substitution. (PDF) [file pone.0041329.s004.pdf]

Supplemental table 2

Gene/ Operon Name	Closest Neighbor	Start Sequence in Neighbor	End Sequence in Neighbor	Mismatch?	# of Mismatches	Location(s) of Mismatch*	Present in Open Reading Frame	Synonomous/Non Synonomous?
JP1 *psmα*	USA 300_TCH1516	475686	477872	yes	40, 15 gaps	4	no	
JP1 *psmα*						208	no	
JP1 *psmα*						1365	no	
JP1 *psmα*						1414	no	
JP1 *psmα*						1451	no	
JP1 *psmα*						1465	no	
JP1 *psmα*						1472	no	
JP1 *psmα*						1480	no	
JP1 *psmα*						1503	no	
JP1 *psmα*						1553	no	
JP1 *psmα*						1576	no	
JP1 *psmα*						1629	no	
JP1 *psmα*						1676	no	
JP1 *psmα*						1678	no	
JP1 *psmα*						1698	no	
JP1 *psmα*						1707	no	
JP1 *psmα*						1724	no	
JP1 *psmα*						1741	no	
JP1 *psmα*						1743	no	
JP1 *psmα*						1745	no	
JP1 *psmα*						1746	no	
JP1 *psmα*						1748	no	
JP1 *psmα*						1750	no	
JP1 *psmα*						1751	no	
JP1 *psmα*						1755	no	
JP1 *psmα*						1758	no	
JP1 *psmα*						1760	no	
JP1 *psmα*						1761	no	
JP1 *psmα*						1894	no	
JP1 *psmα*						1944	no	
JP1 *psmα*						2056	no	
JP1 *psmα*						2155	no	
JP1 *psmα*						2161	no	
JP1 *psmβ*	NCTC 8325	1088078	1088962	yes	2	41	no	
JP1 *psmβ*						289	no	
JP1 *rot*	MSSA476	1839756	1838796	yes	2	714	yes	Synonomous
JP1 *rot*						913	no	
Jp1 *rsb*	USA 300_TCH1516	2188442	2185235	yes	14	381	yes	Synonomous
Jp1 *rsb*						468	yes	Synonomous
Jp1 *rsb*						1086	yes	Synonomous
Jp1 *rsb*						1729	yes	Synonomous
Jp1 *rsb*						2024	yes	Synonomous
Jp1 *rsb*						2165	yes	Synonomous
Jp1 *rsb*						2718	yes	Synonomous
Jp1 *rsb*						2838	yes	Synonomous
Jp1 *rsb*						2949	yes	Synonomous
Jp1 *rsb*						3012	yes	Synonomous
Jp1 *rsb*						3049	yes	Synonomous
Jp1 *rsb*						3061	no	
Jp1 *rsb*						3175	no	
Jp1 *rsb*						3176	no	
JP1 *sae*	USA 300_TCH1516	783165	779748	yes	10	22	no	
JP1 *sae*						680	no	
JP1 *sae*						801	no	
JP1 *sae*						853	no	
JP1 *sae*						2558	yes	Synonomous
JP1 *sae*						2776	yes	Synonomous
JP1 *sae*						2898	yes	Synonomous
JP1 *sae*						2912	yes	Synonomous
JP1 *sae*						3268	no	
JP1 *sae*						3410	no	
JP1 *sarA*	USA 300_TCH1516	679678	678779	yes	3, 2 gaps	24	no	
JP1 *sarA*						720	no	
JP1 *sarA*						923	no	
JP1 *sarR*	USA 300_TCH1516	2411516	2410536	yes	2	111	no	
JP1 *sarR*						896	no	
JP1 *sarS*	Mu3	125598	124229	yes	1	1303	no	
JP1 *sarUT*	MSSA476	2555691	2553325	yes	8	202	no	

Supplemental table 2

Gene/ Operon Name	Closest Neighbor	Start Sequence in Neighbor	End Sequence in Neighbor	Mismatch?	# of Mismatches	Location(s) of Mismatch*	Present in Open Reading Frame	Synonomous/Non Synonomous?
JP1 *sarUT*						205	no	
JP1 *sarUT*						282	no	
JP1 *sarUT*						318	no	
JP1 *sarUT*						1028	yes	Synonomous
JP1 *sarUT*						1408	no	
JP1 *sarUT*						2089	no	
JP1 *sarUT*						2352	no	
JP1 *spa*	Newman	75250	73247	yes	31, 1 gap	10	no	Synonomous
JP1 *spa*						1328	yes	Synonomous
JP1 *spa*						1352	yes	Synonomous
JP1 *spa*						1361	yes	Synonomous
JP1 *spa*						1362	yes	Synonomous
JP1 *spa*						1363	yes	Synonomous
JP1 *spa*						1376	yes	Synonomous
JP1 *spa*						1386	yes	Synonomous
JP1 *spa*						1387	yes	Synonomous
JP1 *spa*						1391	yes	Synonomous
JP1 *spa*						1394	yes	Synonomous
JP1 *spa*						1409	yes	Synonomous
JP1 *spa*						1410	yes	Synonomous
JP1 *spa*						1411	yes	Synonomous
JP1 *spa*						1415	yes	Synonomous
JP1 *spa*						1418	yes	Synonomous
JP1 *spa*						1433	yes	Synonomous
JP1 *spa*						1434	yes	Synonomous
JP1 *spa*						1435	yes	Synonomous
JP1 *spa*						1439	yes	Synonomous
JP1 *spa*						1442	yes	Synonomous
JP1 *spa*						1458	yes	Synonomous
JP1 *spa*						1459	yes	Synonomous
JP1 *spa*						1463	yes	Synonomous
JP1 *spa*						1466	yes	Synonomous
JP1 *spa*						1472	yes	Synonomous
JP1 *spa*						1482	yes	Synonomous
JP1 *spa*						1490	yes	Synonomous
JP1 *spa*						1496	yes	Synonomous
JP1 *spa*						1508	yes	Synonomous
JP1 *spa*						1511	yes	8 codon insertion
JP1 *srrAB*	Newman	1571047	1568120	yes	3	703	yes	Synonomous
JP1 *srrAB*						2916	no	
JP1 *srrAB*						2918	no	

*Locations of mismatches are from the start of the JP1 sequence
