# Supplementary material for: Changes in the Staphylococcus aureus Transcriptome during Early Adaptation to the Lung
Source: PLoS One. 2012 Aug 2;7(8):e41329. doi: 10.1371/journal.pone.0041329 (PMC3410880; doi:10.1371/journal.pone.0041329)
Supplement: Table S3 — Table of non-hybridizing Affymetrix GPL4047 gene chip probes. Columns 1 through 12 give the Affymetrix probe identifier, the S. aureus strain COL, N315, MSRA, MSSA, Mu50, and MW2 gene designations, the gene name, the GenBank identifier, the GenBank description, the gene function, the TIGR main role category and TIGR subcategory role, respectively. (PDF) [file pone.0041329.s005.pdf]

Systematic	COL	N315	MRSA	MSSA	Mu50	MW2	GENE NAME	GenBank ID	GenBank Desc.	Protein Function	TIGR Main Role	TIGR Sub Role
AB009866-cds25_at								AB009866-cds25	bacteriophage phi PVL (specific_host:Staphylococcus aureus ATCC 49775) proviral DNA.			
AP001553-cds11_at								AB009866-cds33	bacteriophage phi PVL (specific_host:Staphylococcus aureus ATCC 49775) proviral DNA.			
AB009866-cds50_x_at								AB009866-cds50	bacteriophage phi PVL (specific_host:Staphylococcus aureus ATCC 49775) proviral DNA.			
AB047088-cds7_s_at								AB014438-cds7	Staphylococcus aureus (strain:85/3907) DNA.			
AB033763-cds11_at								AB033763-cds11	Staphylococcus aureus (strain:NCTC10442) DNA			
AB033763-cds2_at								AB033763-cds2	Staphylococcus aureus (strain:NCTC10442) DNA			
AB033763-cds20_at								AB033763-cds20	Staphylococcus aureus (strain:NCTC10442) DNA			
AB033763-cds27_at								AB033763-cds27	Staphylococcus aureus (strain:NCTC10442) DNA			
AB033763-cds29_at								AB033763-cds29	Staphylococcus aureus (strain:NCTC10442) DNA			
AB033763-cds4_at								AB033763-cds4	Staphylococcus aureus (strain:NCTC10442) DNA			
AB033763-cds46_at								AB033763-cds46	Staphylococcus aureus (strain:NCTC10442) DNA			
AB033763-cds8_at								AB033763-cds8	Staphylococcus aureus (strain:NCTC10442) DNA			
AB037671-cds10_at								AB037671-cds10	Staphylococcus aureus (strain:85/2082) DNA.			
AB037671-cds11_at								AB037671-cds11	Staphylococcus aureus (strain:85/2082) DNA.			
AB037671-cds21_at								AB037671-cds21	Staphylococcus aureus (strain:85/2082) DNA.			
AB037671-cds23_at								AB037671-cds23	Staphylococcus aureus (strain:85/2082) DNA.			
AB037671-cds28_at								AB037671-cds28	Staphylococcus aureus (strain:85/2082) DNA.			
AB037671-cds46_at								AB037671-cds46	Staphylococcus aureus (strain:85/2082) DNA.			
AB037671-cds47_at								AB037671-cds47	Staphylococcus aureus (strain:85/2082) DNA.			
AB037671-cds49_at								AB037671-cds49	Staphylococcus aureus (strain:85/2082) DNA.			
AB037671-cds52_at								AB037671-cds52	Staphylococcus aureus (strain:85/2082) DNA.			
AB037671-cds53_at								AB037671-cds53	Staphylococcus aureus (strain:85/2082) DNA.			
AB037671-cds54_at								AB037671-cds54	Staphylococcus aureus (strain:85/2082) DNA.			
AB037671-cds55_at								AB037671-cds55	Staphylococcus aureus (strain:85/2082) DNA.			
AB037671-cds56_at								AB037671-cds56	Staphylococcus aureus (strain:85/2082) DNA.			
AB037671-cds57_at								AB037671-cds57	Staphylococcus aureus (strain:85/2082) DNA.			
AB037671-cds59_at								AB037671-cds59	Staphylococcus aureus (strain:85/2082) DNA.			
AB037671-cds6_at								AB037671-cds6	Staphylococcus aureus (strain:85/2082) DNA.			
AB037671-cds60_at								AB037671-cds60	Staphylococcus aureus (strain:85/2082) DNA.			
AB037671-cds61_at								AB037671-cds61	Staphylococcus aureus (strain:85/2082) DNA.			
AB037671-cds62_at								AB037671-cds62	Staphylococcus aureus (strain:85/2082) DNA.			
AB037671-cds63_at								AB037671-cds63	Staphylococcus aureus (strain:85/2082) DNA.			
AB037671-cds66_at								AB037671-cds66	Staphylococcus aureus (strain:85/2082) DNA.			
AB037671-cds67_at								AB037671-cds67	Staphylococcus aureus (strain:85/2082) DNA.			
AB037671-cds68_at								AB037671-cds68	Staphylococcus aureus (strain:85/2082) DNA.			
AB037671-cds69_at								AB037671-cds69	Staphylococcus aureus (strain:85/2082) DNA.			
AB037671-cds7_at								AB037671-cds7	Staphylococcus aureus (strain:85/2082) DNA.			
AB037671-cds70_at								AB037671-cds70	Staphylococcus aureus (strain:85/2082) DNA.			
AB037671-cds80_at								AB037671-cds80	Staphylococcus aureus (strain:85/2082) DNA.			
AB037671-cds81_at								AB037671-cds81	Staphylococcus aureus (strain:85/2082) DNA.			
AB037671-cds85_at								AB037671-cds85	Staphylococcus aureus (strain:85/2082) DNA.			
AB037671-cds87_at								AB037671-cds87	Staphylococcus aureus (strain:85/2082) DNA.			
AB047089-cds3_x_at								AB047089-cds3	Staphylococcus aureus (strain:85/3907) DNA.			
AB047089-cds4_at								AB047089-cds4	Staphylococcus aureus (strain:85/3907) DNA.			
AF051916-cds2_at								AF051916-cds2	Gene:rep;Product:replication protein Rep			
AF051917-cds10_at								AF051917-cds10	Gene:;Product:putative membrane protein;Note:Orf77			
AF051917-cds11_at								AF051917-cds11	Gene:;Product:unknown;Note:Orf346			
AF051917-cds12_at								AF051917-cds12	Gene:;Product:unknown;Note:Orf109			
AF051917-cds13_at								AF051917-cds13	Gene:;Product:unknown;Note:Orf86			
AF051917-cds14_at								AF051917-cds14	Gene:rep;Product:putative replication initiation protein Rep			
AF051917-cds16_at								AF051917-cds16	Gene:;Product:membrane protein;Note:Orf248			
AF051917-cds36_at								AF051917-cds36	Gene:;Product:unknown;Note:Orf55; possibly truncated by IS257 transposon			
AF051917-cds38_at								AF051917-cds38	Gene:rep(RC);Product:putative replication initiation protein Rep(RC)			
AF051917-cds7_at								AF051917-cds7	Gene:;Product:LtrC-like protein;Note:Orf575; similar to Lactococcus lactis pRS01 LtrC			
AF051917-cds9_at								AF051917-cds9	Gene:;Product:unknown;Note:Orf90			
AF053140-cds2_at								AF053140-cds2	Gene:sej;Product:enterotoxin J			
AF077865-cds1_at								AF077865-cds1	Gene:ear;Product:unknown			
AF117258-cds1_at								AF117258-cds1	Gene:repE;Product:replication protein RepE;Note:similar to RepE from pAMbeta1			
AF117258-cds2_at								AF117258-cds2	Gene:;Product:unknown;Note:OrfA; contains four hydrophobic domains			
AF117258-cds3_at								AF117258-cds3	Gene:res;Product:resolvase;Note:Res; similar to resolvase from pAMbeta1			
AF117259-cds1_at								AF117259-cds1	Gene:repX;Product:replication protein			
AF117259-cds2_at								AF117259-cds2	Gene:;Product:unknown;Note:OrfB			
AF147744-cds1_at								AF147744-cds1	Gene:;Product:lantibiotic structural protein alpha;Note:SacaA			
AF147744-cds2_at								AF147744-cds2	Gene:;Product:lantibiotic structural protein beta;Note:SacbA			
AF147744-cds3_at								AF147744-cds3	Gene:;Product:lantibiotic modifying enzyme;Note:SacM1			
AF147744-cds4_at								AF147744-cds4	Gene:;Product:transporter;Note:SacT			
AF167161-cds1_at								AF167161-cds1	Gene:;Product:unknown;Note:orf1			
AF167161-cds2_at								AF167161-cds2	Gene:;Product:unknown;Note:orf2			
WAN014INJ_at								AF167161-cds6	Gene:sin;Product:recombinase			
AF167161-cds7_at								AF167161-cds7	Gene:;Product:unknown;Note:orf4			
AF186237-cds1_at								AF186237-cds1	Gene:vgaA variant;Product:ABC protein VgaA variant			
AF203376-cds1_at								AF203376-cds1	Gene:orf245;Product:replication-associated protein;Note:Orf245			
AF203376-cds2_at								AF203376-cds2	Gene:rep;Product:replication initiation protein;Note:Rep			
AF203377-cds1_at								AF203377-cds1	Gene:orf256;Product:replication-associated protein;Note:Orf256			
AF203377-cds2_at								AF203377-cds2	Gene:rep;Product:replication initiation protein;Note:Rep			
AF210055-cds1_at								AF210055-cds1	Gene:agrB;Product:AgrB;Note:processing protein for AgrD			
AF217235-cds18_at								AF217235-cds18	Gene:;Product:Orf18			
AF217235-cds19_at								AF217235-cds19	Gene:;Product:Orf19			
AF217235-cds20_at								AF217235-cds20	Gene:;Product:Orf20			
AF217235-cds21_at								AF217235-cds21	Gene:;Product:integrase-like protein			
AF217235-cds6_at								AF217235-cds6	Gene:;Product:Orf6			
AF217235-cds8_x_at								AF217235-cds8	Gene:;Product:Orf8			
AF217235-cds9_at								AF217235-cds9	Gene:;Product:Orf9			
AF288402-cds1-seg1_at								AF288402-cds1-seq1	Gene:Bap;Product:biofilm-associated surface protein			
AF288402-cds1-seg2_at								AF288402-cds1-seq2	Gene:Bap;Product:biofilm-associated surface protein			
AJ243790-cds1_at								AJ132841-cds1	Gene:mapN;Product:MapN protein			
AJ277173-cds1_at								AJ277173-cds1	Gene:;Product:ADP-ribosyltransferase;Note:C3-like			
AJ309178-cds1_at								AJ309178-cds1	Gene:coa;Product:coagulase			
AJ309180-cds1_at								AJ309180-cds1	Gene:coa;Product:coagulase			
AJ309181-cds1_at								AJ309181-cds1	Gene:coa;Product:coagulase			
AJ309182-cds1_at								AJ309182-cds1	Gene:coa;Product:coagulase			
AJ309184-cds1_at								AJ309184-cds1	Gene:coa;Product:coagulase			
AJ309185-cds1_at								AJ309185-cds1	Gene:coa;Product:coagulase			
AJ309190-cds1_at								AJ309190-cds1	Gene:coa;Product:coagulase			
AJ309191-cds1_x_at								AJ309191-cds1	Gene:coa;Product:coagulase			
AJ311975-cds1_at								AJ311975-cds1	Gene:coa;Product:coagulase			
AJ311976-cds1_at								AJ311976-cds1	Gene:coa;Product:coagulase			
AJ311977-cds1_at								AJ311977-cds1	Gene:coa;Product:coagulase			
AP001553-cds10_at								AP001553-cds10	bacteriophage phi ETA (specific_host:Staphylococcus aureus E-1) DNA.			
AP001553-cds12_at								AP001553-cds12	bacteriophage phi ETA (specific_host:Staphylococcus aureus E-1) DNA.			
AP001553-cds19_x_at								AP001553-cds19	bacteriophage phi ETA (specific_host:Staphylococcus aureus E-1) DNA.			
AP001553-cds2_at								AP001553-cds2	bacteriophage phi ETA (specific_host:Staphylococcus aureus E-1) DNA.			

Systematic	COL	N315	MRSA	MSSA	Mu50	MW2	GENE NAME	GenBank ID	GenBank Desc.	Protein Function	TIGR Main Role	TIGR Sub Role
AP001553-cds21_at								AP001553-cds21	bacteriophage phi ETA (specific_host:Staphylococcus aureus E-1) DNA.			
AP001553-cds27_at								AP001553-cds27	bacteriophage phi ETA (specific_host:Staphylococcus aureus E-1) DNA.			
AP001553-cds3_at								AP001553-cds3	bacteriophage phi ETA (specific_host:Staphylococcus aureus E-1) DNA.			
AP001553-cds31_at								AP001553-cds31	bacteriophage phi ETA (specific_host:Staphylococcus aureus E-1) DNA.			
AP001553-cds5_at								AP001553-cds5	bacteriophage phi ETA (specific_host:Staphylococcus aureus E-1) DNA.			
AP001553-cds54_at								AP001553-cds54	bacteriophage phi ETA (specific_host:Staphylococcus aureus E-1) DNA.			
AP001553-cds57_at								AP001553-cds57	bacteriophage phi ETA (specific_host:Staphylococcus aureus E-1) DNA.			
AP001553-cds6_at								AP001553-cds6	bacteriophage phi ETA (specific_host:Staphylococcus aureus E-1) DNA.			
AP001553-cds61_at								AP001553-cds61	bacteriophage phi ETA (specific_host:Staphylococcus aureus E-1) DNA.			
WAN014HKY_at								AP001553-cds66	bacteriophage phi ETA (specific_host:Staphylococcus aureus E-1) DNA.			
AP001553-cds8_at								AP001553-cds8	bacteriophage phi ETA (specific_host:Staphylococcus aureus E-1) DNA.			
AP001553-cds9_at								AP001553-cds9	bacteriophage phi ETA (specific_host:Staphylococcus aureus E-1) DNA.			
AY029184-cds1_at								AY029184-cds1	Staphylococcus aureus.			
D83951-cds2_at								D83951-cds2	Staphylococcus aureus (strain:P83) DNA.			
J01763-cds1_at								J01763-cds1	Plasmid pSN2 DNA.			
J03947-cds1_at								J03947-cds1	S.aureus (strain BM4611) DNA.			
L43082-cds1_at								L43082-cds1	Staphylococcus aureus.			
M17348-cds1_at								M17348-cds1	Plasmid pRW001 DNA.			
M17990-cds1_at								M17990-cds1	Plasmid pE5 DNA.			
M18086-cds1_s_at								M18086-cds1	Staphylococcus aureus (strain SK982) DNA.			
M21319-cds1_at								M21319-cds1	S.aureus (strain FRI918) DNA			
M32470-cds1_at								M32470-cds1	S.aureus (strain 3AI) DNA.			
M32470-cds2_at								M32470-cds2	S.aureus (strain 3AI) DNA.			
M63917-cds1_at								M63917-cds1	S.aureus DNA.			
AF282215-cds2_at									Staphylococcus aureus GENE=""agrB""			
AF282215-cds4_at									Staphylococcus aureus Bacteria; GENE=""agrC""			
WAN014IY1_at									Staphylococcus epidermidis Bacteria; GENE=""thyE""			
U10927-cds1_at								U10927-cds1	Gene:capA;Product:CapA			
U10927-cds10_at								U10927-cds10	Gene:capJ;Product:CapJ			
U10927-cds11_at								U10927-cds11	Gene:capK;Product:CapK			
U10927-cds12_at								U10927-cds12	Gene:capL;Product:CapL			
U10927-cds13_at								U10927-cds13	Gene:capM;Product:CapM			
U10927-cds2_at								U10927-cds2	Gene:capB;Product:CapB			
U10927-cds3_at								U10927-cds3	Gene:capC;Product:CapC			
U10927-cds4_at								U10927-cds4	Gene:capD;Product:CapD			
U10927-cds5_at								U10927-cds5	Gene:capE;Product:CapE			
U10927-cds6_at								U10927-cds6	Gene:capF;Product:CapF			
U10927-cds7_at								U10927-cds7	Gene:capG;Product:CapG			
U10927-cds8_at								U10927-cds8	Gene:capH;Product:CapH			
U10927-cds9_at								U10927-cds9	Gene:capI;Product:CapI			
U19459-cds1_at								U19459-cds1	Gene:vat B;Product:VAT B;Note:acetyltransferase			
U31979-cds4_at								U31979-cds4	Gene:aroB;Product:5-dehdroquinate synthase;Note:5-dehydroquinate hydrolyase			
U35036-cds4_at								U35036-cds4	Gene:pre;Product:Pre protein			
U38429-cds3_at								U38429-cds3	Gene:pre;Product:recombination protein			
U50077-cds2_x_at								U50077-cds2	Staphylococcus aureus strain=SA2. Gene:qacC'			
U73025-cds1_at								U73025-cds1	Gene:;Product:unknown;Note:orfx			
U73026-cds1_at								U73026-cds1	Gene:sat4;Product:;Note:streptothricine-acetyl-transferase			
U73027-cds1_at								U73027-cds1	Gene:;Product:unknown;Note:orfz.pep			
U81980-cds2_at								U81980-cds2	Gene:qac;Product:			
U82085-cds1_at								U82085-cds1	Gene:vgaB;Product:pristinamycin resistance protein VgaB			
U93688-cds10_at								U93688-cds10	Gene:;Product:;Note:orf10			
U93688-cds12_at								U93688-cds12	Gene:;Product:;Note:orf12			
WAN014HN5_at								U93688-cds14	Gene:;Product:;Note:orf14			
U93688-cds15_at								U93688-cds15	Gene:;Product:orf15			
U93688-cds8_at								U93688-cds8	Gene:;Product:;Note:orf8			
U93688-cds9_at								U93688-cds9	Gene:;Product:;Note:orf9			
WAN014HMD_at								U96609-cds1	Gene:rep;Product:replication protein;Note:REP			
WAN014HME_at								U96609-cds2	Gene:;Product:;Note:ORF64			
WAN014HMH_at								X02166-cds1	Gene:;Product:			
WAN014HMI_s_at								X02166-cds4	Gene:;Product:;Note:pot. reading-frame A (aa 1-315)			
WAN014HMM_at								X06627-cds1	Gene:;Product:ORF (rlx)			
WAN014HNB_at								X12831-cds1	Gene:;Product:;Note:ORF 2 (AA 1 - 236)			
WAN014HMW_at								X59477-cds1	Gene:mupirocin resistence gene			
WAN014A7M-seg2_at												
WAN014A7O-seg2_at	SA2150				SAV2160	MW2086						
WAN014A81-5_at	SA0015	SA0014	SAR0015	SAS0015	SAV0015	MW0015	rplI					
WAN014FRL_at												
WAN014FRZ_at												
WAN014FS4_at												
WAN014FUK_at		SAV085										
WAN014FWO_at												
WAN014G0E_at												
WAN014G0H_at												
WAN014G0I_at												
WAN014GF2_at												
WAN014GFJ_at												
WAN014GFK_at												
WAN014GG5_at												
WAN014GJX_at												
WAN014GMK_at												
WAN014GMM_at												
WAN014GMN_at												
WAN014GPE_at												
WAN014GSD_at												
WAN014GT2_at												
WAN014GWJ_at												
WAN014HOX_at												
WAN014HOY_at												
WAN014HP4_at												
WAN014HPV_at												
WAN014HX2_at												
WAN014HXR_at												
WAN014HZ3_at												
WAN014HZ6_at												
WAN014HZ7_at												
WAN014HZ8_at												
WAN014HZ9_at												
WAN014I7K-seg2_at	SA1472				SAV1435	MW1324	ebh					
WAN014ILF_at												
WAN014ILY_x_at												
WAN014INS_at												
WAN014IQB_at												

Supplemental table 3

Systematic	COL	N315	MRSA	MSSA	Mu50	MW2	GENE NAME	GenBank ID	GenBank Desc.	Protein Function	TIGR Main Role	TIGR Sub Role
WAN014IQC_at												
WAN014ISL_at												
WAN014ISS-seg1_at	SA0379		SAR1507	SAS0944		MW1390						
WAN014IX7_at		SAV197										
WAN014IY2_at												
WAN014IYA_at												
WAN014IYB_at												
WAN014IYD_x_at												
WAN014IYG_at												
WAN014IYH_at												
WAN014IYI_at												
WAN01A54R_s_at												
WAN01A551_x_at												
WAN01A55C_at												
WAN01A57W_x_at												
WAN01A57X_x_at												
WAN01A72A_at												
WAN01A72I_s_at												
WAN01A7HH_at												
WAN01A7IM_x_at												
WAN01BTG2_at												
WAN01BTHY_at												
WAN01BTOS_at												
WAN01BTP0_at												
WAN01BTQB_at												
WAN01BTRL_at												
WAN01BTU3_at												
WAN01BTUI_at												
WAN01BTWI_at												
WAN01BTWN_at												
WAN01BTWO_at												
WAN01BTWP_at												
WAN01BTWV_at												
WAN01BTX7_x_at												
WAN01BTZH_at												
WAN01BU30_at												
WAN014GRN_at	SA0029	SA0029			SAV0038	MW0028				HMG-CoA synthase	Biosynthesis of cofactors, prosthetic groups,	Other
WAN014HGL_at		SA0040	SAR0041		SAV0043			AB037671-cds31	Staphylococcus aureus (strain:85/2082) DNA.	methicillin resistance regulatory protein	Cell envelope	Biosynthesis and degradation of surface polysaccharides a
AB037671-cds32_at			SAR0040		SAV0042	MW0032		AB037671-cds32	Staphylococcus aureus (strain:85/2082) DNA.	Methicillin resistance mecR1 protein	Cell envelope	Biosynthesis and degradation of surface polysaccharides a
WAN014FR8_at	SA0143	SA0151			SAV0156		cap5H	U77308-cds1	Staphylococcus aureus.	cap5H protein	Cell envelope	Biosynthesis and degradation of surface polysaccharides a
WAN014FSL_at	SA0145	SA0153			SAV0158		cap5J	U81973-cds10	Gene:cap5H;Product:O-acetyl transferase;	cap5J protein	Cell envelope	Biosynthesis and degradation of surface polysaccharides a
WAN014FTD_at	SA0146	SA0154			SAV0159		cap5K	U81973-cds11	Gene:cap5J;Product:Cap5J	cap5K protein	Cell envelope	Biosynthesis and degradation of surface polysaccharides a
WAN014FRP_at	SA0144	SA0152			SAV0157		cap5I	U81973-cds9	Gene:cap5K;Product:Cap5K	cap5I protein	Cell envelope	Biosynthesis and degradation of surface polysaccharides a
WAN014GKG_at		SA1808							Gene:cap5I;Product:Cap5I	glycosyl transferase, group 2 family protein domain protein	Cell envelope	Biosynthesis and degradation of surface polysaccharides a
WAN014HWA_at			SAR2299							Divergent AAA domain family	Cell envelope	Biosynthesis and degradation of surface polysaccharides a
WAN014GUL_at	SA0033	SA0038	SAR0039		SAV0041	MW0031	pbp2	AB037671-cds33	Staphylococcus aureus (strain:85/2082) DNA.	penicillin-binding protein 2	Cell envelope	Biosynthesis of murein sacculus and peptidoglycan
WAN014GKW_at	SA0054									Mur ligase family protein	Cell envelope	Biosynthesis of murein sacculus and peptidoglycan
WAN014GUV_at	SA1140		SAR1103				isdA	AB042826-cds1	Staphylococcus aureus (strain:Cowan I) DNA.	29-kDa cell surface protein	Cell envelope	Other
WAN014GGT_at					SAVP006				Staphylococcus aureus subsp. aureus GENE=""SAVP006""	lipoprotein, putative	Cell envelope	Other
WAN014IPE_at	SA2509						fnbB		Staphylococcus aureus Bacteria; GENE=""fnbB""	fibronectin binding protein B	Cell envelope	Other
WAN014HXU_at			SAR2104		SAV2001				Staphylococcus aureus subsp. aureus Mu50 GENE=""SAV2001""	lipoprotein, putative	Cell envelope	Other
WAN014GAS_at				SAS2309	SAV2418	MW2341			Staphylococcus aureus subsp. aureus Mu50 GENE=""sbi""	IgG-binding protein	Cell envelope	Other
WAN014A7P-seg1_at			SAR1841							cell wall surface anchor family protein	Cell envelope	Other
WAN014A7P-seg2_at			SAR1841							cell wall surface anchor family protein	Cell envelope	Other
WAN014G19_at		SAV039			SAV0399					TN916 ORF13 homolog lmo1103	Cell envelope	Other
WAN014G1B_at		SAV040			SAV0401					TN916 ORF15 homolog lmo1105	Cell envelope	Other
WAN014G1C_at		SAV040			SAV0402					TN916 ORF16 homolog lmo1106	Cell envelope	Other
WAN014G1G_at		SAV040			SAV0405		AF206717			TN916 ORF18 homolog lmo1108	Cell envelope	Other
WAN014G1I_at		SAV040			SAV0408					TN916 ORF20 homolog lmo1111	Cell envelope	Other
WAN014G1J_at		SAV040			SAV0409					TN916 ORF21 homolog lmo1112	Cell envelope	Other
WAN014G1K_at		SAV041			SAV0411					TN916 ORF22 homolog lmo1113	Cell envelope	Other
WAN014G3L_at	SA2505	SA2285					sasG			LPXTG-motif cell wall anchor domain protein, pls homolog	Cell envelope	Other
WAN014G7Y_at		SA1640			SAV1822					lipoprotein, putative	Cell envelope	Other
WAN014GA5_at		SA0195		SAS0176	SAV0201	MW0176				membrane protein, putative	Cell envelope	Other
WAN014GC9_at			SAR2734							LPXTG cell wall surface anchor family protein	Cell envelope	Other
WAN014GFP_at		SA1208		SAS1316	SAV1376	MW1263				membrane protein, putative	Cell envelope	Other
WAN014GQK_at	SA0888									membrane protein B, putative	Cell envelope	Other
WAN014GWP_at	SA0479									surface protein, putative	Cell envelope	Other
WAN014GWT_s_at	SA0050						pls			surface protein Pls	Cell envelope	Other
WAN014GWZ_at				SAS0402		MW0400				lipoprotein, putative	Cell envelope	Other
WAN014HGC_at			SAR1816							putative membrane protein	Cell envelope	Other
WAN014HR0_at			SAR0436							surface protein, putative	Cell envelope	Other
WAN014I2K_at		SA1341			SAV1510					membrane protein, putative	Cell envelope	Other
WAN014IKI_at				SAS0399		MW0397				lipoprotein, putative	Cell envelope	Other
WAN014IKJ_at				SAS0401		MW0399				lipoprotein, putative	Cell envelope	Other
WAN014IML_at			SAR0442	SAS0400		MW0398				lipoprotein, putative	Cell envelope	Other
WAN014INQ_at			SAR0106							putative lipoprotein	Cell envelope	Other
WAN014INX_at			SAR0438							staphylococcus tandem lipoprotein	Cell envelope	Other
WAN014IO2_at			SAR0445							staphylococcus tandem lipoprotein	Cell envelope	Other
WAN014IPY_at			SAR1130							fibrinogen-binding protein	Cell envelope	Other
WAN014IWN_at				SAS0206		MW0206	coa			coagulase	Cell envelope	Other
WAN014IWR_at		SA1319								lipoprotein, putative	Cell envelope	Other
WAN014IWU_at		SA1318								lipoprotein, putative	Cell envelope	Other
WAN014IWV_at		SA1317			SAV1486					lipoprotein, putative	Cell envelope	Other
WAN01BTC0_at			SAR0566							sdrC protein	Cell envelope	Other
WAN01BYXJ_at				SAS2387		MW2420	fnbB			fibronectin binding protein B	Cell envelope	Other
WAN014IRR_at		SAV089			SAV0898				Staphylococcus aureus subsp. aureus Mu50 GENE=""SAV0898""	structural phi Mu50B	Cell envelope	Surface structures
WAN014H4V_at	SA1578		SAR1292							FtsK/SpoIIIE family protein	Cellular processes	Cell division
WAN014GMX_at		SA1755	SAR2036							chemotaxis-inhibiting protein CHIPS	Cellular processes	Chemotaxis and motility
WAN014HV1_at		SA1775	SAR2062		SAV1964					phiN315 scaffolding protein-like protein	Cellular processes	Chemotaxis and motility
WAN014HJJ_at		SA1646	SAR1919		SAV1828			AF064774-cds1	Staphylococcus aureus.	extracellular enterotoxin type I precursor	Cellular processes	Detoxification
WAN014HSN_at			SAR0691							arsenical pump membrane protein 1	Cellular processes	Detoxification
WAN014HT1_at			SAR0720							copper-translocating P-type ATPase	Cellular processes	Detoxification
WAN014IRW_at		SA1816			SAV2008	MW0760				extracellular enterotoxin L	Cellular processes	Detoxification
WAN014INP_at			SAR0101							TfoX N-terminal domain family protein	Cellular processes	DNA transformation
WAN014HH7_at			SAR2124	SAS1942		MW1961		AB043555-cds3	Staphylococcus aureus (isolate:#130) DNA. Gene:agrD	accessory gene regulator protein D-related protein	Cellular processes	Pathogenesis
WAN014HG2_at	SA0896							AF217235-cds15	Gene:;Product:Orf15	pathogenicity island, ORF15	Cellular processes	Pathogenesis
WAN014HEI_at	SA0857			SAS0753		MW0766	vwb	AY032850-cds1	Gene:vwb;Product:secreted von Willebrand factor-binding protein;Note:VWbp	coagulase, putative	Cellular processes	Pathogenesis
WAN014HG5_at	SA0897				SAV0791		virE	U93688-cds11	Gene:;Product:;Note:orf11	virulence-associated protein E	Cellular processes	Pathogenesis
WAN014GAT_at			SAR2508							immunoglobulin G binding protein A precursor, putative	Cellular processes	Pathogenesis
WAN014GB2_at			SAR2709							clumping factor B	Cellular processes	Pathogenesis
WAN014GQJ_at			SAR0842							clumping factor A	Cellular processes	Pathogenesis

Systematic	COL	N315	MRSA	MSSA	Mu50	MW2	GENE NAME	GenBank ID	GenBank Desc.	Protein Function	TIGR Main Role	TIGR Sub Role
WAN014HXE_at			SAR1892							hyaluronate lyase precursor 1	Cellular processes	Pathogenesis
WAN014I7K-seg3_at	SA1472		SAR1447		SAV1434		ebh			pathenogenicity protein, putative	Cellular processes	Pathogenesis
WAN014IO7_at			SAR0845							extracellular matrix and plasma binding protein	Cellular processes	Pathogenesis
WAN014IP3_at	SA1865		SAR1902				splE			serine protease SplE	Cellular processes	Pathogenesis
WAN014IPH_at			SAR2125	SAS1943		MW1962	argC			accessory gene regulator protein C	Cellular processes	Pathogenesis
WAN014IQ0_at		SA1842			SAV2036		agrB			accessory gene regulator B	Cellular processes	Pathogenesis
WAN014IQ1_at			SAR2123	SAS1941		MW1960	agrB			accessory gene regulator protein B	Cellular processes	Pathogenesis
WAN014ITA_at			SAR1525	SAS0926		MW1408				virulence-associated protein E	Cellular processes	Pathogenesis
WAN014ITP_s_at			SAR0114							immunoglobulin G binding protein A precursor	Cellular processes	Pathogenesis
WAN014IUU_at	SA2194						hysA			hyaluronate lyase	Cellular processes	Pathogenesis
WAN014IUV_at		SA2003			SAV2202					hyaluronate lyase precursor	Cellular processes	Pathogenesis
WAN014IWK_at	SA0209						coa			coagulase	Cellular processes	Pathogenesis
WAN014IWQ_at		SAS066								AgrD protein [imported]-related protein	Cellular processes	Pathogenesis
WAN014IXB_at	SA0895									pathogenicity island, ORF16	Cellular processes	Pathogenesis
WAN01BX2C_at			SAR0114	SAS0085		MW0084	spa			immunoglobulin G binding protein A precursor	Cellular processes	Pathogenesis
WAN01CAZ2_at		SA1843			SAV2038		agrC			accessory gene regulator protein C	Cellular processes	Pathogenesis
WAN014GLH_at				SAS1881		MW1898		AB009866-cds12	bacteriophage phi PVL (specific_host:Staphylococcus aureus ATCC 49775) proviral DNA.	Bacterial Ig-like domain (group 2) family	Cellular processes	Toxin production and resistance
WAN014HHY_at		SA1642	SAR1916		SAV1824			AB016487-cds1	Gene:seg2;Product:enterotoxin type Gv	Enterotoxin type G precursor (SEG)	Cellular processes	Toxin production and resistance
WAN014HIS_at			SAR0425					AF094826-cds1	Gene:set2;Product:exotoxin 2	exotoxin, putative	Cellular processes	Toxin production and resistance
WAN014HIT_at			SAR0427					AF094826-cds2	Gene:set3;Product:exotoxin 3	exotoxin 3, putative	Cellular processes	Toxin production and resistance
WAN014INH_at			SAR0429					AF094826-cds4	Gene:set5;Product:exotoxin 5	exotoxin 5, putative	Cellular processes	Toxin production and resistance
WAN014INI_at			SAR0431					AF094826-cds5	Gene:set4;Product:exotoxin 4	exotoxin 5, putative	Cellular processes	Toxin production and resistance
U96610-cds1_s_at					SAV2011			AF217235-cds4	Gene:tst;Product:toxic shock syndrome toxin-1	toxic shock syndrome toxin-1	Cellular processes	Toxin production and resistance
WAN014HK2_at		SA1648					seo	AF285760-cds1	Gene:seo;Product:SEO		Cellular processes	Toxin production and resistance
WAN01CAT8_at		SA1647			SAV1829			AF285760-cds2	Gene:sem;Product:SEM	enterotoxin SEM	Cellular processes	Toxin production and resistance
WAN014HK3_at		SA1645	SAR1918		SAV1827			AF285760-cds4	Gene:Yent1;Product:YENT1	enterotoxin SEU variant	Cellular processes	Toxin production and resistance
WAN014HK4_at		SA1644					yent2	AF285760-cds5	Gene:Yent2;Product:YENT2		Cellular processes	Toxin production and resistance
WAN014HK5_at		SA1643	SAR1917					AF285760-cds6	Gene:sen;Product:SEN	enterotoxin type A, putative	Cellular processes	Toxin production and resistance
WAN014IRH_at		SAV090			SAV0909				Staphylococcus aureus subsp. aureus Mu50 GENE=""SAV0909""	cell wall hydrolase	Cellular processes	Toxin production and resistance
WAN014HGZ_at	SA0907						seb	M11118-cds1	Staphylococcus aureus (strain S6); Gene:;Product:enterotoxin B;Note:precursor	staphylococcal enterotoxin B	Cellular processes	Toxin production and resistance
WAN014HLT_at		tetM,			SAV0398		tetO-s	M21136-cds1	Gene:;Product:;Note:tetM	Tetracycline resistance protein tetM (TetA(M))	Cellular processes	Toxin production and resistance
WAN014HMA_at				SAS0051		MW0051		U11702-cds1	Gene:seh;Product:enterotoxin H;Note:staphylococcal enterotoxin H	enterotoxin H	Cellular processes	Toxin production and resistance
X55185-cds1_x_at				SAS1097	SAV1163	MW1044	P83	X55185-cds1	Gene:;Product:truncated alpha-toxin	Alpha-Hemolysin precursor	Cellular processes	Toxin production and resistance
WAN014FS6_at						MW0052	sen			enterotoxin family protein	Cellular processes	Toxin production and resistance
WAN014FU6_at				SAS0389		MW0387	set8			exotoxin 2	Cellular processes	Toxin production and resistance
WAN014FUA_at				SAS0396		MW0394	set15			exotoxin 3	Cellular processes	Toxin production and resistance
WAN014FZO_at				SAS2262		MW2292				macrolide efflux protein, putative	Cellular processes	Toxin production and resistance
WAN014G2B_at		SA0382			SAV0422		set6			exotoxin 6	Cellular processes	Toxin production and resistance
WAN014GI6_at	SA0052									glycosyl transferase, group 1	Cellular processes	Toxin production and resistance
WAN014GK5_at		SA1761					sep				Cellular processes	Toxin production and resistance
WAN014GPB_at	SA0061	SA0080		SAS0054	SAV0084	MW0054				Domain of unknown function domain protein	Cellular processes	Toxin production and resistance
WAN014GTF_at	SA0032	SA0037	SAR0038		SAV0040	MW0030	maoC			MaoC domain protein	Cellular processes	Toxin production and resistance
WAN014GW1_at	SA0468									exotoxin 3	Cellular processes	Toxin production and resistance
WAN014GW8_at	SA0049									Domain of unknown function domain protein	Cellular processes	Toxin production and resistance
WAN014GWE_at				SAS0387		MW0385				exotoxin 2	Cellular processes	Toxin production and resistance
WAN014GWN_at	SA0478						exotoxin 3			exotoxin 3	Cellular processes	Toxin production and resistance
WAN014HQ3_at			SAR1139							exotoxin 1, putative	Cellular processes	Toxin production and resistance
WAN014HQ5_at			SAR1140							exotoxin 3, putative	Cellular processes	Toxin production and resistance
WAN014HQA_at	SA1043			SAS0971		MW0919				glycosyl transferase, group 1	Cellular processes	Toxin production and resistance
WAN014HQY_at			SAR0424							exotoxin, putative	Cellular processes	Toxin production and resistance
WAN014HQZ_at			SAR0435							exotoxin 3, putative	Cellular processes	Toxin production and resistance
WAN014HSM_at			SAR0690							arsenical resistance operon repressor 1	Cellular processes	Toxin production and resistance
WAN014HVC_at			SAR2108							Aerolysin/Leukocidin family protein	Cellular processes	Toxin production and resistance
WAN014IMK_at	SA0474	SA0390		SAS0393	SAV0429	MW0391				exotoxin 4	Cellular processes	Toxin production and resistance
WAN014ITG_at		SA0032	SAR0032		SAV0034		BRP			bleomycin resistance protein	Cellular processes	Toxin production and resistance
WAN014ITM_at	SA0886			SAS1921		MW1938	ent			staphylococcal enterotoxin	Cellular processes	Toxin production and resistance
WAN014ITN_at	SA0887			SAS1920		MW1937	sei			staphylococcal extracellular enterotoxin type I	Cellular processes	Toxin production and resistance
WAN014IW4_at		SA1817								enterotoxin type c-2 precursor	Cellular processes	Toxin production and resistance
WAN01BTRZ_at			SAR1141							exotoxin 3, putative	Cellular processes	Toxin production and resistance
WAN01BUE4_at			SAR1920							enterotoxin	Cellular processes	Toxin production and resistance
WAN01BUSX_at			SAR2438	SAS2244	SAV2353	MW2274	emrA			Multidrug resistance protein A	Cellular processes	Toxin production and resistance
WAN01CBER_s_at		SA2385	SAR1736		SAV1656					Streptomycin 3-adenylyltransferase (AAD(9))	Cellular processes	Toxin production and resistance
WAN014IXC_at		SAV091			SAV0913				Staphylococcus aureus subsp. aureus Mu50 Bacteria; GENE=""SAV0913""	amidase	Central intermediary metabolism	Other
WAN01C5GL_s_at					SAVP027			M18086-cds2	Staphylococcus aureus (strain SK982) DNA.	acetyltransferase, GNAT family	Central intermediary metabolism	Other
WAN014G6Y_at		SAS076			SAV2213					conserved hypothetical protein	conserved hypothetical protein	conserved hypothetical protein
WAN014G7W_at		SA1635			SAV1817					conserved hypothetical protein	conserved hypothetical protein	conserved hypothetical protein
WAN014HUV_at		SA1769	SAR2055		SAV1958					conserved hypothetical protein	conserved hypothetical protein	conserved hypothetical protein
WAN014I4X_at		SA1772	SAR2059		SAV1961					conserved hypothetical protein	conserved hypothetical protein	conserved hypothetical protein
WAN014I4Z_at		SA1779	SAR2066		SAV1968					conserved hypothetical protein	conserved hypothetical protein	conserved hypothetical protein
WAN014I14_at	SA0132					MW0120				replication initiation protein, degenerate	Disrupted reading frame	
WAN014IXT_at	SA1813	SAS055			SAV1785					transposase, putative, degenerate	Disrupted reading frame	
WAN014IXW_at	SA1442					MW1296				transposase, putative, degenerate	Disrupted reading frame	
WAN014HOT_at			SAR0088							putative restriction enzyme modulator protein	DNA metabolism	Degradation of DNA
WAN014G4F_at					SAV0869				Staphylococcus aureus subsp. aureus Mu50 GENE=""SAV0869""	dnaC	DNA metabolism	DNA replication, recombination, and repair
WAN014IWC_at		SAVP00			SAVP001				Staphylococcus aureus subsp. GENE=""repA""	replication initiator protein A	DNA metabolism	DNA replication, recombination, and repair
WAN014GH1_at					SAVP034		Human		Staphylococcus aureus subsp. aureus GENE=""SAVP034""	replication-associated protein	DNA metabolism	DNA replication, recombination, and repair
WAN014FR7_at	SA0042						ccrA1			cassette chromosome recombinase A1	DNA metabolism	DNA replication, recombination, and repair
WAN014G20_at		SAV078			SAV0783					Phage integrase family domain protein	DNA metabolism	DNA replication, recombination, and repair
WAN014G6V_at		SA2010			SAV2209					RNA-directed DNA polymerase	DNA metabolism	DNA replication, recombination, and repair
WAN014H4K_at	SA1573									integrase/recombinase, core domain family, authentic frameshift	DNA metabolism	DNA replication, recombination, and repair
WAN014HLF_at		SA0058	SAR0060		SAV0062					site-specific recombinase	DNA metabolism	DNA replication, recombination, and repair
WAN014HLG_at		SA0057	SAR0059		SAV0061	MW0038				site-specific recombinase	DNA metabolism	DNA replication, recombination, and repair
WAN014HNG_at		SA0028			SAV0029		RepA			repB protein	DNA metabolism	DNA replication, recombination, and repair
WAN014HNK_at		SA0029	SAR0031		SAV0031		Pre			Plasmid recombination enzyme (Mobilization protein)	DNA metabolism	DNA replication, recombination, and repair
WAN014HZ0_at		SA0027			SAV0028		RepA			repB protein	DNA metabolism	DNA replication, recombination, and repair
WAN014I13_at	SA0041						ccrB			cassette chromosome recombinase B, authentic frameshift	DNA metabolism	DNA replication, recombination, and repair
WAN014I1Z_at	SA2141									site-specific recombinase family protein, degenerate	DNA metabolism	DNA replication, recombination, and repair
WAN014ITE_at			SAR1541	SAS0909		MW1425				DNA polymerase I, putative	DNA metabolism	DNA replication, recombination, and repair
WAN01BQWZ_at	SAA0001						repC			replication initiation protein	DNA metabolism	DNA replication, recombination, and repair
WAN01BUTL_at			SAR2460		SAV2371		GnaT			attachment to host cells and virulence	DNA metabolism	DNA replication, recombination, and repair
WAN01CB8G_at		SA2161		SAS2263	SAV2371	MW2293	GnaT			attachment to host cells and virulence	DNA metabolism	DNA replication, recombination, and repair
WAN014FSI_at				SAS0025			HsdR			type I restriction-modification system restriction subunit	DNA metabolism	Restriction/modification
WAN014FSJ_at				SAS0026			HsdS			putative type I restriction enzyme specificity protein	DNA metabolism	Restriction/modification
WAN014FSK_at				SAS0027						Type I restriction-modification system methyltransferase subunit	DNA metabolism	Restriction/modification
WAN014FU9_at				SAS0395		MW0393	hsdS			type I restriction-modification enzyme, S subunit, EcoA family	DNA metabolism	Restriction/modification
WAN014GWK_at	SA0477	SA0392			SAV0432		hsdS			type I restriction-modification system, S subunit, EcoA family	DNA metabolism	Restriction/modification
WAN014GWM_at			SAR0434							type I restriction-modification enzyme, S subunit, EcoA family	DNA metabolism	Restriction/modification
WAN014HL1_at		SA1625			SAV1807					type I R/M system specificity subunit	DNA metabolism	Restriction/modification
WAN014HU8_at			SAR1898							type I restriction-modification enzyme, S subunit, EcoA family	DNA metabolism	Restriction/modification
WAN01CAIK_at		SA1271	SAR1450	SAS1381	SAV1438	MW1327	ilvA			threonine dehydratase	Energy metabolism	Amino acids, peptides and amines
WAN0144LN-seg1_s_at			SAR1447							very large surface anchored protein	Energy metabolism	Anaerobic
WAN014HPS_at			SAR0261							nitric oxide reductase norZ, putative	Energy metabolism	Anaerobic

Systematic	COL	N315	MRSA	MSSA	Mu50	MW2	GENE NAME	GenBank ID	GenBank Desc.	Protein Function	TIGR Main Role	TIGR Sub Role
WAN014IQN_at			SAR2490							transcriptional regulator NirR	Energy metabolism	Anaerobic
WAN014IQQ_at			SAR1826							potential ATP-binding protein	Energy metabolism	Anaerobic
WAN01BU0Q-seg1_at			SAR1447							very large surface anchored protein	Energy metabolism	Anaerobic
WAN01BU0Q-seg6_s_at			SAR1447							very large surface anchored protein	Energy metabolism	Anaerobic
WAN01BUUF_at			SAR2488	SAS2290	SAV2399	MW2321	subunit			assimilatory nitrite reductase	Energy metabolism	Anaerobic
WAN01CB96_x_at		SA2187	SAR2488	SAS2290	SAV2399	MW2321	subunit			assimilatory nitrite reductase	Energy metabolism	Anaerobic
WAN014IOV-seg1_at		SA1267	SAR1447		SAV1434					ATP synthase, Delta/Epsilon chain, long alpha-helix domain, putative	Energy metabolism	ATP-proton motive force interconversion
WAN014IOW-seg2_at		SA1267	SAR1447		SAV1434	MW1324				ATP synthase, Delta/Epsilon chain, long alpha-helix domain, putative	Energy metabolism	ATP-proton motive force interconversion
WAN014IOX-seg3_at		SA1267			SAV1434	MW1324				ATP synthase, Delta/Epsilon chain, long alpha-helix domain, putative	Energy metabolism	ATP-proton motive force interconversion
WAN014IUB_at			SAR1561	SAS0892		MW1441				Na/K ATPase, putative	Energy metabolism	ATP-proton motive force interconversion
WAN01BU0Q-seg3_at			SAR1447		SAV1434					ATP synthase, Delta/Epsilon chain, long alpha-helix domain, putative	Energy metabolism	ATP-proton motive force interconversion
WAN01BU0Q-seg4_at			SAR1447		SAV1434	MW1324				ATP synthase, Delta/Epsilon chain, long alpha-helix domain, putative	Energy metabolism	ATP-proton motive force interconversion
WAN014HL6_at		SA0077	SAR0079		SAV0081					Protein kinase domain protein	Energy metabolism	Glycolysis/gluconeogenesis
WAN014HRX_at			SAR1587							PfkB family carbohydrate kinase	Energy metabolism	Sugars
WAN014G2Q_at			SAR2559	SAS2363		MW2396	fabG			3-oxoacyl-(acyl carrier protein) reductase, authentic point mutation	Fatty acid and phospholipid metabolism	Biosynthesis
WAN014GSU_at	SA0031	SA0036	SAR0037		SAV0039	MW0029				glycerophosphoryl diester phosphodiesterase, putative	Fatty acid and phospholipid metabolism	Degradation
WAN014HXX_at			SAR2592							fatty acid desaturase family protein, putative	Fatty acid and phospholipid metabolism	Other
WAN014GLI_at				SAS1882		MW1899		AB009866-cds11	bacteriophage phi PVL (specific_host:Staphylococcus aureus ATCC 49775) proviral DNA.	conserved hypothetical protein	Hypothetical proteins	Conserved
WAN014HFR_at		SA1800						AB009866-cds35	bacteriophage phi PVL (specific_host:Staphylococcus aureus ATCC 49775) proviral DNA.	conserved hypothetical protein	Hypothetical proteins	Conserved
AB009866-cds37_x_at			SAR1546			MW1430		AB009866-cds37	bacteriophage phi PVL (specific_host:Staphylococcus aureus ATCC 49775) proviral DNA.	conserved hypothetical protein	Hypothetical proteins	Conserved
AB009866-cds56_at				SAS1898		MW1915		AB009866-cds56	bacteriophage phi PVL (specific_host:Staphylococcus aureus ATCC 49775) proviral DNA.	conserved hypothetical protein	Hypothetical proteins	Conserved
WAN014HHN_at				SAS1897		MW1914		AB009866-cds57	bacteriophage phi PVL (specific_host:Staphylococcus aureus ATCC 49775) proviral DNA.	conserved hypothetical protein	Hypothetical proteins	Conserved
WAN014GZC_at	SA0058	SA0078			SAV0082			AB014440-cds4	Staphylococcus aureus (strain:NCTC8325) DNA.	conserved hypothetical protein	Hypothetical proteins	Conserved
AB037671-cds30_at			SAR0042					AB037671-cds30	Staphylococcus aureus (strain:85/2082) DNA.	conserved hypothetical protein	Hypothetical proteins	Conserved
WAN014HHB_at			SAR0086					AB047239-cds1	Staphylococcus aureus (strain:ATCC25923) DNA.	conserved hypothetical protein	Hypothetical proteins	Conserved
WAN014HHF_at			SAR0080					AB047239-cds4	Staphylococcus aureus (strain:ATCC25923) DNA.	conserved hypothetical protein	Hypothetical proteins	Conserved
AF217235-cds11_at					SAV2019			AF217235-cds11	Gene:;Product:Orf11	conserved hypothetical protein	Hypothetical proteins	Conserved
WAN014HJR_at			SAR0372					AF217235-cds17	Gene:;Product:Orf17	conserved hypothetical protein	Hypothetical proteins	Conserved
WAN014INK_at			SAR0380					AF217235-cds7	Gene:;Product:Orf7	conserved hypothetical protein	Hypothetical proteins	Conserved
WAN014GOG_at	SA0285	SA0285	SAR0292		SAV0297			AJ271970-cds2	Gene:;Product:hypothetical protein;Note:ORFB	conserved hypothetical protein	Hypothetical proteins	Conserved
WAN014G3V_at					SAV0785				Staphylococcus aureus subsp. aureus Mu50 GENE=""SAV0785""	conserved hypothetical protein	Hypothetical proteins	Conserved
WAN014HQR_at			SAR0383		SAV0801				Staphylococcus aureus subsp. aureus Mu50 GENE=""SAV0801""	conserved hypothetical protein	Hypothetical proteins	Conserved
WAN014G43_at					SAV0802				Staphylococcus aureus subsp. aureus Mu50 GENE=""SAV0802""	conserved hypothetical protein	Hypothetical proteins	Conserved
WAN014GDD_at					SAV0857				Staphylococcus aureus subsp. aureus Mu50 GENE=""SAV0853""	conserved hypothetical protein	Hypothetical proteins	Conserved
WAN014IS6_at		SAV085			SAV0859				Staphylococcus aureus subsp. aureus Mu50 GENE=""SAV0859""	conserved hypothetical protein	Hypothetical proteins	Conserved
WAN014IRU_at		SAV088			SAV0889				Staphylococcus aureus subsp. aureus Mu50 GENE=""SAV0889""	conserved hypothetical protein	Hypothetical proteins	Conserved
WAN014IRQ_at		SAV089			SAV0899				Staphylococcus aureus subsp. aureus Mu50 GENE=""SAV0899""	conserved hypothetical protein	Hypothetical proteins	Conserved
WAN014G4V_at					SAV0916				Staphylococcus aureus subsp. aureus Mu50 GENE=""SAV0916""	conserved hypothetical protein	Hypothetical proteins	Conserved
WAN014G84_at					SAV1999				Staphylococcus aureus subsp. aureus Mu50 GENE=""SAV1999""	conserved hypothetical protein	Hypothetical proteins	Conserved
WAN014GGS_at					SAVP002				Staphylococcus aureus subsp. aureus Mu50 GENE=""SAVP002""	conserved hypothetical protein	Hypothetical proteins	Conserved
WAN014GGV_at					SAVP010				Staphylococcus aureus subsp. aureus Mu50 GENE=""SAVP010""	conserved hypothetical protein	Hypothetical proteins	Conserved
WAN014IQT_at		SAVP01			SAVP018				Staphylococcus aureus subsp. aureus Mu50 GENE=""SAVP018""	conserved hypothetical protein	Hypothetical proteins	Conserved
WAN01BP62_at				SAS0378	SAV0419	MW0376			Staphylococcus aureus subsp. aureus MW2 GENE=""MW0376""	conserved hypothetical protein	Hypothetical proteins	Conserved
WAN014IQ6_at		SAVP01			SAVP019				Staphylococcus aureus plasmid pSK41	conserved hypothetical protein	Hypothetical proteins	Conserved
WAN014IQ9_at		SAVP01			SAVP015				Staphylococcus aureus plasmid pSK41	conserved hypothetical protein	Hypothetical proteins	Conserved
WAN014IQ8_at		SAVP01			SAVP016				Staphylococcus aureus plasmid pSK41	conserved hypothetical protein	Hypothetical proteins	Conserved
WAN014IQ5_at		SAVP02			SAVP022				Staphylococcus aureus plasmid pSK41	conserved hypothetical protein	Hypothetical proteins	Conserved
WAN014FRW_at				SAS0037						conserved hypothetical protein	Hypothetical proteins	Conserved
WAN014FRX_at				SAS0038						conserved hypothetical protein	Hypothetical proteins	Conserved
WAN014FS3_at				SAS0044						conserved hypothetical protein	Hypothetical proteins	Conserved
WAN014FS5_at				SAS0047						conserved hypothetical protein	Hypothetical proteins	Conserved
WAN014FSB_at				SAS0074		MW0073				conserved hypothetical protein	Hypothetical proteins	Conserved
WAN014FSC_at				SAS0075		MW0074				conserved hypothetical protein	Hypothetical proteins	Conserved
WAN014FSD_at				SAS0076		MW0075				conserved hypothetical protein	Hypothetical proteins	Conserved
WAN014FSE_at				SAS0077		MW0076				conserved hypothetical protein	Hypothetical proteins	Conserved
WAN014FSM_at	SA0332									bacteriophage L54a, conserved hypothetical protein	Hypothetical proteins	Conserved
WAN014FSQ_at				SAS0036						conserved hypothetical protein	Hypothetical proteins	Conserved
WAN014FT2_at		SA0194		SAS0175	SAV0200	MW0175				conserved hypothetical protein	Hypothetical proteins	Conserved
WAN014FUV_at				SAS1089		MW1038				conserved hypothetical protein	Hypothetical proteins	Conserved
WAN014FWW_at			SAR1520	SAS0931		MW1402				conserved hypothetical protein	Hypothetical proteins	Conserved
WAN014FY2_at	SA0043									conserved hypothetical protein	Hypothetical proteins	Conserved
WAN014G1D_at		SAV040			SAV0404					conserved hypothetical protein	Hypothetical proteins	Conserved
WAN014G1F_at	SA2465	SA2246		SAS2349	SAV2457	MW2381				conserved hypothetical protein	Hypothetical proteins	Conserved
WAN014G1H_at		SAV040			SAV0406					conserved hypothetical protein	Hypothetical proteins	Conserved
WAN014G1N_at		SAV041			SAV0414					conserved hypothetical protein	Hypothetical proteins	Conserved
WAN014G21_at		SAV078			SAV0784					conserved hypothetical protein	Hypothetical proteins	Conserved
WAN014G3X_x_at					SAV0788					conserved hypothetical protein	Hypothetical proteins	Conserved
WAN014G5O_at	SA0440	SA0355		SAS0344	SAV0368	MW0344				conserved hypothetical protein	Hypothetical proteins	Conserved
WAN014G61_at		SAV198			SAV1989					conserved hypothetical protein	Hypothetical proteins	Conserved
WAN014G63_at		SA1828	SAR0375		SAV2021					conserved hypothetical protein	Hypothetical proteins	Conserved
WAN014G66_at		SA1829			SAV2022					conserved hypothetical protein	Hypothetical proteins	Conserved
WAN014G6W_at		SA2011			SAV2210					conserved hypothetical protein	Hypothetical proteins	Conserved
WAN014G6X_at		SA2012			SAV2211					conserved hypothetical protein	Hypothetical proteins	Conserved
WAN014G7X_at		SA1636			SAV1818					conserved hypothetical protein	Hypothetical proteins	Conserved
WAN014G87_at		SA1834			SAV2027					conserved hypothetical protein	Hypothetical proteins	Conserved
WAN014G8I_at		SA2015			SAV2216					conserved hypothetical protein	Hypothetical proteins	Conserved
WAN014G9K_at		SA0190		SAS0171	SAV0196	MW0170				conserved hypothetical protein	Hypothetical proteins	Conserved
WAN014GA3_at		SAV039			SAV0395					conserved hypothetical protein	Hypothetical proteins	Conserved
WAN014GA6_at		SA0196		SAS0177	SAV0202	MW0177				conserved hypothetical protein	Hypothetical proteins	Conserved
WAN014GA9_at		SAV040			SAV0407					conserved hypothetical protein	Hypothetical proteins	Conserved
WAN014GAA_at		SAV041			SAV0410					conserved hypothetical protein	Hypothetical proteins	Conserved
WAN014GB7_at		SAV084			SAV0849					conserved hypothetical protein	Hypothetical proteins	Conserved
WAN014GB8_at		SAV085			SAV0854					conserved hypothetical protein	Hypothetical proteins	Conserved
WAN014GFH_at		SA2000			SAV2199					conserved hypothetical protein	Hypothetical proteins	Conserved
WAN014GGE_at	SA0047									conserved hypothetical protein	Hypothetical proteins	Conserved
WAN014GGY_x_at					SAVP021					conserved hypothetical protein	Hypothetical proteins	Conserved
WAN014GJU_at	SA0053									conserved hypothetical protein	Hypothetical proteins	Conserved
WAN014GK6_at		SA1787	SAR1538	SAS0912		MW1422				conserved hypothetical protein	Hypothetical proteins	Conserved
WAN014GKH_at		SA1809								conserved hypothetical protein	Hypothetical proteins	Conserved
WAN014GOL_at	SA0287									conserved hypothetical protein	Hypothetical proteins	Conserved
WAN014GQ4_at	SA0062	SA0081		SAS0055	SAV0085	MW0055				conserved hypothetical protein	Hypothetical proteins	Conserved
WAN014GQP_at	SA0026	SA0024								conserved hypothetical protein	Hypothetical proteins	Conserved
WAN014GQZ_at	SA0340				SAV0866					bacteriophage L54a, conserved hypothetical protein	Hypothetical proteins	Conserved
WAN014GR5_at	SA0027	SA0025	SAR0026		SAV0026	MW0026				conserved hypothetical protein	Hypothetical proteins	Conserved
WAN014GRG_s_at	SA0350									bacteriophage L54a, conserved hypothetical protein	Hypothetical proteins	Conserved
WAN014GS6_at	SA0071			SAS0063		MW0063				conserved hypothetical protein	Hypothetical proteins	Conserved
WAN014GSV_at	SA0073			SAS0065		MW0065				conserved hypothetical protein	Hypothetical proteins	Conserved
WAN014GV7_at	SA0045									conserved hypothetical protein	Hypothetical proteins	Conserved
WAN014GVN_at	SA0048									conserved hypothetical protein	Hypothetical proteins	Conserved
WAN014GX4_s_at		SA0406		SAS0404	SAV0446	MW0402				conserved hypothetical protein	Hypothetical proteins	Conserved
WAN014GZX_at	SA0063	SA0082		SAS0056	SAV0086	MW0056				conserved hypothetical protein	Hypothetical proteins	Conserved
WAN014H16_at	SA0065	SA0084		SAS0058	SAV0088	MW0058				conserved hypothetical protein	Hypothetical proteins	Conserved

Systematic	COL	N315	MRSA	MSSA	Mu50	MW2	GENE NAME	GenBank ID	GenBank Desc.	Protein Function	TIGR Main Role	TIGR Sub Role
WAN014H2J_at		SA1320			SAV1489					conserved hypothetical protein	Hypothetical proteins	Conserved
WAN014H4O_at	SA1574									conserved hypothetical protein	Hypothetical proteins	Conserved
WAN014H4U_at	SA1577		SAR1291							conserved hypothetical protein	Hypothetical proteins	Conserved
WAN014H4W_at	SA0643			SAS0555		MW0551				conserved hypothetical protein	Hypothetical proteins	Conserved
WAN014H4X_at	SA1579		SAR1293							conserved hypothetical protein	Hypothetical proteins	Conserved
WAN014H4Y_at		SA0553			SAV0596					conserved hypothetical protein	Hypothetical proteins	Conserved
WAN014H50_at	SA0644			SAS0556		MW0552				conserved hypothetical protein	Hypothetical proteins	Conserved
WAN014H52_at	SA0645			SAS0557		MW0553				conserved hypothetical protein	Hypothetical proteins	Conserved
WAN014H53_at	SA1581		SAR1295							conserved hypothetical protein	Hypothetical proteins	Conserved
WAN014H54_at	SA0646			SAS0558		MW0554				conserved hypothetical protein	Hypothetical proteins	Conserved
WAN014H55_at	SA1582		SAR1296							conserved hypothetical protein	Hypothetical proteins	Conserved
WAN014H57_at	SA1583		SAR1297							conserved hypothetical protein	Hypothetical proteins	Conserved
WAN014H5C_at	SA0649	SA0554								conserved hypothetical protein	Hypothetical proteins	Conserved
WAN014H5D_at	SA1585		SAR1299							conserved hypothetical protein	Hypothetical proteins	Conserved
WAN014H5E_at	SA0650			SAS0563		MW0558				conserved hypothetical protein	Hypothetical proteins	Conserved
WAN014H5F_at	SA1586		SAR1300							conserved hypothetical protein	Hypothetical proteins	Conserved
WAN014H5G_at	SA0651			SAS0564		MW0559				conserved hypothetical protein	Hypothetical proteins	Conserved
WAN014H5I_at	SA0652			SAS0565		MW0560				conserved hypothetical protein	Hypothetical proteins	Conserved
WAN014H5K_at	SA0653		SAR0603	SAS0566		MW0561				conserved hypothetical protein	Hypothetical proteins	Conserved
WAN014H5M_at	SA0654			SAS0567		MW0562				conserved hypothetical protein	Hypothetical proteins	Conserved
WAN014HL8_at		SA0076	SAR0078		SAV0080					conserved hypothetical protein	Hypothetical proteins	Conserved
WAN014HOG_at			SAR0365							conserved hypothetical protein	Hypothetical proteins	Conserved
WAN014HOR_at			SAR0084							conserved hypothetical protein	Hypothetical proteins	Conserved
WAN014HOU_at			SAR0090							conserved hypothetical protein	Hypothetical proteins	Conserved
WAN014HP6_at			SAR0715							conserved hypothetical protein	Hypothetical proteins	Conserved
WAN014HPW_at			SAR0285							conserved hypothetical protein	Hypothetical proteins	Conserved
WAN014HPX_at			SAR1134							conserved hypothetical protein	Hypothetical proteins	Conserved
WAN014HPY_at			SAR0286							conserved hypothetical protein	Hypothetical proteins	Conserved
WAN014HPZ_at			SAR0287							conserved hypothetical protein	Hypothetical proteins	Conserved
WAN014HQ4_at			SAR0289							conserved hypothetical protein	Hypothetical proteins	Conserved
WAN014HQ6_at			SAR0290							conserved hypothetical protein	Hypothetical proteins	Conserved
WAN014HQQ_at			SAR0379							conserved hypothetical protein	Hypothetical proteins	Conserved
WAN014HQS_at			SAR0384							conserved hypothetical protein	Hypothetical proteins	Conserved
WAN014HTW_at			SAR1884							conserved hypothetical protein	Hypothetical proteins	Conserved
WAN014HU7_at			SAR1897							conserved hypothetical protein	Hypothetical proteins	Conserved
WAN014HUG_at			SAR0983							conserved hypothetical protein	Hypothetical proteins	Conserved
WAN014HUT_at		SA1767	SAR2052		SAV1956					conserved hypothetical protein	Hypothetical proteins	Conserved
WAN014HV4_at		SA1778	SAR2065		SAV1967					conserved hypothetical protein	Hypothetical proteins	Conserved
WAN014HV9_at			SAR2075							conserved hypothetical protein	Hypothetical proteins	Conserved
WAN014HVF_at			SAR2113							conserved hypothetical protein	Hypothetical proteins	Conserved
WAN014HWD_at			SAR1306							conserved hypothetical protein	Hypothetical proteins	Conserved
WAN014HWE_at			SAR1311							conserved hypothetical protein	Hypothetical proteins	Conserved
WAN014HXG_at			SAR1895							conserved hypothetical protein	Hypothetical proteins	Conserved
WAN014HXH_at			SAR1896							conserved hypothetical protein	Hypothetical proteins	Conserved
WAN014HXI_at			SAR1909							conserved hypothetical protein	Hypothetical proteins	Conserved
WAN014HXK_at			SAR1913							conserved hypothetical protein	Hypothetical proteins	Conserved
WAN014HXL_at		SA1641			SAV1823					conserved hypothetical protein	Hypothetical proteins	Conserved
WAN014I23_at	SA2143	SA1957	SAR2240	SAS2053	SAV2152	MW2078				conserved hypothetical protein	Hypothetical proteins	Conserved
WAN014I4O_at	SA2196	SA2005	SAR2294		SAV2204					conserved hypothetical protein	Hypothetical proteins	Conserved
WAN014I56_at	SA2205									conserved hypothetical protein, degenerate	Hypothetical proteins	Conserved
WAN014ILA_at	SA2379		SAR2469							conserved hypothetical protein	Hypothetical proteins	Conserved
WAN014ILX_at	SA0289									conserved hypothetical protein	Hypothetical proteins	Conserved
WAN014IMD_x_at	SA0358									bacteriophage L54a, conserved hypothetical protein	Hypothetical proteins	Conserved
WAN014IMR_x_at						MW0401				conserved hypothetical protein	Hypothetical proteins	Conserved
WAN014IMS_at	SA0069			SAS0061		MW0061				conserved hypothetical protein	Hypothetical proteins	Conserved
WAN014IO8_at			SAR1314							conserved hypothetical protein	Hypothetical proteins	Conserved
WAN014IOC_at			SAR2470	SAS2273	SAV2383	MW2303				conserved hypothetical protein	Hypothetical proteins	Conserved
WAN014IOD_at			SAR2568							conserved hypothetical protein	Hypothetical proteins	Conserved
WAN014IOQ_x_at		SA0364			SAV0379					conserved hypothetical protein	Hypothetical proteins	Conserved
WAN014IQU_at		SA1827	SAR0376		SAV2020					conserved hypothetical protein	Hypothetical proteins	Conserved
WAN014IQV_at		SA2259								conserved hypothetical protein	Hypothetical proteins	Conserved
WAN014IRZ_at		SA1798								conserved hypothetical protein	Hypothetical proteins	Conserved
WAN014IS4_at	SA0341									bacteriophage L54a, conserved hypothetical protein	Hypothetical proteins	Conserved
WAN014ISE_at			SAR0628							conserved hypothetical protein	Hypothetical proteins	Conserved
WAN014ISG_at	SA0354									bacteriophage L54a, conserved hypothetical protein	Hypothetical proteins	Conserved
WAN014ISH_at	SA0348									bacteriophage L54a, conserved hypothetical protein	Hypothetical proteins	Conserved
WAN014ISP_at	SA0381		SAR1505	SAS0946		MW1388				bacteriophage L54a, conserved hypothetical protein	Hypothetical proteins	Conserved
WAN014ISQ_at	SA0380		SAR1506	SAS0945		MW1389				bacteriophage L54a, conserved hypothetical protein	Hypothetical proteins	Conserved
WAN014ISY_at	SA0374		SAR1512	SAS0939		MW1394				bacteriophage L54a, conserved hypothetical protein	Hypothetical proteins	Conserved
WAN014IT4_at	SA0368		SAR1518	SAS0933		MW1400				bacteriophage L54a, conserved hypothetical protein	Hypothetical proteins	Conserved
WAN014IT9_at	SA0363		SAR1523	SAS0928		MW1405				bacteriophage L54a, conserved hypothetical protein	Hypothetical proteins	Conserved
WAN014ITB_at	SA0362		SAR1526	SAS0924		MW1410				bacteriophage L54a, conserved hypothetical protein	Hypothetical proteins	Conserved
WAN014ITJ_at	SA0333									bacteriophage L54a, conserved hypothetical protein	Hypothetical proteins	Conserved
WAN014IU9_at			SAR1538	SAS0912		MW1422				conserved hypothetical protein	Hypothetical proteins	Conserved
WAN014IUA_at	SA0329		SAR1552							bacteriophage L54a, conserved hypothetical protein	Hypothetical proteins	Conserved
WAN014IUQ_at	SA0330		SAR1551							bacteriophage L54a, conserved hypothetical protein	Hypothetical proteins	Conserved
WAN014IUT_at	SA0338									bacteriophage L54a, conserved hypothetical protein	Hypothetical proteins	Conserved
WAN014IVG_at		SAS034								conserved hypothetical protein	Hypothetical proteins	Conserved
WAN014IVN_at			SAR1324							conserved hypothetical protein	Hypothetical proteins	Conserved
WAN014IW2_at	SA0051									conserved hypothetical protein	Hypothetical proteins	Conserved
WAN014IW3_at	SA0349									bacteriophage L54a, conserved hypothetical protein	Hypothetical proteins	Conserved
WAN014IWD_at		SAV085			SAV0858					conserved hypothetical protein	Hypothetical proteins	Conserved
WAN014IWS_at					SAV1490					conserved hypothetical protein	Hypothetical proteins	Conserved
WAN014IWZ_at				SAS1430		MW1444				conserved hypothetical protein	Hypothetical proteins	Conserved
WAN014IXA_at		SA1830								conserved hypothetical protein	Hypothetical proteins	Conserved
WAN014IXF_at	SA0336	SAV198								bacteriophage L54a, conserved hypothetical protein	Hypothetical proteins	Conserved
WAN014IXI_at	SA0355			SAS0917						bacteriophage L54a, conserved hypothetical protein	Hypothetical proteins	Conserved
WAN014IXO_at						MW1917				conserved hypothetical protein	Hypothetical proteins	Conserved
WAN01A737_at					SAV0883					conserved hypothetical protein	Hypothetical proteins	Conserved
WAN01A7HG_at						MW1377				conserved hypothetical protein	Hypothetical proteins	Conserved
WAN01BOU8_at	SA0038									conserved hypothetical protein	Hypothetical proteins	Conserved
WAN01BOUE_x_at	SA0044									conserved hypothetical protein	Hypothetical proteins	Conserved
WAN01BT25_x_at			SAR0219							conserved hypothetical protein	Hypothetical proteins	Conserved
WAN01BT7Y_at			SAR0426							conserved hypothetical protein	Hypothetical proteins	Conserved
WAN01BT8N_at			SAR0451							conserved hypothetical protein	Hypothetical proteins	Conserved
WAN01BTNH_at			SAR0982							conserved hypothetical protein	Hypothetical proteins	Conserved
WAN01BTO3_at			SAR1006							conserved hypothetical protein	Hypothetical proteins	Conserved
WAN01BUNR_at			SAR2259	SAS2069	SAV2167	MW2094				conserved hypothetical protein	Hypothetical proteins	Conserved
WAN01BURL_at			SAR2392	SAS2200	SAV2308	MW2228				conserved hypothetical protein	Hypothetical proteins	Conserved
WAN01BUZL_at			SAR2653							conserved hypothetical protein	Hypothetical proteins	Conserved
WAN01BV1S_at			SAR2725	SAS2532	SAV2646	MW2567				conserved hypothetical protein	Hypothetical proteins	Conserved
WAN01BV3G_at			SAR2783	SAS2586a	SAV2703	MW2622				conserved hypothetical protein	Hypothetical proteins	Conserved

Supplemental table 3

Systematic	COL	N315	MRSA	MSSA	Mu50	MW2	GENE NAME	GenBank ID	GenBank Desc.	Protein Function	TIGR Main Role	TIGR Sub Role
WAN01BX0Q_at				SAS0029						conserved hypothetical protein	Hypothetical proteins	Conserved
WAN01BY5G_at				SAS1429		MW1443				conserved hypothetical protein	Hypothetical proteins	Conserved
WAN01BYWW_at				SAS2367		MW2400				conserved hypothetical protein	Hypothetical proteins	Conserved
WAN01C1SB_x_at					SAV0883					conserved hypothetical protein	Hypothetical proteins	Conserved
WAN01C401_x_at		SAV088			SAV0883					conserved hypothetical protein	Hypothetical proteins	Conserved
WAN01C9TR_at		SA0409	SAR0449	SAS0407	SAV0449	MW0405				conserved hypothetical protein	Hypothetical proteins	Conserved
WAN01CAX9_x_at		SA1782								conserved hypothetical protein	Hypothetical proteins	Conserved
WAN01CAXO_at		SA1797								conserved hypothetical protein	Hypothetical proteins	Conserved
WAN01CAYE_at		SA1821			SAV0799					conserved hypothetical protein	Hypothetical proteins	Conserved
WAN01CAYF_x_at		SA1822	SAR0380		SAV2015					conserved hypothetical protein	Hypothetical proteins	Conserved
WAN01CAYG_x_at		SA1823			SAV2016					conserved hypothetical protein	Hypothetical proteins	Conserved
WAN01CAYH_s_at		SA1824			SAV2017					conserved hypothetical protein	Hypothetical proteins	Conserved
WAN01CAYJ_at		SA1826	SAR0377		SAV2019					conserved hypothetical protein	Hypothetical proteins	Conserved
WAN01CAYO_x_at		SA1831			SAV2024					conserved hypothetical protein	Hypothetical proteins	Conserved
WAN014GQQ_at	SA0066									conserved domain protein	Hypothetical proteins	Domain
WAN01BQI0_s_at	SA2201									conserved domain protein	Hypothetical proteins	Domain
WAN01BQI1_at	SA2202									conserved domain protein	Hypothetical proteins	Domain
WAN014IPC_at	SA2511						fnbA		S.aureus fibronectin-binding protein (fnbA) mRNA	hypothetical protein	Hypothetical proteins	Not Conserved
WAN014FRE_at	SA1340		SAR1320							hypothetical protein	Hypothetical proteins	Not Conserved
WAN014FRR_at	SA1348	SAS039	SAR1325		SAV1314					hypothetical protein	Hypothetical proteins	Not Conserved
WAN014FW1_at	SA2336									hypothetical protein	Hypothetical proteins	Not Conserved
WAN014G07_at	SA2444									hypothetical protein	Hypothetical proteins	Not Conserved
WAN014G0C_at	SA1850	SA1621								hypothetical protein	Hypothetical proteins	Not Conserved
WAN014G35_at	SA2005									hypothetical protein	Hypothetical proteins	Not Conserved
WAN014G5M_at	SA2543									hypothetical protein	Hypothetical proteins	Not Conserved
WAN014G6D_at	SA2558									hypothetical protein	Hypothetical proteins	Not Conserved
WAN014G6E_at	SA2559									hypothetical protein	Hypothetical proteins	Not Conserved
WAN014G6I_at	SA2200	SA2009			SAV2208					hypothetical protein	Hypothetical proteins	Not Conserved
WAN014G74_x_at	SA2259									hypothetical protein	Hypothetical proteins	Not Conserved
WAN014G8R_at	SA2611									hypothetical protein	Hypothetical proteins	Not Conserved
WAN014GAP_at	SA2647	SA2419	SAR2704	SAS2512	SAV2626	MW2546				hypothetical protein	Hypothetical proteins	Not Conserved
WAN014GAW_x_at	SA2420									hypothetical protein	Hypothetical proteins	Not Conserved
WAN014GDM_at	SA0462	SA0377	SAR0411		SAV0416					hypothetical protein	Hypothetical proteins	Not Conserved
WAN014GDY_at	SA2715	SA2483			SAV2691					hypothetical protein	Hypothetical proteins	Not Conserved
WAN014GE6_at	SA2728		SAR2787							hypothetical protein	Hypothetical proteins	Not Conserved
WAN014GE8_at	SA0463	SA0378			SAV0417					hypothetical protein	Hypothetical proteins	Not Conserved
WAN014GMA_at	SA0258									hypothetical protein	Hypothetical proteins	Not Conserved
WAN014GN4_at	SA0268									hypothetical protein	Hypothetical proteins	Not Conserved
WAN014GOT_at	SA0060									hypothetical protein	Hypothetical proteins	Not Conserved
WAN014GOZ_at	SA0290									hypothetical protein	Hypothetical proteins	Not Conserved
WAN014GS5_at	SA0030		SAR0036							hypothetical protein	Hypothetical proteins	Not Conserved
WAN014GUD_at	SA0406									hypothetical protein	Hypothetical proteins	Not Conserved
WAN014GUM_at	SA0035					MW0033				hypothetical protein	Hypothetical proteins	Not Conserved
WAN014GV0_at	SA1156									hypothetical protein	Hypothetical proteins	Not Conserved
WAN014GWQ_at	SA1391									hypothetical protein	Hypothetical proteins	Not Conserved
WAN014GWW_at	SA0485	SA0404					lpl8			hypothetical protein	Hypothetical proteins	Not Conserved
WAN014GWY_at	SA0483									hypothetical protein	Hypothetical proteins	Not Conserved
WAN014GX5_at	SA0487									hypothetical protein	Hypothetical proteins	Not Conserved
WAN014GX7_at	SA0488	SA0407	SAR0447	SAS0405	SAV0447	MW0403				hypothetical protein	Hypothetical proteins	Not Conserved
WAN014GXC_x_at	SA0493									hypothetical protein	Hypothetical proteins	Not Conserved
WAN014GXX_at	SA0055									hypothetical protein	Hypothetical proteins	Not Conserved
WAN014H17_at	SA0133									hypothetical protein	Hypothetical proteins	Not Conserved
WAN014H1B_at	SA1507	SA1300		SAS1409a	SAV1467	MW1357				hypothetical protein	Hypothetical proteins	Not Conserved
WAN014H2A_at	SA1527									hypothetical protein	Hypothetical proteins	Not Conserved
WAN014H2E_at	SA1529									hypothetical protein	Hypothetical proteins	Not Conserved
WAN014H2K_x_at	SA1532									hypothetical protein	Hypothetical proteins	Not Conserved
WAN014H4L_at	SA0076	SA0089								hypothetical protein	Hypothetical proteins	Not Conserved
WAN014H4Q_at	SA1575		SAR1289							hypothetical protein	Hypothetical proteins	Not Conserved
WAN014H51_at	SA1580		SAR1294							hypothetical protein	Hypothetical proteins	Not Conserved
WAN014H5B_at	SA1584		SAR1298							hypothetical protein	Hypothetical proteins	Not Conserved
WAN014H7O_at	SA0082									hypothetical protein	Hypothetical proteins	Not Conserved
WAN014H7U_at	SA0702									hypothetical protein	Hypothetical proteins	Not Conserved
WAN014HAT_at	SA1684									hypothetical protein	Hypothetical proteins	Not Conserved
WAN014HB8_at	SA0087									hypothetical protein	Hypothetical proteins	Not Conserved
WAN014HH5_at	SA0909				SAV0914					hypothetical protein	Hypothetical proteins	Not Conserved
WAN014HIO_at	SA0923									hypothetical protein	Hypothetical proteins	Not Conserved
WAN014HIV_at	SA1815									hypothetical protein	Hypothetical proteins	Not Conserved
WAN014HJN_at	SA0933									hypothetical protein	Hypothetical proteins	Not Conserved
WAN014HKB_at	SA0039									hypothetical protein	Hypothetical proteins	Not Conserved
WAN014HKK_at	SA1851									hypothetical protein	Hypothetical proteins	Not Conserved
WAN014HKN_at	SA1853			SAS1725		MW1743				hypothetical protein	Hypothetical proteins	Not Conserved
WAN014HLN_at	SA0109			SAS0099		MW0098				hypothetical protein	Hypothetical proteins	Not Conserved
WAN014HOC_at	SA0112									hypothetical protein	Hypothetical proteins	Not Conserved
WAN014HQ7_at	SA1042		SAR1007	SAS0970		MW0918				hypothetical protein	Hypothetical proteins	Not Conserved
WAN014HRW_at	SA1959									hypothetical protein	Hypothetical proteins	Not Conserved
WAN014HT8_at	SA0040			SAS0031						hypothetical protein	Hypothetical proteins	Not Conserved
WAN014HV7_at	SA2013			SAS1931		MW1948				hypothetical protein	Hypothetical proteins	Not Conserved
WAN014HYK_x_at	SA2081									hypothetical protein	Hypothetical proteins	Not Conserved
WAN014I3G_at	SA1273									hypothetical protein	Hypothetical proteins	Not Conserved
WAN014I54_at	SA2204									hypothetical protein	Hypothetical proteins	Not Conserved
WAN014IKC_x_at	SA1341									hypothetical protein	Hypothetical proteins	Not Conserved
WAN014IKD_x_at	SA1342									hypothetical protein	Hypothetical proteins	Not Conserved
WAN014IMM_at	SA0481									hypothetical protein	Hypothetical proteins	Not Conserved
WAN014IMN_at	SA0482									hypothetical protein	Hypothetical proteins	Not Conserved
WAN014IMP_at	SA0486									hypothetical protein	Hypothetical proteins	Not Conserved
WAN014IMQ_x_at	SA0484					MW0401				hypothetical protein	Hypothetical proteins	Not Conserved
WAN014IN1_x_at	SA0894				SAV2024					hypothetical protein	Hypothetical proteins	Not Conserved
WAN014IOB_x_at	SA1174			SAS1097a		MW1045				hypothetical protein	Hypothetical proteins	Not Conserved
WAN014IOH_x_at	SA1333	SA1151		SAS1245	SAV1311	MW1193				hypothetical protein	Hypothetical proteins	Not Conserved
WAN014IOI_at	SA2496									hypothetical protein	Hypothetical proteins	Not Conserved
WAN014IOO_at	SA0450		SAR0397	SAS0356		MW0355				hypothetical protein	Hypothetical proteins	Not Conserved
WAN014IRD_at	SA0911									hypothetical protein	Hypothetical proteins	Not Conserved
WAN014IUH_at	SA1347									hypothetical protein	Hypothetical proteins	Not Conserved
WAN014IUI_at	SA1350	SAS040		SAS1257	SAV1316	MW1204				hypothetical protein	Hypothetical proteins	Not Conserved
WAN014IUS_at	SA0489	SA0408	SAR0448	SAS0406	SAV0448	MW0404				hypothetical protein	Hypothetical proteins	Not Conserved
WAN014IUZ_at	SA0219	SAS007		SAS0215	SAV0239	MW0215				hypothetical protein	Hypothetical proteins	Not Conserved
WAN014IVK_at	SA1344		SAR1312							hypothetical protein	Hypothetical proteins	Not Conserved
WAN014IVL_at	SA1339									hypothetical protein	Hypothetical proteins	Not Conserved
WAN014IWW_at	SA1528									hypothetical protein	Hypothetical proteins	Not Conserved
WAN014IWY_at	SA1533									hypothetical protein	Hypothetical proteins	Not Conserved
WAN014IXL_at	SA0893	SA1832			SAV2025					hypothetical protein	Hypothetical proteins	Not Conserved
WAN01BOU7_at	SA0037					MW0035				hypothetical protein	Hypothetical proteins	Not Conserved

Systematic	COL	N315	MRSA	MSSA	Mu50	MW2	GENE NAME	GenBank ID	GenBank Desc.	Protein Function	TIGR Main Role	TIGR Sub Role
WAN01BOUQ_at	SA0056									hypothetical protein	Hypothetical proteins	Not Conserved
WAN01BP0A_at	SA0256									hypothetical protein	Hypothetical proteins	Not Conserved
WAN01BP6C_at	SA0475									hypothetical protein	Hypothetical proteins	Not Conserved
WAN01BPFR_at	SA0819									hypothetical protein	Hypothetical proteins	Not Conserved
WAN01BPTJ_at	SA1318									hypothetical protein	Hypothetical proteins	Not Conserved
WAN01BPTV_at	SA1330									hypothetical protein	Hypothetical proteins	Not Conserved
WAN01BPU8_at	SA1343									hypothetical protein	Hypothetical proteins	Not Conserved
WAN01BPUL_at	SA1356									hypothetical protein	Hypothetical proteins	Not Conserved
WAN01BPZ6_at	SA1521									hypothetical protein	Hypothetical proteins	Not Conserved
WAN01BQ9B_at	SA1886									hypothetical protein	Hypothetical proteins	Not Conserved
WAN01BQ9Z_at	SA1910			SAS1774						hypothetical protein	Hypothetical proteins	Not Conserved
WAN01BQDB_at	SA2032									hypothetical protein	Hypothetical proteins	Not Conserved
WAN01BQE8_at	SA2065									hypothetical protein	Hypothetical proteins	Not Conserved
WAN01BQHM_at	SA2187									hypothetical protein	Hypothetical proteins	Not Conserved
WAN01BQJG_at	SA2254									hypothetical protein	Hypothetical proteins	Not Conserved
WAN01BQMO_at	SA2370									hypothetical protein	Hypothetical proteins	Not Conserved
WAN01BQQ7_at	SA2497									hypothetical protein	Hypothetical proteins	Not Conserved
WAN01BQQK_at	SA2510									hypothetical protein	Hypothetical proteins	Not Conserved
WAN01BT7O_at			SAR0414							hypothetical protein	Hypothetical proteins	Not Conserved
WAN014IXZ_at		SA0764								Tn554, transposase C	Mobile and extrachromosomal element functi	
WAN01CASJ_s_at		SA1622								transposase, degenerate	Mobile and extrachromosomal element functi	
WAN014GOY_at			SAR1562			MW1442		AB006796-cds3	Staphylococcus aureus (strain:ATCC49775) DNA.	integrase	Mobile and extrachromosomal element functi	Prophage functions
AF217235-cds5_at			SAR0382	SAS1932	SAV0800	MW1949	g1p	AF217235-cds5	Gene:;Product:Orf5;Note:similar to phage terminase small subunit	bacteriophage terminase small subunit	Mobile and extrachromosomal element functi	Prophage functions
WAN01BUIE_at			SAR2071					AP001553-cds35	bacteriophage phi ETA (specific_host:Staphylococcus aureus E-1) DNA.	hypothetical phage protein-related protein	Mobile and extrachromosomal element functi	Prophage functions
WAN014IRY_at		SAV088			SAV0885		g1p		Staphylococcus aureus subsp. aureus Mu50 GENE=""SAV0885""	small terminase	Mobile and extrachromosomal element functi	Prophage functions
WAN014IRX_at		SAV088			SAV0886				Staphylococcus aureus subsp. aureus Mu50 GENE=""SAV0886""	phage terminase, large subunit, PBSX family	Mobile and extrachromosomal element functi	Prophage functions
WAN014IL3_at					SAV0887				Staphylococcus aureus subsp. aureus Mu50 GENE=""SAV0887""	phage portal protein, SPP1 family	Mobile and extrachromosomal element functi	Prophage functions
WAN014IRJ_at		SAV090			SAV0907				Staphylococcus aureus subsp. aureus Mu50 GENE=""SAV0907""	phage uncharacterized protein, XkdX family, putative	Mobile and extrachromosomal element functi	Prophage functions
WAN014INB_x_at	SA0905							U93688-cds3	Gene:;Product:;Note:orf3	pathogenicity island, ORF6	Mobile and extrachromosomal element functi	Prophage functions
WAN014IN7_x_at	SA0904							U93688-cds4	Gene:;Product:;Note:orf4	pathogenicity island, ORF7	Mobile and extrachromosomal element functi	Prophage functions
WAN014IN6_x_at	SA0903							U93688-cds5	Gene:;Product:;Note:orf5	pathogenicity island, ORF5	Mobile and extrachromosomal element functi	Prophage functions
WAN014HGF_x_at	SA0900							U93688-cds7	Gene:;Product:;Note:orf7	pathogenicity island, ORF1	Mobile and extrachromosomal element functi	Prophage functions
WAN014FUJ_at				SAS0891						prophage L54a, integrase	Mobile and extrachromosomal element functi	Prophage functions
WAN014FUM_at				SAS0900						hypothetical phage protein-related protein	Mobile and extrachromosomal element functi	Prophage functions
WAN014FWM_at				SAS0899						hypothetical phage protein-related protein	Mobile and extrachromosomal element functi	Prophage functions
WAN014FWN_at				SAS0901						hypothetical phage protein-related protein	Mobile and extrachromosomal element functi	Prophage functions
WAN014FWS_at				SAS0922						hypothetical phage protein	Mobile and extrachromosomal element functi	Prophage functions
WAN014G17_at		int, i			SAV0392					integrase homolog	Mobile and extrachromosomal element functi	Prophage functions
WAN014G5Y_at		SAV197			SAV1975					phage integrase family protein	Mobile and extrachromosomal element functi	Prophage functions
WAN014GL3_at			SAR2047							hypothetical phage protein-related protein	Mobile and extrachromosomal element functi	Prophage functions
WAN014GOA_at			SAR1545							hypothetical phage protein	Mobile and extrachromosomal element functi	Prophage functions
WAN014HG9_at	SA0898				SAV0792					pathogenicity island, ORF12	Mobile and extrachromosomal element functi	Prophage functions
WAN014HOH_at			SAR0366							prophage L54a, integrase	Mobile and extrachromosomal element functi	Prophage functions
WAN014HS8_at			SAR0629							phage integrase family protein	Mobile and extrachromosomal element functi	Prophage functions
WAN014HUS_at			SAR2051							hypothetical phage protein-related protein	Mobile and extrachromosomal element functi	Prophage functions
WAN014HUY_at		SA1771	SAR2058		SAV1960					phage head-tail adaptor, putative	Mobile and extrachromosomal element functi	Prophage functions
WAN014HV2_at		SA1776	SAR2063		SAV1965					phage portal protein, HK97 family	Mobile and extrachromosomal element functi	Prophage functions
WAN014HV3_at		SA1777	SAR2064		SAV1966					phage terminase, large subunit, putative	Mobile and extrachromosomal element functi	Prophage functions
WAN014HVB_at			SAR2089							hypothetical phage protein	Mobile and extrachromosomal element functi	Prophage functions
WAN014HX4_at	SA2702		SAR1557							similar to protein gp35 from Bacteriophage A118	Mobile and extrachromosomal element functi	Prophage functions
WAN014HXS_at		SA1802	SAR2097							hypothetical phage protein	Mobile and extrachromosomal element functi	Prophage functions
WAN014I4V_at			SAR2053							hypothetical phage protein	Mobile and extrachromosomal element functi	Prophage functions
WAN014IKL_x_at				SAS0921						hypothetical phage protein-related protein	Mobile and extrachromosomal element functi	Prophage functions
WAN014IKM_x_at			SAR1522	SAS0929						prophage L54a, transcriptional regulator, RinA family	Mobile and extrachromosomal element functi	Prophage functions
WAN014IL6_x_at					SAV0896					phage protein, HK97 gp10 family	Mobile and extrachromosomal element functi	Prophage functions
WAN014ILZ_at		SA1805	SAR2100							repressor protein, putative	Mobile and extrachromosomal element functi	Prophage functions
WAN014INC_x_at	SA0906		SAR0382	SAS1932	SAV0800	MW1949				terminase small subunit	Mobile and extrachromosomal element functi	Prophage functions
WAN014INV_at			SAR0382	SAS1932	SAV0800	MW1949	g1p			bacteriophage terminase small subunit	Mobile and extrachromosomal element functi	Prophage functions
WAN014IQS_at	SA0901	SA1825								pathogenicity island, ORF10	Mobile and extrachromosomal element functi	Prophage functions
WAN014IR3_at		int, i			SAV0847		int			integrase	Mobile and extrachromosomal element functi	Prophage functions
WAN014IVD_at			SAR1524	SAS0927		MW1406				phage protein, putative	Mobile and extrachromosomal element functi	Prophage functions
WAN014IX6_at		SA1835			SAV2028					integrase-like protein	Mobile and extrachromosomal element functi	Prophage functions
WAN01A5HF_x_at			SAR1522	SAS0929						prophage L54a, transcriptional regulator, RinA family	Mobile and extrachromosomal element functi	Prophage functions
WAN01A5HH_x_at			SAR1524	SAS0927		MW1406				phage protein, putative	Mobile and extrachromosomal element functi	Prophage functions
WAN01A5HS_x_at				SAS0916						phage conserved hypothetical protein TIGR01671	Mobile and extrachromosomal element functi	Prophage functions
WAN01A72E_at					SAV0912					holin	Mobile and extrachromosomal element functi	Prophage functions
WAN01A72V_at					SAV0896					phage protein, HK97 gp10 family	Mobile and extrachromosomal element functi	Prophage functions
WAN01A72W_x_at					SAV0896					phage protein, HK97 gp10 family	Mobile and extrachromosomal element functi	Prophage functions
WAN01A733_at					SAV0887					phage portal protein, SPP1 family	Mobile and extrachromosomal element functi	Prophage functions
WAN01A7I7_x_at			SAR1522	SAS0929						prophage L54a, transcriptional regulator, RinA family	Mobile and extrachromosomal element functi	Prophage functions
WAN01A7I9_x_at			SAR1524	SAS0927		MW1406				phage protein, putative	Mobile and extrachromosomal element functi	Prophage functions
WAN01BU2W_x_at			SAR1522	SAS0929						prophage L54a, transcriptional regulator, RinA family	Mobile and extrachromosomal element functi	Prophage functions
WAN01BU34_at			SAR1528							hypothetical phage protein-related protein	Mobile and extrachromosomal element functi	Prophage functions
WAN01BU35_at			SAR1529							hypothetical phage protein-related protein	Mobile and extrachromosomal element functi	Prophage functions
WAN01CAYD_at		SA1820	SAR0382		SAV2012		g1p			bacteriophage terminase small subunit	Mobile and extrachromosomal element functi	Prophage functions
WAN01CBET_s_at		SA2387	SAR1738		SAV1658		Tn554	AB037671-cds17	Staphylococcus aureus (strain:85/2082) DNA.	Transposase B from transposon Tn554	Mobile and extrachromosomal element functi	Transposon functions
WAN014IN9_at		SA0763	SAR1738		SAV1658		Tn554	AB037671-cds76	Staphylococcus aureus (strain:85/2082) DNA.	Transposase B from transposon Tn554	Mobile and extrachromosomal element functi	Transposon functions
WAN014INE_at			SAR0082		SAV1804			AB047239-cds3	Staphylococcus aureus (strain:ATCC25923) DNA.	truncated transposase	Mobile and extrachromosomal element functi	Transposon functions
WAN01CBEU_s_at		SA2388	SAR1739		SAV1659		Tn554	L10909-cds1	Staphylococcus aureus (strain R35) DNA.	Transposase A from transposon Tn554	Mobile and extrachromosomal element functi	Transposon functions
WAN014IQ7_at		SAVP01			SAVP017		HIN		Staphylococcus aureus Bacteria; Firmicutes; GENE=""res""	resolvase	Mobile and extrachromosomal element functi	Transposon functions
WAN014GGW_at					SAVP013				Staphylococcus aureus subsp. aureus Mu50 GENE=""truncated-res""	resolvase	Mobile and extrachromosomal element functi	Transposon functions
WAN014G57_at		SA1624			SAV1806					Transposase for insertion sequence element	Mobile and extrachromosomal element functi	Transposon functions
WAN014GAC_at		tnp, t			SAV0415					transposase, Mutator family	Mobile and extrachromosomal element functi	Transposon functions
WAN014GFO_at		SA2014			SAV2215					Transposase for insertion sequence element	Mobile and extrachromosomal element functi	Transposon functions
WAN014HNQ_at		SA0062			SAV0066		orfB			transposase	Mobile and extrachromosomal element functi	Transposon functions
WAN014HNT_at		SA0063			SAV0067					transposase	Mobile and extrachromosomal element functi	Transposon functions
WAN014HSK_at			SAR0726							IS431mec transposase	Mobile and extrachromosomal element functi	Transposon functions
WAN014INO_at			SAR0081							transposase, IS3 family	Mobile and extrachromosomal element functi	Transposon functions
WAN014IQR_at			SAR1827							Transposase for transposon Tn552 (ORF 480)	Mobile and extrachromosomal element functi	Transposon functions
WAN014IRC_at		SA0064	SAR0066		SAV0068		pXO1			transposase	Mobile and extrachromosomal element functi	Transposon functions
WAN014IU6_at			SAR2307				ORFB			transposase	Mobile and extrachromosomal element functi	Transposon functions
WAN014IU7_at			SAR0520							tnpA-IS1253-like	Mobile and extrachromosomal element functi	Transposon functions
WAN01BXQ2_at			SAR1560	SAS0893		MW1440				hypothetical protein	No Data	No Data
WAN01BYP5_at				SAS2107		MW2133				hypothetical protein	No Data	No Data
WAN01BZ3H_at				SAS2579a		MW2614				hypothetical protein	No Data	No Data
WAN014H4S_at	SA1576		SAR1290							traG protein	Other categories	Plasmid functions
WAN014HFO_at	SA0892									Tn557, ORF14	Other categories	Plasmid functions
WAN014I41_at	SA0318						int	M27965-cds2	Bacteriophage L54a DNA.	bacteriophage L54a, integrase	Other categories	Prophage functions
WAN014GQE_at	SA0885						int	U93688-cds17	Staphylococcus aureus.	integrase	Other categories	Prophage functions
WAN014GQL_at	SA0331									bacteriophage L54a, hypothetical protein	Other categories	Prophage functions
WAN014GQM_at	SA0889									gp35, putative	Other categories	Prophage functions
WAN014GQX_at	SA0339									bacteriophage L54a, single-stranded DNA binding protein	Other categories	Prophage functions

Systematic	COL	N315	MRSA	MSSA	Mu50	MW2	GENE NAME	GenBank ID	GenBank Desc.	Protein Function	TIGR Main Role	TIGR Sub Role
WAN014GR9_at	SA0346									bacteriophage L54a, DNA N-6-adenine-methyltransferase	Other categories	Prophage functions
WAN014GRW_at	SA0359				SAV0878					bacteriophage L54a, hypothetical protein	Other categories	Prophage functions
WAN014I4L_at	SA0319									bacteriophage L54a, hypothetical protein	Other categories	Prophage functions
WAN014IMA_x_at	SA0353									bacteriophage L54a, hypothetical protein	Other categories	Prophage functions
WAN014IMF_x_at	SA0364		SAR1522	SAS0929						bacteriophage L54a, hypothetical protein	Other categories	Prophage functions
WAN014IQA_at	SA0328		SAR1549			MW1433				bacteriophage L54a, hypothetical protein	Other categories	Prophage functions
WAN014IS1_at	SA0344									bacteriophage L54a, hypothetical protein	Other categories	Prophage functions
WAN014IS2_at	SA0343									bacteriophage L54a, DnaB-like helicase family protein	Other categories	Prophage functions
WAN014IS3_at	SA0342									bacteriophage L54a, hypothetical protein	Other categories	Prophage functions
WAN014IS5_at	SA0335									bacteriophage L54a, hypothetical protein	Other categories	Prophage functions
WAN014IS7_at	SA0325									bacteriophage L54a, antirepressor, putative	Other categories	Prophage functions
WAN014IS9_at	SA0323									bacteriophage L54a, hypothetical protein	Other categories	Prophage functions
WAN014ISA_at	SA0322									bacteriophage L54a, Cro-related protein	Other categories	Prophage functions
WAN014ISI_at	SA0388		SAR1498	SAS0953		MW1381				bacteriophage L54a, holin	Other categories	Prophage functions
WAN014ISK_at	SA0386		SAR1500	SAS0951		MW1383				bacteriophage L54a, hypothetical protein	Other categories	Prophage functions
WAN014ISM_at	SA0384		SAR1502	SAS0949		MW1385				bacteriophage L54a, hypothetical protein	Other categories	Prophage functions
WAN014ISN_at	SA0383		SAR1503	SAS0948		MW1386				bacteriophage L54a, hypothetical protein	Other categories	Prophage functions
WAN014ISO_at	SA0382		SAR1504	SAS0947		MW1387				bacteriophage L54a, hypothetical protein	Other categories	Prophage functions
WAN014IST_at	SA0378		SAR1508	SAS0943						bacteriophage L54a, hypothetical protein	Other categories	Prophage functions
WAN014ISU_at	SA0377		SAR1509	SAS0942		MW1391				bacteriophage L54a, hypothetical protein	Other categories	Prophage functions
WAN014ISX_at	SA0375		SAR1511	SAS0940		MW1393				bacteriophage L54a, hypothetical protein	Other categories	Prophage functions
WAN014ISZ_at	SA0373		SAR1513	SAS0938		MW1395				bacteriophage L54a, hypothetical protein	Other categories	Prophage functions
WAN014IT0_at	SA0372		SAR1514	SAS0937		MW1396				bacteriophage L54a, hypothetical protein	Other categories	Prophage functions
WAN014IT1_at	SA0371		SAR1515	SAS0936		MW1397				bacteriophage L54a, hypothetical protein	Other categories	Prophage functions
WAN014IT2_at	SA0370		SAR1516	SAS0935		MW1398				bacteriophage L54a, hypothetical protein	Other categories	Prophage functions
WAN014IT3_at	SA0369		SAR1517	SAS0934		MW1399	clpP			bacteriophage L54a, ATP-dependent Clp protease, proteolytic subunit ClpP	Other categories	Prophage functions
WAN014IT5_at	SA0367									bacteriophage L54a, phage D3 terminase, putative	Other categories	Prophage functions
WAN014IT6_at	SA0366		SAR1520	SAS0931		MW1402				bacteriophage L54a, hypothetical protein	Other categories	Prophage functions
WAN014IT8_at	SA0365									bacteriophage L54a, HNH endonuclease family protein	Other categories	Prophage functions
WAN014ITK_at	SA0327		SAR1550			MW1434				bacteriophage L54a, hypothetical protein	Other categories	Prophage functions
WAN014IW5_at	SA0326				SAV0856					bacteriophage L54a, hypothetical protein	Other categories	Prophage functions
WAN014IX9_at	SA0389									bacteriophage L54a, amidase, putative	Other categories	Prophage functions
WAN01BP23_at	SA0321									bacteriophage L54a, repressor protein	Other categories	Prophage functions
WAN01BQCT_at	SA2014		SAR0382	SAS1932	SAV0800	MW1949				terminase small subunit	Other categories	Prophage functions
WAN014GEW_at	SA0464	SA0379			SAV0418					transposase family protein	Other categories	Transposon functions
WAN014H0K_at	SA0036					MW0034				IS1272, transposase	Other categories	Transposon functions
WAN014IY0_at	SA1839					MW0122				transposase, IS200 family	Other categories	Transposon functions
WAN014GT1_at			SAR1022			MW0932		AF309515-cds1	Gene:sspA;Product:SspA, AA-68to268	glutamyl endopeptidase precursor	Protein fate	Degradation of proteins, peptides, and glycopeptides
WAN01BV1J_at			SAR2716					AJ249166-cds1	Gene:aur;Product:aureolysin	zinc metalloproteinase aureolysin	Protein fate	Degradation of proteins, peptides, and glycopeptides
WAN014G4P_at					SAV0892				Staphylococcus aureus subsp. aureus Mu50 GENE=""SAV0892""	head protein	Protein fate	Degradation of proteins, peptides, and glycopeptides
WAN014IRT_at		SAV089			SAV0892				Staphylococcus aureus subsp. aureus Mu50 GENE=""SAV0892""	head protein	Protein fate	Degradation of proteins, peptides, and glycopeptides
WAN014GLC_at		SA1766								M23/M37 peptidase domain protein protein	Protein fate	Degradation of proteins, peptides, and glycopeptides
WAN014IP7_at				SAS1735	SAV1812	MW1754				serine proteinase SplB	Protein fate	Degradation of proteins, peptides, and glycopeptides
WAN01BQ8D_x_at	SA1852						sprT			peptidase, SprT family	Protein fate	Degradation of proteins, peptides, and glycopeptides
WAN01BUDP_at			SAR1905							serine protease SplA	Protein fate	Degradation of proteins, peptides, and glycopeptides
WAN01BT5N_at			SAR0343				tatC			Sec-independent protein translocase TatC	Protein fate	Protein and peptide secretion and trafficking
AP001553-cds30_at					SAV0875	MW1419		AP001553-cds30	bacteriophage phi ETA (specific_host:Staphylococcus aureus E-1) DNA.	phi PVL ORF 52 homologue	Protein fate	Protein folding and stabilization
WAN014GL9_at		SA1764		SAS1876	SAV1953	MW1893				phi PVL ORF 20 and 21 homologue	Protein fate	Protein folding and stabilization
WAN01C4UE_at		SAV197			SAV1976					phi PVL ORF 52 homologue	Protein fate	Protein folding and stabilization
WAN014G5G_at			SAR0990	SAS0889	SAV1020	MW0901	RF		Staphylococcus aureus subsp. aureus GENE=""SAV1020""	Peptide chain release factor 3 (RF-3)	Protein synthesis	Translation factors
WAN014HPO_at			SAR0228							putative glutamine amidotransferase class-I	Protein synthesis	tRNA aminoacylation
WAN014G2A_at					SAV0397				Staphylococcus aureus subsp. aureus Mu50 GENE=""SAV0397""	Helix-turn-helix domain protein	Regulatory functions	DNA interactions
WAN014FUL_at		SAV085		SAS0897	SAV0851					Helix-turn-helix domain protein	Regulatory functions	DNA interactions
WAN014G8P_at	SA2610	SA2379	SAR2673	SAS2479	SAV2593	MW2513				transcriptional regulator, tetR family	Regulatory functions	DNA interactions
WAN014GEA_at	SA2732	SA2495		SAS2588	SAV2705	MW2624				transcriptional regulator, putative	Regulatory functions	DNA interactions
WAN014GOF_at		SA1804	SAR2099							Helix-turn-helix domain protein	Regulatory functions	DNA interactions
WAN014GQN_at	SA0890									transcriptional regulator, Cro/CI family	Regulatory functions	DNA interactions
WAN014H2W_at	SA0072			SAS0064		MW0064				transcriptional regulator, LysR family	Regulatory functions	DNA interactions
WAN014H3G_at	SA0074			SAS0066		MW0066				transcriptional regulator, lysR family	Regulatory functions	DNA interactions
WAN014HFM_at	SA0891									transcriptional regulator, putative	Regulatory functions	DNA interactions
WAN014HOZ_at			SAR0097							transcriptional regulator, TetR family, putative	Regulatory functions	DNA interactions
WAN014HWK_at			SAR1332							response regulator	Regulatory functions	DNA interactions
WAN014HX5_at			SAR2451							transcriptional regulator, TetR family, putative	Regulatory functions	DNA interactions
WAN014HYR_at			SAR2593							transcriptional regulator, GntR family/aminotransferase, class I, putative	Regulatory functions	DNA interactions
WAN014ILC_x_at		SA0337		SAS0325	SAV0349	MW0325				Helix-turn-helix, putative	Regulatory functions	DNA interactions
WAN01BT5R_x_at			SAR0346							transcriptional regulator, Cro/CI family-related protein	Regulatory functions	DNA interactions
WAN01BX9C_x_at				SAS0325	SAV0349	MW0325				Helix-turn-helix, putative	Regulatory functions	DNA interactions
WAN014HGK_at		SA0041			SAV0044			AB037671-cds29	Staphylococcus aureus (strain:85/2082) DNA.	xylose repressor	Regulatory functions	Other
WAN014HJG_at					SAVP031			AF053771-cds1	Staphylococcus aureus.	Transcriptional regulator qacR	Regulatory functions	Other
WAN014GGM_at					SAVP004				Staphylococcus aureus subsp. aureus GENE=""SAVP004""	transcriptional regulator, Cro/CI family	Regulatory functions	Other
WAN014G3W_at					SAV0786	MW0747			Staphylococcus aureus subsp. aureus Mu50 GENE=""SAV0786""	transcriptional regulator, Cro/CI family	Regulatory functions	Other
WAN014G4L_at					SAV0884				Staphylococcus aureus subsp. aureus Mu50 GENE=""SAV0884""	Transcriptional activator rinA	Regulatory functions	Other
WAN014GGU_at					SAVP008				Staphylococcus aureus subsp. aureus Mu50 GENE=""SAVP008""	Gram positive anchor domain protein	Regulatory functions	Other
WAN014IL7_at					SAV1998				Staphylococcus aureus subsp. aureus Mu50 GENE=""SAV1998""	repressor homolog	Regulatory functions	Other
WAN014A7Y-seg1_at			SAR2734	SAS2540	SAV2654	MW2575				serine-threonine rich antigen	Regulatory functions	Other
WAN014A7Y-seg2_at				SAS2540	SAV2654	MW2575				serine-threonine rich antigen	Regulatory functions	Other
WAN014G51_at	SA2524									transcriptional regulator, MarR family	Regulatory functions	Other
WAN014G67_at		SA1833			SAV2026		trbA			transcription activator of competence development and sporula slr	Regulatory functions	Other
WAN014GMD_at		SAV199			SAV1994					anti repressor	Regulatory functions	Other
WAN014GMF_at		SA1801	SAR2096							anti repressor	Regulatory functions	Other
WAN014GTC_at			SAR1029	SAS0991	SAV1056	MW0939				transcriptional regulator	Regulatory functions	Other
WAN014GTY_at	SA0034	SA0039	SAR0040		SAV0042	MW0032	mecR			methicillin-resistance regulatory protein	Regulatory functions	Other
WAN014HLC_at		SA0067	SAR0069		SAV0071		kdpD			sensor protein KdpD	Regulatory functions	Other
WAN014HNU_at		SA0066	SAR0068		SAV0070					transcription regulator protein kdpE	Regulatory functions	Other
WAN014IOA_at			SAR1926							signal transduction protein TRAP	Regulatory functions	Other
WAN01C0U3_at			SAR2468	SAS2271	SAV2381	MW2301				transcription regulatory protein	Regulatory functions	Other
WAN01C9KB_at		SA0079	SAR0099	SAS0059	SAV0083	MW0059				Nitrogen regulation protein NIFR3	Regulatory functions	Other
WAN014IO4_at			SAR0670							sensor histidine kinase	Regulatory functions	Protein interactions
WAN014HNL_at		SA0033	SAR0033		SAV0035					kanamycin nucleotidyltransferase	Transcription	Degradation of RNA
WAN014GK7_at		SA1794								RecT protein family	Transcription	Other
WAN014FZM_at			SAR1859							Sigma-70 region 2 domain protein	Transcription	Transcription factors
WAN014HSO_at			SAR0692							Arsenate reductase (Arsenical pump modifier)	Transport and binding proteins	Anions
WAN014GLO_at			SAR0263							PTS system, IIA component	Transport and binding proteins	Carbohydrates, organic alcohols, and acids
WAN014HIR_at			SAR0724					AB037671-cds19	Staphylococcus aureus (strain:85/2082) DNA.	Cadmium efflux system accessory protein homolog	Transport and binding proteins	Cations and iron carrying compounds
WAN014IO3_at			SAR0632	SAS0591	SAV0624	MW0587	X93358			Na+/H+ antiporter	Transport and binding proteins	Cations and iron carrying compounds
WAN014IRV_at		SA0069	SAR0070		SAV0072		kdpA			K+-transporting ATPase, A subunit	Transport and binding proteins	Cations and iron carrying compounds
WAN014IU5_at		SA0070	SAR0071		SAV0073		kdpB			K+-transporting ATPase, B subunit	Transport and binding proteins	Cations and iron carrying compounds
WAN014IVC_at		SA0071			SAV0074		kdpC			K+-transporting ATPase, C subunit	Transport and binding proteins	Cations and iron carrying compounds
WAN01BTDR_at			SAR0631	SAS0590	SAV0623	MW0586	ubiquinone			Na+/H+ antiporter	Transport and binding proteins	Cations and iron carrying compounds
WAN01BTDV_at			SAR0635	SAS0594	SAV0627	MW0590				Na+/H+ antiporter	Transport and binding proteins	Cations and iron carrying compounds
WAN01BTG6_at			SAR0723							cadmium-translocating P-type ATPase	Transport and binding proteins	Cations and iron carrying compounds
WAN014IPD_at		SA2291			SAV2503				Staphylococcus aureus subsp. aureus N315 genomic DNA	Fibronectin-binding protein precursor (FNBP)	Transport and binding proteins	Other

Systematic	COL	N315	MRSA	MSSA	Mu50	MW2	GENE NAME	GenBank ID	GenBank Desc.	Protein Function	TIGR Main Role	TIGR Sub Role
WAN014IPF_at		SA2290			SAV2502				Staphylococcus aureus subsp. aureus N315 genomic DNA	fibronectin-binding protein B	Transport and binding proteins	Other
WAN014G4U_at	SA2523									drug transporter, putative	Transport and binding proteins	Other
WAN01BQX0_at	SAA0002						tetK			tetracycline resistance protein	Transport and binding proteins	Other
WAN014FT0_at		SA0192		SAS0173	SAV0198	MW0172				ABC transporter ATP-binding protein	Transport and binding proteins	Unknown substrate
WAN014FT3_at		SA0197		SAS0178	SAV0203	MW0178	III			ABC transporter ATP-binding protein	Transport and binding proteins	Unknown substrate
WAN014GRM_at	SA0070			SAS0062		MW0062				permease, putative	Transport and binding proteins	Unknown substrate
WAN014GV2_at	SA0422	SA0339	SAR0348	SAS0327	SAV0351	MW0327				ABC transporter, ATP-binding protein	Transport and binding proteins	Unknown substrate
WAN014HO0_at			SAR0094							multidrug resistance protein (efflux transporter)	Transport and binding proteins	Unknown substrate
WAN014HSF_at			SAR0672							ABC transporter, permease protein	Transport and binding proteins	Unknown substrate
WAN014HXP_at			SAR2544							ABC transporter, ATP-binding protein	Transport and binding proteins	Unknown substrate
WAN014HYH_at			SAR2452							ABC transporter, ATP-binding protein	Transport and binding proteins	Unknown substrate
WAN014HYI_at			SAR2453							ABC transporter efflux protein, DrrB family, putative	Transport and binding proteins	Unknown substrate
WAN014IWH_at		SA2013			SAV2212					transposase, degenerate	transposase, degenerate	transposase, degenerate
WAN01CASK_x_at		SA1623			SAV1805					transposase, degenerate	transposase, degenerate	transposase, degenerate
WAN014HH1_at						MW1379		AB006796-cds1	Staphylococcus aureus (strain:ATCC49775) DNA.	LukS-PV	Unclassified	Role category not yet assigned
WAN014HH2_at						MW1378		AB006796-cds2	Staphylococcus aureus (strain:ATCC49775) DNA.	LukF-PV	Unclassified	Role category not yet assigned
WAN014GL7_at			SAR2048					AB009866-cds19	bacteriophage phi PVL (specific_host:Staphylococcus aureus ATCC 49775) proviral DNA.	77ORF002	Unclassified	Role category not yet assigned
WAN014A7N-seg1_at		SA1964			SAV2160	MW2087		AB015223-cds1	Staphylococcus aureus (strain:KSA8) DNA.	FmtB protein	Unclassified	Role category not yet assigned
WAN014HGI_at		SA0046			SAV0049			AB037671-cds24	Staphylococcus aureus (strain:85/2082) DNA.	unnamed protein product; ORF4	Unclassified	Role category not yet assigned
WAN014HGJ_at		SA0045	SAR0047		SAV0048			AB037671-cds25	Staphylococcus aureus (strain:85/2082) DNA.	unnamed protein product; ORF5	Unclassified	Role category not yet assigned
WAN014HIX_at		SA0044	SAR0046		SAV0047			AB037671-cds26	Staphylococcus aureus (strain:85/2082) DNA.	unnamed protein product; ORF6	Unclassified	Role category not yet assigned
WAN014HIY_at		SA0043			SAV0046			AB037671-cds27	Staphylococcus aureus (strain:85/2082) DNA.	unnamed protein product; ORF7	Unclassified	Role category not yet assigned
WAN01CA3W_s_at		SA0766	SAR1735		SAV1655		ORF2	AB037671-cds73	Staphylococcus aureus (strain:85/2082) DNA.	rRNA adenine N-6-methyltransferase (Macrolide-lincosamide-streptogramin B	Unclassified	Role category not yet assigned
WAN014HIL_at					SAVP032			AF053771-cds2	Gene:qacB;Product:multidrug efflux protein QacB	Antiseptic resistance protein	Unclassified	Role category not yet assigned
WAN014HIN_at					SAVP033			AF053771-cds3	Gene:;Product:delta-orf186;Note:truncated by IS257 insertion	delta-orf186	Unclassified	Role category not yet assigned
WAN014HJM_at			SAR0385					AF217235-cds3	Gene:;Product:Orf3	putative membrane protein	Unclassified	Role category not yet assigned
WAN01BUID_at			SAR2070					AP001553-cds36	bacteriophage phi ETA (specific_host:Staphylococcus aureus E-1) DNA.	77ORF031	Unclassified	Role category not yet assigned
WAN014IKX_at				SAS1928	SAV0803	MW1945	III		Staphylococcus aureus subsp. aureus Mu50 GENE=""SAV0803""	ferric hydroxamate receptor 1	Unclassified	Role category not yet assigned
WAN014IS8_at		SAV085			SAV0855				Staphylococcus aureus subsp. aureus Mu50 GENE=""SAV0855""	anti-repressor	Unclassified	Role category not yet assigned
WAN014G4D_at					SAV0865				Staphylococcus aureus subsp. aureus Mu50 GENE=""SAV0865""	unnamed protein product; orf36.1	Unclassified	Role category not yet assigned
WAN014G4E_at					SAV0868				Staphylococcus aureus subsp. aureus Mu50 GENE=""SAV0868""	77ORF016	Unclassified	Role category not yet assigned
WAN014G4H_at					SAV0872				Staphylococcus aureus subsp. aureus Mu50 GENE=""SAV0872""	77ORF028	Unclassified	Role category not yet assigned
WAN014G4K_at					SAV0880				Staphylococcus aureus subsp. aureus Mu50 GENE=""SAV0880""	GH08353p	Unclassified	Role category not yet assigned
WAN014IL1_at					SAV0881				Staphylococcus aureus subsp. aureus Mu50 GENE=""SAV0881""	77ORF031	Unclassified	Role category not yet assigned
WAN014G4O_at					SAV0890		KSHV		Staphylococcus aureus subsp. aureus Mu50 GENE=""SAV0890""	phi Mu50B-like protein	Unclassified	Role category not yet assigned
WAN014IW1_at		SAV089			SAV0893				Staphylococcus aureus subsp. aureus Mu50 GENE=""SAV0893""	phi Mu50B-like protein	Unclassified	Role category not yet assigned
WAN014IRS_at		SAV089			SAV0897				Staphylococcus aureus subsp. aureus Mu50 GENE=""SAV0897""	phi Mu50B-like protein	Unclassified	Role category not yet assigned
WAN014IRP_at		SAV090			SAV0900				Staphylococcus aureus subsp. aureus Mu50 GENE=""SAV0900""	unknown phi protein	Unclassified	Role category not yet assigned
WAN014IRO_at		SAV090			SAV0902				Staphylococcus aureus subsp. aureus Mu50 GENE=""SAV0902""	phi ETA orf 54-like protein	Unclassified	Role category not yet assigned
WAN014IRN_at		SAV090			SAV0903				Staphylococcus aureus subsp. aureus Mu50 GENE=""SAV0903""	phi ETA orf 55-like protein	Unclassified	Role category not yet assigned
WAN014G4S_at					SAV0905				Staphylococcus aureus subsp. aureus Mu50 GENE=""SAV0905""	phiETA ORF57-like protein	Unclassified	Role category not yet assigned
WAN014IRK_at		SAV090			SAV0906				Staphylococcus aureus subsp. aureus Mu50 GENE=""SAV0906""	phi105 orf 44-like protein	Unclassified	Role category not yet assigned
WAN014GGL_at					SAVP003				Staphylococcus aureus subsp. aureus Mu50 GENE=""traA""	MobA/MobL family family	Unclassified	Role category not yet assigned
WAN01C5GK_at					SAVP026			M18086-cds3	Staphylococcus aureus (strain SK982) DNA.	Bifunctional AAC/APH [Includes: 6-aminoglycoside N-acetyltransferase(EC 2.3	Unclassified	Role category not yet assigned
WAN014G4C_at					SAV0864		SSB		Staphylococcus aureus subsp. GENE=""SAV0864""	Single-strand binding protein 1 (SSB 1) (Helix-destabilizing protein1)	Unclassified	Role category not yet assigned
WAN014GGZ_at					SAVP030		HIN		Staphylococcus aureus subsp. aureus Mu50 GENE=""truncated-res""	Res4	Unclassified	Role category not yet assigned
U93688-cds1_x_at					SAV2010			U93688-cds1	Gene:;Product:;Note:orf1	Ear	Unclassified	Role category not yet assigned
WAN01BUBL_at			SAR1828					X52734-cds3	Gene:;Product:;Note:binL protein (AA 1-197)	DNA-invertase	Unclassified	Role category not yet assigned
WAN014A7Q-seg1_at		SA1577			SAV1758					fmtB-like protein	Unclassified	Role category not yet assigned
WAN014FS0_at				SAS0043						Far1	Unclassified	Role category not yet assigned
WAN014FSP_at				SAS0034						putative membrane protein	Unclassified	Role category not yet assigned
WAN014FSZ_at		SA0191		SAS0172	SAV0197	MW0171				SA0191/BacG-like protein	Unclassified	Role category not yet assigned
WAN014FT1_at		SA0193		SAS0174	SAV0199	MW0174				Enterococcus faecalis plasmid pPD1 bacI	Unclassified	Role category not yet assigned
WAN014FUB_at		SA0394			SAV0434					Gram-positive signal peptide, YSIRK family domain protein	Unclassified	Role category not yet assigned
WAN014FUC_at				SAS0397		MW0395				Gram-positive signal peptide, YSIRK family domain protein	Unclassified	Role category not yet assigned
WAN014FY1_at		SA0059	SAR0061		SAV0063					unnamed protein product; ORF13	Unclassified	Role category not yet assigned
WAN014G1A_at		SAV040			SAV0400		pXO2			TN916 ORF14 and to L. monocytogenes P60 protein homolog lmo11	Unclassified	Role category not yet assigned
WAN014G1L_at		SAV041			SAV0412					uncharacterized conserved protein CAC1439	Unclassified	Role category not yet assigned
WAN014G1M_at		SAV041			SAV0413					UvrD/REP helicase domain protein	Unclassified	Role category not yet assigned
WAN014G2H_at		SA0399			SAV0439					Staphylococcus tandem lipoproteins subfamily	Unclassified	Role category not yet assigned
WAN014G7V_at		SA1633			SAV1815					Ear	Unclassified	Role category not yet assigned
WAN014GB1_at				SAS2516		MW2551	clfB			clumping factor B	Unclassified	Role category not yet assigned
WAN014GBM_at			SAR2531		SAV2441					fmtA-like protein	Unclassified	Role category not yet assigned
WAN014GI9_x_at			SAR2471							transposase	Unclassified	Role category not yet assigned
WAN014GLB_at		SA1765	SAR2049		SAV1954					77ORF004	Unclassified	Role category not yet assigned
WAN014GO0_at		SA0281		SAS0268	SAV0293	MW0268				YeeE	Unclassified	Role category not yet assigned
WAN014GO9_x_at				SAS0912						77ORF050	Unclassified	Role category not yet assigned
WAN014GQG_at				SAS0752		MW0764				clumping factor	Unclassified	Role category not yet assigned
WAN014H4Z_at			SAR0602							putative membrane protein	Unclassified	Role category not yet assigned
WAN014H7Q_at		SA0095			SAV0099					Staphylococcus tandem lipoproteins subfamily	Unclassified	Role category not yet assigned
WAN014HEK_at		SA0743			SAV0812					secreted von Willebrand factor-binding protein	Unclassified	Role category not yet assigned
WAN014HLA_at		SA0074	SAR0076		SAV0078					unnamed protein product; ORF N009	Unclassified	Role category not yet assigned
WAN014HLB_at		SA0073	SAR0075		SAV0076					unnamed protein product; ORF N010	Unclassified	Role category not yet assigned
WAN014HLD_at		SA0065	SAR1144	SAS1104	SAV0069	MW1052				conserved hypotehtical protein SA0065	Unclassified	Role category not yet assigned
WAN014HLH_at		SA0056	SAR0058		SAV0060					unnamed protein product; ORF17	Unclassified	Role category not yet assigned
WAN014HNM_at		SA0042			SAV0045		putative			unnamed protein product; ORF7	Unclassified	Role category not yet assigned
WAN014HNP_at		SA0061	SAR0063		SAV0065					unnamed protein product; ORF11	Unclassified	Role category not yet assigned
WAN014HNV_at		SA0072	SAR0074		SAV0075					unnamed protein product; ORF13	Unclassified	Role category not yet assigned
WAN014HNW_at			SAR0092							Ren71, putative	Unclassified	Role category not yet assigned
WAN014HNX_at			SAR0093							putative membrane protein	Unclassified	Role category not yet assigned
WAN014HOI_at			SAR0367							DNA-binding protein	Unclassified	Role category not yet assigned
WAN014HP3_at			SAR0638							putative membrane protein	Unclassified	Role category not yet assigned
WAN014HQ0_at			SAR0288							putative membrane protein	Unclassified	Role category not yet assigned
WAN014HQ9_at			SAR1288							putative lipoprotein	Unclassified	Role category not yet assigned
WAN014HRJ_at			SAR1535							77ORF030	Unclassified	Role category not yet assigned
WAN014HRM_at			SAR1554							77ORF023	Unclassified	Role category not yet assigned
WAN014HRP_at			SAR1555							77ORF077	Unclassified	Role category not yet assigned
WAN014HTY_at			SAR1886							putative exported protein	Unclassified	Role category not yet assigned
WAN014HTZ_at			SAR1889							putative exported protein	Unclassified	Role category not yet assigned
WAN014HU0_at			SAR1890							putative exported protein	Unclassified	Role category not yet assigned
WAN014HU6_at			SAR1894							putative exported protein	Unclassified	Role category not yet assigned
WAN014HUU_at		SA1768	SAR2054		SAV1957					77ORF020	Unclassified	Role category not yet assigned
WAN014HUZ_at		SA1773	SAR2060		SAV1962					77ORF045	Unclassified	Role category not yet assigned
WAN014HV0_at		SA1774	SAR2061		SAV1963					77ORF006	Unclassified	Role category not yet assigned
WAN014HV5_at		SA1780	SAR2067		SAV1969					77ORF026	Unclassified	Role category not yet assigned
WAN014HVG_at			SAR1131							putative exported protein	Unclassified	Role category not yet assigned
WAN014HW1_at			SAR2260							hypothethical protein, similar to BAB43261.1 of S. aureus N315	Unclassified	Role category not yet assigned
WAN014HX3_at	SA2704		SAR1556							77ORF011	Unclassified	Role category not yet assigned
WAN014HXJ_at			SAR1911							putative exported protein	Unclassified	Role category not yet assigned
WAN014HYS_at			SAR2788							putative exported protein	Unclassified	Role category not yet assigned
WAN014HYV_at			SAR2789							SprC	Unclassified	Role category not yet assigned
WAN014I4W_at		SA1770			SAV1959					77ORF029	Unclassified	Role category not yet assigned

Systematic	COL	N315	MRSA	MSSA	Mu50	MW2	GENE NAME	GenBank ID	GenBank Desc.	Protein Function	TIGR Main Role	TIGR Sub Role
WAN014IKK_x_at				SAS0905						phi PVL orf 39-like protein	Unclassified	Role category not yet assigned
WAN014IKT_at		SA0398			SAV0438		lpl3			Staphylococcus tandem lipoproteins subfamily	Unclassified	Role category not yet assigned
WAN014IKU_at		SA0400			SAV0440					Staphylococcus tandem lipoproteins subfamily	Unclassified	Role category not yet assigned
WAN014IKV_at		SA0401			SAV0441					Staphylococcus tandem lipoproteins subfamily	Unclassified	Role category not yet assigned
WAN014IKW_at		SA0402			SAV0442					Staphylococcus tandem lipoproteins subfamily	Unclassified	Role category not yet assigned
WAN014IL8_at		SA1818			SAV2010					Ear	Unclassified	Role category not yet assigned
WAN014IMB_at				SAS0915						77ORF030	Unclassified	Role category not yet assigned
WAN014IMO_x_at		SA0403								Staphylococcus tandem lipoproteins subfamily	Unclassified	Role category not yet assigned
WAN014INW_x_at		SA0397			SAV0437		lpl2			Staphylococcus tandem lipoproteins subfamily	Unclassified	Role category not yet assigned
WAN014INY_at		SA0396								Staphylococcus tandem lipoproteins subfamily	Unclassified	Role category not yet assigned
WAN014IO1_at		SA0405			SAV0445					Staphylococcus tandem lipoproteins subfamily	Unclassified	Role category not yet assigned
WAN014IO5_at			SAR0671							ABC transporter ATP-binding protein	Unclassified	Role category not yet assigned
WAN014IOL_at		SA2389			SAV2596					glutamyl-endopeptidase	Unclassified	Role category not yet assigned
WAN014IOM_at		SA2382			SAV2596					glutamyl-endopeptidase	Unclassified	Role category not yet assigned
WAN014ITC_at			SAR1539	SAS0911		MW1423				PVL orf 50-like protein	Unclassified	Role category not yet assigned
WAN014ITD_at			SAR1540	SAS0910		MW1424				ETA orf 26-like protein-related protein	Unclassified	Role category not yet assigned
WAN014ITH_at			SAR1543	SAS0907		MW1427				phi APSE P51-like protein	Unclassified	Role category not yet assigned
WAN014ITO_s_at		SA0107	SAR0114	SAS0085	SAV0111	MW0084				protein A	Unclassified	Role category not yet assigned
WAN014IU1_at			SAR1305							transposase	Unclassified	Role category not yet assigned
WAN014IU2_at		SA0047	SAR1734		SAV0050					unnamed protein product; ORF N050	Unclassified	Role category not yet assigned
WAN014IWJ_s_at		SA0222								staphylocoagulase precursor [imported]	Unclassified	Role category not yet assigned
WAN014IX2_at		SA0745		SAS0755	SAV0814	MW0768				secreted von Willebrand factor-binding protein	Unclassified	Role category not yet assigned
WAN014IX4_at			SAR0846							putative exported protein	Unclassified	Role category not yet assigned
WAN014IXK_at			SAR1533							phi PV83 orf 27-like protein	Unclassified	Role category not yet assigned
WAN014IYC_x_at			SAR2471							transposase	Unclassified	Role category not yet assigned
WAN01A54A_s_at					SAV0894					phi Mu50B-like protein	Unclassified	Role category not yet assigned
WAN01A54B_s_at					SAV0893					phi Mu50B-like protein	Unclassified	Role category not yet assigned
WAN01A54F_s_at					SAV0888					phi Mu50B-like protein	Unclassified	Role category not yet assigned
WAN01A72X_s_at					SAV0894					phi Mu50B-like protein	Unclassified	Role category not yet assigned
WAN01A732_s_at					SAV0888					phi Mu50B-like protein	Unclassified	Role category not yet assigned
WAN01BTJF_at			SAR0838							putative membrane protein	Unclassified	Role category not yet assigned
WAN01BU2B_s_at			SAR1501	SAS0950		MW1384				SLT orf 129-like protein	Unclassified	Role category not yet assigned
WAN01BU38_x_at			SAR1532							phi PVL ORF 52 homologue	Unclassified	Role category not yet assigned
WAN01BUCJ_at			SAR1861							putative membrane protein	Unclassified	Role category not yet assigned
WAN01BUDD_at			SAR1893							putative exported protein	Unclassified	Role category not yet assigned
WAN01BUDU_at			SAR1910							putative membrane protein	Unclassified	Role category not yet assigned
WAN01BUJK_at			SAR2119		SAV2032	MW1956				serine-aspartate repeat protein	Unclassified	Role category not yet assigned
WAN01BUUJ_at			SAR2493	SAS2294	SAV2403	MW2325	formate			NirC protein	Unclassified	Role category not yet assigned
WAN01BUWT_at			SAR2562							putative membrane protein	Unclassified	Role category not yet assigned
WAN01BUWU_at			SAR2563							putative membrane protein	Unclassified	Role category not yet assigned
WAN01BV1L_at			SAR2718							putative exported protein	Unclassified	Role category not yet assigned
WAN01BXFG_at				SAS0520		MW0517	sdrD			sdrD protein	Unclassified	Role category not yet assigned
WAN01BZ4R_at			SAR1828							DNA-invertase	Unclassified	Role category not yet assigned
WAN01BZVA_at			SAR0997	SAS0960	SAV1028	MW0908	lplA			lipoate-protein ligase-like protein	Unclassified	Role category not yet assigned
WAN01C1RM_at			SAR2091							putative exported protein	Unclassified	Role category not yet assigned
WAN01C9JL_at		SA0054	SAR0056		SAV0058		D86934			unnamed protein product; ORF N043	Unclassified	Role category not yet assigned
WAN01C9JM_at		SA0055	SAR0057		SAV0059					unnamed protein product; ORF19	Unclassified	Role category not yet assigned
WAN01C9JR_at		SA0060	SAR0062		SAV0064					unnamed protein product; ORF12	Unclassified	Role category not yet assigned
WAN014G7L_at	SA2583	SA2355		SAS2454	SAV2568	MW2489				acetyltransferase, GNAT family	Unknown function	Enzymes of unknown specificity
WAN014GBU_at	SA0046									metallo-beta-lactamase family protein	Unknown function	Enzymes of unknown specificity
WAN014GEF_at	SA2612	SA2380	SAR2674	SAS2480	SAV2594	MW2514				hydrolase, CocE/NonD family	Unknown function	Enzymes of unknown specificity
WAN014GOW_at		SA1806	SAR2101							exonuclease, putative	Unknown function	Enzymes of unknown specificity
WAN014GQR_at	SA0908									beta-lactamase, putative	Unknown function	Enzymes of unknown specificity
WAN014H0L_at	SA0064	SA0083		SAS0057	SAV0087	MW0057				metallo-beta-lactamase family protein	Unknown function	Enzymes of unknown specificity
WAN014HTI_at			SAR0824							malate oxidoreductase	Unknown function	Enzymes of unknown specificity
WAN014HUX_at	SA2012			SAS1930		MW1947				Acetyltransferase (GNAT) family	Unknown function	Enzymes of unknown specificity
WAN014ILJ_at	SA2541		SAR2609	SAS2415		MW2449				acetyltransferase, GNAT family	Unknown function	Enzymes of unknown specificity
WAN014IOE_at			SAR2779							N-acetyltransferase family protein	Unknown function	Enzymes of unknown specificity
WAN014FSR_at				SAS0046						glyoxalase family protein, putative	Unknown function	General
WAN014GDZ_at	SA2716	SA2484			SAV2692					30S ribosomal protein S14 homolog-related	Unknown function	General
WAN014GNM_at			SAR0284							diarrheal toxin	Unknown function	General
WAN014H9H_at				SAS0639	SAV0674	MW0636				Protein of unknown function (DUF456) superfamily	Unknown function	General
WAN014HQN_at			SAR0368							DNA binding domain, excisionase family protein, putative	Unknown function	General
WAN014HT2_at			SAR0721							mulitcopper oxidase domain protein	Unknown function	General
WAN014HTX_at			SAR1885							Domain of unknown function (DUF955) family	Unknown function	General
WAN014HXW_at			SAR2591							LysE/YggA family protein	Unknown function	General
WAN014I21_at	SA2142									SAP domain protein	Unknown function	General
WAN014I4N_at	SA2195	SA2004								M23/M37 peptidase domain protein	Unknown function	General
WAN014I52_at	SA2203									ClpA-related protein	Unknown function	General
WAN014IPV_at		SA0761	SAR0865	SAS0774	SAV0834	MW0787				conserved protein, ortholog YwqG B. subtilis	Unknown function	General
WAN014IR0_at			SAR2717							immunodominant antigen B	Unknown function	General
WAN014ISR-seg2_at	SA0379		SAR1507	SAS0944		MW1390				bacteriophage L54a, M23/M37 peptidase domain protein	Unknown function	General
WAN014ISV_at	SA0376		SAR1510	SAS0941		MW1392				bacteriophage L54a, bacterial Ig-like domain group 2 family protein	Unknown function	General
WAN014IX5_at	SA2015					MW1951				integrase/recombinase-related protein, authentic frameshift	Unknown function	General
WAN01BUCK_at			SAR1862							abortive infection protein family	Unknown function	General
WAN01BUCL_at			SAR1863							abortive infection protein family	Unknown function	General
WAN01BX0W_at				SAS0035						unnamed protein product; ORF12	Unknown function	General
WAN01CAXR_at		SA1799	SAR2092	SAS1911	SAV1991	MW1928				Domain of unknown function (DUF771) superfamily	Unknown function	General
WAN014HFS_at								AB013298-cds2	Staphylococcus aureus (strain:MS8968) DNA.			
WAN014IN2_at								AB014436-cds1	Staphylococcus aureus (strain:85/2082) DNA.			
WAN014HFU_at								AB014438-cds1	Staphylococcus aureus (strain:85/3907) DNA.			
WAN014HHU_at								AB014438-cds10	Staphylococcus aureus (strain:85/3907) DNA.			
WAN014HFX_at								AB014438-cds11	Staphylococcus aureus (strain:85/3907) DNA.			
WAN014HHV_at								AB014438-cds12	Staphylococcus aureus (strain:85/3907) DNA.			
WAN014HFV_at								AB014438-cds2	Staphylococcus aureus (strain:85/3907) DNA.			
WAN014HHQ_at								AB014438-cds3	Staphylococcus aureus (strain:85/3907) DNA.			
WAN014HHR_at								AB014438-cds4	Staphylococcus aureus (strain:85/3907) DNA.			
WAN014HFW_at								AB014438-cds5	Staphylococcus aureus (strain:85/3907) DNA.			
WAN014HHS_at								AB014438-cds8	Staphylococcus aureus (strain:85/3907) DNA.			
WAN014HHT_at								AB014438-cds9	Staphylococcus aureus (strain:85/3907) DNA.			
WAN014HFZ_at								AB016613-cds1	Staphylococcus aureus plasmid:pEP2104 DNA.			
WAN014HG0_at								AB016613-cds2	Staphylococcus aureus plasmid:pEP2104 DNA.			
WAN014HG1_at								AB016613-cds3	Staphylococcus aureus plasmid:pEP2104 DNA.			
WAN014HIW_at								AB037671-cds22	Staphylococcus aureus (strain:85/2082) DNA.			
WAN014HGN_at								AB037671-cds50	Staphylococcus aureus (strain:85/2082) DNA.			
WAN014HGQ_at								AB037671-cds79	Staphylococcus aureus (strain:85/2082) DNA.			
WAN014HGS_at								AB037671-cds82	Staphylococcus aureus (strain:85/2082) DNA.			
WAN014HGT_at								AB037671-cds83	Staphylococcus aureus (strain:85/2082) DNA.			
WAN014HGU_at								AB037671-cds84	Staphylococcus aureus (strain:85/2082) DNA.			
WAN014HGV_at								AB037671-cds86	Staphylococcus aureus (strain:85/2082) DNA.			
WAN014HGW_at								AB037671-cds89	Staphylococcus aureus (strain:85/2082) DNA.			
WAN014HGX_at								AB037671-cds90	Staphylococcus aureus (strain:85/2082) DNA.			

Systematic	COL	N315	MRSA	MSSA	Mu50	MW2	GENE NAME	GenBank ID	GenBank Desc.	Protein Function	TIGR Main Role	TIGR Sub Role
AB047089-cds1_at					SAV0057			AB047089-cds1	Staphylococcus aureus (strain:85/3907) DNA.			
WAN014HJB_at								AF051916-cds4	Gene:;Product:unknown;Note:ORF140; truncated by IS257 insertion			
WAN014HJC_at								AF051916-cds5	Gene:dfrA;Product:trimethoprim resistance protein DfrA;Note:DHFR			
WAN014HJF_at								AF051917-cds22	Gene:artA;Product:putative regulator of transfer genes ArtA			
WAN014HI1_at								AF051917-cds23	Gene:traA;Product:TraA			
WAN014HI2_at								AF051917-cds24	Gene:traB;Product:putative membrane protein TraB			
WAN014HI5_at								AF051917-cds25	Gene:traC;Product:putative membrane protein TraC			
WAN014HI6_at								AF051917-cds26	Gene:traD;Product:TraD			
WAN014HI7_at								AF051917-cds27	Gene:traE;Product:putative ATPase TraE			
WAN014HI8_at								AF051917-cds28	Gene:traF;Product:putative membrane protein TraF			
WAN014HI9_at								AF051917-cds29	Gene:traG;Product:putative membrane protein TraG			
WAN014HIA_at								AF051917-cds30	Gene:traH;Product:lipoprotein TraH			
WAN014HIB_at								AF051917-cds31	Gene:traI;Product:putative topoisomerase TraI			
WAN014HIC_at								AF051917-cds32	Gene:traJ;Product:putative membrane protein TraJ			
WAN014HID_at								AF051917-cds33	Gene:traK;Product:putative membrane protein TraK			
WAN014HIE_at								AF051917-cds34	Gene:traL;Product:putative membrane protein TraL			
WAN014HII_at								AF051917-cds35	Gene:traM;Product:			
WAN014ING_x_at								AF053772-cds1	Gene:sin;Product:putative recombinase Sin			
WAN014HJ1_at								AF117258-cds4	Gene:vat;Product:acetyltransferase Vat			
WAN014HJ2_at								AF117258-cds5	Gene:vgb;Product:hydrolase VgB			
WAN014HJ3_at								AF117259-cds3	Gene:;Product:ATP binding protein VgA;Note:putative ATP transporter			
WAN014HG4_x_at								AF217235-cds15	Gene:;Product:Orf15			
X58434-cds1_at								AF235026-cds1	Gene:pdhB;Product:pyruvate dehydrogenase beta subunit PdhB			
WAN014HJU_at								AF255950-cds1	Gene:agrD;Product:AgrD signal peptide precursor;Note:autoinducing-peptide			
WAN014HKG_s_at								AJ132841-cds1	Gene:mapN;Product:MapN protein			
AP001553-cds14_x_at			SAR1546					AP001553-cds14	bacteriophage phi ETA (specific_host:Staphylococcus aureus E-1) DNA.			
WAN014GM8_at								AP001553-cds18	bacteriophage phi ETA (specific_host:Staphylococcus aureus E-1) DNA.			
WAN014GM7_at								AP001553-cds22	bacteriophage phi ETA (specific_host:Staphylococcus aureus E-1) DNA.			
WAN014GM6_at								AP001553-cds23	bacteriophage phi ETA (specific_host:Staphylococcus aureus E-1) DNA.			
WAN014GO8_at								AP001553-cds28	bacteriophage phi ETA (specific_host:Staphylococcus aureus E-1) DNA.			
AP001553-cds44_at								AP001553-cds44	bacteriophage phi ETA (specific_host:Staphylococcus aureus E-1) DNA.			
WAN014HKQ_at		SAV199			SAV1997			AP001553-cds7	bacteriophage phi ETA (specific_host:Staphylococcus aureus E-1) DNA.			
WAN014IWP_at									Staphylococcus aureus Bacteria; Firmicutes; GENE=""coa""			
WAN014HL5_at								D42144-cds1	Staphylococcus aureus (strain:P83) DNA.			
WAN014IWM_at									Staphylococcus aureus Bacteria; GENE=""fbpA""			
WAN014GGN_at					SAVP005				Staphylococcus aureus subsp. aureus Mu50 GENE=""SAVP005""			
WAN014HIJ_at									Staphylococcus aureus Bacteria; Firmicutes; GENE=N/A			
WAN014IQ4_at									Staphylococcus aureus Bacteria; Firmicutes; GENE=N/A			
WAN014HIK_at									Staphylococcus aureus Bacteria; Firmicutes; GENE=N/A			
WAN014FWL_at				SAS0898	SAV0852				Staphylococcus aureus subsp. aureus Mu50 GENE=""SAV0852""			
WAN014GDP_at					SAV0895				Staphylococcus aureus subsp. aureus Mu50 GENE=""SAV0895""			
WAN014G4W_at					SAV0917				Staphylococcus aureus subsp. aureus Mu50 GENE=""SAV0917""			
WAN01C4VF_at									Staphylococcus aureus subsp. aureus Mu50 GENE=""SAV2013""			
WAN01C4VG_at									Staphylococcus aureus subsp. aureus Mu50 GENE=""SAV2014""			
WAN014GGO_at					SAVP007				Staphylococcus aureus subsp. aureus Mu50 GENE=""SAVP007""			
WAN014GGP_at					SAVP009				Staphylococcus aureus subsp. aureus Mu50 GENE=""SAVP009""			
WAN014GGQ_at					SAVP011				Staphylococcus aureus subsp. aureus Mu50 GENE=""SAVP011""			
WAN014IU4_at									Staphylococcus epidermidis Bacteria; Firmicutes; GENE=""tnp""			
J04551-cds1_at								J04551-cds1	Plasmid pI258 DNA.			
WAN014HLR_at					SAV0848			M27965-cds1	Bacteriophage L54a DNA.			
WAN014HLW_at	SA2703							M28521-cds1	Gene:entD;Product:enterotoxin D precursor			
WAN014HLX_at	SA2701							M36694-cds1	S.aureus (strain PS80) DNA.			
WAN014IRB_at									Staphylococcus aureus GENE=""smr""			
WAN014IQY_at									Staphylococcus aureus Bacteria; Firmicutes; GENE=""nes""			
WAN014IWE_at									Staphylococcus aureus Bacteria; Firmicutes; GENE=""pre""			
WAN014IWL_at									Staphylococcus aureus Bacteria; GENE=N/A			
WAN014HM7_at								U75367-cds1	Gene:;Product:transposase			
X03216-cds7_at								X03216-cds7	Gene:;Product:peptide 1;Note:(aa 1-15)			
X06627-cds4_at								X06627-cds4	Gene:;Product:ORF (str)			
X16298-cds2_at								X16298-cds2	Gene:;Product:;Note:bin3 product			
X53096-cds1_at								X53096-cds1	Gene:;Product:Sau96I DNA methyltransferase			
X53096-cds2_at								X53096-cds2	Gene:;Product:Sau96I restriction endonuclease			
X75439-cds1_at								X75439-cds1	Gene:;Product:;Note:ORF B			
X75439-cds3_at								X75439-cds3	Gene:;Product:;Note:ORF C			
Y07536-cds4_x_at								Y07536-cds4	Gene:;Product:transposase			
Y07739-cds1_at								Y07739-cds1	Staphylococcus phage Twort.			
Y07739-cds2_at								Y07739-cds2	Staphylococcus phage Twort.			
Y07740-cds1_at								Y07740-cds1	Staphylococcus phage 187.			
Y13600-cds4_at								Y13600-cds4	Staphylococcus aureus.			
Y13766-cds1_at								Y13766-cds1	Staphylococcus aureus.			
WAN014FRY_at				SAS0040								
WAN014FWT_at				SAS0930								
WAN014G18_at		SAV039			SAV0393							
WAN014G9L_at												
WAN014GA2_at		SAV039			SAV0394							
WAN014GA4_at		SAV040			SAV0403							
WAN014GCJ_at												
WAN014GDG_x_at					SAV0863							
WAN014GE4_at												
WAN014GEC_x_at												
WAN014GF1_at												
WAN014GFN_at		SAS077			SAV2214							
WAN014GG8_at												
WAN014GGX_x_at					SAVP020							
WAN014GH2_at		SA0030	SAR0031a		SAV0032							
WAN014GH3_at		SA0031	SAR0031b		SAV0033							
WAN014GH4_at		SAS003	SAR0075a		SAV0077							
WAN014GH7_at		SAS004		SAS0101a	SAV0127							
WAN014GH8_at	SA2699	SA0188	SAR0195	SAS0169	SAV0194	MW0168						
WAN014GHB_at		SAS006		SAS0179	SAV0204	MW0179						
WAN014GHD_at		SAS011		SAS0352	SAV0375	MW0350						
WAN014GHH_at		SAS013			SAV0430							
WAN014GHO_at		SAS038		SAS1231a	SAV1299	MW1181						
WAN014GIF_at		SA1610		SAS1712a	SAV1792	MW1730						
WAN014GIN_at		SAS075		SAS2099	SAV2198	MW2124						
WAN014GK0_at												
WAN014GKA_x_at												
WAN014GKF_at												
WAN014GKI_at												
WAN014GKO_at												
WAN014GLR_at				SAS1657a		MW1673						
WAN014GM2_at												

Systematic	COL	N315	MRSA	MSSA	Mu50	MW2	GENE NAME	GenBank ID	GenBank Desc.	Protein Function	TIGR Main Role	TIGR Sub Role
WAN014GML_at		SA1803	SAR2098									
WAN014GOD_at			SAR1548									
WAN014GOQ_at												
WAN014GOX_at			SAR2102									
WAN014GRF_at												
WAN014GSL_at												
WAN014GT6_at												
WAN014GX6_at												
WAN014H2L_x_at												
WAN014HL9_at		SA0075	SAR0077		SAV0079							
WAN014HQD_at												
WAN014HQP_at												
WAN014HQV_at												
WAN014HRQ_at												
WAN014HRR_at												
WAN014HRS_at												
WAN014HTN_at												
WAN014HU5_at												
WAN014HV8_at			SAR2073									
WAN014HW6_at												
WAN014HW9_at												
WAN014HZL_at												
WAN014HZM_at												
WAN014I6M_at												
WAN014INZ_at												
WAN014IO0_at												
WAN014IO6_at												
WAN014IO9_at			SAR1322									
WAN014IOF_at		SA0350	SAR0359	SAS0338	SAV0362	MW0338						
WAN014IQ3_at		SA0053			SAV0057							
WAN014IRG_at												
WAN014IRL_at												
WAN014ISF_at												
WAN014ISW_at												
WAN014IT7_at												
WAN014ITF_at												
WAN014ITI_at												
WAN014ITY_at												
WAN014IUW_at												
WAN014IVA_at			SAR1137									
WAN014IVU_at												
WAN014IVY_at		SAV086			SAV0867							
WAN014IW6_at												
WAN014IW7_at												
WAN014IW8_at												
WAN014IW9_at		SAV165			SAV1654							
WAN014IWA_at												
WAN014IWB_at												
WAN014IX0_at												
WAN014IXJ_s_at		SAV197			SAV1977							
WAN014IXP_at												
WAN014IXX_at												
WAN01A584_s_at					SAV0867							
WAN01A72C_x_at					SAV0915							
WAN01A736_at												
WAN01A73B_at												
WAN01A73T_x_at												
WAN01A771_x_at												
WAN01A776_at												
WAN01A7IN_x_at				SAS0914								
WAN01A7IY_x_at												
WAN01A7JA_at												
WAN01A7WT_at												
WAN01A8BV_at					SAV2208							
WAN01A8BX_s_at												
WAN01A8MR_at												
WAN01A8SB_at												
WAN01AAWB_s_at												
WAN01AB5H_at												
WAN01ABL8_at												
WAN01AC23_at												
WAN01ACOT_at												
WAN01BRCD_at			SAR0604									
WAN01BSDG_at												
WAN01BSVG_at												
WAN01BSY9_at												
WAN01BSYF_at			SAR0089									
WAN01BSYQ_at			SAR0100									
WAN01BT0Y_at												
WAN01BT18_at												
WAN01BT3F_at												
WAN01BT4Z_at												
WAN01BT6H_at			SAR0371									
WAN01BT6Y_x_at												
WAN01BT76_x_at			SAR395a									
WAN01BT7P_at												
WAN01BT82_at												
WAN01BT83_at												
WAN01BTCM_at												
WAN01BTCY_at												
WAN01BTCZ_x_at												
WAN01BTD4_at												
WAN01BTE0_at												
WAN01BTRQ_at			SAR1133									
WAN01BTRU_x_at			SAR1136a									
WAN01BTWH_at			SAR1303									
WAN01BU0Q-seg2_at			SAR1447			MW1324						
WAN01BU2V_at			SAR1521									
WAN01BU3A_at			SAR1534									
WAN01BU3M_at												
WAN01BU3T_at			SAR1553									
WAN01BUB0_at												

Systematic	COL	N315	MRSA	MSSA	Mu50	MW2	GENE NAME	GenBank ID	GenBank Desc.	Protein Function	TIGR Main Role	TIGR Sub Role
WAN01BUBX_at												
WAN01BUCD_at												
WAN01BUCI_at			SAR1860									
WAN01BUD8_at												
WAN01BUDN_at												
WAN01BUDO_at												
WAN01BUDS_at			SAR1908									
WAN01BUDW_at												
WAN01BUE7_at												
WAN01BUEG_at												
WAN01BUIS_x_at			SAR2088									
WAN01BUIU_x_at			SAR2090									
WAN01BUJ8_at												
WAN01BUJD_at												
WAN01BUJF_at			SAR2114									
WAN01BUJG_at			SAR2115									
WAN01BUM7_at												
WAN01BUON_at			SAR2289a									
WAN01BUOX_at			SAR2297a									
WAN01BUOY_at			SAR2297b									
WAN01BUT1_s_at												
WAN01BUUK_at			SAR2494									
WAN01BUVL_at												
WAN01BUVW_at												
WAN01BUVX_at												
WAN01BUX0_at												
WAN01BUX3_at												
WAN01BUX4_at												
WAN01BUYQ_at												
WAN01BUYZ_at												
WAN01BV0F_at												
WAN01BV10_x_at												
WAN01BV21_at												
WAN01BW3M_x_at												
WAN01BWZ7_at												
WAN01BX0L_at												
WAN01BX0R_at				SAS0030								
WAN01BX10_at												
WAN01BX13_at												
WAN01BX4S_at												
WAN01BXBA_x_at												
WAN01BXBJ_at												
WAN01BXDL_at												
WAN01BXGF_x_at												
WAN01BXQZ_at												
WAN01BXVP_at												
WAN01BXY7_at												
WAN01BY0E_at												
WAN01BY3W_at						MW1324						
WAN01BY5D_at												
WAN01BYLV_x_at												
WAN01BYTK_at												
WAN01BYU4_at				SAS2278a		MW2309						
WAN01BZ41_at												
WAN01BZ42_at												
WAN01BZ43_at												
WAN01BZ44_at												
WAN01BZ45_at												
WAN01BZ47_at												
WAN01BZ48_at												
WAN01BZ49_at												
WAN01BZ4A_at												
WAN01BZ50_at												
WAN01BZ51_at												
WAN01BZ52_at												
WAN01BZ54_at												
WAN01BZ55_at												
WAN01C1E4_at												
WAN01C1EJ_at												
WAN01C1PZ_at												
WAN01C1RL_x_at												
WAN01C1ST_s_at												
WAN01C26O_at												
WAN01C299_at												
WAN01C2H9_at												
WAN01C2HO_at												
WAN01C2TP_at												
WAN01C2V3_at												
WAN01C2V7_at												
WAN01C3MI_at		SAV039			SAV0396							
WAN01C4US_at		SAV199			SAV1990							
WAN01C7GQ_at												
WAN01C7NC_at												
WAN01C8MO_at												
WAN01C8OH_x_at												
WAN01C8OY_at												
WAN01C8P5_at												
WAN01C8TY_at												
