# Supplementary material for: Changes in the Staphylococcus aureus Transcriptome during Early Adaptation to the Lung
Source: PLoS One. 2012 Aug 2;7(8):e41329. doi: 10.1371/journal.pone.0041329 (PMC3410880; doi:10.1371/journal.pone.0041329)
Supplement: Table S4 — Disposition of Pathogenicity Islands and Mobile Genetic Elements in S. aureus strain JP1. Column 1, genome locus; column 2, notes on the presence and arrangement of the genetic element within S. aureus strain JP1. (PDF) [file pone.0041329.s006.pdf]

Supplemental table 4: Disposition of Pathogenicity Islands and Mobile Genetic Elements in *S. aureus* strain JP1

Island/MGE	Comments
SCC*mec*	Not present
ΦCOL	62 of 72 *orfs* absent. The 10 present code for unknown functions
νSaα	Contains 5' genes from Type II (MW0368 - MW0381 except for MW0376) but contains only *set18*, *20*, *23*. These are same as *set8*, *10* and *12* of *N316*. Has *set7-13*, *set15* of Type I of N315, so region is a mix of type I and II. Missing restriction mod system *hsdM*, *hsdS*. Missing lipoprotein cluster *lpl1-9* (N315) or *lpl10-14* (MW2).
νSaβ	Type II but contains *splA* and *splC* (type I)
νSaγ	Complete except missing COL-SA1163
ϕSa1mu	58 of 71 genes not found. Of 13 present, 10 are also in ϕCOL or ϕSa□.
ϕSa2mw	Not present in JP1, but 3 genes of unknown function located in ϕSa2mw are found in JP1
ϕSa3	Similar to MW2, but contains 11 alleles found in N315 version
ϕSa4	Not found in JP1
