# Supplementary material for: Changes in the Staphylococcus aureus Transcriptome during Early Adaptation to the Lung
Source: PLoS One. 2012 Aug 2;7(8):e41329. doi: 10.1371/journal.pone.0041329 (PMC3410880; doi:10.1371/journal.pone.0041329)
Supplement: Table S5 — Table of Affymetrix GPL4047 gene chip probes upregulated in cells harvested after 30′ in vivo compared to stationary phase cells. Columns 1 through 16 give the Affymetrix probe identifier, the log2 probe intensities for the stationary phase, early log phase, 30′, 120, and 360′ in vivo samples, the S. aureus strain COL, N315, MSRA, MSSA, Mu50, and MW2 gene designations, the gene name, the gene product function, the TIGR main role category and TIGR subcategory role, respectively. The following 4 columns give the fold change difference from stationary phase for the early log phase (LB), 30′ (30′ in vivo), 120′ (120′ in vivo) and 360′ (360′ in vivo) derived cells. The values are color coded with green indicating significantly downregulated and dark gold indicating significantly upregulated. White indicates no significant difference. (PDF) [file pone.0041329.s007.pdf]

																Fold change vs stationary phase			
Systematic	Stat.	Log	30'	120'	360'	COL	N315	MRSA	MSSA	Mu50	MW2	GENE NAME	Protein Function	TIGR Main Role	TIGR Sub Role	LB	30' in vivo	120' in vivo	360' in vivo
WAN014FVH_at		0.9	7.6	4.3	3.7	SA1407	SA1203	SAR1384	SAS1311	SAV1371	MW1258	trpF	N-(5phosphoribosyl)-anthranilate isomerase	Amino acid biosynthesis	Aromatic amino acid family				
WAN014FVL_at	1.9	2.2	44.4	19.0	13.9	SA1409	SA1205	SAR1386	SAS1313	SAV1373	MW1260	trpA	tryptophan synthase, alpha subunit	Amino acid biosynthesis	Aromatic amino acid family	1.14	23.44	10.03	7.35
WAN014FVJ_at	1.4	1.3	22.8	10.7	8.9	SA1408	SA1204	SAR1385	SAS1312	SAV1372	MW1259	trpB	tryptophan synthase, beta subunit	Amino acid biosynthesis	Aromatic amino acid family	-1.09	16.42	7.73	6.46
WAN014G2V_at	1.4	1.6	3.6	3.4	3.1	SA0429	SA0345	SAR0354	SAS0333	SAV0357	MW0333	metE2	homocysteine S-methyltransferase	Amino acid biosynthesis	Aspartate family	1.17	2.67	2.54	2.29
WAN014FW0_at	1.6	2.0	3.9	9.3	7.8	SA1428	SA1225	SAR1405	SAS1334	SAV1393	MW1281	lysC	aspartate kinase, monofunctional class, putative	Amino acid biosynthesis	Aspartate family	1.22	2.44	5.76	4.85
WAN014G24_at	2.5	3.9	5.8	5.2	9.3	SA0428	SA0344	SAR0353	SAS0332	SAV0356	MW0332	metE	5-methyltetrahydropteroyltriglutamate--homocysteine methyltransferase	Amino acid biosynthesis	Aspartate family	1.56	2.32	2.10	3.73
WAN014GRK_at		0.9	3.9	5.1	6.7	SA0964	SA0822	SAR0923	SAS0831	SAV0961	MW0843	argG	argininosuccinate synthase	Amino acid biosynthesis	Glutamate family				
WAN014GYD_at	3.0	1.6	281.8	236.8	105.6	SA0515	SA0431	SAR0472	SAS0430	SAV0473	MW0427	gltD	glutamate synthase, small subunit	Amino acid biosynthesis	Glutamate family	-1.85	93.27	78.39	34.96
WAN014GYA_at	2.3	1.7	116.1	114.9	56.2	SA0514	SA0430	SAR0471	SAS0429	SAV0472	MW0426	gltB	glutamate synthase, large subunit	Amino acid biosynthesis	Glutamate family	-1.39	49.52	49.00	23.97
WAN014GRH_at	1.1	1.1	7.7	10.9	13.0	SA0963	SA0821	SAR0922	SAS0830	SAV0960	MW0842	argH	argininosuccinate lyase	Amino acid biosynthesis	Glutamate family	1.04	7.03	9.92	11.87
WAN014GDB_at			3.9	2.4	1.4	SA2702	SA2470	SAR2760	SAS2563	SAV2678	MW2597	hisD	histidinol dehydrogenase	Amino acid biosynthesis	Histidine family				
WAN014GD9_at			3.2	2.2	1.4	SA2701	SA2469					hisC2	histidinol-phosphate aminotransferase, putative	Amino acid biosynthesis	Histidine family				
WAN014GD7_at		1.1	8.4	5.5	2.6	SA2700	SA2468	SAR2758	SAS2561	SAV2676	MW2595	hisB	imidazoleglycerol-phosphate dehydratase	Amino acid biosynthesis	Histidine family				
WAN014GD5_at			7.0	5.0	2.5	SA2699	SA2467	SAR2757	SAS2560	SAV2675	MW2594	hisH	amidotransferase HisH	Amino acid biosynthesis	Histidine family				
WAN014IOS_at	1.6	1.2	68.6	37.2	15.2	SA2696	SA2464	SAR2754	SAS2557	SAV2672	MW2591	hisI	phosphoribosyl-ATP pyrophosphatase/phosphoribosyl-AMP cyclohydrolase	Amino acid biosynthesis	Histidine family	-1.34	42.40	23.02	9.39
WAN014IOR_at	1.5		18.6	13.3	7.9	SA2697	SA2465	SAR2755	SAS2558	SAV2673	MW2592	hisF	imidazoleglycerol phosphate synthase, cyclase subunit	Amino acid biosynthesis	Histidine family		12.10	8.67	5.14
WAN014GD3_at	1.0	0.8	7.7	5.1	2.3	SA2698	SA2466	SAR2756	SAS2559	SAV2674	MW2593	hisA	phosphoribosylformimino-5-aminoimidazole carboxamide ribotide isomerase	Amino acid biosynthesis	Histidine family	-1.13	8.11	5.32	2.37
WAN014GDF_at	1.3		4.1	2.7	1.6	SA2704	SA2472	SAR2762	SAS2565	SAV2680	MW2599	hisZ	ATP phosphoribosyltransferase	Amino acid biosynthesis	Histidine family		3.28	2.14	1.25
WAN014GDC_at	0.7		1.9	1.2	0.9	SA2703	SA2471	SAR2761	SAS2564	SAV2679	MW2598	hisG	ATP phosphoribosyltransferase	Amino acid biosynthesis	Histidine family		2.54	1.56	1.19
WAN014G4I_at			31.0	21.7	11.5	SA2049	SA1865	SAR2147	SAS1965	SAV2060	MW1984	leuD	3-isopropylmalate dehydratase, small subunit	Amino acid biosynthesis	Pyruvate family				
WAN014G44_at			29.1	24.7	15.5	SA2045	SA1861	SAR2143	SAS1961	SAV2056	MW1980	ilvC	ketol-acid reductoisomerase	Amino acid biosynthesis	Pyruvate family				
WAN014G4A_at	1.2	0.8	48.0	38.6	21.5	SA2048	SA1864	SAR2146	SAS1964	SAV2059	MW1983	leuC	3-isopropylmalate dehydratase, large subunit	Amino acid biosynthesis	Pyruvate family	-1.52	40.09	32.19	17.94
WAN014G46_at	1.6		54.1	50.0	28.9	SA2046	SA1862	SAR2144	SAS1962	SAV2057	MW1981	leuA	2-isopropylmalate synthase	Amino acid biosynthesis	Pyruvate family		34.76	32.11	18.53
WAN014G4M_at	1.0		33.0	26.0	16.5	SA2050	SA1866	SAR2148	SAS1966	SAV2061	MW1985	ilvA2	threonine dehydratase, biosynthetic, putative	Amino acid biosynthesis	Pyruvate family		33.46	26.37	16.76
WAN014G40_at	2.7	2.1	86.8	75.9	44.2	SA2043	SA1859	SAR2141	SAS1959	SAV2054	MW1978	ilvB	acetolactate synthase, large subunit, biosynthetic type	Amino acid biosynthesis	Pyruvate family	-1.29	31.68	27.71	16.14
WAN014G41_at	1.3	0.9	33.5	32.7	18.1	SA2044	SA1860			SAV2055	MW1979	ilvN	acetolactate synthase, small subunit	Amino acid biosynthesis	Pyruvate family	-1.44	26.34	25.70	14.23
WAN014G48_at	1.5		37.2	36.0	25.8	SA2047	SA1863	SAR2145	SAS1963	SAV2058	MW1982	leuB	3-isopropylmalate dehydrogenase	Amino acid biosynthesis	Pyruvate family		24.38	23.61	16.89
WAN014G3T_at	3.9	1.4	72.8	76.5	39.9	SA2042	SA1858	SAR2140	SAS1958	SAV2053	MW1977	ilvD	dihydroxy-acid dehydratase	Amino acid biosynthesis	Pyruvate family	-2.71	18.85	19.80	10.34
WAN014FYQ_at	3.3	3.5	13.3	14.6	10.1	SA1772	SA1544	SAR1800	SAS1649	SAV1723	MW1665	serC	aminotransferase, class V	Amino acid biosynthesis	Serine family	1.07	4.02	4.42	3.07
WAN014FYS_at	5.2	7.1	19.3	19.3	14.7	SA1773	SA1545	SAR1801	SAS1650	SAV1724	MW1666	serA	D-3-phosphoglycerate dehydrogenase	Amino acid biosynthesis	Serine family	1.38	3.74	3.74	2.85
WAN014GXQ_at	1.2	14.0	3.8	2.2	2.4	SA0503	SA0419	SAR0460	SAS0418	SAV0460	MW0415	metB	trans-sulfuration enzyme family protein	Amino acid biosynthesis	Serine family	12.15	3.34	1.88	2.13
WAN014G3D_at	1.8	2.4	4.7	4.3	3.7	SA0430	SA0346	SAR0355	SAS0334	SAV0358	MW0334	metB	trans-sulfuration enzyme family protein	Amino acid biosynthesis	Serine family	1.37	2.66	2.46	2.10
WAN014HCA_at	3.9	8.2	22.0	11.6	11.5	SA1709	SA1487	SAR1742	SAS1591	SAV1662	MW1606	folC	folylpolyglutamate synthase	Biosynthesis of cofactors, prosthetic groups, and carriers	Folic acid	2.09	5.60	2.94	2.93
WAN014FY6_at	3.6	4.2	12.8	8.1	5.3	SA2396	SA2186					sirB	uroporphyrinogen III methylase SirB, putative	Biosynthesis of cofactors, prosthetic groups, and carriers	Heme, porphyrin, and cobalamin	1.18	3.57	2.24	1.47
WAN014HR9_at	1.4	2.4	3.4	2.9	3.0	SA1950	SA1707	SAR1982	SAS1813	SAV1891	MW1832	cobQ	cobyric acid synthase	Biosynthesis of cofactors, prosthetic groups, and carriers	Heme, porphyrin, and cobalamin	1.77	2.46	2.09	2.18
WAN014GXA_at	2.0	1.1	4.6	3.4	4.3	SA0491	SA0410	SAR0450	SAS0408	SAV0450	MW0406		cobalamin synthesis protein, putative	Biosynthesis of cofactors, prosthetic groups, and carriers	Heme, porphyrin, and cobalamin	-1.88	2.32	1.70	2.14
WAN014G83_at	0.8	0.9	1.6	1.4	1.3	SA2598	SA2368	SAR2662	SAS2468	SAV2582	MW2502		cobalamin synthesis protein, putative	Biosynthesis of cofactors, prosthetic groups, and carriers	Heme, porphyrin, and cobalamin	1.13	2.09	1.85	1.72
WAN014GSN_at	9.0	23.2	23.8	23.9	25.2	SA1049	SA0894	SAR1015	SAS0977	SAV1041	MW0925		1,4-dihydroxy-2-naphthoate octaprenyltransferase, putative	Biosynthesis of cofactors, prosthetic groups, and carriers	Menaquinone and ubiquinone	2.57	2.63	2.64	2.78
WAN014GO7_at	3.0	3.8	6.6	6.5	4.8	SA0771	SA0666	SAR0764	SAS0676	SAV0711	MW0673	ptpS	6-pyruvoyl tetrahydrobiopterin synthase, putative	Biosynthesis of cofactors, prosthetic groups, and carriers	Other	1.28	2.23	2.18	1.62
WAN014GD8_at	9.1	25.2	19.7	13.2	18.4	SA2561	SA2334	SAR2626	SAS2432	SAV2546	MW2467	mvaS	hydroxymethylglutaryl-CoA synthase	Biosynthesis of cofactors, prosthetic groups, and carriers	Other	2.77	2.17	1.45	2.03
WAN014G5C_at	8.4	10.7	38.3	31.2	28.4	SA2122	SA1932	SAR2218	SAS2033	SAV2130	MW2054	coaW	pantothenate kinase, putative	Biosynthesis of cofactors, prosthetic groups, and carriers	Pantothenate and coenzyme A	1.27	4.58	3.73	3.39
WAN014HZ5_at	1.8	5.4	11.7	7.4	5.6	SA2088	SA1898	SAR2184	SAS1999	SAV2095	MW2020		sceD protein, putative	Biosynthesis of cofactors, prosthetic groups, and carriers	Thiamine	2.97	6.47	4.09	3.07
WAN014HF8_at	1.0	2.0	2.6	2.6	2.2	SA1764	SA1537	SAR1793	SAS1642	SAV1715	MW1658	thiI	thiazole biosynthesis protein ThiI	Biosynthesis of cofactors, prosthetic groups, and carriers	Thiamine	2.00	2.62	2.62	2.20
WAN014GD6_at	2.9	9.5	132.4	97.5	87.1	SA2557	SA2332	SAR2624	SAS2430	SAV2544	MW2465	similar to S	CHAP domain protein	Cell envelope	Biosynthesis and degradation of murein sacculus and peptidoglycan	3.27	45.50	33.52	29.93
WAN014GMS_at	36.7	71.8	301.8	293.2	235.4	SA0723	SA0620	SAR0675	SAS0630	SAV0665	MW0627		LysM domain protein	Cell envelope	Biosynthesis and degradation of murein sacculus and peptidoglycan	1.95	8.22	7.99	6.41
WAN014GY1_at	1.4	1.4	5.4	3.7	3.4	SA0507	SA0423	SAR0464	SAS0422		MW0419	similar to S	LysM domain protein	Cell envelope	Biosynthesis and degradation of murein sacculus and peptidoglycan	-1.01	3.83	2.64	2.42
WAN014HJI_at	3.5	8.0	19.2	15.7	15.5	SA1825	SA1593	SAR1857	SAS1698	SAV1775	MW1715		N-acetylmuramoyl-L-alanine amidase	Cell envelope	Biosynthesis and degradation of surface polysaccharides and lipopolysaccharides	2.29	5.49	4.48	4.41
WAN014HJZ_at	19.5	35.9	82.6	61.6	57.1	SA0938	SA0796	SAR0897	SAS0805	SAV0935	MW0817	dltD	extramembranal protein	Cell envelope	Biosynthesis and degradation of surface polysaccharides and lipopolysaccharides	1.84	4.23	3.16	2.92
WAN014HJV_at	16.1	35.8	61.2	53.3	52.3	SA0936	SA0794	SAR0895	SAS0803	SAV0933	MW0815	dltB	dltB protein	Cell envelope	Biosynthesis and degradation of surface polysaccharides and lipopolysaccharides	2.22	3.80	3.31	3.25
WAN014HJX_at	2.5	4.2	7.5	5.2	4.7	SA0937	SA0795	SAR0896	SAS0804	SAV0934	MW0816	dltC	D-alanyl carrier protein	Cell envelope	Biosynthesis and degradation of surface polysaccharides and lipopolysaccharides	1.68	3.01	2.11	1.90
WAN014G08_at	1.4	2.0	3.7	2.9	4.5	SA2446	SA2231	SAR2532	SAS2334	SAV2442	MW2366		Nucleoside-diphosphate-sugar epimerase/dehydratase, putative	Cell envelope	Biosynthesis and degradation of surface polysaccharides and lipopolysaccharides	1.41	2.55	2.03	3.11
WAN014GT7_s_at	50.3	39.1	110.4	108.6	82.6	SA1062	trunca	SAR1026	SAS0988	SAV1052	MW0936	atl	bifunctional autolysin precursor	Cell envelope	Biosynthesis and degradation of surface polysaccharides and lipopolysaccharides	-1.29	2.19	2.16	1.64
WAN014GMW_at	3.8	4.9	12.0	9.3	11.6	SA0263	SA0265	SAR0273	SAS0252	SAV0276	MW0252	lytM	peptidoglycan hydrolase	Cell envelope	Biosynthesis of murein sacculus and peptidoglycan	1.29	3.17	2.46	3.08
WAN014GMJ_at	2.7	3.9	7.2	5.4	4.9	SA0699	SA0598	SAR0652	SAS0608	SAV0642	MW0604	pbp4	penicillin-binding protein 4	Cell envelope	Biosynthesis of murein sacculus and peptidoglycan	1.42	2.68	1.98	1.81
WAN014HRE_at	4.7	7.4	12.0	8.6	9.1	SA1951	SA1708	SAR1983	SAS1814	SAV1892	MW1833		UDP-N-acetylmuramyl tripeptide synthetase, putative	Cell envelope	Biosynthesis of murein sacculus and peptidoglycan	1.57	2.55	1.83	1.94
WAN014HYP_at	3.4	8.3	8.5	8.6	6.5	SA1194	SA1024	SAR1157	SAS1115	SAV1181	MW1064	pbp1	penicillin-binding protein 1	Cell envelope	Biosynthesis of murein sacculus and peptidoglycan	2.46	2.53	2.55	1.95
WAN014G7P_at	42.9	213.7	750.9	763.3	784.1	SA2584	SA2356	SAR2650	SAS2455	SAV2569	MW2490	isaA	immunodominant antigen A	Cell envelope	Other	4.99	17.52	17.81	18.30
WAN014ITL_at	1.9	5.2	11.3	10.9	10.3	SA0790	SA0684	SAR0783	SAS0694	SAV0729	MW0691		integral membrane domain protein	Cell envelope	Other	2.75	5.97	5.78	5.47
WAN014HYM_at	8.0	14.0	44.6	35.2	35.0	SA2082	SA1893	SAR2179	SAS1994	SAV2090	MW2013	yidC	membrane protein, putative	Cell envelope	Other	1.77	5.61	4.43	4.41
WAN014FWP_at	1.1	3.6	2.9	3.2	3.4	SA1468	SA1265	SAR1445	SAS1375	SAV1432	MW1321		membrane protein, putative	Cell envelope	Other	3.25	2.57	2.87	3.01
WAN01BTFS_at	26.8	250.0	109.9	97.1	103.6			SAR0706					putative membrane protein	Cell envelope	Role category not yet assigned	9.34	4.11	3.63	3.87
WAN014GFX_at	63.5	65.1	690.6	897.3	714.3	SA2731	SA2494	SAR2790	SAS2587	SAV2704	MW2623	cspB	cold-shock protein CspB	Cellular processes	Adaptations to atypical conditions	1.03	10.87	14.13	11.24
WAN014HER_at	355.6	151.1	1112.2	1248.0	1013.5	SA0861	SA0747	SAR0848	SAS0757	SAV0816	MW0770	cspC	cold-shock protein CspC	Cellular processes	Adaptations to atypical conditions	-2.35	3.13	3.51	2.85
WAN014G0M_at	3.5	15.4	9.7	11.3	9.1	SA0425	SA0341	SAR0350	SAS0329	SAV0353	MW0329	ltrA	low temperature requirement protein LtrA	Cellular processes	Adaptations to atypical conditions	4.45	2.79	3.26	2.62
WAN014G9F_at	1.3		3.1	1.2	0.6	SA2628	SA2406	SAR2691	SAS2498	SAV2613	MW2532	gbsA	glycine betaine aldehyde dehydrogenase	Cellular processes	Adaptations to atypical conditions		2.44	-1.06	-2.06
WAN014G9D_at	1.6	1.1	3.9	2.2	1.7	SA2627	SA2405	SAR2690	SAS2497	SAV2612	MW2531	betA	choline dehydrogenase	Cellular processes	Adaptations to atypical conditions	-1.47	2.38	1.34	1.04
WAN014ITZ_at	6.3	22.8	23.6	21.0	19.2	SA1807	SA1578	SAR1842	SAS1683	SAV1759	MW1700		rhodanese family protein	Cellular processes	Detoxification	3.63	3.74	3.34	3.05
WAN014FZI_at	4.0	7.1	12.8	9.0	6.9	SA1824						arsC	arsenate reductase	Cellular processes	Detoxification	1.77	3.19	2.26	1.71
WAN014GFD_at	1.6	2.0	4.5	4.3	3.4	SA2713	SA2481	SAR2771	SAS2575	SAV2689	MW2609		rhodanese family protein	Cellular processes	Detoxification	1.25	2.79	2.67	2.14
WAN014I6F_at	3.2	3.7	7.4	6.4	5.5	SA1327	SA1148	SAR1282	SAS1240	SAV1308	MW1190		aluminum resistance protein	Cellular processes	Detoxification	1.17	2.34	2.02	1.73
WAN014GEO_at	3.4	3.3	7.4	6.0	6.3	SA2738	SA2501	SAR2798	SAS2594	SAV2712	MW2630	thdF	thiophene and furan oxidation protein ThdF	Cellular processes	Detoxification	-1.03	2.15	1.75	1.83
WAN014G7H_at	1.2	0.8	83.1	95.1	54.0	SA2581	SA2353	SAR2648	SAS2452	SAV2566	MW2487		staphyloxanthin biosynthesis protein	Cellular processes	Pathogenesis	-1.49	70.33	80.40	45.67
WAN014G7Q_at	6.3	90.4	307.4	280.7	313.9	SA2291	SA2093	SAR2383	SAS2189	SAV2299	MW2217	ssaA	secretory antigen precursor SsaA	Cellular processes	Pathogenesis	14.39	48.94	44.68	49.97
WAN014G7Z_at	5.6	33.0	150.1	125.9	91.2	SA2295	SA2097	SAR2388	SAS2194	SAV2304	MW2222	similar to S	secretory antigen precursor SsaA, putative	Cellular processes	Pathogenesis	5.90	26.82	22.49	16.30
WAN014HJS_at	13.5	18.5	45.8	42.3	41.4	SA0935	SA0793	SAR0894	SAS0802	SAV0932	MW0814	dltA	D-alanine-D-alanyl carrier protein ligase DltA	Cellular processes	Toxin production and resistance	1.37	3.39	3.13	3.06
WAN014HC6_at	7.2	11.3	23.3	17.8	18.8	SA0810	SA0702	SAR0801	SAS0712	SAV0747	MW0709	llm	undecaprenyl-phosphate alpha-N-acetylglucosaminyltransferase	Cellular processes	Toxin production and resistance	1.57	3.25	2.48	2.61
WAN014GNE_at	4.6	9.2	13.1	10.7	11.8	SA0743	SA0638	SAR0736	SAS0648	SAV0683	MW0645	bacA	bacitracin resistance protein	Cellular processes	Toxin production and resistance	2.01	2.87	2.34	2.58
WAN014HJY_at	6.5	18.6	15.8	16.9	16.1	SA1837	SA1608	SAR1870	SAS1711	SAV1790	MW1728	metK	S-adenosylmethionine synthetase	Central intermediary metabolism	One-carbon metabolism	2.87	2.43	2.60	2.48
WAN014FSG_at	1.2	1.4	3.8	4.0	3.5	SA1357	SA1160	SAR1334	SAS1264	SAV1324	MW1211	nuc	staphylococcal nuclease family protein	DNA metabolism	Degradation of DNA	1.21	3.23	3.36	2.92
WAN014H48_at	7.2	21.2	15.2	11.9	14.2	SA1567	SA1353	SAR1600	SAS1461	SAV1522	MW1475		exodeoxyribonuclease VII, small subunit	DNA metabolism	Degradation of DNA	2.93	2.10	1.65	1.96
WAN014GVD_at	30.2	40.7	241.9	215.8	330.1	SA0438	SA0353	SAR0363	SAS0342	SAV0366	MW0342	ssb	single-stranded DNA-binding protein family	DNA metabolism	DNA replication, recombination, and repair	1.35	8.02	7.15	10.94
WAN014FUQ_at	2.7	6.9	11.7	9.7	9.7	SA1390	SA1189	SAR1367	SAS1295	SAV1355	MW1242	parC	DNA topoisomerase IV, A subunit	DNA metabolism	DNA replication, recombination, and repair	2.57	4.37	3.62	3.63
WAN014FUO_at	3.6	7.4	14.2	15.4	13.3	SA1389	SA1188	SAR1366	SAS1294	SAV1354	MW1241	parE	DNA topoisomerase IV, B subunit	DNA metabolism	DNA replication, recombination, and repair	2.07	3.96	4.29	3.71
WAN014H6W_at	8.2	12.9	30.0	23.2	26.1	SA1614	SA1386	SAR1634	SAS1495	SAV1557	MW1509	nfo	endonuclease IV	DNA metabolism	DNA replication, recombination, and repair	1.58	3.67	2.84	3.19
WAN014I32_at	8.7	19.0	26.5	29.4	27.1	SA1267	SA1093	SAR1226	SAS1184	SAV1250	MW1133	topA	DNA topoisomerase I	DNA metabolism	DNA replication, recombination, and repair	2.18	3.04	3.37	3.11
WAN014HBI_at	8.7	10.8	23.5	20.4	19.8	SA1696	SA1467	SAR1721	SAS1577	SAV1641	MW1591	ruvB	Holliday junction DNA helicase RuvB	DNA metabolism	DNA replication, recombination, and repair	1.24	2.71	2.35	2.27
WAN014I5W_at	2.9	7.1	6.7	4.4	4.4	SA1316	SA1138	SAR1272	SAS1230	SAV1297	MW1179	hexB	DNA mismatch repair protein HexB	DNA metabolism	DNA replication, recombination, and repair	2.48	2.35	1.54	1.52
WAN014FX6_at	4.6	6.4	9.7	9.9	8.3	SA1489	SA1282	SAR1460	SAS1392	SAV1449	MW1339	recU	recombination protein U	DNA metabolism	DNA replication, recombination, and repair	1.39	2.11	2.15	1.82
WAN014H49_at	2.2	4.0	4.4	3.1	3.7	SA0627	SA0538	SAR0586	SAS0539	SAV0581	MW0536	ung	uracil-DNA glycosylase	DNA metabolism	DNA replication, recombination, and repair	1.80	2.00	1.43	1.68
WAN014G5R_at	1.5	6.0	5.6	4.7	5.1	SA2545	SA2319	SAR2611	SAS2417	SAV2531	MW2452	sdhB	L-serine dehydratase, iron-sulfur-dependent, beta subunit	Energy metabolism	Amino acids and amines	3.96	3.71	3.08	3.34
WAN014H5N_at	3.4	18.0	10.0	7.1	7.6	SA1593	SA1365	SAR1612	SAS1473	SAV1535	MW1487	gcvPB	glycine cleavage system P protein, subunit 2	Energy metabolism	Amino acids and amines	5.25	2.92	2.09	2.21
WAN014G5P_at	5.4	19.8	15.3	13.3	13.2	SA2544	SA2318	SAR2610	SAS2416	SAV2530	MW2451	sdhA	L-serine dehydratase, iron-sulfur-dependent, alpha subunit	Energy metabolism	Amino acids and amines	3.66	2.83	2.47	2.44
WAN014H5P_at	2.3	12.3	5.5	4.8	4.3	SA1594	SA1366	SAR1613	SAS1474	SAV1536	MW1488	gcvPA	glycine cleavage system P protein, subunit 1	Energy metabolism	Amino acids and amines	5.37	2.42	2.09	1.85
WAN014G25_at	1.2	1.0	2.6	2.5	1.1	SA0162	SA0171	SAR0178	SAS0152	SAV0177	MW0151	fdh	formate dehydrogenase, NAD-dependent, putative	Energy metabolism	Anaerobic	-1.21	2.20	2.04	-1.07
WAN014FXB_at	16.1	18.2	80.4	77.6	116.8	SA1525	SA1315	SAR1492	SAS1424	SAV1484	MW1372	fer	ferredoxin, putative	Energy metabolism	Electron transport	1.13	5.00	4.82	7.26
WAN014HSY_at	4.8	13.7	11.5	10.8	12.3	SA1095	SA0938	SAR1060	SAS1022	SAV1087	MW0969	cydB	cytochrome d ubiquinol oxidase, subunit II	Energy metabolism	Electron transport	2.83	2.36	2.22	2.54
WAN014I27_at	5.2	12.4	17.2	7.1	5.7	SA0135	SA0143	SAR0150	SAS0123	SAV0148	MW0123	adhE	alcohol dehydrogenase, iron-containing	Energy metabolism	Fermentation	2.38	3.31	1.36	1.09
WAN014HDR_at	48.1	126.6	232.7	210.1	205.6	SA0838	SA0727	SAR0828	SAS0738	SAV0772	MW0734	gapA1	glyceraldehyde 3-phosphate dehydrogenase	Energy metabolism	Glycolysis/gluconeogenesis	2.63	4.84	4.37	4.28
WAN014GI3_at	119.4	418.2	479.3	254.3	304.7	SA0222	SA0232	SAR0234	SAS0217	SAV0241	MW0217	ldh1	L-lactate dehydrogenase	Energy metabolism	Glycolysis/gluconeogenesis	3.50	4.02	2.13	2.55
WAN014HDT_at	10.4	31.2	32.4	29.3	27.9	SA0839	SA0728	SAR0829	SAS0739	SAV0773	MW0735	pgk	phosphoglycerate kinase	Energy metabolism	Glycolysis/gluconeogenesis	3.01	3.12	2.83	2.69
WAN014HDX_at	5.3	15.5	14.8	11.5	11.6	SA0841	SA0730	SAR0831	SAS0741	SAV0775	MW0737	pgm	phosphoglycerate mutase, 2,3-bisphosphoglycerate-independent	Energy metabolism	Glycolysis/gluconeogenesis	2.93	2.81	2.18	2.20

Systematic	Stat.	Log	30'	120'	360'	COL	N315	MRSA	MSSA	Mu50	MW2	GENE NAME	Protein Function	TIGR Main Role	TIGR Sub Role	Fold change vs stationary phase			
																LB	30' in vivo	120' in vivo	360' in vivo
WAN014I1O_at	16.3	29.7	61.1	48.1	48.9	SA1240	SA1069	SAR1202	SAS1160	SAV1226	MW1109		conserved hypothetical protein	Energy metabolism	Other	1.82	3.75	2.95	3.00
WAN014HC4_at	4.9	5.3	10.4	7.3	9.0	SA0805	SA0697	SAR0796	SAS0707	SAV0742	MW0704		glycerate kinase family protein	Energy metabolism	Other	1.08	2.13	1.49	1.85
WAN014H4G_at	12.9	30.9	27.0	24.4	26.6	SA1571	SA1357	SAR1604	SAS1465	SAV1526	MW1479	accC	acetyl-CoA carboxylase, biotin carboxylase	Fatty acid and phospholipid metabolism	Biosynthesis	2.41	2.10	1.90	2.07
WAN014HVH_at	5.3	16.3	78.1	80.0	85.2	SA1136	SA0975	SAR1100	SAS1061	SAV1127	MW1009		conserved hypothetical protein	Hypothetical proteins	Conserved	3.06	14.70	15.05	16.03
WAN014HBR_at	29.4	64.0	253.4	243.7	324.6	SA1701	SA1472	SAR1726	SAS1582	SAV1646	MW1596		conserved hypothetical protein	Hypothetical proteins	Conserved	2.18	8.62	8.29	11.04
WAN014FTV_at	4.5	8.2	25.9	21.0	18.6	SA1380	SA1179	SAR1355	SAS1284	SAV1344	MW1231		conserved hypothetical protein	Hypothetical proteins	Conserved	1.81	5.71	4.64	4.11
WAN014H4T_at	4.1	5.5	22.9	24.1	17.3	SA0639	SA0550	SAR0599	SAS0552	SAV0593	MW0548		conserved hypothetical protein	Hypothetical proteins	Conserved	1.35	5.59	5.89	4.23
WAN014G2D_at	1.3		6.7	7.5	5.9	SA2477	SA2256	SAR2555	SAS2359	SAV2468	MW2392		conserved hypothetical protein	Hypothetical proteins	Conserved		5.27	5.93	4.64
WAN014G2G_at	1.8		8.6	8.7	7.2	SA2478	SA2257	SAR2556	SAS2360	SAV2469	MW2393		conserved hypothetical protein	Hypothetical proteins	Conserved		4.74	4.76	3.99
WAN014GQ7_at	2.5	7.0	10.5	9.6	8.8		SA0647	SAR0745	SAS0657	SAV0692	MW0654		Membrane protein	Hypothetical proteins	Conserved	2.80	4.22	3.87	3.55
WAN014G2J_at	1.8		7.5	6.8	6.2	SA2479	SA2258	SAR2557	SAS2361	SAV2470	MW2394		conserved hypothetical protein	Hypothetical proteins	Conserved		4.15	3.77	3.42
WAN014FXC_at	6.7	17.1	26.5	17.7	12.3	SA2371	SA2163	SAR2462	SAS2265	SAV2373	MW2295		conserved hypothetical protein	Hypothetical proteins	Conserved	2.53	3.93	2.62	1.82
WAN014GNJ_at	3.5	8.9	13.4	11.5	12.0	SA0752	SA0648	SAR0746	SAS0658	SAV0693	MW0655		conserved hypothetical protein	Hypothetical proteins	Conserved	2.53	3.82	3.28	3.42
WAN014HPH_at	4.7	5.8	15.4	12.2	11.7	SA1925	SA1684	SAR1957	SAS1789	SAV1867	MW1807		conserved hypothetical protein	Hypothetical proteins	Conserved	1.24	3.29	2.61	2.50
WAN014HF6_at	3.1	5.1	8.0	7.1	8.8	SA1763	SA1536	SAR1792	SAS1641	SAV1714	MW1657		conserved hypothetical protein	Hypothetical proteins	Conserved	1.61	2.55	2.25	2.80
WAN014GMZ_at	7.7	9.7	18.5	20.5	13.3			SAR2093	SAS1912	SAV1992	MW1929		conserved hypothetical protein	Hypothetical proteins	Conserved	1.26	2.41	2.66	1.73
WAN014H0X_at	3.6	5.7	8.0	8.0	6.4	SA1501	SA1294	SAR1472	SAS1404	SAV1461	MW1351		conserved hypothetical protein	Hypothetical proteins	Conserved	1.57	2.23	2.22	1.78
WAN014H28_at	12.3	8.0	27.0	26.1	26.0	SA1526	SA1316	SAR1493	SAS1425	SAV1485	MW1373		conserved hypothetical protein	Hypothetical proteins	Conserved	-1.54	2.19	2.11	2.11
WAN014FUX_at	4.0	14.1	8.2	8.8	8.8	SA2306	SA2106	SAR2397	SAS2206	SAV2313	MW2234		abortive infection protein family	Hypothetical proteins	Conserved	3.56	2.08	2.22	2.22
WAN014HWQ_at	1.6	2.5	3.3	2.2	2.6	SA2052	SA1867	SAR2150	SAS1967	SAV2062	MW1986		sprt protein, putative	Hypothetical proteins	Conserved	1.55	2.06	1.40	1.67
WAN014I48_at	4.7	3.8	9.4	7.8	11.1	SA1286	SA1110	SAR1243	SAS1201	SAV1267	MW1150		conserved hypothetical protein	Hypothetical proteins	Conserved	-1.24	2.02	1.68	2.38
WAN014HWO_at	0.9	0.9	6.5	8.4	10.0	SA2051							hypothetical protein	Hypothetical proteins	Not Conserved	-1.05	7.14	9.21	11.00
WAN014H00_at	11.0	26.4	62.3	58.7	49.3	SA1482	SA1276	SAR1455	SAS1386	SAV1443	MW1332		hypothetical protein	Hypothetical proteins	Not Conserved	2.39	5.64	5.31	4.46
WAN014GCY_at	1.3		6.5	6.1	2.5	SA2695							hypothetical protein	Hypothetical proteins	Not Conserved		4.90	4.61	1.88
WAN01BQGT_at	7.7	8.5	30.1	28.6	19.9	SA2158	SA1971	SAR2259	SAS2069	SAV2167	MW2094	sepA	hypothetical protein	Hypothetical proteins	Not Conserved	1.10	3.90	3.70	2.58
WAN014I5F_at	5.1	8.9	15.2	15.8	11.9	SA1307	SA1130	SAR1264	SAS1222	SAV1288	MW1171		hypothetical protein	Hypothetical proteins	Not Conserved	1.75	2.98	3.10	2.33
WAN01BPBU_at	1.2	0.8	3.3	3.0	2.8	SA0674							hypothetical protein	Hypothetical proteins	Not Conserved	-1.44	2.85	2.63	2.39
WAN01BQPQ_at	17.6	1.7	47.5	87.5	63.3	SA2480							hypothetical protein	Hypothetical proteins	Not Conserved	-10.21	2.70	4.97	3.60
WAN014GNZ_at	3.4	3.9	8.0	7.1	6.6	SA0769	SA0664	SAR0762	SAS0674	SAV0709	MW0671		hypothetical protein	Hypothetical proteins	Not Conserved	1.16	2.35	2.08	1.94
WAN014FTR_at	5.2	12.5	12.1	11.7	12.5	SA1378	SA1178	SAR1353	SAS1283	SAV1343	MW1230		hypothetical protein	Hypothetical proteins	Not Conserved	2.40	2.31	2.25	2.38
WAN014I1T_at	37.7	73.8	85.4	101.0	66.6	SA2134	SA1944	SAR2230	SAS2045	SAV2142	MW2066		hypothetical protein	Hypothetical proteins	Not Conserved	1.96	2.27	2.68	1.77
WAN014FTT_at	2.6	6.5	5.7	6.5	7.5	SA1379							hypothetical protein	Hypothetical proteins	Not Conserved	2.55	2.23	2.53	2.92
WAN014GR0_at	1.4	0.8	3.0	3.7	3.5	SA0948	SA0806	SAR0907	SAS0815	SAV0945	MW0827		hypothetical protein	Hypothetical proteins	Not Conserved	-1.72	2.13	2.56	2.49
WAN014GL4_at	2.1	7.8	32.8	33.3	41.4		#N/A						hypothetical protein	Hypothetical proteins	Role category not yet assigned	3.66	15.41	15.66	19.46
WAN014I3B_at	2.7	6.8	17.8	18.2	25.1		#N/A						hypothetical protein	Hypothetical proteins	Role category not yet assigned	2.51	6.54	6.70	9.24
WAN014GQU x a	37.4	28.0	196.7	204.4	151.1		#N/A						hypothetical protein	Hypothetical proteins	Role category not yet assigned	-1.33	5.26	5.47	4.04
WAN014GLG_at	3.0	1.6	13.6	13.3	16.9		#N/A						hypothetical protein	Hypothetical proteins	Role category not yet assigned	-1.91	4.59	4.48	5.70
WAN01BUQ1_at	2.3	2.7	8.0	8.5	12.3		#N/A						hypothetical protein	Hypothetical proteins	Role category not yet assigned	1.19	3.49	3.72	5.39
WAN01C4TN_at	1.5	0.9	4.6	5.3	2.6					SAV1949			hypothetical protein	Hypothetical proteins	Role category not yet assigned	-1.73	3.10	3.59	1.77
WAN014GPT_at	2.3	7.4	5.7	8.5	11.9		#N/A						hypothetical protein	Hypothetical proteins	Role category not yet assigned	3.29	2.53	3.77	5.30
WAN014GKZ_at	7.5	4.0	19.6	17.2	19.6		SA1756						truncated amidase-related protein	Mobile and extrachromosomal element functions	Prophage functions	-1.88	2.63	2.30	2.63
WAN014I6P_at	3.5	1.4	8.7	7.0	5.6		SAS070			SAV2150	MW2074		transposase, degenerate	Mobile and extrachromosomal element functions	Transposon functions	-2.50	2.53	2.03	1.61
WAN014FU0_at	2.9	7.9	6.4	5.2	4.5				SAS0375		MW0374		hypothetical protein	No Data	No Data	2.74	2.21	1.80	1.56
WAN014HD0_at	10.7	26.4	32.1	27.0	27.4	SA1721	SA1498	SAR1754	SAS1603	SAV1674	MW1618	clpX	ATP-dependent Clp protease, ATP-binding subunit ClpX	Protein fate	Degradation of proteins, peptides, and glycopeptides	2.47	3.01	2.53	2.57
WAN014H9V_at	8.7	11.4	22.7	19.7	16.2	SA1667	SA1440	SAR1691	SAS1548	SAV1612	MW1562		peptidase, U32 family	Protein fate	Degradation of proteins, peptides, and glycopeptides	1.30	2.60	2.26	1.86
WAN014H9W_at	4.0	3.7	9.9	7.0	6.7	SA1668	SA1441	SAR1692	SAS1549	SAV1613	MW1563	pepU32	peptidase, U32 family	Protein fate	Degradation of proteins, peptides, and glycopeptides	-1.08	2.51	1.78	1.68
WAN014I3W_at	4.3	11.0	9.4	9.5	11.9	SA1281	SA1105	SAR1238	SAS1196	SAV1262	MW1145		membrane-associated zinc metalloprotease, putative	Protein fate	Degradation of proteins, peptides, and glycopeptides	2.58	2.21	2.24	2.79
WAN014FTG_at	1.4	1.6	3.0	2.7	2.6	SA1373	SA1173	SAR1348	SAS1278	SAV1338	MW1225		SCP-like extracelluar protein	Protein fate	Degradation of proteins, peptides, and glycopeptides	1.22	2.19	1.98	1.90
WAN014HPM_at	12.2	14.0	25.8	25.7	26.5	SA1036	SA0885	SAR0999	SAS0962	SAV1030	MW0910		protease, putative	Protein fate	Degradation of proteins, peptides, and glycopeptides	1.15	2.11	2.10	2.17
WAN014HKZ_at	10.4	22.3	41.0	28.8	26.5	SA0968	SA0825	SAR0926	SAS0834	SAV0964	MW0846	spsA	signal peptidase I	Protein fate	Protein and peptide secretion and trafficking	2.14	3.93	2.76	2.54
WAN014HL2_at	18.6	41.3	60.0	51.6	47.1	SA0969	SA0826	SAR0927	SAS0835	SAV0965	MW0847	spsB	signal peptidase I	Protein fate	Protein and peptide secretion and trafficking	2.22	3.22	2.77	2.52
WAN014I5Z_at	68.5	118.2	214.5	215.3	284.4	SA2219	SA2028	SAR2315	SAS2121	SAV2230	MW2149	secY	preprotein translocase, SecY subunit	Protein fate	Protein and peptide secretion and trafficking	1.73	3.13	3.14	4.15
WAN014HD2_at	6.1	15.1	18.3	14.4	17.7	SA1722	SA1499	SAR1755	SAS1604	SAV1675	MW1619	tig	trigger factor	Protein fate	Protein and peptide secretion and trafficking	2.49	3.01	2.37	2.91
WAN014IVR_at	3.7	8.2	9.3	10.9	10.2	SA0417	SA0334					tatC	MttB family protein	Protein fate	Protein and peptide secretion and trafficking	2.18	2.47	2.92	2.73
WAN014HBB_at	28.1	60.3	56.9	49.4	54.9	SA1692	SA1463	SAR1717	SAS1573	SAV1637	MW1587		protein-export membrane protein, SecDF family	Protein fate	Protein and peptide secretion and trafficking	2.14	2.02	1.75	1.95
WAN014I0L_at	7.7	12.6	47.5	35.3	29.1	SA2109	SA1919	SAR2205	SAS2020	SAV2117	MW2041		modification methylase, HemK family	Protein fate	Protein modification and repair	1.63	6.14	4.56	3.76
WAN014GU3_at	13.8	26.6	36.2	36.5	27.8	SA1100	SA0942	SAR1065	SAS1026	SAV1091	MW0974	def	polypeptide deformylase	Protein fate	Protein modification and repair	1.93	2.63	2.65	2.02
WAN014H4J_at	15.7	46.1	33.2	23.9	46.5	SA0635	SA0546	SAR0595	SAS0548	SAV0589	MW0544		lipoate-protein ligase A	Protein fate	Protein modification and repair	2.94	2.12	1.53	2.97
WAN014HEA_at	20.5	13.3	50.3	28.7	20.7	SA0847	SA0736	SAR0837	SAS0747	SAV0782	MW0743	smpB	SsrA-binding protein	Protein synthesis	Other	-1.55	2.45	1.40	1.01
WAN014HDC_at	9.6	16.2	180.1	153.2	135.3	SA1726	SA1503	SAR1759	SAS1608	SAV1679	MW1623	rpmI	ribosomal protein L35	Protein synthesis	Ribosomal proteins: synthesis and modification	1.68	18.74	15.94	14.08
WAN014HDA_at	6.8	10.8	112.9	89.6	73.8	SA1725	SA1502	SAR1758	SAS1607	SAV1678	MW1622	rplT	ribosomal protein L20	Protein synthesis	Ribosomal proteins: synthesis and modification	1.59	16.67	13.24	10.90
WAN014I3I_at	17.2	34.5	244.6	216.6	269.7	SA1274	SA1099	SAR1232	SAS1190	SAV1256	MW1139	rpsB	ribosomal protein S2	Protein synthesis	Ribosomal proteins: synthesis and modification	2.01	14.23	12.60	15.69
WAN014FYL_at	9.1	29.4	129.0	122.9	148.0	SA1769	SAS052	SAR1797	SAS1646	SAV1719	MW1662	rpsD	ribosomal protein S4	Protein synthesis	Ribosomal proteins: synthesis and modification	3.22	14.12	13.46	16.20
WAN014H2O_at	4.8	9.9	58.9	62.5	43.3	SA0590	SA0502	SAR0549	SAS0502	SAV0544	MW0499	L7A	30S ribosomal protein L7 Ae	Protein synthesis	Ribosomal proteins: synthesis and modification	2.06	12.29	13.05	9.03
WAN014FRF_at	9.6	14.2	108.7	105.8	130.1	SA2240	SA2048	SAR2336	SAS2142	SAV2251	MW2170	rpsJ	ribosomal protein S10	Protein synthesis	Ribosomal proteins: synthesis and modification	1.48	11.36	11.06	13.61
WAN014H2D_at	17.4	64.3	197.2	176.0	253.4	SA0585	SA0497	SAR0544	SAS0497	SAV0539	MW0494	rplJ	ribosomal protein L10	Protein synthesis	Ribosomal proteins: synthesis and modification	3.69	11.31	10.09	14.53
WAN014FRC_at	17.1	42.0	186.8	181.9	249.6	SA2238	SA2046	SAR2334	SAS2140	SAV2249	MW2168	rplD	ribosomal protein L4/L1 family	Protein synthesis	Ribosomal proteins: synthesis and modification	2.46	10.94	10.65	14.61
WAN014I6S_at	17.5	42.9	173.3	168.2	244.1	SA2232	SA2040	SAR2328	SAS2134	SAV2243	MW2162	rplP	ribosomal protein L16	Protein synthesis	Ribosomal proteins: synthesis and modification	2.45	9.92	9.62	13.97
WAN014FRA_at	4.4	11.8	42.4	42.5	51.2	SA2237	SA2045	SAR2333	SAS2139	SAV2248	MW2167	rplW	ribosomal protein L23	Protein synthesis	Ribosomal proteins: synthesis and modification	2.70	9.73	9.75	11.75
WAN014GVF_at	49.8	74.1	465.7	455.0	476.0	SA0439	SA0354	SAR0364	SAS0343	SAV0367	MW0343	rpsR	ribosomal protein S18	Protein synthesis	Ribosomal proteins: synthesis and modification	1.49	9.35	9.14	9.56
WAN014I6O_at	3.0	7.0	27.6	20.4	25.8	SA2231	SA2039	SAR2327	SAS2133	SAV2242	MW2161	rpmC	ribosomal protein L29	Protein synthesis	Ribosomal proteins: synthesis and modification	2.35	9.23	6.80	8.62
WAN014HBV_at	8.6	17.7	79.1	79.6	95.8	SA1702	SA1473	SAR1727	SAS1583	SAV1647	MW1597	rplU	ribosomal protein L21	Protein synthesis	Ribosomal proteins: synthesis and modification	2.07	9.23	9.28	11.18
WAN014I6T_at	14.9	34.3	136.5	119.2	155.2	SA2233	SA2041	SAR2329	SAS2135	SAV2244	MW2163	rpsC	ribosomal protein S3	Protein synthesis	Ribosomal proteins: synthesis and modification	2.30	9.18	8.01	10.44
WAN014I6K_at	19.2	41.0	175.7	143.6	206.2	SA2229	SA2037	SAR2325	SAS2131	SAV2240	MW2159	rplN	ribosomal protein L14	Protein synthesis	Ribosomal proteins: synthesis and modification	2.13	9.13	7.46	10.72
WAN014I6Y_at	11.1	23.1	99.9	103.1	124.7	SA2235	SA2043	SAR2331	SAS2137	SAV2246	MW2165	rpsS	ribosomal protein S19	Protein synthesis	Ribosomal proteins: synthesis and modification	2.08	9.01	9.30	11.25
WAN014I6D_at	15.9	39.1	141.2	134.2	180.6	SA2226	SAS079	SAR2322	SAS2128	SAV2237	MW2156	rpsN	ribosomal protein S14	Protein synthesis	Ribosomal proteins: synthesis and modification	2.46	8.88	8.45	11.36
WAN014I70_at	18.3	58.8	159.2	133.6	192.2	SA2236	SA2044	SAR2332	SAS2138	SAV2247	MW2166	rplB	ribosomal protein L2	Protein synthesis	Ribosomal proteins: synthesis and modification	3.21	8.68	7.28	10.48
WAN014I6B_at	3.5	9.5	29.6	23.3	29.2	SA2225	SA2034	SAR2321	SAS2127	SAV2236	MW2155	rpsH	ribosomal protein S8	Protein synthesis	Ribosomal proteins: synthesis and modification	2.75	8.53	6.72	8.41
WAN014H2F_at	7.1	21.9	58.4	49.0	69.1	SA0586	SA0498	SAR0545	SAS0498	SAV0540	MW0495	rplL	ribosomal protein L7/L12	Protein synthesis	Ribosomal proteins: synthesis and modification	3.10	8.27	6.94	9.78
WAN014I6W_at	7.4	17.3	60.3	50.1	60.6	SA2234	SA2042	SAR2330	SAS2136	SAV2245	MW2164	rplV	ribosomal protein L22	Protein synthesis	Ribosomal proteins: synthesis and modification	2.32	8.11	6.74	8.15
WAN014I5B_at	35.8	84.0	287.4	282.0	303.7	SA2207	SA2017	SAR2301	SAS2109	SAV2218	MW2137	rplM	ribosomal protein L13	Protein synthesis	Ribosomal proteins: synthesis and modification	2.35	8.03	7.88	8.49
WAN014FRD_at	11.2	16.3	88.5	84.1	97.1	SA2239	SA2047	SAR2335	SAS2141	SAV2250	MW2169	rplC	ribosomal protein L3	Protein synthesis	Ribosomal proteins: synthesis and modification	1.45	7.87	7.48	8.64
WAN014H2S_at	23.9	53.2	180.9	164.0	158.7	SA0592	SA0504	SAR0551	SAS0504	SAV0546	MW0501	rpsG	ribosomal protein S7	Protein synthesis	Ribosomal proteins: synthesis and modification	2.22	7.56	6.85	6.63
WAN014I6G_at	9.6	21.6	70.8	68.1	89.3	SA2227	SA2035	SAR2323	SAS2129	SAV2238	MW2157	rplE	ribosomal protein L5	Protein synthesis	Ribosomal proteins: synthesis and modification	2.24	7.34	7.07	9.27
WAN014I2E_at	23.4	63.3	168.9	142.5	80.8	SA1254	SA1081	SAR1214	SAS1172	SAV1238	MW1121	rpsP	ribosomal protein S16	Protein synthesis	Ribosomal proteins: synthesis and modification	2.71	7.22	6.09	3.45
WAN014I6I_at	3.7	6.7	26.6	22.7	25.8	SA2228	SA2036	SAR2324	SAS2130	SAV2239	MW2158	rplX	ribosomal protein L24	Protein synthesis	Ribosomal proteins: synthesis and modification	1.80	7.12	6.08	6.91
WAN014I67_at	3.6	9.4	25.2	20.0	27.7	SA2223	SA2032	SAR2319	SAS2125	SAV2234	MW2153	rplR	ribosomal protein L18	Protein synthesis	Ribosomal proteins: synthesis and modification	2.62	7.03	5.60	7.74
WAN014H2Q_at	20.4	31.1	142.9	138.5	161.5	SA0591	SA0503	SAR0550	SAS0503	SAV0545	MW0500	rpsL	ribosomal protein S12	Protein synthesis	Ribosomal proteins: synthesis and modification	1.52	7.00	6.78	7.91
WAN014I69_at	18.5	53.4	128.4	116.3	176.2	SA2224	SA2033	SAR2320	SAS2126	SAV2235	MW2154	rplF	ribosomal protein L6	Protein synthesis	Ribosomal proteins: synthesis and modification	2.89	6.96	6.30	9.55
WAN014I58_at	50.0	98.4	330.7	300.4	323.8	SA2206	SA2016	SAR2300	SAS2108	SAV2217	MW2136	rpsI	ribosomal protein S9	Protein synthesis	Ribosomal proteins: synthesis and modification	1.97	6.61	6.00	6.47
WAN014I65_at	25.4	71.1	152.2	133.8	199.7	SA2222	SA2031	SAR2318	SAS2124	SAV2233	MW2152	rpsE	ribosomal protein S5	Protein synthesis	Ribosomal proteins: synthesis and modification	2.80	5.98	5.26	7.85
WAN014GES_at	2.1	3.2	12.6	12.3	11.1	SA2740	SAS093	SAR2800	SAS2596	SAV2714	MW2632	rpmH	ribosomal protein L34	Protein synthesis	Ribosomal proteins: synthesis and modification	1.51	5.85	5.74	5.15
WAN014HVK_at	14.8	29.5	85.1	92.0	125.8	SA1137	SAS033	SAR1101	SAS1062	SAV1128	MW1010	rpmF	ribosomal protein L32	Protein synthesis	Ribosomal proteins: synthesis and modification	1.99	5.74	6.21	8.49
WAN014I0S_at	70.3	228.4	384.6	425.6	430.8	SA2112	SA1922	SAR2208	SAS2023	SAV2120	MW2044	rpmE	ribosomal protein L31	Protein synthesis	Ribosomal proteins: synthesis and modification	3.25	5.47	6.05	6.12
WAN014I63_at	47.7	137.4	253.3	239.4	389.8	SA2221	SA2030	SAR2317	SAS2123	SAV2232	MW2151	rpmD	ribosomal protein L30p/L7e	Protein synthesis	Ribosomal proteins: synthesis and modification	2.88	5.32	5.02	8.18
WAN014I2L_at	66.9	186.6	355.6	340.8	409.8	SA1257	SA1084	SAR1217	SAS1175	SAV1241	MW1124	rplS	ribosomal protein L19	Protein synthesis	Ribosomal proteins: synthesis and modification	2.79	5.32	5.09	6.13
WAN014H2C_at	46.4	76.9	232.6	238.1	311.0	SA0584	SA0496	SAR0543	SAS0496	SAV0538	MW0493	rplA	ribosomal protein L1	Protein synthesis	Ribosomal proteins: synthesis and modification	1.66	5.01	5.13	6.70
WAN014FT9_at	1.4	2.4	6.5	5.2	6.5	SA1370	SA1171	SAR1346	SAS1276	SAV1336	MW1223	rpsN	ribosomal protein S14	Protein synthesis	Ribosomal proteins: synthesis and modification	1.76	4.70	3.79	4.73
WAN014I6L_at	1.5	2.7	6.9	6.3	6.4	SA2230	SA2038	SAR2326	SAS2132	SAV2241	MW2160	rpsQ	ribosomal protein S17	Protein synthesis	Ribosomal proteins: synthesis and modification	1.84	4.67	4.27	4.35
WAN014I4I_at	23.2	20.1	90.8	92.3	114.2	SA1292	SA1116	SAR1249	SAS1207	SAV1273	MW1156	rpsO	ribosomal protein S15	Protein synthesis	Ribosomal proteins: synthesis and modification	-1.15	3.91	3.98	4.92
WAN014I61_at	15.7	38.8	61.2	53.5	75.7	SA2220	SA2029	SAR2316	SAS2122	SAV2231	MW2150	rplO	ribosomal protein L15	Protein synthesis	Ribosomal proteins: synthesis and modification	2.46	3.89	3.40	4.81

Systematic	Stat.	Log	30'	120'	360'	COL	N315	MRSA	MSSA	Mu50	MW2	GENE NAME	Protein Function	TIGR Main Role	TIGR Sub Role	Fold change vs stationary phase			
																LB	30' in vivo	120' in vivo	360' in vivo
WAN014H29_at	50.3	84.9	194.6	163.2	187.1	SA0583	SA0495	SAR0542	SAS0495	SAV0537	MW0492	rplK	ribosomal protein L11	Protein synthesis	Ribosomal proteins: synthesis and modification	1.69	3.86	3.24	3.72
WAN014I5K_at	24.6	57.3	90.0	66.2	93.0	SA2212	SA2022	SAR2308	SAS2114	SAV2223	MW2142	rplQ	ribosomal protein L17	Protein synthesis	Ribosomal proteins: synthesis and modification	2.34	3.66	2.70	3.79
WAN014HBQ_at	167.6	269.6	560.3	642.9	904.4	SA1700	SA1471	SAR1725	SAS1581	SAV1645	MW1595	rpmA	ribosomal protein L27	Protein synthesis	Ribosomal proteins: synthesis and modification	1.61	3.34	3.84	5.40
WAN014I5Q_at	17.3	36.2	52.7	45.1	63.1	SA2215	SA2025	SAR2311	SAS2117	SAV2226	MW2145	rpmJ	ribosomal protein L36	Protein synthesis	Ribosomal proteins: synthesis and modification	2.10	3.05	2.61	3.65
WAN014I5O_at	69.5	147.6	210.4	173.3	285.9	SA2214	SA2024	SAR2310	SAS2116	SAV2225	MW2144	rpsK	ribosomal protein S11	Protein synthesis	Ribosomal proteins: synthesis and modification	2.12	3.03	2.49	4.11
WAN014FXU_at	4.3	11.2	12.5	11.1	14.6	SA1642	SA1414	SAR1663	SAS1523	SAV1586	MW1537	rpsT	ribosomal protein S20	Protein synthesis	Ribosomal proteins: synthesis and modification	2.59	2.89	2.57	3.37
WAN014H6M_at	3.9	8.8	10.5	11.1	13.2	SA1608	SAS047	SAR1628	SAS1489	SAV1551	MW1503	rpmG	ribosomal protein L33	Protein synthesis	Ribosomal proteins: synthesis and modification	2.25	2.67	2.84	3.36
WAN014GOM_at	1.6	4.6	9.3	6.6	5.8	SA0789	SA0683	SAR0782	SAS0693	SAV0728	MW0690		7-aminomethyl-7-carbaguanine:NADP+ oxidoreductase	Protein synthesis	RNA and tRNA modification	2.97	5.94	4.19	3.73
WAN014HDE_at	5.6	6.9	77.4	71.0	79.0	SA1727	SA1504	SAR1760	SAS1609	SAV1680	MW1624	infC	translation initiation factor IF-3	Protein synthesis	Translation factors	1.21	13.71	12.58	14.00
WAN014HUD_at	5.7	10.3	75.4	66.8	69.1	SA1118	SA0959	SAR1083	SAS1044	SAV1109	MW0992	typA	elongation factor, Tu family	Protein synthesis	Translation factors	1.80	13.14	11.63	12.03
WAN014I5V_at	18.2	44.4	78.6	63.9	80.8	SA2217	SA2026	SAR2313	SAS2119	SAV2228	MW2147	infA	translation initiation factor IF-1	Protein synthesis	Translation factors	2.44	4.32	3.51	4.44
WAN014H2U_at	80.7	173.0	285.0	285.4	383.4	SA0593	SA0505	SAR0552	SAS0505	SAV0547	MW0502	fusA	translation elongation factor G	Protein synthesis	Translation factors	2.15	3.53	3.54	4.75
WAN014I0N_at	5.5	8.1	16.4	13.0	10.7	SA2110	SA1920	SAR2206	SAS2021	SAV2118	MW2042	prfA	peptide chain release factor 1	Protein synthesis	Translation factors	1.47	2.97	2.35	1.93
WAN014I4B_at	38.1	28.5	82.9	64.2	72.6	SA1288	SA1112	SAR1245	SAS1203	SAV1269	MW1152	infB	translation initiation factor IF-2	Protein synthesis	Translation factors	-1.34	2.18	1.69	1.91
WAN014HAX_at	1.1	4.4	11.4	9.7	8.9	SA1686	SA1457	SAR1711	SAS1567	SAV1631	MW1581	hisS	histidyl-tRNA synthetase	Protein synthesis	tRNA aminoacylation	3.96	10.33	8.84	8.11
WAN014HCD_at	7.4	16.7	50.5	36.5	25.7	SA1710	SA1488	SAR1743	SAS1592	SAV1663	MW1607	valS	valyl-tRNA synthetase	Protein synthesis	tRNA aminoacylation	2.26	6.82	4.93	3.47
WAN014HI0_at	3.9	20.3	19.5	13.5	11.8	SA1808	SA1579	SAR1843	SAS1684	SAV1760	MW1701	leuS	leucyl-tRNA synthetase	Protein synthesis	tRNA aminoacylation	5.15	4.94	3.42	2.99
WAN01BZZL_at	10.5	58.5	42.8	36.2	37.8			SAR1169	SAS1127	SAV1193	MW1076	ileS	isoleucyl-tRNA synthetase	Protein synthesis	tRNA aminoacylation	5.57	4.07	3.45	3.60
WAN014HZU_s_at	7.7	40.0	24.4	21.1	24.6	SA1206	SA1036	SAR1169	SAS1127	SAV1193	MW1076	ileS	isoleucyl-tRNA synthetase	Protein synthesis	tRNA aminoacylation	5.21	3.18	2.76	3.20
WAN014I3Y_at	4.8	12.2	11.1	9.9	10.6	SA1282	SA1106	SAR1239	SAS1197	SAV1263	MW1146	proS	prolyl-tRNA synthetase	Protein synthesis	tRNA aminoacylation	2.54	2.32	2.06	2.21
WAN014HFN_at	1.6	3.9	3.3	3.5	3.3	SA1778	SA1550	SAR1806	SAS1655	SAV1729	MW1671	tyrS	tyrosyl-tRNA synthetase	Protein synthesis	tRNA aminoacylation	2.41	2.06	2.14	2.04
WAN014GJG_at	7.7	12.0	101.8	99.8	94.5		#N/A					tRNA-Met	tRNA-Met	Protein synthesis	tRNA and rRNA base modification	1.55	13.17	12.91	12.22
WAN014IR4_at	52.5	64.6	585.5	617.8	479.4		tRNA12					tRNA-Pro	tRNA-Pro	Protein synthesis	tRNA and rRNA base modification	1.23	11.15	11.77	9.13
WAN014GJC_at	18.4	26.2	199.1	191.1	173.9		#N/A					tRNA-Cys	tRNA-Cys	Protein synthesis	tRNA and rRNA base modification	1.43	10.85	10.41	9.48
WAN014IR2_at	12.2	26.0	116.4	128.5	122.3		tRNA07					tRNA-Thr	tRNA-Thr	Protein synthesis	tRNA and rRNA base modification	2.13	9.55	10.54	10.04
WAN014H2H_at	5.0	4.3	43.9	33.8	21.5	SA0587	SA0499	SAR0546	SAS0499	SAV0541	MW0496		Methyltransferase small domain	Protein synthesis	tRNA and rRNA base modification	-1.15	8.78	6.76	4.30
WAN014IR6_at	35.8	103.4	245.2	257.6	229.5		tRNA57					tRNA-Lys	tRNA-Lys	Protein synthesis	tRNA and rRNA base modification	2.89	6.86	7.20	6.42
WAN014I2I_at	1.8	2.7	11.7	8.8	8.3	SA1256	SA1083	SAR1216	SAS1174	SAV1240	MW1123	trmD	tRNA (guanine-N1)-methyltransferase	Protein synthesis	tRNA and rRNA base modification	1.52	6.52	4.94	4.67
WAN014ILT_x_at	60.9	80.3	383.4	409.7	443.7		#N/A					tRNA-Leu	tRNA-Leu	Protein synthesis	tRNA and rRNA base modification	1.32	6.30	6.73	7.29
WAN014H02_at	1.9	2.6	11.1	7.9	8.0	SA1483	SA1277	SAR1456	SAS1387	SAV1444	MW1333		Putative RNA methylase family UPF0020	Protein synthesis	tRNA and rRNA base modification	1.41	5.91	4.18	4.23
WAN01C9HD_x_at	57.2	78.0	333.2	361.2	456.0		SAtRNA					tRNA-Leu	tRNA-Leu	Protein synthesis	tRNA and rRNA base modification	1.36	5.82	6.31	7.97
WAN014IR5_at	88.7	154.5	430.1	531.0	609.1		tRNA11					tRNA-Arg	tRNA-Arg	Protein synthesis	tRNA and rRNA base modification	1.74	4.85	5.99	6.87
WAN014IR9_at	125.2	144.8	559.9	607.7	600.3		tRNA14					tRNA-Ile	tRNA-Ile	Protein synthesis	tRNA and rRNA base modification	1.16	4.47	4.85	4.79
WAN014GJJ_at	22.3	79.1	96.7	108.7	114.0		#N/A					tRNA-Leu	tRNA-Leu	Protein synthesis	tRNA and rRNA base modification	3.54	4.33	4.87	5.10
WAN014I34_at	8.1	19.8	31.2	30.5	37.3	SA1268	SA1094	SAR1227	SAS1185	SAV1251	MW1134	trmFO	tRNA:m(5)U-54 methyltransferase	Protein synthesis	tRNA and rRNA base modification	2.45	3.86	3.76	4.60
WAN014HBF_at	7.8	8.7	29.4	27.9	29.0	SA1694	SA1465	SAR1719	SAS1575	SAV1639	MW1589	tgt	queuine tRNA-ribosyltransferase	Protein synthesis	tRNA and rRNA base modification	1.11	3.77	3.58	3.72
WAN014GJD_at	144.6	97.1	513.9	546.9	451.8		#N/A					tRNA-Gln	tRNA-Gln	Protein synthesis	tRNA and rRNA base modification	-1.49	3.55	3.78	3.12
WAN014I00_at	5.4	7.3	18.2	13.5	15.4	SA1209	SA1040	SAR1173	SAS1131	SAV1197	MW1080		ribosomal large subunit pseudouridine synthases, RluD subfamily	Protein synthesis	tRNA and rRNA base modification	1.36	3.38	2.51	2.87
WAN014GJ8_at	60.6	36.2	190.3	195.2	183.6		#N/A					tRNA-Phe	tRNA-Phe	Protein synthesis	tRNA and rRNA base modification	-1.67	3.14	3.22	3.03
WAN014GJ7_at	158.7	156.0	449.7	472.7	553.3		#N/A					tRNA-His	tRNA-His	Protein synthesis	tRNA and rRNA base modification	-1.02	2.83	2.98	3.49
WAN014GJF_at	103.3	91.5	291.7	275.0	264.7		#N/A					tRNA-Tyr	tRNA-Tyr	Protein synthesis	tRNA and rRNA base modification	-1.13	2.82	2.66	2.56
WAN014GJH_at	171.1	108.0	476.0	488.3	619.0		#N/A					tRNA-Met	tRNA-Met	Protein synthesis	tRNA and rRNA base modification	-1.58	2.78	2.85	3.62
WAN014HBG_at	3.5	3.8	8.2	7.0	6.9	SA1695	SA1466	SAR1720	SAS1576	SAV1640	MW1590	queA	S-adenosylmethionine:tRNA ribosyltransferase-isomerase	Protein synthesis	tRNA and rRNA base modification	1.11	2.37	2.02	2.01
WAN014HBO_at	14.9	17.5	32.0	33.8	31.7	SA1699	SA1470	SAR1724	SAS1580	SAV1644	MW1594	ctgA	GTP-binding protein, Obg family GTPase CgtA	Protein synthesis	tRNA and rRNA base modification	1.17	2.14	2.27	2.12
WAN014G11_at	6.7	7.7	14.2	14.1	7.8	SA1907	SA1668	SAR1941	SAS1771	SAV1850	MW1791		ribosomal large subunit pseudouridine synthases, RluD subfamily	Protein synthesis	tRNA and rRNA base modification	1.16	2.13	2.11	1.17
WAN014GJ2_at	3.5	2.3	7.2	7.9	9.8		#N/A					tRNA-Arg	tRNA-Arg	Protein synthesis	tRNA and rRNA base modification	-1.50	2.07	2.28	2.83
WAN014HH0_at	7.2	10.3	14.8	11.3	8.9	SA1798	SA1569	SAR1833	SAS1674	SAV1748	MW1691		tRNA (guanine-N(7)-)-methyltransferase	Protein synthesis	tRNA and rRNA base modification	1.44	2.07	1.57	1.24
WAN014GJK_at	86.9	55.9	178.9	167.6	192.0		#N/A					tRNA-Leu	tRNA-Leu	Protein synthesis	tRNA and rRNA base modification	-1.55	2.06	1.93	2.21
WAN014FZA_at	3.2	5.3	6.5	5.6	4.2	SA1811	SA1582	SAR1846	SAS1687	SAV1763	MW1704	mraW	SAM-dependent methyltransferase, MraW methylase family	Protein synthesis	tRNA and rRNA base modification	1.68	2.04	1.76	1.34
WAN014FTB_at	1.1	1.2	11.9	13.4	11.9	SA1371	SA1172	SAR1347	SAS1277	SAV1337	MW1224	guaC	GMP reductase	Purines, pyrimidines, nucleosides, and nucleotides	Nucleotide and nucleoside interconversions	1.08	10.67	11.99	10.61
WAN014I5X_at	29.4	52.8	119.4	100.1	149.0	SA2218	SA2027	SAR2314	SAS2120	SAV2229	MW2148	adk	adenylate kinase	Purines, pyrimidines, nucleosides, and nucleotides	Nucleotide and nucleoside interconversions	1.80	4.06	3.41	5.07
WAN014I0P_at	6.1	7.9	16.1	13.6	11.8	SA2111	SA1921	SAR2207	SAS2022	SAV2119	MW2043	tdk	thymidine kinase	Purines, pyrimidines, nucleosides, and nucleotides	Nucleotide and nucleoside interconversions	1.29	2.64	2.23	1.94
WAN014GJT_at	4.4	5.5	173.6	138.3	124.5	SA0018	SA0016	SAR0017	SAS0017	SAV0017	MW0017	purA	adenylosuccinate synthetase	Purines, pyrimidines, nucleosides, and nucleotides	Purine ribonucleotide biosynthesis	1.26	39.70	31.64	28.47
WAN014HS6_at	5.9	11.9	37.5	27.1	21.4	SA1083	SA0926	SAR1048	SAS1010	SAV1074	MW0957	purD	phosphoribosylamine--glycine ligase	Purines, pyrimidines, nucleosides, and nucleotides	Purine ribonucleotide biosynthesis	2.00	6.31	4.57	3.60
WAN014HRY_at	1.5	3.6	6.9	5.6	5.1	SA1080	SA0923	SAR1045	SAS1007	SAV1071	MW0954	purM	phosphoribosylformylglycinamidine cyclo-ligase	Purines, pyrimidines, nucleosides, and nucleotides	Purine ribonucleotide biosynthesis	2.32	4.49	3.67	3.33
WAN014HRT_at	3.3	7.5	12.8	11.8	11.3	SA1078	SA0921	SAR1043	SAS1005	SAV1069	MW0952	purL	phosphoribosylformylglycinamidine synthase II	Purines, pyrimidines, nucleosides, and nucleotides	Purine ribonucleotide biosynthesis	2.30	3.94	3.62	3.46
WAN014HRV_at	2.4	5.1	8.3	6.9	5.5	SA1079	SA0922	SAR1044	SAS1006	SAV1070	MW0953	purF	amidophosphoribosyltransferase	Purines, pyrimidines, nucleosides, and nucleotides	Purine ribonucleotide biosynthesis	2.17	3.51	2.93	2.31
WAN014HS2_at	2.0	4.2	6.2	5.0	4.9	SA1082	SA0925	SAR1047	SAS1009	SAV1073	MW0956	purH	phosphoribosylaminoimidazolecarboxamide formyltransferase/IMP cyclohydrolase	Purines, pyrimidines, nucleosides, and nucleotides	Purine ribonucleotide biosynthesis	2.12	3.11	2.53	2.48
WAN014HS0_at	2.2	3.3	6.7	5.2	5.5	SA1081	SA0924	SAR1046	SAS1008	SAV1072	MW0955	purN	phosphoribosylglycinamide formyltransferase	Purines, pyrimidines, nucleosides, and nucleotides	Purine ribonucleotide biosynthesis	1.47	2.99	2.34	2.44
WAN014HRK_at	1.0	2.3	2.6	2.1	1.7	SA1076	SA0919	SAR1041	SAS1003	SAV1067	MW0950	purS	phosphoribosylformylglycinamidine synthase, PurS protein	Purines, pyrimidines, nucleosides, and nucleotides	Purine ribonucleotide biosynthesis	2.26	2.57	2.00	1.68
WAN014HRD_at	1.5	2.0	3.3	2.9	3.3	SA1073	SA0916	SAR1038	SAS1000	SAV1064	MW0947	purE	phosphoribosylaminoimidazole carboxylase, catalytic subunit	Purines, pyrimidines, nucleosides, and nucleotides	Purine ribonucleotide biosynthesis	1.35	2.21	1.95	2.18
WAN014HRN_at	1.5	2.9	3.3	3.3	3.6	SA1077	SA0920	SAR1042	SAS1004	SAV1068	MW0951	purQ	phosphoribosylformylglycinamidine synthetase I	Purines, pyrimidines, nucleosides, and nucleotides	Purine ribonucleotide biosynthesis	1.92	2.16	2.15	2.35
WAN014I18_at	1.6	4.8	28.6	23.3	19.9	SA2119	SA1929	SAR2215	SAS2030	SAV2127	MW2051	pyrG	CTP synthase	Purines, pyrimidines, nucleosides, and nucleotides	Pyrimidine ribonucleotide biosynthesis	2.97	17.72	14.42	12.33
WAN014I07_at	1.4	1.1	9.2	5.3	3.4	SA1213	SA1044	SAR1177	SAS1135	SAV1201	MW1084	pyrC	dihydroorotase	Purines, pyrimidines, nucleosides, and nucleotides	Pyrimidine ribonucleotide biosynthesis	-1.25	6.76	3.85	2.50
WAN014I0B_at	2.3	2.6	13.0	7.2	8.1	SA1215	SA1046	SAR1179	SAS1137	SAV1203	MW1086	carB	carbamoyl-phosphate synthase, large subunit	Purines, pyrimidines, nucleosides, and nucleotides	Pyrimidine ribonucleotide biosynthesis	1.12	5.66	3.15	3.51
WAN014I09_at	1.4	1.2	7.3	4.2	4.0	SA1214	SA1045	SAR1178	SAS1136	SAV1202	MW1085	carA	carbamoyl-phosphate synthase, small subunit	Purines, pyrimidines, nucleosides, and nucleotides	Pyrimidine ribonucleotide biosynthesis	-1.16	5.29	3.01	2.90
WAN014I0G_at	1.3	1.3	3.5	1.9	3.0	SA1216	SA1047	SAR1180	SAS1138	SAV1204	MW1087	pyrF	orotidine 5'-phosphate decarboxylase	Purines, pyrimidines, nucleosides, and nucleotides	Pyrimidine ribonucleotide biosynthesis	1.01	2.63	1.43	2.24
WAN014HB5_at	5.5	10.3	16.9	13.5	15.6	SA1690	SA1461	SAR1715	SAS1571	SAV1635	MW1585	apt	adenine phosphoribosyltransferase	Purines, pyrimidines, nucleosides, and nucleotides	Salvage of nucleosides and nucleotides	1.87	3.06	2.44	2.83
WAN014H9T_at	15.5	21.7	41.5	29.9	30.5	SA1666	SA1439	SAR1690	SAS1547	SAV1611	MW1561	udk	uridine kinase	Purines, pyrimidines, nucleosides, and nucleotides	Salvage of nucleosides and nucleotides	1.40	2.67	1.92	1.96
WAN014HDP_at	18.6	35.3	130.8	108.2	73.4	SA0837	SA0726	SAR0827	SAS0737	SAV0771	MW0733	gapR	gap transcriptional regulator	Regulatory functions	DNA interactions	1.90	7.05	5.83	3.95
WAN014HO2_at	4.5	8.0	19.8	24.8	18.8	SA1904	SA1665	SAR1938	SAS1768	SAV1847	MW1788		transcriptional regulator, putative	Regulatory functions	DNA interactions	1.77	4.35	5.46	4.13
WAN014GXB_at	1.2	1.2	3.2	5.6	3.6	SA1420	SA1217	SAR1398	SAS1326	SAV1385	MW1273		phosphate transport system regulatory protein, putative	Regulatory functions	DNA interactions	-1.03	2.67	4.64	3.01
WAN014FZE_at	1.3	1.8	3.5	2.9	2.8	SA1822	SA1591	SAR1855	SAS1696	SAV1773	MW1713	arsR	arsenical resistance operon repressor	Regulatory functions	DNA interactions	1.38	2.64	2.14	2.11
WAN014H6S_at	1.0	1.5	2.4	3.3	2.4	SA1611	SA1383	SAR1631	SAS1492	SAV1554	MW1506	furA	transcriptional regulator, Fur family	Regulatory functions	DNA interactions	1.45	2.35	3.35	2.43
WAN014G7R_at	2.7	3.0	25.1	25.0	21.0	SA2585	SA2357	SAR2651	SAS2456	SAV2570	MW2491		regulatory protein, putative	Regulatory functions	Other	1.12	9.25	9.21	7.73
WAN014G5T_at	3.2	9.2	14.3	14.3	11.4	SA2546	SA2320	SAR2612	SAS2418	SAV2532	MW2453		regulatory protein, putative	Regulatory functions	Other	2.83	4.42	4.42	3.52
WAN014FV4_at	2.7	5.2	9.7	7.0	7.8	SA1398	SA1195	SAR1374	SAS1302	SAV1362	MW1249	msrR	transcriptional regulator, putative	Regulatory functions	Other	1.94	3.62	2.62	2.89
WAN014GAK_at	2.8	4.8	7.3	4.3	5.5	SA2645	SA2417	SAR2702	SAS2510	SAV2624	MW2544		sensor histidine kinase	Regulatory functions	Protein interactions	1.70	2.60	1.54	1.94
WAN014I0H_at	2.7	3.3	5.8	4.3	2.9	SA2107	SA1917	SAR2203	SAS2018	SAV2115	MW2039		phosphotyrosine protein phosphatase	Regulatory functions	Protein interactions	1.23	2.18	1.60	1.09
WAN014I1D_at	10.8	25.8	22.3	17.2	16.5	SA1232	SA1063	SAR1196	SAS1154	SAV1220	MW1103	pknB	protein kinase	Regulatory functions	Protein interactions	2.39	2.06	1.59	1.53
WAN014I02_at	5.0	6.1	15.9	12.6	7.0	SA1210	SA1041	SAR1174	SAS1132	SAV1198	MW1081	pyrR	pyrimidine operon regulatory protein	Regulatory functions	RNA interactions	1.21	3.15	2.51	1.40
WAN014GP6_at	7.0	5.3	23.3	20.5	20.7	SA0809	SA0701	SAR0800	SAS0711	SAV0746	MW0708		GGDEF family protein, putative	Regulatory functions	Small molecule interactions	-1.32	3.33	2.92	2.96
WAN014HE6_at	5.4	5.3	12.3	8.9	8.3	SA0846	SA0735	SAR0836	SAS0746	SAV0780	MW0742	vacB	ribonuclease R	Transcription	Degradation of RNA	-1.02	2.28	1.65	1.54
WAN014IQL_at	3.6	9.5	15.9	16.9	12.8	SA2120	SA1930	SAR2216	SAS2031	SAV2128	MW2052	rpoE	DNA-directed RNA polymerase, delta subunit	Transcription	DNA-dependent RNA polymerase	2.64	4.42	4.69	3.55
WAN014I5M_at	64.8	139.7	207.6	168.0	256.4	SA2213	SA2023	SAR2309	SAS2115	SAV2224	MW2143	rpoA	DNA-directed RNA polymerase, alpha subunit	Transcription	DNA-dependent RNA polymerase	2.16	3.20	2.59	3.96
WAN014HY3_at	6.4	12.4	294.2	252.9	205.9	SA2072	SA1885	SAR2168	SAS1985	SAV2081	MW2004		ATP-dependent RNA helicase, DEAD/DEAH box family	Transcription	Other	1.95	46.21	39.73	32.35
WAN014H6Y_at	2.0	3.1	5.0	3.9	3.4	SA1615	SA1387	SAR1635	SAS1496	SAV1558	MW1510		ATP-dependent RNA helicase, DEAD/DEAH box family	Transcription	Other	1.59	2.52	2.00	1.74
WAN014I2G_at	1.5	1.7	7.9	6.8	5.2	SA1255	SA1082	SAR1215	SAS1173	SAV1239	MW1122	rimM	16S rRNA processing protein RimM	Transcription	RNA processing	1.14	5.26	4.53	3.48
WAN014GEQ_at	1.7	1.3	5.0	4.2	2.9	SA2739	SA2502	SAR2799	SAS2595	SAV2713	MW2631	rnpA	ribonuclease P protein component	Transcription	RNA processing	-1.30	2.93	2.46	1.72
WAN014I0U_at	7.8	22.5	29.1	24.4	26.6	SA2113	SA1923	SAR2209	SAS2024	SAV2121	MW2045	rho	transcription termination factor Rho	Transcription	Transcription factors	2.89	3.74	3.14	3.42
WAN014GEJ_at	15.8	8.4	145.3	135.9	88.8	SA2619	SA2396	SAR2681	SAS2488	SAV2603	MW2522		amino acid permease	Transport and binding proteins	Amino acids, peptides and amines	-1.89	9.19	8.60	5.62
WAN014HOA_at	6.4	7.3	46.6	54.7	36.8	SA1018	SA0871	SAR0980	SAS0882	SAV1013	MW0894		sodium:alanine symporter family protein	Transport and binding proteins	Amino acids, peptides and amines	1.14	7.27	8.55	5.74
WAN014FUD_at	15.5	51.1	100.3	83.5	86.3	SA1384	SA1183	SAR1361	SAS1288	SAV1348	MW1236	opuD	osmoprotectant transporter, BCCT family	Transport and binding proteins	Amino acids, peptides and amines	3.29	6.47	5.39	5.56
WAN014H8D_at	8.1	14.3	49.1	45.0	45.8	SA0711	SA0609	SAR0664	SAS0619	SAV0654	MW0616		Branched-chain amino acid transport protein	Transport and binding proteins	Amino acids, peptides and amines	1.78	6.10	5.59	5.68
WAN014GXR_at	2.9	10.7	17.5	19.0	14.1	SA1443	SA1239	SAR1419	SAS1350	SAV1407	MW1297	brnQ	branched-chain amino acid transport system II carrier protein	Transport and binding proteins	Amino acids, peptides and amines	3.72	6.06	6.58	4.88
WAN014I0E_at	19.7	19.1	109.8	98.2	82.0	SA0302	SA0294	SAR0303	SAS0283	SAV0306	MW0283	brnQ	branched-chain amino acid transport system II carrier protein	Transport and binding proteins	Amino acids, peptides and amines	-1.03	5.57	4.98	4.16
WAN014H4F_at	34.3	7.9	133.8	72.6	70.4	SA0630	SA0541	SAR0589	SAS0542	SAV0584	MW0539		amino acid permease	Transport and binding proteins	Amino acids, peptides and amines	-4.32	3.90	2.12	2.05
WAN014G9J_at	3.0	3.0	9.1	5.6	4.8	SA2632	SA2408	SAR2693	SAS2501	SAV2615	MW2535	cudT	osmoprotectant transporter, BCCT family	Transport and binding proteins	Amino acids, peptides and amines	-1.02	3.02	1.85	1.57
WAN014HT7_at	4.0	6.9	11.0	12.6	8.4	SA0010	SA0010	SAR0010	SAS0010	SAV0010	MW0010	azlC	azlC protein	Transport and binding proteins	Amino acids, peptides and amines	1.71	2.73	3.13	2.08
WAN014G1Z_at	2.3	3.1	6.0	6.6	4.6	SA2473	SA2252	SAR2551	SAS2355	SAV2464	MW2388	opp-1D	peptide ABC transporter, ATP-binding protein	Transport and binding proteins	Amino acids, peptides and amines	1.35	2.63	2.87	2.02
WAN014G23_at	3.1	4.3	7.4	8.6	5.2	SA2474	SA2253	SAR2552	SAS2356	SAV2465	MW2389	opp-1C	peptide ABC transporter, permease protein	Transport and binding proteins	Amino acids, peptides and amines	1.39	2.40	2.78	1.68

																	Fold change vs stationary phase			
Systematic	Stat.	Log	30'	120'	360'	COL	N315	MRSA	MSSA	Mu50	MW2	GENE NAME	Protein Function	TIGR Main Role	TIGR Sub Role	LB	30' in vivo	120' in vivo	360' in vivo
WAN014G1X_at	2.4	3.9	5.6	6.5	5.8	SA2472	SA2251	SAR2550	SAS2354	SAV2463	MW2387	opp-1F	peptide ABC transporter, ATP-binding protein	Transport and binding proteins	Amino acids, peptides and amines	1.64	2.37	2.75	2.46
WAN014G29_at	4.5	3.7	9.9	9.3	8.7	SA2476	SA2255	SAR2554	SAS2358	SAV2467	MW2391	opp-1A	peptide ABC transporter, peptide-binding protein	Transport and binding proteins	Amino acids, peptides and amines	-1.22	2.20	2.06	1.92
WAN014G27_at	4.4	3.9	9.0	9.6	8.1	SA2475	SA2254	SAR2553	SAS2357	SAV2466	MW2390	opp-1B	peptide ABC transporter, permease protein, putative	Transport and binding proteins	Amino acids, peptides and amines	-1.11	2.06	2.19	1.85
WAN014FXO_at	2.8	3.9	12.9	7.2	7.2	SA2386	SA2176	SAR2476	SAS2278	SAV2388	MW2308	narK	nitrate transporter	Transport and binding proteins	Anions	1.39	4.67	2.62	2.60
WAN014H8U_at	32.7	52.1	77.0	75.0	72.3	SA0722	SA0619	SAR0674	SAS0629	SAV0664	MW0626		phosphate transporter family protein	Transport and binding proteins	Anions	1.60	2.36	2.30	2.21
WAN014G62_at	1.3	6.4	9.5	12.8	21.7	SA2552	SA2326	SAR2618	SAS2424	SAV2538	MW2459	ptsG	PTS system, glucose-specific component	Transport and binding proteins	Carbohydrates, organic alcohols, and acids	4.88	7.27	9.78	16.58
WAN01BQNJ_at	3.8	20.5	23.8	19.5	16.4	SA2401	SA2191	SAR2493	SAS2294	SAV2403	MW2325		formate/nitrite transporter	Transport and binding proteins	Carbohydrates, organic alcohols, and acids	5.35	6.22	5.08	4.29
WAN014FXK_at	15.6	61.3	57.5	43.3	49.1	SA2382	SA2172	SAR2472	SAS2274	SAV2384	MW2304	gltT	sodium:dicarboxylate symporter family	Transport and binding proteins	Carbohydrates, organic alcohols, and acids	3.93	3.69	2.78	3.15
WAN014H8A_at	12.2	23.7	28.2	34.4	52.6	SA0175	SA0183	SAR0190	SAS0164	SAV0189	MW0163	glcA	PTS system, glucose-specific component	Transport and binding proteins	Carbohydrates, organic alcohols, and acids	1.95	2.31	2.82	4.31
WAN014G6N_at	8.4	14.9	18.3	21.9	21.4	SA2246	SA2053	SAR2341	SAS2147	SAV2256	MW2175		sugar transporter, putative	Transport and binding proteins	Carbohydrates, organic alcohols, and acids	1.77	2.18	2.61	2.55
WAN014GXN_at	2.1	15.2	132.3	147.3	102.9	SA0501	SA0417	SAR0458	SAS0416	SAV0458	MW0413		sodium-dependent transporter, putative	Transport and binding proteins	Cations	7.39	64.39	71.71	50.10
WAN014HP1_at	10.4	17.5	86.9	72.2	91.1	SA1026	SA0878	SAR0991	SAS0890	SAV1022	MW0902		Integral membrane protein TerC family	Transport and binding proteins	Cations	1.69	8.38	6.96	8.78
WAN014FTC_at	5.2	7.6	22.3	17.2	17.4	SA2277	SA2079	SAR2368	SAS2174	SAV2284	MW2202		iron compound ABC transporter, substrate-binding protein	Transport and binding proteins	Cations	1.46	4.26	3.28	3.33
WAN014GTG_at	14.8	24.8	33.9	36.4	29.7	SA0088	SA0100	SAR0110	SAS0081	SAV0104	MW0080		Na/Pi cotransporter, putative	Transport and binding proteins	Cations	1.68	2.29	2.46	2.00
WAN014I31_at	2.7	4.0	21.9	25.8	23.7	SA2164	SA1976	SAR2265	SAS2075	SAV2174	MW2100		transporter gate domain protein	Transport and binding proteins	Cations and iron carrying compounds	1.48	8.04	9.48	8.68
WAN014HKJ_at	3.5	4.1	25.2	16.8	9.5	SA0946	SA0804	SAR0905	SAS0813	SAV0943	MW0825		Na+/H+ antiporter family protein	Transport and binding proteins	Cations and iron carrying compounds	1.16	7.18	4.78	2.70
WAN014G6M_at	1.4	1.4	3.7	4.0	2.1	SA2565	SA2338	SAR2631	SAS2437	SAV2551	MW2472	feoA	FeoA domain protein	Transport and binding proteins	Cations and iron carrying compounds	1.01	2.59	2.81	1.47
WAN014FRG_at	10.3	22.0	98.6	86.0	83.1	SA2242	SA2050	SAR2338	SAS2144	SAV2253	MW2172		xanthine/uracil permease family protein	Transport and binding proteins	Nucleosides, purines and pyrimidines	2.13	9.56	8.33	8.05
WAN014I04_at	1.0		7.6	3.9	2.2	SA1211	SA1042	SAR1175	SAS1133	SAV1199	MW1082	pyrP	uracil permease	Transport and binding proteins	Nucleosides, purines and pyrimidines		7.50	3.80	2.19
WAN014HPD_at	2.1	3.5	15.7	13.3	12.6	SA1924	SA1683	SAR1956	SAS1788	SAV1866	MW1806	msbA	toxin exporting ABC transporter, ATP-binding/permease protein	Transport and binding proteins	Other	1.62	7.35	6.23	5.89
WAN014HAM_at	4.5	1.7	17.8	11.3	5.3	SA0086	SA0099	SAR0109	SAS0080	SAV0103	MW0079		drug transporter, putative	Transport and binding proteins	Other	-2.61	3.99	2.54	1.19
WAN014G71_at	2.2	2.4	6.8	4.5	4.4	SA2257	SA2061	SAR2350	SAS2156	SAV2266	MW2184		drug transporter	Transport and binding proteins	Other	1.07	3.06	2.02	1.97
WAN014FZG_at	1.3	1.9	3.7	3.3	2.6	SA1823	SA1592					arsB	arsenical pump membrane protein	Transport and binding proteins	Other	1.51	2.86	2.57	2.01
WAN014HI4_at	4.3	4.4	11.3	10.2	11.2	SA1809	SA1580	SAR1844	SAS1685	SAV1761	MW1702		drug transporter, putative	Transport and binding proteins	Other	1.02	2.65	2.38	2.63
WAN014HUO_at	2.1	1.4	4.9	4.3	4.6	SA0261	SA0263	SAR0271	SAS0250	SAV0274	MW0250		drug transporter, putative	Transport and binding proteins	Other	-1.47	2.30	2.01	2.17
WAN014FVY_at	2.5	9.9	16.9	14.9	16.6	SA1427	SA1224	SAR1404	SAS1333	SAV1392	MW1280		ABC transporter, ATP-binding protein	Transport and binding proteins	Unknown substrate	3.89	6.63	5.86	6.51
WAN014FYI_at	2.4	1.9	15.9	13.7	13.5	SA2403	SA2194	SAR2496	SAS2297	SAV2406	MW2328	znuA	ABC transporter, substrate-binding protein	Transport and binding proteins	Unknown substrate	-1.27	6.60	5.67	5.59
WAN014H6T_at	2.3	3.5	14.1	12.4	10.1	SA1612	SA1384	SAR1632	SAS1493	SAV1555	MW1507	znuB	ABC3 transporter family protein	Transport and binding proteins	Unknown substrate	1.55	6.16	5.43	4.41
WAN014G9U_at	3.9	12.6	14.2	11.4	7.9	SA2375	SA2166	SAR2465	SAS2268	SAV2376	MW2298		transporter	Transport and binding proteins	Unknown substrate	3.21	3.63	2.90	2.01
WAN014H6V_at	1.3	1.3	3.9	3.6	2.8	SA1613	SA1385	SAR1633	SAS1494	SAV1556	MW1508	znuC	ABC transporter, ATP-binding protein	Transport and binding proteins	Unknown substrate	1.02	3.04	2.82	2.20
WAN014H7F_at	8.3	14.6	22.0	21.2	17.6	SA0695	SA0594	SAR0648	SAS0604	SAV0638	MW0600	tagG	ABC-2 type transporter	Transport and binding proteins	Unknown substrate	1.77	2.66	2.57	2.14
WAN014GXV_at	0.9	3.7	2.5	2.4	2.0	SA0505	SA0421	SAR0462	SAS0420	SAV0463	MW0417	metI	ABC transporter, permease protein	Transport and binding proteins	Unknown substrate	3.94	2.65	2.51	2.07
WAN014G1V_at	6.4	9.9	16.6	17.4	16.3	SA2471	SA2250	SAR2549	SAS2353	SAV2462	MW2386		transporter, putative	Transport and binding proteins	Unknown substrate	1.54	2.59	2.72	2.54
WAN014GY3_at	9.5	18.2	83.9	67.1	42.8					SAV0465			autolysin (N-acetylmuramoyl-L-alanine amidase	Unclassified	Role category not yet assigned	1.93	8.86	7.08	4.52
WAN014HVJ_at	0.6	0.5	3.6	4.6	4.9			SAR2149					putative exported protein	Unclassified	Role category not yet assigned	-1.11	6.01	7.83	8.22
WAN014G94_at	2.4	1.1	11.1	8.3	9.2	SA2620	SA2397	SAR2682	SAS2489	SAV2604	MW2523	gabT	aminotransferase, class-III	Unknown function	Enzymes of unknown specificity	-2.28	4.58	3.42	3.79
WAN014G7J_at	7.0	21.4	21.8	16.6	15.8	SA2582	SA2354	SAR2649	SAS2453	SAV2567	MW2488		acetyltransferase	Unknown function	Enzymes of unknown specificity	3.07	3.13	2.39	2.26
WAN014G28_at	2.0	3.1	6.1	6.4	5.9	SA1954	SA1710	SAR1985	SAS1816	SAV1894	MW1835		exonuclease	Unknown function	Enzymes of unknown specificity	1.51	3.02	3.17	2.92
WAN014GO1_at	5.2	4.6	13.9	11.6	8.2	SA0770	SA0665	SAR0763	SAS0675	SAV0710	MW0672		radical activating enzyme family protein	Unknown function	Enzymes of unknown specificity	-1.12	2.68	2.23	1.59
WAN014H1Y_at	2.5	4.8	6.3	4.9	5.6	SA1520	SA1311	SAR1488	SAS1420	SAV1480	MW1368		pyridine nucleotide-disulfide oxidoreductase	Unknown function	Enzymes of unknown specificity	1.94	2.55	1.99	2.25
WAN014HFA_at	0.6	0.7	1.5	1.5	1.2	SA1765	SA1538						aminotransferase, class V	Unknown function	Enzymes of unknown specificity	1.18	2.54	2.39	2.00
WAN014H9Y_at	1.9	1.7	4.2	4.1	2.9	SA1669	SA1442	SAR1693	SAS1550	SAV1614	MW1564		O-methyltransferase family protein	Unknown function	Enzymes of unknown specificity	-1.07	2.27	2.19	1.55
WAN014FR3_at	6.0	20.1	83.9	75.8	58.1	SA0007	SA0007	SAR0007	SAS0007	SAV0007	MW0007		YjeF-related protein, C-terminus	Unknown function	General	3.34	13.97	12.61	9.68
WAN014GOC_at	1.4	2.7	7.2	7.3	5.9	SA0772	SA0667	SAR0765	SAS0677	SAV0712	MW0674	exsB	exsB protein	Unknown function	General	1.99	5.23	5.28	4.27
WAN014HKL_at	4.0	4.1	15.9	12.4	8.2	SA0947	SA0805	SAR0906	SAS0814	SAV0944	MW0826	yuxO	comA operon protein 2	Unknown function	General	1.00	3.95	3.07	2.03
WAN014HBM_at	5.0	6.2	11.7	12.6	12.5	SA1698	SA1469	SAR1723	SAS1579	SAV1643	MW1593	pheB	ACT domain protein	Unknown function	General	1.25	2.37	2.54	2.53
WAN014HWS_at	2.3	4.9	5.3	3.6	3.6	SA2053	SA1868	SAR2151	SAS1968	SAV2063	MW1987		S1 RNA binding domain protein	Unknown function	General	2.12	2.31	1.55	1.58
WAN014I0J_at	4.8	6.9	10.2	8.1	6.1	SA2108	SA1918	SAR2204	SAS2019	SAV2116	MW2040		Sua5/YciO/YrdC/YwlC family protein	Unknown function	General	1.45	2.14	1.70	1.28
