# Supplementary material for: Changes in the Staphylococcus aureus Transcriptome during Early Adaptation to the Lung
Source: PLoS One. 2012 Aug 2;7(8):e41329. doi: 10.1371/journal.pone.0041329 (PMC3410880; doi:10.1371/journal.pone.0041329)
Supplement: Table S6 — Table of Affymetrix GPL4047 gene chip probes downregulated in cells harvested after 30′ in vivo compared to stationary phase cells. Columns 1 through 16 give the Affymetrix probe identifier, the log2 probe intensities for the stationary phase, early log phase, 30′, 120, and 360′ in vivo samples, the S. aureus strain COL, N315, MSRA, MSSA, Mu50, and MW2 gene designations, the gene name, the gene product function, the TIGR main role category and TIGR subcategory role, respectively. The following 4 columns give the fold change difference from stationary phase for the early log phase (LB), 30′ (30′ in vivo), 120′ (120′ in vivo) and 360′ (360′ in vivo) derived cells. The values are color coded with green indicating significantly downregulated and dark gold indicating significantly upregulated. White indicates no significant difference. (PDF) [file pone.0041329.s008.pdf]

Systematic	Stat.	Log	30'	120'	360'	COL	N315	MRSA	MSSA	Mu50	MW2	GENE NAME	Protein Function	TIGR Main Role	TIGR Sub Role	Fold change vs stationary phase			
																LB	30' in vivo	120' in vivo	360' in vivo
WAN014HEZ_at	1.9	1.8	0.9	1.0	1.4	SA0873	SA0756	SAR0860	SAS0769	SAV0829	MW0782	aroD	3-dehydroquinate dehydratase, type I	Amino acid biosynthesis	Aromatic amino acid family	-1.05	-2.06	-1.97	-1.39
WAN014G39_at	13.9	1.9	2.4	2.0	1.6			SAR2109	SAS1926	SAV2006	MW1943	dapE	succinyl-diaminopimelate desuccinylase	Amino acid biosynthesis	Aspartate family	-7.43	-5.83	-6.83	-8.84
WAN014GTL_at	26.4	14.7	10.4	9.1	10.3	SA1072	SA0915	SAR1037	SAS0999	SAV1063	MW0946	folD	methylenetetrahydrofolate dehydrogenase/methenyltetrahydrofolate cyclohydrolase	Biosynthesis of cofactors, prosthetic groups, and carriers	Folic acid	-1.79	-2.54	-2.91	-2.57
WAN014GZ4_at	8.0	5.8	3.2	2.6	2.5	SA1461	SA1259	SAR1439	SAS1369	SAV1426	MW1316	folA	dihydrofolate reductase	Biosynthesis of cofactors, prosthetic groups, and carriers	Folic acid	-1.39	-2.49	-3.03	-3.22
WAN014G9Z_at	11.2	8.7	4.5	3.9	5.0	SA2638	SA2412					cysG	siroheme synthase, putative	Biosynthesis of cofactors, prosthetic groups, and carriers	Heme, porphyrin, and cobalamin	-1.28	-2.48	-2.90	-2.25
WAN014G77_at	12.7	7.4	5.2	5.5	3.5	SA2267	SA2069	SAR2358	SAS2164	SAV2274	MW2192	moaC	molybdenum cofactor biosynthesis protein C	Biosynthesis of cofactors, prosthetic groups, and carriers	Molybdopterin	-1.72	-2.44	-2.32	-3.60
WAN014FSO_at	6.1	8.1	2.9	2.6	3.2	SA2266	SA2068	SAR2357	SAS2163	SAV2273	MW2191	moeA	molybdopterin biosynthesis MoeA protein, putative	Biosynthesis of cofactors, prosthetic groups, and carriers	Molybdopterin	1.32	-2.10	-2.38	-1.90
WAN014FS8_at	7.3	5.7	3.7	2.9	3.3	SA2262	SA2064	SAR2353	SAS2159	SAV2269	MW2187	mobA	molybdopterin-guanine dinucleotide biosynthesis protein A, putative	Biosynthesis of cofactors, prosthetic groups, and carriers	Molybdopterin	-1.28	-1.99	-2.51	-2.20
WAN014G7D_at	36.5	0.9	3.6	1.6	2.4	SA2579	SA2351	SAR2646	SAS2450	SAV2564	MW2485	crtP	phytoene dehydrogenase, putative	Biosynthesis of cofactors, prosthetic groups, and carriers	Other	-42.73	-10.04	-22.38	-15.40
WAN014ITV_at	15.4	3.7	5.1	3.0	3.3	SA0172	SA0181					entB	isochorismatase (2,3 dihydro-2,3 dihydroxybenzoate synthase)	Biosynthesis of cofactors, prosthetic groups, and carriers	Other	-4.21	-3.00	-5.21	-4.64
WAN014HT6_at	4.4	6.6	1.6	1.4	2.4	SA1975	SA1729	SAR2006	SAS1837	SAV1913	MW1854	pncB	nicotinate phosphoribosyltransferase	Biosynthesis of cofactors, prosthetic groups, and carriers	Pyridine nucleotides	1.52	-2.74	-3.13	-1.85
WAN014HT4_at	4.0	5.4	1.6	1.2	1.5	SA1974	SA1728	SAR2005	SAS1836	SAV1912	MW1853	nadE	NH(3)-dependent NAD+ synthetase	Biosynthesis of cofactors, prosthetic groups, and carriers	Pyridine nucleotides	1.35	-2.48	-3.22	-2.61
WAN014H1C_at	19.1	7.5	3.3	7.1	11.8	SA0564	SA0477	SAR0522	SAS0476	SAV0519	MW0474	pdxS	pyridoxine biosynthesis protein	Biosynthesis of cofactors, prosthetic groups, and carriers	Pyridoxine	-2.54	-5.76	-2.69	-1.62
WAN014HYO_at	1.3	1.7	0.6	0.7		SA2083	SA1894	SAR2180	SAS1995	SAV2091	MW2014	thiE	thiamine-phosphate pyrophosphorylase	Biosynthesis of cofactors, prosthetic groups, and carriers	Thiamine	1.24	-2.17	-1.97	
WAN014G6B_at	32.8	8.7	0.9	0.3	1.1	SA0177	SA0185	SAR0192	SAS0166	SAV0191	MW0165	murQ	N-acetylmuramic acid 6-phosphate etherase [EC:4.2.-.-], Glucokinase regulator-relat	Cell envelope	Biosynthesis and degradation of murein sacculus and peptidoglycan	-3.77	-34.79	-113.71	-29.99
WAN014I4K_at	22.4	1.6	3.3	2.0	3.4	SA0139	SA0147	SAR0154	SAS0127	SAV0152	MW0127	cap5D	cap5D protein	Cell envelope	Biosynthesis and degradation of surface polysaccharides and lipopolysaccharides	-14.32	-6.81	-11.38	-6.64
WAN014I40_at	15.1	1.0	2.3	1.1	1.4	SA0138	SA0146	SAR0153	SAS0126	SAV0151	MW0126	cap5C	cap5C protein	Cell envelope	Biosynthesis and degradation of surface polysaccharides and lipopolysaccharides	-14.51	-6.55	-13.99	-10.74
WAN014I6E_at	6.2	1.0	1.0	0.7	1.1	SA0142	SA0150	SAR0157	SAS0130	SAV0155	MW0130	cap5G	UDP-N-acetylglucosamine 2-epimerase Cap5G	Cell envelope	Biosynthesis and degradation of surface polysaccharides and lipopolysaccharides	-6.21	-6.44	-9.50	-5.69
WAN014G7B_at	14.8		2.3	1.2	2.2	SA2578	SA2350	SAR2645	SAS2449	SAV2563	MW2484	crtQ	glycosyl transferase family protein	Cell envelope	Biosynthesis and degradation of surface polysaccharides and lipopolysaccharides		-6.35	-11.95	-6.61
WAN014I2T_at	19.8	1.6	3.3	2.2	3.3	SA0136	SA0144	SAR0151	SAS0124	SAV0149	MW0124	cap5A	cap5A protein	Cell envelope	Biosynthesis and degradation of surface polysaccharides and lipopolysaccharides	-12.45	-6.02	-9.08	-5.98
WAN014I5T_at	6.3	1.1	1.1	0.5	1.3	SA0141	SA0149	SAR0156	SAS0129	SAV0154	MW0129	cap5F	cap5F protein	Cell envelope	Biosynthesis and degradation of surface polysaccharides and lipopolysaccharides	-5.70	-5.67	-11.47	-4.89
WAN014HMR_at	4.4	1.1	0.9	0.8	0.8			SAR0159	SAS0132		MW0132	capI	Cap8I	Cell envelope	Biosynthesis and degradation of surface polysaccharides and lipopolysaccharides	-4.03	-5.15	-5.76	-5.54
WAN014FW9_at	7.7	1.3	1.5	0.9	1.1	SA0150	SA0158	SAR0165	SAS0138	SAV0163	MW0138	cap5O	cap5O protein/UDP-N-acetyl-D-mannosaminuronic acid dehydrogenase	Cell envelope	Biosynthesis and degradation of surface polysaccharides and lipopolysaccharides	-5.98	-5.09	-8.48	-6.97
WAN014FUF_at	6.1	1.3	1.2	0.8	1.1	SA0147	SA0155	SAR0162	SAS0135	SAV0160	MW0135	cap5L	cap5L protein/glycosyltransferase, putative	Cell envelope	Biosynthesis and degradation of surface polysaccharides and lipopolysaccharides	-4.60	-4.99	-7.53	-5.49
WAN014HAQ_at	31.5	6.4	6.6	3.9	4.8	SA0764	SA0659	SAR0757	SAS0669	SAV0704	MW0666		glycosyl transferase family protein	Cell envelope	Biosynthesis and degradation of surface polysaccharides and lipopolysaccharides	-4.90	-4.77	-8.17	-6.54
WAN014HMQ_at	2.1		0.5					SAR0158	SAS0131		MW0131	capH	capsular polysaccharide biosynthesis protein Cps4G, putative	Cell envelope	Biosynthesis and degradation of surface polysaccharides and lipopolysaccharides		-4.57		
WAN014H22_at	87.1	52.2	20.5	16.6	21.6	SA1522	SA1312					ebpS	elastin binding protein	Cell envelope	Biosynthesis and degradation of surface polysaccharides and lipopolysaccharides	-1.67	-4.26	-5.26	-4.03
WAN014FV5_at	3.9	1.0	1.0	0.4	1.1	SA0148	SA0156	SAR0163	SAS0136	SAV0161	MW0136	cap5M	capsular polysaccharide biosynthesis galactosyltransferase Cap5M	Cell envelope	Biosynthesis and degradation of surface polysaccharides and lipopolysaccharides	-3.95	-4.08	-9.49	-3.60
WAN014HMS_at	3.5	0.9	1.0		1.5			SAR0160	SAS0133		MW0133	capJ	maltose O-acetyltransferase, putative	Cell envelope	Biosynthesis and degradation of surface polysaccharides and lipopolysaccharides	-3.83	-3.67		-2.41
WAN014FVP_at	2.4		0.7			SA0149	SA0157	SAR0164	SAS0137	SAV0162	MW0137	cap5N	cap5N protein/UDP-glucose 4-epimerase, putative	Cell envelope	Biosynthesis and degradation of surface polysaccharides and lipopolysaccharides		-3.63		
WAN014I06_at	28.5	15.2	8.0	6.9	7.8	SA2103	SA1913	SAR2199	SAS2014	SAV2111	MW2035	wecB	UDP-N-acetylglucosamine 2-epimerase	Cell envelope	Biosynthesis and degradation of surface polysaccharides and lipopolysaccharides	-1.88	-3.55	-4.12	-3.67
WAN014FX0_at	6.2	1.9	1.8	1.2	1.2	SA0151	SA0159	SAR0166	SAS0139	SAV0164	MW0139	cap5P	UDP-N-acetylglucosamine 2-epimerase Cap5P	Cell envelope	Biosynthesis and degradation of surface polysaccharides and lipopolysaccharides	-3.35	-3.46	-5.35	-5.05
WAN014I59_at	4.2	1.1	1.2	0.9	1.0	SA0140	SA0148	SAR0155	SAS0128	SAV0153	MW0128	cap5E	cap5E protein	Cell envelope	Biosynthesis and degradation of surface polysaccharides and lipopolysaccharides	-3.78	-3.45	-4.86	-4.29
WAN014HMT_at	2.0	0.8	0.6					SAR0161	SAS0134		MW0134	capK	porS protein, putative, cps flipase	Cell envelope	Biosynthesis and degradation of surface polysaccharides and lipopolysaccharides	-2.49	-3.45		
WAN014G3P_at	4.5	3.8	1.5	1.6	1.8	SA2508	SA2288	SAR2579	SAS2386	SAV2500	MW2419	galU	UTP-glucose-1-phosphate uridylyltransferase	Cell envelope	Biosynthesis and degradation of surface polysaccharides and lipopolysaccharides	-1.17	-2.98	-2.74	-2.49
WAN014I3E_at	#N/A	#N/A	#N/A	#N/A	#N/A	SA0137	SA0145					cap5B	cap5B exopolysaccharide biosynthesis protein, putative	Cell envelope	Biosynthesis and degradation of surface polysaccharides and lipopolysaccharides				
WAN014HWX_at	13.7	16.8	5.0	4.4	3.3	SA1161	SA0997	SAR1123	SAS1084	SAV1151	MW1033	murI	glutamate racemase	Cell envelope	Biosynthesis of murein sacculus and peptidoglycan	1.22	-2.76	-3.09	-4.21
WAN014H7K_at	3.3	3.5	1.5	1.3	1.4	SA0697	SA0596	SAR0650	SAS0606	SAV0640	MW0602	tagX	tagX protein	Cell envelope	Biosynthesis of murein sacculus and peptidoglycan	1.05	-2.15	-2.49	-2.28
WAN014H7I_at	2.5	1.5	1.2	1.0	1.1	SA0696	SA0595	SAR0649	SAS0605	SAV0639	MW0601	tagB	tagB protein	Cell envelope	Biosynthesis of murein sacculus and peptidoglycan	-1.65	-2.10	-2.57	-2.39
WAN014H6O_at	12.2	14.2	6.1	5.9	5.7	SA1609	SA1381	SAR1629	SAS1490	SAV1552	MW1504	pbp3	penicillin-binding protein 3	Cell envelope	Biosynthesis of murein sacculus and peptidoglycan	1.17	-1.99	-2.06	-2.15
WAN014G6F_at	62.1	1.0	4.2	2.2	3.1	SA2197	SA2006	SAR2295	SAS2104	SAV2205	MW2130		map protein, putative	Cell envelope	Other	-62.06	-14.64	-28.71	-20.04
WAN014HXM_at	8.2	2.0	0.7	2.2	2.4	SA1169	SA1004		SAS1092	SAV1159	MW1041	fib	fibrinogen-binding protein precursor-related protein	Cell envelope	Other	-4.04	-11.80	-3.70	-3.44
Y18641-cds1_at	88.5	2.9	21.2	11.8	18.8			SAR2780	SAS2584	SAV2700	MW2619		membrane protein, putative	Cell envelope	Other	-30.26	-4.18	-7.53	-4.72
WAN014GQH_s_a	11.7	2.9	3.0	2.6	3.8					SAV0811			fibrinogen-binding protein	Cell envelope	Other	-3.99	-3.85	-4.49	-3.07
WAN014FY4_at	10.1	14.7	2.8	2.5	2.6	SA0411	SA0329	SAR0338	SAS0317	SAV0341	MW0317		membrane protein, putative	Cell envelope	Other	1.45	-3.63	-3.98	-3.94
WAN014GUU_at	2.3	3.8	0.7	0.8	0.9	SA1139	SA0977		SAS1064	SAV1130	MW1012	sai-1	29-kDa cell surface protein, staphylococcal cell wall anchored protein	Cell envelope	Other	1.66	-3.18	-3.00	-2.58
WAN014HVL_x_at	25.6	19.4	8.2	8.3	6.9	SA2019	SA1839			SAV2032	MW1956		sdrH protein, putative	Cell envelope	Other	-1.32	-3.13	-3.09	-3.68
WAN014GJY_at	6.3	5.2	2.4	2.5	2.4	SA0632	SA0543	SAR0591	SAS0544	SAV0586	MW0541		membrane protein, putative	Cell envelope	Other	-1.21	-2.59	-2.49	-2.59
WAN014GFM_at	20.7	16.1	8.4	6.4	7.4	SA0466	SA0380	SAR0420	SAS0382	SAV0420	MW0380		membrane protein, putative	Cell envelope	Other	-1.28	-2.47	-3.24	-2.80
WAN014HVM_at	1.3	0.9	0.6	0.7		SA1141	SA0978	SAR1104	SAS1065	SAV1131	MW1013	isdC	NPQTN cell wall surface anchor protein	Cell envelope	Other	-1.49	-2.24	-1.84	
WAN014HM5_at	114.0	41.2	8.1	4.5	5.6	SA0985	SA0841	SAR0944	SAS0851	SAV0981	MW0863		map protein, putative	Cell envelope	Pathogenesis	-2.77	-14.09	-25.60	-20.42
WAN014FY9_at	47.4	37.7	6.6	6.5	7.7	SA1753	SA1528	SAR1784	SAS1633	SAV1706	MW1649		universal stress protein family	Cellular processes	Adaptations to atypical conditions	-1.26	-7.14	-7.26	-6.18
WAN014FYF_at	432.5	267.2	73.1	87.3	205.3	SA1759	SA1532	SAR1788	SAS1637	SAV1710	MW1653		universal stress protein family	Cellular processes	Adaptations to atypical conditions	-1.62	-5.92	-4.95	-2.11
WAN014FVE_at	62.0	3.0	11.8	6.8	6.5	SA2321	SA2119	SAR2413	SAS2221	SAV2328	MW2249	ydaD	general stress protein 39	Cellular processes	Adaptations to atypical conditions	-20.41	-5.26	-9.12	-9.58
WAN014IKO_at	5.0	0.8	1.3	1.0	1.2		SA2170		SAS2272	SAV2382	MW2302		general stress protein 26	Cellular processes	Adaptations to atypical conditions	-5.86	-3.83	-4.79	-4.13
WAN014I3H_at	423.3	10.5	138.0	74.5	96.8	SA2173	SA1984	SAR2273	SAS2083	SAV2182	MW2108	asp23	alkaline shock protein 23	Cellular processes	Adaptations to atypical conditions	-40.44	-3.07	-5.68	-4.37
WAN014HB3_at	29.1	37.1	11.5	11.5	11.8	SA1689	SA1460	SAR1714	SAS1570	SAV1634	MW1584	relA	GTP pyrophosphokinase	Cellular processes	Adaptations to atypical conditions	1.27	-2.54	-2.52	-2.47
WAN014H8C_at	2.5	2.2	1.1	1.0	1.5	SA1638	SA1410	SAR1658	SAS1519	SAV1581	MW1533	grpE	heat shock protein GrpE	Cellular processes	Adaptations to atypical conditions	-1.11	-2.36	-2.39	-1.67
WAN014IN0_at	1.8	1.3	0.7	0.8	1.4	SA0858	SA0744					ssp	secretory extracellular matrix and plasma binding protein	Cellular processes	Cell adhesion	-1.39	-2.52	-2.18	-1.24
WAN014GP8_at	30.2	3.0	6.5	4.3	5.2	SA0834	SA0724	SAR0825	SAS0734	SAV0769	MW0731		cell division inhibitor	Cellular processes	Cell division	-10.03	-4.66	-7.08	-5.83
WAN014GAE_at	221.1	145.9	25.1	43.9	105.3	SA0452	SA0366	SAR0399	SAS0358	SAV0381	MW0357	ahpC	alkyl hydroperoxide reductase, subunit C	Cellular processes	Detoxification	-1.52	-8.80	-5.04	-2.10
WAN014GW0_at	12.2	12.3	3.0	3.9	6.1	SA1325	SA1146	SAR1280	SAS1238	SAV1306	MW1188	gpxA	glutathione peroxidase	Cellular processes	Detoxification	1.00	-4.12	-3.15	-2.01
WAN014FT5_at	116.3	35.5	30.2	28.5	37.6	SA1368	SA1170	SAR1344	SAS1274	SAV1334	MW1221	katA	catalase	Cellular processes	Detoxification	-3.28	-3.85	-4.08	-3.09
WAN014G9N_at	602.4	465.6	159.6	199.1	519.3	SA0451	SA0365	SAR0398	SAS0357	SAV0380	MW0356	ahpF	alkyl hydroperoxide reductase, subunit F	Cellular processes	Detoxification	-1.29	-3.77	-3.02	-1.16
WAN014HF3_at	10.0	11.1	3.1	3.1	3.6	SA0876	SA0759	SAR0863	SAS0772	SAV0832	MW0785		arsenate reductase, putative	Cellular processes	Detoxification	1.11	-3.27	-3.28	-2.81
WAN014H6Q_at	238.7	56.2	82.5	78.0	106.7	SA1610	SA1382	SAR1630	SAS1491	SAV1553	MW1505	sodA	superoxide dismutase	Cellular processes	Detoxification	-4.25	-2.90	-3.06	-2.24
WAN014FYO_at	6.5	4.2	3.2	3.0	3.1	SA1771	SA1543	SAR1799	SAS1648	SAV1722	MW1664		organic hydroperoxide resistance protein, putative	Cellular processes	Detoxification	-1.56	-2.04	-2.17	-2.10
WAN014H8N_at	25.8	20.0	9.3	10.2	9.5	SA1645	SA1417	SAR1666	SAS1526	SAV1589	MW1540	comEB	comE operon protein 2	Cellular processes	DNA transformation	-1.29	-2.77	-2.52	-2.72
WAN014G3C_at	7.4	5.7	2.4	2.2	3.3	SA2018	SA1838	SAR2118	SAS1937	SAV2031	MW1955	abi	abortive infection protein	Cellular processes	Other	-1.30	-3.13	-3.37	-2.27
WAN014IP2_at	51.3	1.4	1.9	1.5	3.2	SA1866	SA1628					splD	serine protease SplD	Cellular processes	Pathogenesis	-38.01	-27.09	-34.38	-16.25
WAN014IP6_at	13.4	0.8	1.1	1.4	1.8	SA1868	SA1630		SAS1735	SAV1812	MW1754	splB	serine protease SplB	Cellular processes	Pathogenesis	-16.79	-12.42	-9.58	-7.60
WAN014ITR_x_at	18.4	51.6	1.8	8.7	17.3			SAR0114					immunoglobulin G binding protein A precursor	Cellular processes	Pathogenesis	2.81	-10.31	-2.11	-1.06
WAN014HLM_at	11.2		1.1	0.9	1.3	SA1869	SA1631					splA	serine protease SplA	Cellular processes	Pathogenesis		-10.18	-12.00	-8.45
WAN014ITQ_x_at	14.7	39.7	1.7	7.2	14.2	SA0095		SAR0114	SAS0085		MW0084	spa	immunoglobulin G binding protein A precursor	Cellular processes	Pathogenesis	2.69	-8.84	-2.05	-1.04
WAN01BQ8O_at	7.0	0.7	1.0	0.7		SA1863						splF	serine protease SplF	Cellular processes	Pathogenesis	-10.14	-7.08	-10.59	
WAN014IPZ_at	254.0	103.2	41.3	29.5	75.2	SA2023						agrB	accessory gene regulator protein B	Cellular processes	Pathogenesis	-2.46	-6.14	-8.60	-3.38
WAN01BQD3_at	343.3	178.4	58.3	34.5	96.9							agrD	accessory gene regulator protein D	Cellular processes	Pathogenesis	-1.92	-5.88	-9.96	-3.54
WAN014G3O_at	325.4	120.0	63.0	40.2	113.6	SA2026	SA1844	SAR2126	SAS1944	SAV2039	MW1963	agrA	accessory gene regulator protein A	Cellular processes	Pathogenesis	-2.71	-5.17	-8.09	-2.86
WAN014IPG_at	51.8	27.9	11.4	8.6	22.6	SA2025						argC	accessory gene regulator protein C	Cellular processes	Pathogenesis	-1.86	-4.56	-6.06	-2.29
WAN014HIH_at	26.9	10.5	6.2	3.8	4.2	SA1812	SA1583	SAR1847	SAS1688	SAV1764	MW1705	rot	virulence factor regulator protein	Cellular processes	Pathogenesis	-2.56	-4.31	-7.04	-6.39
WAN014IQ2_at	7.8	3.4	2.2	2.8	3.4	SA2023	SA1842	SAR2123	SAS1941	SAV2036	MW1960	agrB	accessory gene regulator protein B	Cellular processes	Pathogenesis	-2.26	-3.52	-2.77	-2.27
WAN014IPJ_at	9.4	6.1	2.9	3.4	5.7	SA2025	SA1843			SAV2038		argC	accessory gene regulator protein C	Cellular processes	Pathogenesis	-1.53	-3.22	-2.75	-1.65
WAN014HYX_at	10.4	13.7	3.4	4.7	3.3	SA0270	SA0270	SAR0278	SAS0257	SAV0281	MW0257	ssaA homo	secretory antigen precursor SsaA	Cellular processes	Pathogenesis	1.31	-3.02	-2.23	-3.20
WAN014HIQ_at	17.6	9.7	8.7	8.3	11.7		#N/A						beta-lactamase	Cellular processes	Pathogenesis	-1.82	-2.02	-2.13	-1.51
WAN014HY9_at	256.5	170.5	3.7	7.4	26.4	SA1186		SAR1150	SAS1107a		MW1056	psmB1	antibacterial protein	Cellular processes	Toxin production and resistance	-1.50	-69.74	-34.44	-9.72
WAN014HYB_at	326.0	203.3	7.0	11.9	57.1	SA1187			SAS1108		MW1057	psmB2	antibacterial protein	Cellular processes	Toxin production and resistance	-1.60	-46.69	-27.49	-5.71
WAN014GB3_at	44.7	4.8	1.2	1.5	3.3	SA2422	SA2209	SAR2511	SAS2312	SAV2421	MW2344	hlgB	leukocidin precursor, F subunit	Cellular processes	Toxin production and resistance	-9.31	-36.76	-29.94	-13.42
WAN014HVN_at	443.1	118.2	14.5	21.3	136.4	SA2022	SAS065	SAR2122	SAS1940a	SAV2035	MW1959	hld	delta-hemolysin	Cellular processes	Toxin production and resistance	-3.75	-30.46	-20.80	-3.25
WAN014GAY_at	16.9	3.2	0.7	0.8	4.0	SA2421	SA2208	SAR2510	SAS2311	SAV2420	MW2343	hlgC	leukocidin precursor, S subunit	Cellular processes	Toxin production and resistance	-5.29	-24.99	-21.06	-4.22
WAN014GQ9_at	66.6	8.3	4.6	4.1	5.3	SA0317	SA0309	SAR0317	SAS0297	SAV0320	MW0297	geh	glycerol ester hydrolase	Cellular processes	Toxin production and resistance	-8.01	-14.51	-16.13	-12.64
WAN01CBPA_at	113.5	119.4	12.7	37.8	170.8	Jot annotate	#N/A	Jot annotate	Jot annotate	Jot annotate	Jot annotate	psma1.2.3.	Leucotoxin, haemolysin	Cellular processes	Toxin production and resistance	1.05	-8.91	-3.00	1.50
WAN014HLV_at	42.0	5.2	5.9	3.1	3.4	SA1872			SAS1740		MW1759	epiE	epidermin immunity protein F	Cellular processes	Toxin production and resistance	-8.03	-7.09	-13.44	-12.42
WAN014IP9_at	9.3	1.9	1.4	1.9	4.6		trunca			SAV1938	MW1880		truncated cell surface protein map-w	Cellular processes	Toxin production and resistance	-4.80	-6.53	-4.79	-2.01
WAN014HM1_at	5.8	1.0	1.0	0.7		SA1873			SAS1741		MW1760	epiF	epidermin immunity protein F	Cellular processes	Toxin production and resistance	-5.81	-5.86	-7.78	
WAN014IPB_s_at	11.3	2.6	2.0	3.0	6.7					SAV1938	MW1880		truncated cell surface protein map-w	Cellular processes	Toxin production and resistance	-4.36	-5.61	-3.79	-1.68
WAN014HMJ_at	5.2	1.1	1.0	1.0	1.5	SA1880	SA1637		SAS1748	SAV1819	MW1767	lukD	leukotoxin LukD	Cellular processes	Toxin production and resistance	-4.86	-5.45	-5.42	-3.52
WAN014HSP_at	230.7	97.1	44.1	59.3	66.5			SAR0694					putative bacteriocin precursor	Cellular processes	Toxin production and resistance	-2.38	-5.23	-3.89	-3.47
WAN014GDW_at	2.4	3.2	0.5			SA2712	SA2480	SAR2770	SAS2574	SAV2688	MW2608	drp35	Drp35 protein	Cellular processes	Toxin production and resistance	1.34	-5.11		
WAN014ITS_at	41.5	8.6	8.3	10.2	6.5	SA1173	SA1007		SAS1097	SAV1163	MW1044	hla	alpha-hemolysin precursor	Cellular processes	Toxin production and resistance	-4.80	-5.01	-4.06	-6.36
WAN014HUM_at	2.8	3.4	0.6	0.4	1.1	SA2006	SA1813		SAS1925	SAV2005	MW1942	lukM	leukotoxin LukM	Cellular processes	Toxin production and resistance	1.24	-4.27	-7.18	-2.53
WAN014GBY_at	9.6	5.7	2.7	1.9	3.2	SA2670	SA2441	SAR2727	SAS2534	SAV2648	MW2569	gtfA	glycosyl transferase, group 1 family protein	Cellular processes	Toxin production and resistance	-1.69	-3.56	-5.16	-3.00

																Fold change vs stationary phase			
Systematic	Stat.	Log	30'	120'	360'	COL	N315	MRSA	MSSA	Mu50	MW2	GENE NAME	Protein Function	TIGR Main Role	TIGR Sub Role	LB	30' in vivo	120' in vivo	360' in vivo
WAN014HML_at	2.9		0.9	0.7	0.9	SA1881	SA1638		SAS1749	SAV1820	MW1768	lukS	synergohymenotropic toxin lukS	Cellular processes	Toxin production and resistance		-3.45	-4.19	-3.39
WAN014IP8_x_at	3.3	1.4	1.0	1.2	2.8		SA1751					mapW_2	truncated cell surface protein map-w	Cellular processes	Toxin production and resistance	-2.45	-3.29	-2.82	-1.19
WAN014HLQ_at	3.3	0.9	1.1	0.5	0.8	SA1871	SA1634		SAS1739		MW1758	epiG	epidermin immunity protein F	Cellular processes	Toxin production and resistance	-3.52	-3.08	-6.79	-4.12
WAN014IL9_at	1.7	1.2	0.6	0.9	1.0		SA2317			SAV2529			N-acetyltransferase	Cellular processes	Toxin production and resistance	-1.41	-2.77	-1.95	-1.68
WAN014G2X_at	6.5	8.1	2.5	2.4	1.6	SA1988	SA1739	SAR2017	SAS1849	SAV1925	MW1866		acyl-coenzyme A:6-aminopenicillanic acid acyl-transferase	Cellular processes	Toxin production and resistance	1.23	-2.63	-2.72	-4.02
WAN014I2D_at	13.3	15.8	5.2	4.0	4.3	SA2151	SA1965	SAR2252	SAS2063	SAV2161	MW2088	femD	femD protein, phosphoglucosamine mutase	Cellular processes	Toxin production and resistance	1.19	-2.54	-3.35	-3.06
WAN014GIV_at	18.2	13.0	7.2	6.7	6.8	SA0612	SA0523	SAR0569	SAS0523	SAV0565	MW0520		glycosyl transferase, group 1	Cellular processes	Toxin production and resistance	-1.40	-2.53	-2.72	-2.67
WAN014H3O_at	2.4	2.8	1.0	0.9		SA0611	SA0522	SAR0568	SAS0522	SAV0564	MW0519		glycosyl transferase, group 1	Cellular processes	Toxin production and resistance	1.16	-2.50	-2.65	
WAN014A82-seq2	12.6	12.0	5.4	5.2	8.6	SA0164	SA0173	SAR0180	SAS0154	SAV0179	MW0153		gramicidin S synthetase 2	Cellular processes	Toxin production and resistance	-1.05	-2.33	-2.44	-1.47
WAN014GBT_at	8.7	5.3	4.0	2.6	2.9	SA2669	SA2440	SAR2726	SAS2533	SAV2647	MW2568	gtfB	conserved hypothetical protein	Cellular processes	Toxin production and resistance	-1.63	-2.18	-3.31	-3.01
WAN014GVE_at	3.4	3.6	1.6	1.3	1.3	SA1184	SA1016	SAR1147	SAS1106	SAV1173	MW1054		exfoliative toxin A	Cellular processes	Toxin production and resistance	1.06	-2.13	-2.58	-2.68
WAN014IUC_at	1.6	1.4	0.7	0.6	1.6	SA2004	SA1812					lukF	leukocidin precursor, F subunit, putative	Cellular processes	Toxin production and resistance	-1.11	-2.13	-2.44	1.02
WAN014I2A_at	30.7	15.9	4.1	3.6	5.0	SA2145	SA1959	SAR2242	SAS2055	SAV2154	MW2080	glmS	glucosamine--fructose-6-phosphate aminotransferase (isomerizing)	Central intermediary metabolism	Amino sugars	-1.93	-7.57	-8.58	-6.12
WAN014HOF_at	57.4	6.8	14.6	10.2	10.1	SA1912	SA1671	SAR1945	SAS1777	SAV1854	MW1795		glucosamine-6-phosphate isomerase , putative	Central intermediary metabolism	Amino sugars	-8.48	-3.93	-5.64	-5.69
WAN014G81_at	19.1	14.0	1.8	1.9	2.3	SA2296	SA2098	SAR2389	SAS2196	SAV2305	MW2224		D-isomer specific 2-hydroxyacid dehydrogenase family protein	Central intermediary metabolism	Other	-1.37	-10.34	-9.81	-8.34
WAN014G76_at	22.8	1.6	3.2	1.7	4.6	SA2576	SA2348	SAR2642	SAS2447	SAV2561	MW2482	crtN	dehydrosqualene desaturase	Central intermediary metabolism	Other	-13.99	-7.08	-13.58	-4.98
WAN014G79_at	7.3		1.3	1.0	2.3	SA2577	SA2349		SAS2448	SAV2562	MW2483	crtM	dehydrosqualene synthase	Central intermediary metabolism	Other		-5.74	-7.55	-3.25
WAN014I0W_at	150.0	9.0	35.2	17.9	17.9	SA2114	SA1924	SAR2210	SAS2025	SAV2122	MW2046	aldA	glycine betaine aldehyde dehydrogenase	Central intermediary metabolism	Other	-16.59	-4.26	-8.37	-8.37
WAN014HJL_at	12.8	14.4	4.2	4.0	4.1	SA0932	SA0791	SAR0892	SAS0800	SAV0930	MW0812		D-isomer specific 2-hydroxyacid dehydrogenase family protein	Central intermediary metabolism	Other	1.13	-3.02	-3.17	-3.09
WAN014G72_at	10.3	5.4	3.5	2.7	3.0	SA2574	SA2346	SAR2640	SAS2445	SAV2559	MW2480		D-isomer specific 2-hydroxyacid dehydrogenase family protein	Central intermediary metabolism	Other	-1.91	-2.95	-3.83	-3.46
WAN014HK9_at	6.5	4.4	2.8	2.6	2.1	SA1843	SA1614					menC	o-succinylbenzoic acid (OSB) synthetase	Central intermediary metabolism	Other	-1.49	-2.29	-2.46	-3.09
WAN014H3I_at	7.5	8.3	3.6	3.3	2.8	SA0607	SA0518	SAR0565	SAS0518	SAV0560	MW0515		azoreductase	Central intermediary metabolism	Other	1.11	-2.08	-2.25	-2.64
WAN014GA0_at	3.8	4.6	1.2	1.2	1.6	SA2639	SA2413	SAR2698	SAS2506	SAV2620	MW2540	cysJ	sulfite reductase (NADPH) flavoprotein alpha-component	Central intermediary metabolism	Sulfur metabolism	1.21	-3.04	-3.29	-2.41
WAN014H1M_at	454.5	369.2	212.4	238.3	291.5	SA1513	SA1305	SAR1482	SAS1414	SAV1473	MW1362	hup	DNA-binding protein HU	DNA metabolism	Chromosome-associated proteins	-1.23	-2.14	-1.91	-1.56
WAN014HEP_at	39.9	4.7	1.3	1.7	2.5	SA0860	SA0746	SAR0847	SAS0756	SAV0815	MW0769	nuc	thermonuclease precursor	DNA metabolism	Degradation of DNA	-8.55	-31.38	-23.11	-16.06
WAN014IKP_at	31.9	5.7	6.0	6.3	7.9		#N/A					pinR	DNA invertase Pin homolog	DNA metabolism	DNA replication, recombination and repair	-5.59	-5.34	-5.07	-4.05
WAN014FY7_at	55.3	60.8	12.4	15.4	13.9	SA1711	SA1489	SAR1744	SAS1593	SAV1664	MW1608	tag	DNA-3-methyladenine glycosylase I	DNA metabolism	DNA replication, recombination, and repair	1.10	-4.46	-3.59	-3.98
WAN014GUK_at	17.1	9.4	4.8	4.2	4.6	SA0004	SA0004	SAR0004	SAS0004	SAV0004	MW0004	recF	recF protein	DNA metabolism	DNA replication, recombination, and repair	-1.83	-3.60	-4.10	-3.72
WAN014HDY_at	8.0	8.1	2.7	2.3	2.6	SA1737	SA1513	SAR1769	SAS1618	SAV1690	MW1633	polA	DNA polymerase I	DNA metabolism	DNA replication, recombination, and repair	1.02	-2.95	-3.50	-3.10
WAN014H0J_at	38.7	25.7	13.2	10.6	14.4	SA0005	SA0005	SAR0005	SAS0005	SAV0005	MW0005	gyrB	DNA gyrase, B subunit	DNA metabolism	DNA replication, recombination, and repair	-1.51	-2.93	-3.66	-2.68
WAN014HNJ_at	4.9	6.0	1.9	1.8	1.6	SA1900	SA1662	SAR1935	SAS1765	SAV1844	MW1785		DNA repair exonuclease family protein	DNA metabolism	DNA replication, recombination, and repair	1.23	-2.53	-2.71	-3.00
WAN014HL7_at	9.7	13.1	3.9	3.4	3.6	SA0971	SA0828	SAR0929	SAS0837	SAV0967	MW0849	rexA	exonuclease RexA	DNA metabolism	DNA replication, recombination, and repair	1.35	-2.52	-2.82	-2.68
WAN014H6G_at	71.9	51.7	30.9	23.1	38.5	SA0006	SA0006	SAR0006	SAS0006	SAV0006	MW0006	gyrA	DNA gyrase, A subunit	DNA metabolism	DNA replication, recombination, and repair	-1.39	-2.32	-3.11	-1.87
WAN014GZ5_at	2.9	3.5	1.4	1.2	1.5	SA0526	SA0442	SAR0485	SAS0441	SAV0484	MW0439	holB	DNA polymerase III, delta prime subunit	DNA metabolism	DNA replication, recombination, and repair	1.20	-2.15	-2.38	-1.90
WAN014H42_at	4.7	3.7	2.2	2.0	2.6	SA1564	SA1350	SAR1597	SAS1458	SAV1519	MW1472	recN	DNA repair protein RecN	DNA metabolism	DNA replication, recombination, and repair	-1.27	-2.13	-2.40	-1.84
WAN014HD1_at	52.1	26.4	25.9	21.7	31.4	SA0824	SA0714	SAR0813	SAS0724	SAV0759	MW0721	uvrA	excinuclease ABC, A subunit	DNA metabolism	DNA replication, recombination, and repair	-1.98	-2.01	-2.41	-1.66
WAN014HFB_at	9.4	1.7	3.7	2.4	3.4	SA0880	SA0768	SAR0867	SAS0776	SAV0835	MW0788		Toprim domain protein	DNA metabolism	Other	-5.52	-2.58	-3.89	-2.75
WAN014G8F_at	5.2	6.3	1.6	1.8	2.4	SA0180	SA0189	SAR0196	SAS0170	SAV0195	MW0169	hsdR	type I restriction-modification enzyme, R subunit, putative	DNA metabolism	Restriction/modification	1.21	-3.21	-2.93	-2.18
WAN014HL3_at	4.0	4.4	1.6	1.6	1.7	SA1862	SA1626	SAR1899	SAS1732	SAV1808	MW1751	hsdM	type I restriction-modification system, M subunit	DNA metabolism	Restriction/modification	1.09	-2.58	-2.55	-2.31
WAN014HL0_at	2.6	2.7	1.3	1.1	1.4	SA1861			SAS1731		MW1750	hsdS	type I restriction-modification enzyme, S subunit, EcoA family	DNA metabolism	Restriction/modification	1.01	-2.05	-2.44	-1.90
WAN014G30_at	14.3	17.7	4.7	4.5	3.2	SA2000	SA1749	SAR2028	SAS1860	SAV1936	MW1878	aspC3	aminotransferase, putative	Energy metabolism	Amino acid transport and metabolism	1.24	-3.05	-3.17	-4.46
WAN014G6S_at	155.6	51.9	4.7	9.9	19.3	SA2569	SA2341	SAR2634	SAS2440	SAV2554	MW2475	rocA	1-pyrroline-5-carboxylate dehydrogenase, putative	Energy metabolism	Amino acids and amines	-3.00	-32.95	-15.67	-8.07
WAN014FZC_at	26.8	6.9	2.4	3.2	4.5	SA1816	SA1585	SAR1849	SAS1690	SAV1766	MW1707	putA	proline dehydrogenase	Energy metabolism	Amino acids and amines	-3.91	-11.37	-8.44	-5.95
WAN01BPXY_at	13.1	2.6	1.2	1.2	2.0	SA1477		SAR1450	SAS1381	SAV1438	MW1327	ilvA	threonine dehydratase, catabolic	Energy metabolism	Amino acids and amines	-4.96	-10.84	-10.86	-6.46
WAN014HKU_at	159.6	72.9	19.6	20.2	40.7	SA0961	SA0819	SAR0920	SAS0828	SAV0958	MW0840	gluD	glutamate dehydrogenase, NAD-specific	Energy metabolism	Amino acids and amines	-2.19	-8.15	-7.88	-3.93
WAN014HKS_at	31.5	13.3	4.0	4.9	7.6	SA0960	SA0818	SAR0919	SAS0827	SAV0957	MW0839	rocD2	ornithine aminotransferase, putative	Energy metabolism	Amino acids and amines	-2.37	-7.97	-6.40	-4.17
WAN014H7P_at	10.6	4.5	1.4	1.4	2.3	SA0173	SA0182	SAR0189	SAS0163	SAV0188	MW0162	ipdC	indolepyruvate decarboxylase, putative	Energy metabolism	Amino acids and amines	-2.37	-7.83	-7.39	-4.52
WAN014HEY_at	98.8	23.6	13.8	16.4	25.2	SA1758	SA1531	SAR1787	SAS1636	SAV1709	MW1652	ald2	alanine dehydrogenase	Energy metabolism	Amino acids and amines	-4.19	-7.16	-6.02	-3.92
WAN014GWS_at	29.2	21.7	5.5	6.1	5.6	SA1399	SAS044	SAR1376	SAS1303	SAV1363	MW1250	dmpI	4-oxalocrotonate tautomerase	Energy metabolism	Amino acids and amines	-1.34	-5.33	-4.78	-5.18
WAN014FVI_at	3.1	1.2	0.6	0.4	1.0	SA2323	SA2121	SAR2416	SAS2223	SAV2330	MW2251	hutI	imidazolonepropionase	Energy metabolism	Amino acids and amines	-2.49	-5.23	-7.87	-3.06
WAN014H2Z_at	63.5	46.9	15.0	13.6	19.5	SA0596	SA0508	SAR0555	SAS0508	SAV0550	MW0505		aminotransferase, class II	Energy metabolism	Amino acids and amines	-1.35	-4.23	-4.65	-3.26
WAN014FVM_at	96.4	5.8	22.8	11.4	13.1	SA2327	SA2125	SAR2420	SAS2226	SAV2334	MW2254	hutG	formiminoglutamase	Energy metabolism	Amino acids and amines	-16.63	-4.22	-8.46	-7.37
WAN014I2J_at	20.7	9.1	5.6	6.8	7.5	SA2154	SA1968					rocF	arginase	Energy metabolism	Amino acids and amines	-2.28	-3.71	-3.04	-2.77
WAN014HF5_at	17.3	14.1	4.7	4.6	5.5	SA0877	SA0760	SAR0864	SAS0773	SAV0833	MW0786	gcvH	glycine cleavage system H protein	Energy metabolism	Amino acids and amines	-1.23	-3.69	-3.76	-3.15
WAN014I0A_at	61.0	41.8	17.8	13.7	20.4	SA2105	SA1915	SAR2201	SAS2016	SAV2113	MW2037	glyA	serine hydroxymethyltransferase	Energy metabolism	Amino acids and amines	-1.46	-3.42	-4.44	-2.98
WAN014HCG_at	5.9	5.5	1.8	2.2	2.9	SA0008	SA0008	SAR0008	SAS0008	SAV0008	MW0008	hutH	histidine ammonia-lyase	Energy metabolism	Amino acids and amines	-1.07	-3.31	-2.65	-2.05
WAN014HH8_at	14.4	13.1	4.4	3.3	3.1	SA1800	SA1571	SAR1835	SAS1676	SAV1750	MW1693	dat	D-amino acid aminotransferase	Energy metabolism	Amino acids and amines	-1.10	-3.30	-4.35	-4.66
WAN014GW2_at	2.6	1.3	1.2	1.4	1.3	SA1349	SA1154	SAR1326	SAS1256	SAV1315	MW1203		threonine aldolase	Energy metabolism	Amino acids and amines	-1.91	-2.14	-1.87	-2.02
WAN014GZS_at	5.1	1.9		0.1		SA1478	SA1272	SAR1451	SAS1382	SAV1439	MW1328	ald1	alanine dehydrogenase	Energy metabolism	Amino acids and amines	-2.73		-40.67	
WAN014FVK_at	5.4	1.9				SA2324	SA2122	SAR2417	SAS2224	SAV2331	MW2252	hutU	urocanate hydratase	Energy metabolism	Amino acids and amines	-2.77			
WAN014FUP_at	24.2	8.5	4.7	3.6	4.6	SA2301	SA2102	SAR2393	SAS2201	SAV2309	MW2229		formate dehydrogenase, alpha subunit, putative	Energy metabolism	Anaerobic	-2.85	-5.18	-6.66	-5.25
WAN014GCU_at	11.8	47.4	2.4	1.9	2.1	SA2535	SA2312	SAR2605	SAS2410	SAV2524	MW2444	ddh	D-isomer specific 2-hydroxyacid dehydrogenase family protein	Energy metabolism	Anaerobic	4.02	-4.83	-6.10	-5.50
WAN014H3D_at	7.2	4.4	1.1	0.9		SA1551	SA1338	SAR1584	SAS1447	SAV1507	MW1461	malA	alpha-glucosidase	Energy metabolism	Biosynthesis and degradation of polysaccharides	-1.66	-6.62	-7.89	
WAN014HVR_at	5.8	6.2	1.7	1.4	1.8	SA2029	SA1846	SAR2128	SAS1946	SAV2041	MW1965	cscA	sucrose-6-phosphate dehydrogenase	Energy metabolism	Biosynthesis and degradation of polysaccharides	1.06	-3.49	-4.12	-3.17
WAN014GM0_at	3.6	22.5	1.1	1.6	2.8	SA0251	SA0256	SAR0264	SAS0243	SAV0266	MW0242	bglA	6-phospho-beta-glucosidase	Energy metabolism	Biosynthesis and degradation of polysaccharides	6.34	-3.31	-2.18	-1.26
WAN014GQ5_at	3.8	8.7	1.3	1.5	1.4	SA0315	SA0307	SAR0315	SAS0295	SAV0318	MW0295	nanE	N-acetylmannosamine-6-P epimerase, putative	Energy metabolism	Biosynthesis and degradation of polysaccharides	2.28	-2.99	-2.52	-2.76
WAN014GYI_at	2.8	67.7	1.3	1.6	2.4	SA0517	SA0433	SAR0474	SAS0432	SAV0475	MW0429	amyA	alpha amylase family protein	Energy metabolism	Biosynthesis and degradation of polysaccharides	24.53	-2.15	-1.73	-1.17
WAN014GVS_at	13.7	9.1	3.4	3.3	4.1	SA0453	SA0367	SAR0400	SAS0359	SAV0382	MW0358		NAD(P)H-flavin oxidoreductase	Energy metabolism	Electron transport	-1.50	-3.98	-4.11	-3.38
WAN014GTJ_at	132.9	173.7	37.4	41.0	37.7	SA1070	SA0913	SAR1034	SAS0996	SAV1061	MW0944	qoxA	quinol oxidase, subunit II	Energy metabolism	Electron transport	1.31	-3.55	-3.24	-3.53
WAN014HFD_at	54.7	10.2	17.2	14.1	19.1	SA0881	SAS023	SAR0868	SAS0777	SAV0836	MW0789		thioredoxin, putative	Energy metabolism	Electron transport	-5.37	-3.17	-3.88	-2.86
WAN014GQC_at	82.4	82.3	26.0	26.6	33.5	SA0875	SA0758	SAR0862	SAS0771	SAV0831	MW0784		thioredoxin, putative	Energy metabolism	Electron transport	-1.00	-3.17	-3.10	-2.46
WAN014GTH_at	564.9	731.0	192.7	208.7	310.0	SA1069	SA0912	SAR1033	SAS0995	SAV1060	MW0943	qoxB	quinol oxidase, subunit I	Energy metabolism	Electron transport	1.29	-2.93	-2.71	-1.82
WAN014GQY_at	46.0	24.6	15.9	13.5	13.3	SA0941	SA0799	SAR0900	SAS0808	SAV0938	MW0820		NADH dehydrogenase, putative, authentic frameshift	Energy metabolism	Electron transport	-1.87	-2.90	-3.40	-3.45
WAN014HDD_at	113.1	95.9	50.4	44.1	63.5	SA0829	SA0719	SAR0818	SAS0729	SAV0764	MW0726	trxB	thioredoxin reductase	Energy metabolism	Electron transport	-1.18	-2.25	-2.56	-1.78
WAN014G5B_at	4.0	6.2	1.8	2.5	2.5	SA2534	SA2311		SAS2409	SAV2523	MW2443		NAD(P)H-flavin oxidoreductase	Energy metabolism	Electron transport	1.56	-2.23	-1.59	-1.59
WAN014HGP_at	36.0	19.1	16.7	10.7	14.7	SA1794	SA1565	SAR1822	SAS1670	SAV1744	MW1687		thioredoxin, putative	Energy metabolism	Electron transport	-1.89	-2.15	-3.35	-2.46
WAN014GTE_at	334.0	464.0	157.3	135.3	183.9	SA1068	SA0911	SAR1032	SAS0994	SAV1059	MW0942	qoxC	quinol oxidase, subunit III	Energy metabolism	Electron transport	1.39	-2.12	-2.47	-1.82
WAN014G45_at	2.2	2.6	0.8	0.8	1.0	SA2515	SA2294	SAR2583	SAS2391	SAV2506	MW2424	gntK	gluconokinase	Energy metabolism	Entner-Doudoroff	1.15	-2.73	-2.69	-2.26
WAN014ILV_at	4.0	5.2	1.5	1.9	2.2				SAS1919		MW1936		repressor	Energy metabolism	Entner-Doudoroff	1.28	-2.68	-2.16	-1.84
WAN014HFY_at	48.9	7.8	1.7	3.4	5.9	SA1783	SA1554	SAR1811	SAS1659	SAV1733	MW1676	acs	acetyl-CoA synthetase	Energy metabolism	Fermentation	-6.27	-29.03	-14.26	-8.30
WAN014FXH_at	35.0	11.3	2.7	6.4	13.6	SA0154	SA0162	SAR0169	SAS0142	SAV0167	MW0142	aldA1	aldehyde dehydrogenase	Energy metabolism	Fermentation	-3.10	-13.18	-5.48	-2.58
WAN014GH9_at	55.0	356.4	4.5	4.1	10.1	SA0204	SA0218	SAR0217	SAS0201	SAV0226	MW0201	pflB	formate acetyltransferase	Energy metabolism	Fermentation	6.48	-12.12	-13.37	-5.45
WAN014HG8_at	23.3	1.8	2.8	4.0	7.0	SA0215	SA0227	SAR0227	SAS0211	SAV0235	MW0211	fadX	propionate CoA-transferase, putative	Energy metabolism	Fermentation	-12.92	-8.20	-5.79	-3.35
WAN014GHF_at	103.7	642.7	22.5	20.5	35.4	SA0205	SA0219	SAR0218	SAS0202	SAV0227	MW0202	pflA	pyruvate formate-lyase-activating enzyme	Energy metabolism	Fermentation	6.20	-4.62	-5.06	-2.93
WAN014G8Z_at	12.8	14.9	3.6	2.8	2.7	SA2617	SA2394	SAR2679	SAS2486	SAV2601	MW2520	budA2	alpha-acetolactate decarboxylase, putative	Energy metabolism	Fermentation	1.16	-3.53	-4.62	-4.67
WAN014I3R_at	50.7	20.9	19.9	14.2	17.6	SA2178	SA1989	SAR2278	SAS2088	SAV2187	MW2113		alcohol dehydrogenase, zinc-containing	Energy metabolism	Fermentation	-2.43	-2.54	-3.58	-2.88
WAN014G2T_at	20.6	14.9	8.2	6.2	7.5	SA1984	SA1736	SAR2013	SAS1844	SAV1920	MW1861	aldH	aldehyde dehydrogenase	Energy metabolism	Fermentation	-1.39	-2.50	-3.33	-2.75
WAN014I5H_at	19.6	23.2	9.3	9.7	11.0	SA1308	SA1131	SAR1265	SAS1223	SAV1289	MW1172	porA	pyruvate ferredoxin oxidoreductase, alpha subunit, putative	Energy metabolism	Fermentation	1.18	-2.11	-2.02	-1.78
WAN014I3P_at	2.7	2.0	1.3	1.4	1.6	SA2177	SA1988	SAR2277	SAS2087	SAV2186	MW2112		alcohol dehydrogenase, zinc-containing	Energy metabolism	Fermentation	-1.34	-2.04	-1.86	-1.67
WAN014HDS_at	114.6	6.5	4.2	11.5	20.1	SA1734	SA1510	SAR1766	SAS1615	SAV1687	MW1630	gapA2	glyceraldehyde 3-phosphate dehydrogenase	Energy metabolism	Glycolysis/gluconeogenesis	-17.50	-27.02	-9.94	-5.71
WAN014FZX_at	74.8	20.5	6.1	11.6	20.9	SA1838	SA1609	SAR1871	SAS1712	SAV1791	MW1729	pckA	phosphoenolpyruvate carboxykinase (ATP)	Energy metabolism	Glycolysis/gluconeogenesis	-3.66	-12.35	-6.42	-3.58
WAN014G92_at	54.5	45.9	4.6	3.9	3.7	SA2618	SA2395	SAR2680	SAS2487	SAV2602	MW2521	ldh	L-lactate dehydrogenase	Energy metabolism	Glycolysis/gluconeogenesis	-1.19	-11.85	-13.96	-14.76
WAN014GCM_at	11.3	11.2	1.1	1.6	2.0	SA2527	SA2304					fbp	fructose-1,6-bisphosphatase, putative	Energy metabolism	Glycolysis/gluconeogenesis	-1.01	-10.63	-6.97	-5.52
WAN014GEN_at	29.1	13.0	6.2	5.6	7.6	SA2622	SA2399	SAR2684	SAS2491	SAV2606	MW2525	fda	fructose-bisphosphate aldolase, class I	Energy metabolism	Glycolysis/gluconeogenesis	-2.24	-4.66	-5.20	-3.81
WAN014HKV_at	24.3	55.7	8.9	7.9	6.9	SA0966	SA0823	SAR0924	SAS0832	SAV0962	MW0844	pgi	glucose-6-phosphate isomerase	Energy metabolism	Glycolysis/gluconeogenesis	2.29	-2.72	-3.07	-3.50
WAN014FZ2_at	16.4	18.6	7.3	7.4	6.7	SA2415	SA2204	SAR2506	SAS2307	SAV2416	MW2339	gpm	phosphoglycerate mutase	Energy metabolism	Glycolysis/gluconeogenesis	1.14	-2.26	-2.23	-2.46
WAN014HAE_at	2.8	2.5	1.4	1.1	2.1	SA0758	SA0654	SAR0752	SAS0664	SAV0699	MW0661	fruB	1-phosphofructokinase	Energy metabolism	Glycolysis/gluconeogenesis	-1.10	-2.04	-2.58	-1.31
WAN014H6B_at	24.0	21.4	12.0	9.2	10.8	SA1604	SA1377	SAR1624	SAS1485	SAV1547	MW1499	glkA	glucokinase	Energy metabolism	Glycolysis/gluconeogenesis	-1.12	-2.00	-2.62	-2.23
WAN014FYZ_at	14.8	7.6	1.2	1.3	1.5	SA1785	SA1556	SAR1813	SAS1661	SAV1735	MW1678	acuC	acetoin utilization protein AcuC	Energy metabolism	Other	-1.94	-12.77	-11.24	-10.06
WAN014I1K_at	69.8	65.1	6.1	4.8	9.5	SA2128	SA1938	SAR2224	SAS2039	SAV2136	MW2060	deoC	deoxyribose-phosphate aldolase	Energy metabolism	Other	-1.07	-11.38	-14.63	-7.33
WAN014FYX_at	7.5	3.8	1.0	0.9	1.5	SA1784	SA1555	SAR1812	SAS1660	SAV1734	MW1677	acuA	acetoin utilization protein AcuA	Energy metabolism	Other	-1.99	-7.65	-8.37	-5.08

Systematic	Stat.	Log	30'	120'	360'	COL	N315	MRSA	MSSA	Mu50	MW2	GENE NAME	Protein Function	TIGR Main Role	TIGR Sub Role	Fold change vs stationary phase			
																LB	30' in vivo	120' in vivo	360' in vivo
WAN014H86_at	5.2	6.3	1.0	0.9	1.4	SA0708	SA0606	SAR0661	SAS0616	SAV0651	MW0613	dhaL	dihydroxyacetone kinase, L subunit	Energy metabolism	Other	1.23	-5.39	-5.58	-3.65
WAN014H84_at	6.4	8.4	1.2	1.4	2.2	SA0707	SA0605	SAR0660	SAS0615	SAV0650	MW0612	dhaK	dihydroxyacetone kinase family protein	Energy metabolism	Other	1.30	-5.21	-4.69	-2.89
WAN014G98_at	77.8	34.6	16.1	11.9	13.3	SA2624	SA2402	SAR2687	SAS2494	SAV2609	MW2528	acs	acetyl-CoA synthetase, putative	Energy metabolism	Other	-2.25	-4.82	-6.52	-5.84
WAN014IUF_at	61.4	57.9	14.2	10.6	15.1	SA0123	SA0133					deoC	deoxyribose-phosphate aldolase	Energy metabolism	Other	-1.06	-4.31	-5.81	-4.07
WAN014HLE_at	44.1	33.2	16.7	13.3	16.4	SA0973	SA0829	SAR0930	SAS0838	SAV0968	MW0850		fumarylacetoacetate hydrolase family protein	Energy metabolism	Other	-1.33	-2.65	-3.31	-2.69
WAN014I62_at	8.7	9.1	3.8	6.4	7.5	SA1320	SA1141	SAR1275	SAS1233	SAV1301	MW1183	gplK	glycerol kinase	Energy metabolism	Other	1.05	-2.32	-1.36	-1.16
WAN014FVO_at	6.9	13.7	3.0	2.8	2.3	SA2329	SA2127	SAR2422	SAS2228	SAV2336	MW2256	rpiA	ribose 5-phosphate isomerase	Energy metabolism	Pentose phosphate pathway	1.99	-2.30	-2.46	-3.03
WAN014FXJ_at	56.7	58.2	28.3	29.3	26.7	SA1549	SA1336	SAR1582	SAS1445	SAV1505	MW1459	zwf	glucose-6-phosphate 1-dehydrogenase	Energy metabolism	Pentose phosphate pathway	1.03	-2.01	-1.94	-2.12
WAN014H33_at	5.7	9.3	1.1	1.3	1.4	SA0598	SA0510	SAR0557	SAS0510	SAV0552	MW0507	araB	L-ribulokinase	Energy metabolism	Sugars	1.64	-5.06	-4.42	-4.18
WAN014G5Z_at	4.2		1.1	0.7	1.7	SA2149	SA1963	SAR2247	SAS2060	SAV2159	MW2085	mtlD	mannitol-1-phosphate 5-dehydrogenase	Energy metabolism	Sugars		-3.92	-5.82	-2.56
WAN014H3R_at	25.5	8.6	6.8	4.9	6.5	SA0617	SA0528	SAR0574	SAS0528	SAV0570	MW0525	hxlA	hexulose-6-phosphate synthase, putative	Energy metabolism	Sugars	-2.97	-3.72	-5.18	-3.89
WAN014GC6_at	5.9	12.2	2.0	2.0	1.9	SA2501	SA2279	SAR2576	SAS2378	SAV2491	MW2411	manB	phosphoglucomutase/phosphomannomutase family protein	Energy metabolism	Sugars	2.08	-2.95	-2.98	-3.18
WAN014HVP_at	6.4	6.6	2.3	2.3	2.6	SA2028	SA1845	SAR2127	SAS1945	SAV2040	MW1964	frk	fructokinase, putative	Energy metabolism	Sugars	1.04	-2.79	-2.81	-2.49
WAN014GY5_at	51.4	27.0	4.2	4.0	5.6	SA1448	SA1244	SAR1424	SAS1355	SAV1412	MW1302	sucB	2-oxoglutarate dehydrogenase, E2 component, dihydrolipoamide succinyltransferase	Energy metabolism	TCA cycle	-1.91	-12.25	-12.79	-9.25
WAN014GY8_at	10.5	6.2	1.0	0.8	1.5	SA1449	odhA,	SAR1425	SAS1356	SAV1413	MW1303	sucA	2-oxoglutarate dehydrogenase, E1 component	Energy metabolism	TCA cycle	-1.70	-10.76	-12.65	-6.87
WAN014HO7_at	27.1	18.0	2.9	3.6	3.6	SA1908	SA1669	SAR1942	SAS1772	SAV1851	MW1792	fumC	fumarate hydratase, class II	Energy metabolism	TCA cycle	-1.51	-9.48	-7.60	-7.60
WAN014HWR_at	33.9	18.7	4.5	6.8	6.3	SA1158	SA0994	SAR1120	SAS1081	SAV1147	MW1030	sdhC	succinate dehydrogenase, cytochrome b558 subunit	Energy metabolism	TCA cycle	-1.81	-7.46	-4.96	-5.35
WAN014HWT_at	120.3	64.3	17.1	27.3	33.0	SA1159	SA0995	SAR1121	SAS1082	SAV1148	MW1031	sdhA	succinate dehydrogenase, flavoprotein subunit	Energy metabolism	TCA cycle	-1.87	-7.04	-4.42	-3.64
WAN014FWY_at	62.3	31.2	10.8	10.3	17.8	SA2362	SA2155	SAR2454	SAS2256	SAV2365	MW2286	mqo	malate:quinone oxidoreductase	Energy metabolism	TCA cycle	-2.00	-5.79	-6.06	-3.49
WAN014HWV_at	92.5	50.4	18.6	21.6	28.5	SA1160	SA0996	SAR1122	SAS1083	SAV1149	MW1032	sdhB	succinate dehydrogenase, iron-sulfur protein	Energy metabolism	TCA cycle	-1.83	-4.97	-4.28	-3.25
WAN014GY9_at	7.7	5.1	1.6	1.3	2.2		SA1245	SAR1425	SAS1356	SAV1413	MW1303	sucA	2-oxoglutarate dehydrogenase, E1 component	Energy metabolism	TCA cycle	-1.52	-4.75	-5.99	-3.55
WAN014I2Q_at	67.6	71.0	16.3	15.2	31.9	SA1262	SA1088	SAR1221	SAS1179	SAV1245	MW1128	sucC	succinyl-CoA synthase, beta subunit	Energy metabolism	TCA cycle	1.05	-4.15	-4.46	-2.12
WAN014HE7_at	19.5	8.5	5.0	6.9	12.3	SA1742	SA1518	SAR1774	SAS1623	SAV1695	MW1639	gltA	citrate synthase	Energy metabolism	TCA cycle	-2.28	-3.90	-2.83	-1.58
WAN014HE5_at	52.1	18.2	14.6	19.5	40.0	SA1741	SA1517	SAR1773	SAS1622	SAV1694	MW1638	icd	isocitrate dehydrogenase, NADP-dependent	Energy metabolism	TCA cycle	-2.87	-3.56	-2.67	-1.30
WAN014FUH_at	68.6	51.3	19.5	24.9	54.0	SA1385	SA1184	SAR1362	SAS1289	SAV1350	MW1237		aconitate hydratase	Energy metabolism	TCA cycle	-1.34	-3.51	-2.76	-1.27
WAN014I2V_at	142.6	114.4	60.0	62.1	118.0	SA1263	SA1089	SAR1222	SAS1180	SAV1246	MW1129	sucD	succinyl-CoA synthase, alpha subunit	Energy metabolism	TCA cycle	-1.25	-2.38	-2.30	-1.21
WAN014HDO_at	11.0	1.8	1.2	1.1	2.5	SA0211	SA0223	SAR0223	SAS0207	SAV0231	MW0207	atoB	acetyl-CoA acetyltransferase	Fatty acid and phospholipid metabolism	Biosynthesis	-6.12	-9.05	-9.86	-4.45
WAN014H7L_at	7.8	7.1	3.1	2.9	3.5	SA1626	SA1398	SAR1646	SAS1507	SAV1569	MW1521	dgkA2	diacylglycerol kinase, putative	Fatty acid and phospholipid metabolism	Biosynthesis	-1.10	-2.53	-2.71	-2.24
WAN014G2P_at	4.3	1.4	2.0	1.5	1.5	SA2482	SA2260			SAV2472		fabG	3-oxoacyl-(acyl carrier protein) reductase, authentic point mutation	Fatty acid and phospholipid metabolism	Biosynthesis	-3.00	-2.20	-2.79	-2.78
WAN014GCV_at	128.3	18.0	2.1	2.3	4.5	SA2694	SA2463	SAR2753	SAS2556	SAV2671	MW2590	lip	lipase	Fatty acid and phospholipid metabolism	Degradation	-7.13	-61.82	-56.88	-28.36
WAN014HEU_at	9.9		0.4	0.3	2.0	SA0213	SA0225	SAR0225	SAS0209	SAV0233	MW0209	fadD	acyl-CoA dehydrogenase family protein	Fatty acid and phospholipid metabolism	Degradation		-23.28	-33.01	-5.00
WAN014HE9_at	6.2		0.8		2.6	SA0212	SA0224	SAR0224	SAS0208	SAV0232	MW0208	fadB	3-hydroxyacyl-CoA dehydrogenase family protein	Fatty acid and phospholipid metabolism	Degradation		-8.00		-2.40
WAN014HFF_at	4.2		0.9	0.7	1.5	SA0214	SA0226	SAR0226	SAS0210	SAV0234	MW0210	fadE	long-chain-fatty-acid--CoA ligase, putative	Fatty acid and phospholipid metabolism	Degradation		-4.74	-5.64	-2.89
WAN014G5V_at	29.5	24.0	8.4	7.3	9.9	SA2549	SA2323	SAR2615	SAS2421	SAV2535	MW2456		esterase, putative	Fatty acid and phospholipid metabolism	Degradation	-1.22	-3.50	-4.01	-2.97
WAN014H5U_at	2.1	1.4	0.7	0.7	0.9	SA0078	SA0091	SAR0105		SAV0095	MW0070	plc	1-phosphatidylinositol phosphodiesterase precursor	Fatty acid and phospholipid metabolism	Degradation	-1.45	-3.10	-3.05	-2.29
WAN014FWC_at	12.4	19.0	4.2	4.9	3.9	SA2345	SA2140	SAR2435	SAS2241	SAV2350	MW2271		esterase, putative	Fatty acid and phospholipid metabolism	Degradation	1.54	-2.93	-2.54	-3.14
WAN014HR1_at	65.7	47.7	27.1	23.3	32.3	SA1054	SA0898	SAR1019	SAS0981	SAV1045	MW0929	menB	enoyl-CoA hydratase/isomerase family protein	Fatty acid and phospholipid metabolism	Degradation	-1.38	-2.42	-2.82	-2.03
WAN014HIP_at	29.7	15.3	14.9	12.4	10.6	SA1814	SA1584	SAR1848	SAS1689	SAV1765	MW1706		lysophospholipase, putative	Fatty acid and phospholipid metabolism	Degradation	-1.94	-1.99	-2.40	-2.81
WAN014FWG_at	9.0	6.2	2.9	2.2	2.7	SA1439	SA1236	SAR1416	SAS1345	SAV1404	MW1292	acyP	Acylphosphatase	Fatty acid and phospholipid metabolism	Other	-1.46	-3.14	-4.14	-3.28
WAN014H41_at	2.1	1.4	0.9	1.1	1.0	SA0621	SA0533	SAR0580	SAS0533	SAV0575	MW0530	vraA	substrate--CoA ligase, putative	Fatty acid and phospholipid metabolism	Other	-1.46	-2.27	-1.86	-2.03
WAN014G5N_at	18.4	4.4	0.6	0.4	1.3	SA0176	SA0184	SAR0191	SAS0165	SAV0190	MW0164		conserved hypothetical protein	Hypothetical proteins	Conserved	-4.23	-33.30	-48.05	-13.90
WAN014G64_at	45.4	14.4	1.8	5.2	6.3	SA2163	SAS074	SAR2264	SAS2074	SAV2173	MW2099		conserved hypothetical protein	Hypothetical proteins	Conserved	-3.16	-24.88	-8.81	-7.24
WAN014GNW_at	27.2	5.1	1.3	2.6	5.7	SA0768	SA0663	SAR0761	SAS0673	SAV0708	MW0670	saeP	hypothetical protein	Hypothetical proteins	Conserved	-5.30	-20.43	-10.54	-4.74
WAN014GNU_at	46.0	9.3	3.2	6.5	11.3	SA0767	SA0662	SAR0760	SAS0672			saeQ	conserved hypothetical protein	Hypothetical proteins	Conserved	-4.92	-14.45	-7.07	-4.08
WAN014H35_at	31.5	24.4	3.2	4.2	6.2	SA0599	SA0511	SAR0558	SAS0511	SAV0553	MW0508	wcaG	conserved hypothetical protein	Hypothetical proteins	Conserved	-1.29	-9.84	-7.52	-5.08
WAN014G02_at	25.7	16.1	3.1	3.6	5.6	SA1846	SA1617						hypothetical protein	Hypothetical proteins	Conserved	-1.59	-8.17	-7.16	-4.58
WAN014I1I_at	91.3	75.3	11.5	10.5	13.1	SA2127	SA1937	SAR2223	SAS2038	SAV2135	MW2059		conserved hypothetical protein	Hypothetical proteins	Conserved	-1.21	-7.93	-8.67	-6.98
WAN014G8L_at	12.6	1.1	1.6	1.3	1.4	SA2605	SA2374	SAR2668	SAS2474	SAV2588	MW2508		conserved hypothetical protein	Hypothetical proteins	Conserved	-11.06	-7.84	-9.74	-8.94
WAN01C3NL_at	5.5	1.4	0.9	1.1	1.7		#N/A						hypothetical protein	Hypothetical proteins	Conserved	-3.90	-6.26	-5.01	-3.29
WAN014GVK_at	78.4	10.2	13.4	10.0	11.1	SA1226	SA1057	SAR1190	SAS1148	SAV1214	MW1097		conserved hypothetical protein	Hypothetical proteins	Conserved	-7.67	-5.84	-7.87	-7.04
WAN014G7F_at	11.3		2.0	0.9	1.1	SA2580	SA2352	SAR2647	SAS2451	SAV2565	MW2486	crtO	staphyloxanthine biosynthesis (ORF 3)	Hypothetical proteins	Conserved		-5.75	-12.34	-10.54
WAN014I1X_at	20.4		3.7	2.1	2.6	SA2136	SA1946	SAR2232	SAS2047	SAV2144	MW2068		conserved hypothetical protein	Hypothetical proteins	Conserved		-5.51	-9.96	-7.93
WAN014GOR_at	20.8	10.1	3.9	3.6	4.2	SA0003	SA0003	SAR0003	SAS0003	SAV0003	MW0003		conserved hypothetical protein	Hypothetical proteins	Conserved	-2.05	-5.39	-5.78	-4.92
WAN014G99_at	29.6	1.0	5.6	3.3	4.2	SA2625	SA2403	SAR2688	SAS2495	SAV2610	MW2529		conserved hypothetical protein	Hypothetical proteins	Conserved	-29.58	-5.30	-8.88	-7.00
WAN014I1P_at	4.9	5.5	1.0	0.9	0.7	SA2132	SA1942	SAR2228	SAS2043	SAV2140	MW2064		conserved hypothetical protein	Hypothetical proteins	Conserved	1.12	-5.12	-5.26	-7.21
WAN014H1H_at	17.4	14.2	3.5	3.9	5.8	SA0568	SA0481	SAR0526	SAS0480	SAV0523	MW0478		conserved hypothetical protein	Hypothetical proteins	Conserved	-1.23	-5.02	-4.50	-3.02
WAN014GUA_at	11.7	16.1	2.5	2.3	2.8	SA1115	SA0957	SAR1080	SAS1041	SAV1106	MW0989		conserved hypothetical protein	Hypothetical proteins	Conserved	1.37	-4.78	-5.04	-4.19
WAN014GUH_at	9.8	2.4	2.1	1.5	2.9	SA0409	SA0327	SAR0335	SAS0315	SAV0339	MW0315		conserved hypothetical protein	Hypothetical proteins	Conserved	-4.10	-4.60	-6.53	-3.35
WAN014H7X_at	58.0	21.1	13.2	10.5	18.1	SA1631	SA1403	SAR1651	SAS1512	SAV1574	MW1526		conserved hypothetical protein	Hypothetical proteins	Conserved	-2.75	-4.39	-5.53	-3.20
WAN014G10_at	19.8	0.9	4.5	2.7	4.5	SA2461	SA2242		SAS2345	SAV2453	MW2377	ybbM	conserved hypothetical protein TIGR00245	Hypothetical proteins	Conserved	-21.27	-4.38	-7.45	-4.38
WAN014H1E_at	12.9	4.7	3.1	5.7	6.9	SA0565	SA0478	SAR0523	SAS0477	SAV0520	MW0475	pdxT	conserved hypothetical protein	Hypothetical proteins	Conserved	-2.73	-4.20	-2.29	-1.86
WAN014HIM_at	29.6	7.6	7.1	5.7	4.8	SA0922	SA0781	SAR0883	SAS0791	SAV0920	MW0803		conserved hypothetical protein	Hypothetical proteins	Conserved	-3.88	-4.18	-5.22	-6.15
WAN014GE3_at	136.6	6.9	33.2	19.7	31.9	SA2723	SA2491	SAR2780	SAS2584	SAV2700	MW2619		conserved hypothetical protein	Hypothetical proteins	Conserved	-19.88	-4.11	-6.93	-4.28
WAN014HES_at	78.4	12.8	19.3	11.0	12.2	SA1754	SA1529	SAR1785	SAS1634	SAV1707	MW1650		conserved hypothetical protein, authentic frameshift	Hypothetical proteins	Conserved	-6.10	-4.06	-7.12	-6.42
WAN014H7V_at	89.0	36.1	22.0	15.6	30.2	SA1630	SA1402	SAR1650	SAS1511	SAV1573	MW1525		conserved hypothetical protein	Hypothetical proteins	Conserved	-2.47	-4.04	-5.70	-2.94
WAN01BQKQ_at	29.8	9.9	7.6	5.0	6.3	SA2300	SA2101	SAR2392	SAS2200	SAV2308	MW2228		conserved hypothetical protein	Hypothetical proteins	Conserved	-3.00	-3.91	-5.99	-4.75
WAN014H5A_at	2.0	2.0	0.5			SA0648	SA0555						conserved hypothetical protein	Hypothetical proteins	Conserved	-1.01	-3.74		
WAN014H56_at	6.6	7.7	1.9	1.7	1.7	SA0647			SAS0559		MW0555		conserved hypothetical protein	Hypothetical proteins	Conserved	1.17	-3.50	-3.98	-3.87
WAN014HX8_at	97.2	54.4	27.8	25.7	18.4	SA2059	SAS067	SAR2157	SAS1974	SAV2069	MW1993		conserved hypothetical protein	Hypothetical proteins	Conserved	-1.79	-3.49	-3.79	-5.28
WAN014GGI_at	4.1	4.1	1.2	1.0	1.3		SA0023	SAR0024	SAS0024	SAV0024	MW0024		conserved hypothetical protein TIGR00246	Hypothetical proteins	Conserved	1.00	-3.47	-4.34	-3.26
WAN014HOK_at	21.4	15.1	6.2	5.6	6.7	SA1020	SA0873	SAR0985	SAS0884	SAV1015	MW0896		conserved hypothetical protein	Hypothetical proteins	Conserved	-1.42	-3.44	-3.86	-3.20
WAN014IOP_x_at	8.8	10.5	2.6	2.8	3.4			SAR0397	SAS0356	SAV0379	MW0355		conserved hypothetical protein	Hypothetical proteins	Conserved	1.19	-3.42	-3.12	-2.61
WAN014FW3_at	4.0	2.8	1.2	1.0	1.3	SA2338	SA2133						conserved hypothetical protein, authentic frameshift	Hypothetical proteins	Conserved	-1.40	-3.39	-4.14	-2.99
WAN014I0M_at	7.2	2.6	2.1	1.8	1.4	SA1219	SA1050	SAR1183	SAS1141	SAV1207	MW1090		conserved hypothetical protein	Hypothetical proteins	Conserved	-2.81	-3.39	-4.02	-5.00
WAN014H98_at	5.6	4.4	1.7	1.5	2.2	SA0730	SA0626	SAR0682	SAS0636	SAV0671	MW0633		conserved hypothetical protein	Hypothetical proteins	Conserved	-1.29	-3.38	-3.72	-2.51
WAN014HTF_at	6.0	1.6	1.8	1.7	1.5	SA1980	SA1733	SAR2010	SAS1841	SAV1917	MW1858		conserved hypothetical protein	Hypothetical proteins	Conserved	-3.83	-3.36	-3.58	-3.95
WAN014HDH_at	32.3	5.8	9.7	6.0	6.5	SA0831	SA0721	SAR0821	SAS0731	SAV0766	MW0728		conserved hypothetical protein	Hypothetical proteins	Conserved	-5.60	-3.33	-5.34	-4.99
WAN014GVL_at	151.4	4.6	45.8	20.7	23.2	SA0444	SA0359	SAR0390	SAS0349	SAV0372	MW0347		conserved hypothetical protein	Hypothetical proteins	Conserved	-32.99	-3.31	-7.31	-6.53
WAN014H7D_at	3.7	3.2	1.2	1.1	1.4	SA1621	SA1393			SAV1564	MW1516		conserved hypothetical protein	Hypothetical proteins	Conserved	-1.17	-3.17	-3.55	-2.75
WAN014I2H_at	10.9	10.0	3.4	2.9	3.1	SA2153	SA1967	SAR2254	SAS2065	SAV2163	MW2090		conserved hypothetical protein TIGR00159	Hypothetical proteins	Conserved	-1.08	-3.16	-3.72	-3.48
WAN014HEQ_at	12.4	5.9	4.0	3.3	2.8	SA1752	SA1527	SAR1783	SAS1632	SAV1705	MW1648		conserved hypothetical protein	Hypothetical proteins	Conserved	-2.11	-3.12	-3.79	-4.38
WAN014H7A_at	6.3	4.6	2.1	1.8	1.7	SA1620	SA1392	SAR1640	SAS1501	SAV1563	MW1515		conserved hypothetical protein	Hypothetical proteins	Conserved	-1.37	-2.96	-3.47	-3.71
WAN014H88_at	2.5	2.5	0.9	0.7	0.6	SA0709	SA0607	SAR0662	SAS0617	SAV0652	MW0614		conserved hypothetical protein	Hypothetical proteins	Conserved	1.00	-2.92	-3.41	-4.42
WAN014GN9_at	32.8	4.9	11.9	8.5	10.1	SA0741	SA0636	SAR0734	SAS0646	SAV0681	MW0643		conserved hypothetical protein	Hypothetical proteins	Conserved	-6.67	-2.75	-3.87	-3.23
WAN014G8G_at	10.3	12.4	3.8	2.7	1.8	SA0449	SA0363	SAR0396	SAS0355	SAV0378	MW0354		conserved hypothetical protein	Hypothetical proteins	Conserved	1.21	-2.74	-3.78	-5.58
WAN014GHJ_x_at	10.5	13.0	3.9	3.8	4.9		SAS024		SAS0843	SAV0973	MW0855			Hypothetical proteins	Conserved	1.24	-2.71	-2.73	-2.16
WAN014GGF_at	1.3	1.1	0.5			SA0198	SA0212	SAR0211	SAS0195	SAV0219	MW0195		conserved hypothetical protein	Hypothetical proteins	Conserved	-1.22	-2.71		
WAN014GDT_at	11.1	8.7	4.1	3.5	4.7	SA2589	SA2360	SAR2654	SAS2460	SAV2573	MW2494		conserved hypothetical protein	Hypothetical proteins	Conserved	-1.28	-2.70	-3.15	-2.37
WAN014GOH_at	45.2	1.2	16.9	9.3	7.6	SA0787	SA0681	SAR0780	SAS0691	SAV0726	MW0688		conserved hypothetical protein TIGR00147	Hypothetical proteins	Conserved	-36.35	-2.68	-4.85	-5.98
WAN01BQUF_at	33.7	36.4	12.7	11.7	8.7			SAR2706a	SAS2513a		MW2548		conserved hypothetical protein	Hypothetical proteins	Conserved	1.08	-2.65	-2.88	-3.85
WAN014HZD_at	9.2	9.0	3.6	3.3	3.3	SA1200	SA1030	SAR1163	SAS1121	SAV1187	MW1070		conserved hypothetical protein TIGR00726	Hypothetical proteins	Conserved	-1.03	-2.59	-2.77	-2.83
WAN014GD2_at	16.4	29.1	6.4	8.3	7.3	SA2548	SA2322	SAR2614	SAS2420	SAV2534	MW2455		conserved hypothetical protein	Hypothetical proteins	Conserved	1.77	-2.58	-1.98	-2.26
WAN014FV9_at	10.9	6.7	4.2	3.6	4.4	SA1402	SA1198	SAR1379	SAS1306	SAV1366	MW1253		conserved hypothetical protein	Hypothetical proteins	Conserved	-1.61	-2.57	-3.01	-2.50
WAN014H7N_at	6.2	5.2	2.4	2.1	2.6	SA1627	SA1399	SAR1647	SAS1508	SAV1570	MW1522		conserved hypothetical protein TIGR00043	Hypothetical proteins	Conserved	-1.18	-2.55	-2.93	-2.35
WAN014G8C_at	3.8	3.7	1.5	1.2	1.6	SA2601	SA2371	SAR2665	SAS2471	SAV2585	MW2505		conserved hypothetical protein	Hypothetical proteins	Conserved	-1.05	-2.51	-3.14	-2.35
WAN01BX7O_at	2.6	3.6	1.0	0.9	1.0				SAS0268		MW0268		conserved hypothetical protein	Hypothetical proteins	Conserved	1.39	-2.49	-2.85	-2.54
WAN014I3F_at	3.1	1.4	1.3	1.6	1.4	SA0316	SA0308	SAR0316	SAS0296	SAV0319	MW0296		conserved hypothetical protein	Hypothetical proteins	Conserved	-2.26	-2.48	-1.94	-2.33
WAN014GSH_at	12.6	6.5	5.1	6.3	6.8		SA2401						conserved hypothetical protein	Hypothetical proteins	Conserved	-1.95	-2.47	-2.01	-1.85
WAN014HK8_at	36.1	22.5	14.7	17.8	21.0	SA0943	SA0801	SAR0902	SAS0810	SAV0940	MW0822		conserved hypothetical protein	Hypothetical proteins	Conserved	-1.60	-2.46	-2.03	-1.72
WAN01BQ3K_at	1.5		0.6	0.6				SAR1704	SAS1560	SAV1624	MW1574		conserved hypothetical protein	Hypothetical proteins	Conserved		-2.45	-2.53	
WAN014FTK_at	2.7	1.3	1.1	1.5	1.4				SAS0377				conserved hypothetical protein	Hypothetical proteins	Conserved	-1.99	-2.43	-1.80	-1.93

																Fold change vs stationary phase			
**Systematic**	**Stat.**	**Log**	**30'**	**120'**	**360'**	**COL**	**N315**	**MRSA**	**MSSA**	**Mu50**	**MW2**	**GENE NAME**	**Protein Function**	**TIGR Main Role**	**TIGR Sub Role**	**LB**	**30' in vivo**	**120' in vivo**	**360' in vivo**
WAN014HGE_at	136.3	20.0	56.8	34.9	33.2	SA1789	SA1560	SAR1817	SAS1665	SAV1739	MW1682		conserved hypothetical protein	Hypothetical proteins	Conserved	-6.83	-2.40	-3.91	-4.11
WAN014I2F_at	2.1	2.1	0.9	0.7	0.8	SA2152	SA1966	SAR2253	SAS2064	SAV2162	MW2089		conserved hypothetical protein	Hypothetical proteins	Conserved	1.03	-2.37	-2.80	-2.47
WAN014GMU_at	3.6	2.6	1.5	1.7	1.4		SAS041	SAR1333	SAS1263	SAV1323	MW1210		conserved hypothetical protein	Hypothetical proteins	Conserved	-1.40	-2.33	-2.17	-2.56
WAN014GN8_at	203.2	145.7	88.7	75.0	115.6	SA0271	SA0271	SAR0279	SAS0258	SAV0282	MW0258	esxA	conserved hypothetical protein	Hypothetical proteins	Conserved	-1.39	-2.29	-2.71	-1.76
WAN014GCE_at	5.2	1.6	2.3	1.8	1.4	SA2682	SA2452	SAR2740	SAS2545	SAV2659	MW2579		conserved hypothetical protein	Hypothetical proteins	Conserved	-3.29	-2.27	-2.95	-3.80
WAN014IXM_x_at	4.9	2.8	2.2	1.8	2.9		SA1788						conserved hypothetical protein	Hypothetical proteins	Conserved	-1.72	-2.25	-2.72	-1.65
WAN014GVP_at	125.4	10.0	56.4	42.1	76.5	SA0446	SA0360	SAR0392	SAS0351	SAV0374	MW0349		conserved hypothetical protein	Hypothetical proteins	Conserved	-12.54	-2.23	-2.98	-1.64
WAN014GHW_at	2498.3	1570.8	1136.4	1326.7	1365.3		SAS050	SAR1709	SAS1565	SAV1629	MW1579		conserved hypothetical protein	Hypothetical proteins	Conserved	-1.59	-2.20	-1.88	-1.83
WAN014FWI_at	5.4	3.1	2.4	1.9	1.7	SA1440	SA1237	SAR1417	SAS1346	SAV1405	MW1293		conserved hypothetical protein	Hypothetical proteins	Conserved	-1.72	-2.20	-2.88	-3.11
WAN014HNF_at	15.6	21.3	7.1	6.1	6.4	SA1899	SA1661	SAR1934	SAS1764	SAV1843	MW1784		conserved hypothetical protein	Hypothetical proteins	Conserved	1.37	-2.19	-2.57	-2.45
WAN014FYK_at	5.2	3.8	2.4	2.2	2.2	SA2404	SA2195	SAR2497	SAS2298	SAV2407	MW2329		conserved hypothetical protein	Hypothetical proteins	Conserved	-1.39	-2.17	-2.37	-2.33
WAN014FVX_at	4.6	6.1	2.1	1.8	2.3	SA2334	SA2131	SAR2426	SAS2232	SAV2340	MW2260	natB	conserved hypothetical protein	Hypothetical proteins	Conserved	1.32	-2.17	-2.59	-2.03
WAN014G22_at	2.3	2.9	1.1	1.2	1.4	SA1947	SA1705	SAR1980	SAS1811	SAV1889	MW1829	ygaE	conserved hypothetical protein	Hypothetical proteins	Conserved	1.25	-2.16	-1.88	-1.69
WAN014HO1_at	9.4	14.6	4.4	4.3	4.1	SA1011	SA0865	SAR0974	SAS0876	SAV1007	MW0888	ppnK	conserved hypothetical protein	Hypothetical proteins	Conserved	1.55	-2.14	-2.17	-2.30
WAN014HWF_at	2.0	1.3	0.9	1.1	1.0			SAR1313					conserved hypothetical protein	Hypothetical proteins	Conserved	-1.48	-2.14	-1.77	-2.06
WAN014HHA_at	3.1	1.9	1.4	1.2	1.8		SA1796	SAR2087	SAS1908	SAV1987	MW1925		conserved hypothetical protein	Hypothetical proteins	Conserved	-1.59	-2.14	-2.47	-1.73
WAN014GYS_at	51.3	51.9	24.0	18.4	22.7	SA0521	SA0437	SAR0478	SAS0436	SAV0479	MW0434		conserved hypothetical protein TIGR00103	Hypothetical proteins	Conserved	1.01	-2.14	-2.78	-2.26
WAN014ILW_x_at	1.2	0.6	0.6	0.8	1.0	SA0286							conserved hypothetical protein	Hypothetical proteins	Conserved	-2.05	-2.13	-1.57	-1.24
WAN014G53_at	3.2	1.3	1.5	1.4	1.7	SA2529	SA2306						conserved hypothetical protein	Hypothetical proteins	Conserved	-2.48	-2.11	-2.31	-1.88
WAN014HNR_at	3.7	5.2	1.8	1.6	1.4	SA1009	SA0863	SAR0972	SAS0874	SAV1005	MW0886		conserved hypothetical protein	Hypothetical proteins	Conserved	1.39	-2.09	-2.34	-2.68
WAN014IOK_at	1.9	2.1	0.9	0.7						SAV2484			conserved hypothetical protein	Hypothetical proteins	Conserved	1.12	-2.08	-2.74	
WAN014HJK_at	1.2		0.6	0.5	0.9	SA0106	SA0118	SAR0125	SAS0096	SAV0122	MW0095		conserved hypothetical protein	Hypothetical proteins	Conserved		-2.08	-2.43	-1.43
WAN014HJD_at	1.5	1.6	0.7	0.8	1.2	SA0930	SA0789	SAR0890	SAS0798	SAV0928	MW0810		conserved hypothetical protein	Hypothetical proteins	Conserved	1.06	-2.07	-1.80	-1.23
WAN014FTH_at	1.5	1.2	0.7	0.6					SAS0370		MW0370		conserved hypothetical protein	Hypothetical proteins	Conserved	-1.28	-2.07	-2.60	
WAN014GE0_at	4.7	2.2	2.3	1.5	2.5	SA2596	SA2366	SAR2660	SAS2466	SAV2580	MW2500		conserved hypothetical protein	Hypothetical proteins	Conserved	-2.12	-2.06	-3.18	-1.84
WAN014G5X_at	22.1	35.4	10.7	9.2	7.8	SA2550	SA2324	SAR2616	SAS2422	SAV2536	MW2457		conserved hypothetical protein	Hypothetical proteins	Conserved	1.60	-2.06	-2.41	-2.85
WAN014IM3_x_at	1.1	0.9	0.5	0.3		SA0296	SA0289						conserved hypothetical protein	Hypothetical proteins	Conserved	-1.17	-2.03	-3.52	
WAN01BYX5_at	1.2	1.3	0.6						SAS2374		MW2407		conserved hypothetical protein	Hypothetical proteins	Conserved	1.05	-2.03		
WAN014GG0_at	2.5	2.0	1.2	1.3	1.5	SA0467	SA0381	SAR0421	SAS0383	SAV0421	MW0381		conserved hypothetical protein	Hypothetical proteins	Conserved	-1.27	-2.02	-1.91	-1.72
WAN014GS0_at	6.2	7.0	3.1	2.8	2.7	SA1006	SA0860	SAR0969	SAS0871	SAV1001	MW0883		conserved hypothetical protein	Hypothetical proteins	Conserved	1.12	-2.00	-2.20	-2.28
WAN014GZH_at	7.8	8.9	3.9	3.1	3.0	SA1467	SA1264	SAR1444	SAS1374	SAV1431			conserved hypothetical protein	Hypothetical proteins	Conserved	1.15	-1.98	-2.48	-2.60
WAN014G04_at	16.6	10.8	1.9	2.1	2.5	SA1847	SA1618						conserved domain protein	Hypothetical proteins	Domain	-1.53	-8.95	-7.85	-6.76
WAN014GNB_at	19.3	2.8	4.4	2.5	2.7	SA0742	SA0637	SAR0735	SAS0647	SAV0682	MW0644		conserved domain protein	Hypothetical proteins	Domain	-6.83	-4.33	-7.78	-7.23
WAN014G0J_at	27.6	2.6	1.2	1.2		SA1870			SAS1738		MW1757		hypothetical protein	Hypothetical proteins	Not Conserved	-10.73	-22.85	-23.80	
WAN014GGG_at	58.6	3.4	2.8	3.6	7.0	SA0480	SA0395	SAR0437	SAS0398		MW0396		hypothetical protein	Hypothetical proteins	Not Conserved	-17.22	-20.71	-16.40	-8.34
WAN01BQVN_at	8.9	2.2	0.5	0.7	1.3	SA2693							hypothetical protein	Hypothetical proteins	Not Conserved	-4.01	-17.44	-12.83	-6.79
WAN014FTI_at	16.0	10.6	1.9	1.8	2.8				SAS0371		MW0372		hypothetical protein	Hypothetical proteins	Not Conserved	-1.52	-8.64	-9.02	-5.67
WAN014GD0_at	257.9	28.7	34.6	27.1	37.8	SA0457	SA0372						hypothetical protein	Hypothetical proteins	Not Conserved	-8.99	-7.46	-9.53	-6.82
WAN014HPI_at	9.2	2.0	1.2	1.6	2.6	SA1033	SA0883	SAR0996	SAS0959	SAV1027	MW0907		hypothetical protein	Hypothetical proteins	Not Conserved	-4.67	-7.38	-5.63	-3.51
WAN014GPJ_at	5.3	8.7	0.8	0.9	1.2	SA0851	SA0739	SAR0839	SAS0749	SAV0808	MW0761		hypothetical protein	Hypothetical proteins	Not Conserved	1.62	-6.94	-5.75	-4.49
WAN014GSG_at	57.6	7.6	8.4	6.7	9.6	SA1046		SAR1012	SAS0974		MW0922		hypothetical protein	Hypothetical proteins	Not Conserved	-7.61	-6.88	-8.57	-5.97
WAN014G2Y_at	41.1	32.1	6.7	4.9	4.3	SA1992	SA1743	SAR2021	SAS1853	SAV1929	MW1870		hypothetical protein	Hypothetical proteins	Not Conserved	-1.28	-6.16	-8.31	-9.52
WAN014FX4_at	63.8	28.1	10.7	5.7	5.9	SA2365	SA2158						hypothetical protein	Hypothetical proteins	Not Conserved	-2.27	-5.95	-11.16	-10.84
WAN014HCH_at	9.0	2.3	1.6	1.1	1.2	SA0092	SA0105	SAR0112	SAS0083	SAV0109	MW0082		hypothetical protein	Hypothetical proteins	Not Conserved	-3.99	-5.63	-8.44	-7.47
WAN014GJQ_at	109.7	44.3	20.2	75.4	117.7	SA0625	SAS016	SAR0584	SAS0537	SAV0579	MW0534		hypothetical protein	Hypothetical proteins	Not Conserved	-2.48	-5.42	-1.45	1.07
WAN01BP6R_at	19.7	7.5	3.7	3.9	7.5	SA0490		SAR0449	SAS0407		MW0405		hypothetical protein	Hypothetical proteins	Not Conserved	-2.63	-5.27	-5.03	-2.61
WAN014GCG_at	11.6	5.4	2.3	2.0	2.2	SA0456	SA0371	SAR0404	SAS0362	SAV0386	MW0362		hypothetical protein	Hypothetical proteins	Not Conserved	-2.16	-5.01	-5.75	-5.32
WAN014I3J_at	60.8	1.3	12.8	6.6	7.8	SA2174	SA1985	SAR2274	SAS2084	SAV2183	MW2109		hypothetical protein	Hypothetical proteins	Not Conserved	-48.16	-4.75	-9.17	-7.82
WAN014HGA_x_at	14.3	1.8	3.0	2.0	2.5	SA1788							hypothetical protein	Hypothetical proteins	Not Conserved	-7.74	-4.69	-7.22	-5.71
WAN014H2X_at	2.8		0.6			SA0156	SA0164						hypothetical protein	Hypothetical proteins	Not Conserved		-4.46		
WAN01BQBV_at	24.2	8.6	5.7	6.6	5.8	SA1978							hypothetical protein	Hypothetical proteins	Not Conserved	-2.81	-4.28	-3.69	-4.14
WAN014GPH_at	9.2	16.9	2.2	1.9	2.1	SA0850							hypothetical protein	Hypothetical proteins	Not Conserved	1.84	-4.23	-4.78	-4.35
WAN014G5L_at	7.4	78.0	1.8	1.9	2.3	SA2138	SA1948	SAR2234	SAS2049	SAV2146	MW2070		hypothetical protein	Hypothetical proteins	Not Conserved	10.52	-4.16	-3.96	-3.21
WAN014H7T_at	69.9	34.9	16.8	12.6	18.8	SA1629	SA1401	SAR1649	SAS1510	SAV1572	MW1524		hypothetical protein	Hypothetical proteins	Not Conserved	-2.00	-4.15	-5.56	-3.71
WAN014IV8_at	231.3	123.3	56.1	63.9	47.2	SA1041	SA0889			SAV1036	MW0917		hypothetical protein	Hypothetical proteins	Not Conserved	-1.88	-4.12	-3.62	-4.90
WAN014HMN_at	5.6	4.9	1.4	1.6	1.4	SA0110	SA0121						hypothetical protein	Hypothetical proteins	Not Conserved	-1.14	-3.97	-3.53	-4.01
WAN014IV1_at	3.2		0.8	0.6	0.6	SA2720	SA2488						hypothetical protein	Hypothetical proteins	Not Conserved		-3.89	-5.21	-5.48
WAN014G3Q_at	16.3	13.0	4.2	4.4	4.4	SA2027							hypothetical protein	Hypothetical proteins	Not Conserved	-1.25	-3.85	-3.73	-3.73
WAN014GDE_at	5.3	3.3	1.4	3.3	4.6	SA2571	SA2343	SAR2636	SAS2442	SAV2556	MW2477		hypothetical protein	Hypothetical proteins	Not Conserved	-1.61	-3.77	-1.60	-1.17
WAN014GHE_at	3.9	0.9	1.1	0.7	1.9		SAS014			SAV0451			hypothetical protein	Hypothetical proteins	Not Conserved	-4.21	-3.60	-5.86	-2.02
WAN014GPD_at	7.2	12.2	2.0	2.6	2.2	SA0849	SA0738			SAV0807			hypothetical protein	Hypothetical proteins	Not Conserved	1.69	-3.54	-2.77	-3.21
WAN014HEW_at	51.3	23.2	14.6	19.8	28.3	SA1757							hypothetical protein	Hypothetical proteins	Not Conserved	-2.21	-3.52	-2.59	-1.81
WAN014ILB_at	2.8	3.8	0.8	1.0		SA2381	SA2171	SAR2470	SAS2273	SAV2383	MW2303		hypothetical protein	Hypothetical proteins	Not Conserved	1.38	-3.48	-2.67	
WAN014HU9_at	9.8	11.7	2.8	3.3	2.8	SA1113	SA0955	SAR1078	SAS1039	SAV1104	MW0987		hypothetical protein	Hypothetical proteins	Not Conserved	1.20	-3.45	-2.99	-3.46
WAN014HJE_at	8.6	1.8	2.5	2.1	1.9	SA1821	SA1590	SAR1854	SAS1695	SAV1772	MW1712		hypothetical protein	Hypothetical proteins	Not Conserved	-4.70	-3.42	-4.17	-4.58
WAN014I3L_at	259.0	3.6	77.0	38.1	45.4	SA2175	SA1986	SAR2275	SAS2085	SAV2184	MW2110		hypothetical protein	Hypothetical proteins	Not Conserved	-72.13	-3.36	-6.79	-5.71
WAN014G0W_at	387.0	91.1	119.2	86.9	80.0	SA1895	SA1657	SAR1930	SAS1760	SAV1839	MW1779		hypothetical protein	Hypothetical proteins	Not Conserved	-4.25	-3.25	-4.45	-4.84
WAN014GWA_at	5.7	4.3	1.8	1.7	1.5	SA1366	SA1168	SAR1342	SAS1272	SAV1332	MW1219		hypothetical protein	Hypothetical proteins	Not Conserved	-1.34	-3.23	-3.44	-3.71
WAN014G6J_at	20.7	11.7	6.7	6.0	8.1	SA2241	SA2049	SAR2337	SAS2143	SAV2252	MW2171		hypothetical protein	Hypothetical proteins	Not Conserved	-1.76	-3.11	-3.43	-2.56
WAN014HUI_at	53.3	22.1	17.2	27.2	40.2	SA1999		SAR2027a	SAS1859		MW1877		hypothetical protein	Hypothetical proteins	Not Conserved	-2.41	-3.10	-1.96	-1.33
WAN014G91_at	7.4	6.6	2.4	2.2	1.7	SA2344	SA2139	SAR2434	SAS2240	SAV2349	MW2270		hypothetical protein	Hypothetical proteins	Not Conserved	-1.11	-3.05	-3.36	-4.32
WAN014GQ0_at	50.2	1.9	16.8	8.2	7.6	SA0868	SA0752	SAR0855	SAS0764	SAV0823	MW0777		hypothetical protein	Hypothetical proteins	Not Conserved	-27.12	-2.99	-6.14	-6.57
WAN014ILK_at	10.5	5.6	3.5	3.5	5.9	SA2588	SA2359			SAV2572			hypothetical protein	Hypothetical proteins	Not Conserved	-1.86	-2.97	-2.96	-1.78
WAN014FZW_at	60.3	24.9	20.3	29.5	18.4	SA2433							hypothetical protein	Hypothetical proteins	Not Conserved	-2.42	-2.97	-2.05	-3.28
WAN014GHA_at	1.2		0.4	0.1	0.6	SA0492							hypothetical protein	Hypothetical proteins	Not Conserved		-2.92	-15.39	-2.02
WAN014HU3_at	67.1	25.0	23.0	19.6	23.3	SA1112	SA0954	SAR1077	SAS1038	SAV1103	MW0986		hypothetical protein	Hypothetical proteins	Not Conserved	-2.69	-2.92	-3.42	-2.88
WAN014HYC_at	104.8	27.5	36.0	35.8	23.6	SA2076	SAS068	SAR2173	SAS1989	SAV2085	MW2008		hypothetical protein	Hypothetical proteins	Not Conserved	-3.81	-2.91	-2.92	-4.45
WAN014G2I_at	6.0	2.8	2.1	1.7	1.9	SA1971	SA1726	SAR2002	SAS1833	SAV1910	MW1851		hypothetical protein	Hypothetical proteins	Not Conserved	-2.10	-2.90	-3.60	-3.14
WAN014HTV_at	11.6	8.6	4.0	4.1	4.4	SA1993	SA1744	SAR2022	SAS1854	SAV1930	MW1871		hypothetical protein	Hypothetical proteins	Not Conserved	-1.34	-2.89	-2.80	-2.60
WAN014G9B_at	2.3	2.0	0.8	1.2		SA2626	SA2404	SAR2689	SAS2496	SAV2611	MW2530		hypothetical protein	Hypothetical proteins	Not Conserved	-1.18	-2.89	-1.99	
WAN014GBW_at	9.9	8.5	3.5	3.2	3.3	SA0455	SA0370						hypothetical protein	Hypothetical proteins	Not Conserved	-1.16	-2.83	-3.12	-3.02
WAN01BPH1_at	27.7	1.1	9.8	5.4	6.2	SA0866							hypothetical protein	Hypothetical proteins	Not Conserved	-25.71	-2.83	-5.13	-4.48
WAN014GUC_at	43.6	23.1	16.4	21.8	22.1	SA1117	SAS031	SAR1082	SAS1043	SAV1108	MW0991		hypothetical protein	Hypothetical proteins	Not Conserved	-1.89	-2.66	-2.00	-1.97
WAN01BQT6_at	1.5		0.6			SA2604							hypothetical protein	Hypothetical proteins	Not Conserved		-2.66		
WAN014GV1_at	3.7	2.6	1.4	2.2	1.9	SA1166	SA1001			SAV1156			hypothetical protein	Hypothetical proteins	Not Conserved	-1.42	-2.64	-1.69	-1.96
WAN014G2N_at	1.4		0.5	0.7		SA2481			SAS2362		MW2395		hypothetical protein	Hypothetical proteins	Not Conserved		-2.55	-2.10	
WAN01BPDF_at	2.6	1.9	1.0			SA0732							hypothetical protein	Hypothetical proteins	Not Conserved	-1.38	-2.47		
WAN014FVD_at	3.3	2.1	1.3	1.2	1.9	SA2320	SA2118						hypothetical protein	Hypothetical proteins	Not Conserved	-1.53	-2.45	-2.69	-1.77
WAN014I3A_at	5.2	1.9	2.2	1.6	1.3	SA2169	SA1981	SAR2270	SAS2080	SAV2179	MW2105		hypothetical protein	Hypothetical proteins	Not Conserved	-2.76	-2.38	-3.18	-4.05
WAN014HM0_at	1.1	0.9	0.5	0.7	0.9	SA0983	SA0839	SAR0942	SAS0849		MW0861		hypothetical protein	Hypothetical proteins	Not Conserved	-1.24	-2.37	-1.62	-1.23
WAN014FV1_at	22.4	26.5	9.5	9.1	9.6	SA2312	SAS085	SAR2403	SAS2212	SAV2319	MW2240		hypothetical protein	Hypothetical proteins	Not Conserved	1.19	-2.34	-2.46	-2.32
WAN014H9M_at	53.2	6.5	23.0	14.1	12.1	SA0738	SA0633	SAR0731	SAS0643	SAV0678	MW0640		hypothetical protein	Hypothetical proteins	Not Conserved	-8.16	-2.32	-3.77	-4.40
WAN014G38_at	1.8	2.3	0.8	1.0	1.3	SA2495	SA2271			SAV2483			hypothetical protein	Hypothetical proteins	Not Conserved	1.30	-2.30	-1.79	-1.36
WAN014G68_at	6.3	5.9	2.8	2.5	2.3	SA2171	SA1983	SAR2272	SAS2082	SAV2181	MW2107		hypothetical protein	Hypothetical proteins	Not Conserved	-1.08	-2.22	-2.54	-2.77
WAN014FSN_at	2.6	3.5	1.2	1.3	1.0	SA1359	SA1162	SAR1336	SAS1266	SAV1326	MW1213		hypothetical protein	Hypothetical proteins	Not Conserved	1.35	-2.22	-2.09	-2.75
WAN014G36_at	10.0	15.3	4.5	4.5	3.4	SA2494	SA2270			SAV2482			hypothetical protein	Hypothetical proteins	Not Conserved	1.54	-2.21	-2.23	-2.89
WAN014G8D_at	18.2	12.4	8.3	9.5	9.1	SA2310	SA2110	SAR2401	SAS2210	SAV2317	MW2238		hypothetical protein	Hypothetical proteins	Not Conserved	-1.47	-2.20	-1.91	-1.99
WAN01BPPH_at	80.9	100.3	37.2	38.8	29.3	SA1171	SAS035		SAS1094	SAV1160	MW1042		hypothetical protein	Hypothetical proteins	Not Conserved	1.24	-2.17	-2.08	-2.76
WAN01BX6J_x_at	5.0	3.3	2.3	2.5	3.7				SAS0230				hypothetical protein	Hypothetical proteins	Not Conserved	-1.55	-2.15	-2.04	-1.37
WAN01BQPX_x_at	2.5	2.0	1.2	0.9	1.6	SA2487	SA2265			SAV2477			hypothetical protein	Hypothetical proteins	Not Conserved	-1.21	-2.11	-2.73	-1.58
WAN014H90_at	1.3	1.5	0.6	0.7		SA0726	SA0623	SAR0679	SAS0633	SAV0668	MW0630		hypothetical protein	Hypothetical proteins	Not Conserved	1.21	-2.11	-1.77	

																Fold change vs stationary phase			
Systematic	Stat.	Log	30'	120'	360'	COL	N315	MRSA	MSSA	Mu50	MW2	GENE NAME	Protein Function	TIGR Main Role	TIGR Sub Role	LB	30' in vivo	120' in vivo	360' in vivo
WAN014HZ2_at	13.5	12.8	6.4	7.3	8.7	SA2087					MW2018		hypothetical protein	Hypothetical proteins	Not Conserved	-1.06	-2.10	-1.86	-1.55
WAN014ILH_at	6.9	2.1	3.3	2.4	2.7	SA2489	SA2267	SAR2568	SAS2371	SAV2479	MW2404		hypothetical protein	Hypothetical proteins	Not Conserved	-3.24	-2.09	-2.89	-2.59
WAN014GPG_at	7.3	9.9	3.5	2.8	4.4	SA0300	SA0292	SAR0301	SAS0281	SAV0304	MW0281		hypothetical protein	Hypothetical proteins	Not Conserved	1.35	-2.07	-2.58	-1.66
WAN014GNV_at	2.5	3.4	1.2	1.0		SA0280	SA0280		SAS0267	SAV0292	MW0267		hypothetical protein	Hypothetical proteins	Not Conserved	1.35	-2.05	-2.45	
WAN014H9A_at	1.7	1.8	0.8	0.7		SA1658	SA1431		SAS1539	SAV1603	MW1553		hypothetical protein	Hypothetical proteins	Not Conserved	1.05	-2.04	-2.27	
WAN01BQJM_x_a	2.1	1.6	1.0	1.2		SA2260							hypothetical protein	Hypothetical proteins	Not Conserved	-1.26	-2.03	-1.74	
WAN014H36_at	3.5	4.4	1.7	1.2	1.9	SA1544	SA1332	SAR1577		SAV1501			hypothetical protein	Hypothetical proteins	Not Conserved	1.26	-2.01	-2.92	-1.88
WAN014GNP_at	1.1	2.2	0.6	0.2		SA0278	SA0278		SAS0265	SAV0290	MW0265	esxB	hypothetical protein	Hypothetical proteins	Not Conserved	2.02	-2.00	-6.89	
WAN01BQ5O_at	4.2	2.8	2.1	2.6	2.2	SA1755							hypothetical protein	Hypothetical proteins	Not Conserved	-1.53	-2.00	-1.60	-1.89
WAN014H6L_at	99.9	33.9	50.3	33.9	34.7	SA0669	SA0570	SAR0622	SAS0581	SAV0613	MW0577		hypothetical protein	Hypothetical proteins	Not Conserved	-2.95	-1.99	-2.95	-2.88
WAN01BXAD_at	2.3	2.2	1.1	0.9	1.2		#N/A						antisense orf	Hypothetical proteins	Role category not yet assigned	-1.03	-2.12	-2.66	-1.92
WAN014GLL_at	1.3	0.8	0.3						SAS1885		MW1902		uncharacterized phage protein (possible DNA packaging)	Mobile and extrachromosomal element functions	Prophage functions	-1.54	-3.84		
WAN014GMH_at	6.0	3.0	2.0	1.5	2.1		#N/A						hypothetical phage protein-related protein	Mobile and extrachromosomal element functions	Prophage functions	-2.01	-3.06	-3.88	-2.85
WAN01BZ3A_at	3.7	1.4	0.9	0.7	0.9				SAS2573		MW2607		hypothetical protein	No Data	No Data	-2.70	-4.16	-5.02	-4.13
WAN014FTJ_at	1.8	1.0	0.6	0.6	0.7				SAS0376		MW0375		hypothetical protein	No Data	No Data	-1.82	-3.15	-2.80	-2.65
WAN014GR7_at	51.8	24.9	23.3	27.8	30.7			SAR2237	SAS2051		MW2072		hypothetical protein	No Data	No Data	-2.09	-2.22	-1.86	-1.69
WAN014HX6_at	37.1	29.1	14.2	12.7	10.4	SA2058	SA1873	SAR2156	SAS1973	SAV2068	MW1992		pemK protein, putative	Other categories	Plasmid functions	-1.28	-2.61	-2.92	-3.59
WAN014HND_at	21.1	23.1	9.2	7.1	7.7	SA1898	SA1660	SAR1933	SAS1763	SAV1842	MW1783	cbf1	cmp-binding-factor 1	Other categories	Plasmid functions	1.10	-2.30	-2.96	-2.74
WAN014GRZ_at	1.4	1.0	0.7	0.6	1.0	SA0360				SAV0879			bacteriophage L54a, hypothetical protein	Other categories	Prophage functions	-1.39	-2.16	-2.25	-1.46
WAN014HMG_at	5.1	5.1	1.8	1.8	2.4	SA1878			SAS1746		MW1765		IS1181, transposase	Other categories	Transposon functions	1.01	-2.75	-2.82	-2.09
WAN014GST_at	91.0	11.3	7.1	4.8	4.6	SA1055	SA0899	SAR1020	SAS0982	SAV1046	MW0930	sspC	Staphostatin B	Protein fate	Degradation of proteins, peptides, and glycopeptides	-8.03	-12.72	-18.86	-19.61
WAN014GSZ_x_at	24.0	4.4	2.2	2.1	2.1	SA1057		SAR1022	SAS0984	SAV1048	MW0932	sspA (v8 p	glutamyl endopeptidase precursor	Protein fate	Degradation of proteins, peptides, and glycopeptides	-5.40	-11.13	-11.22	-11.41
WAN014GSW_at	5.8	1.7	0.9	1.1	1.0	SA1056	SA0900	SAR1021	SAS0983	SAV1047	MW0931	sspB	staphopain	Protein fate	Degradation of proteins, peptides, and glycopeptides	-3.31	-6.11	-5.42	-5.51
WAN014G37_s_at	13.9	1.5	2.4	1.5	1.6	SA2007	SA1814						peptidase, M20/M25/M40 family, authentic frameshift	Protein fate	Degradation of proteins, peptides, and glycopeptides	-9.16	-5.84	-9.58	-8.86
WAN014HNN_at	22.1	43.4	4.4	4.6	4.7	SA1005	SA0859	SAR0968	SAS0870	SAV1000	MW0882	pepF	oligoendopeptidase F	Protein fate	Degradation of proteins, peptides, and glycopeptides	1.96	-5.08	-4.83	-4.75
WAN014GDA_at	11.4	4.3	2.7	1.3	1.7	SA2563	SA2336	SAR2628	SAS2434	SAV2548	MW2469	clpC	ATP-dependent Clp protease, ATP-binding subunit ClpC	Protein fate	Degradation of proteins, peptides, and glycopeptides	-2.63	-4.27	-9.08	-6.57
WAN014FYE_at	4.8	5.3	1.3	1.6	1.3	SA1756	SA1530	SAR1786	SAS1635	SAV1708	MW1651		proline dipeptidase	Protein fate	Degradation of proteins, peptides, and glycopeptides	1.09	-3.73	-3.11	-3.74
WAN014G2F_at	6.8	2.5	1.9	1.4	1.9	SA1970	SA1725	SAR2001	SAS1832	SAV1909	MW1850	sspB	staphopain	Protein fate	Degradation of proteins, peptides, and glycopeptides	-2.68	-3.59	-4.74	-3.65
WAN014HLL_at	12.5	5.1	3.6	7.2	14.8	SA0979	SA0835	SAR0938	SAS0845	SAV0975	MW0857	clpB	ATP-dependent Clp protease, ATP-binding subunit ClpB	Protein fate	Degradation of proteins, peptides, and glycopeptides	-2.48	-3.50	-1.74	1.18
WAN014H3L_at	7.4	5.0	2.1	2.0	2.2	SA1555	SA1343	SAR1590	SAS1451	SAV1512	MW1465	pepT	peptidase T	Protein fate	Degradation of proteins, peptides, and glycopeptides	-1.49	-3.48	-3.69	-3.35
WAN014H1L_at	29.7	26.0	8.8	9.8	15.2	SA0570	SA0483	SAR0528	SAS0482	SAV0525	MW0480	clpC	ATP-dependent Clp protease, ATP-binding subunit ClpC, authentic frameshift	Protein fate	Degradation of proteins, peptides, and glycopeptides	-1.14	-3.36	-3.03	-1.96
WAN014FVV_at	15.7	17.1	5.2	5.1	3.9	SA1419	SA1216	SAR1397	SAS1325	SAV1384	MW1272	pepF	oligoendopeptidase F	Protein fate	Degradation of proteins, peptides, and glycopeptides	1.09	-3.02	-3.08	-4.05
WAN014FYU_at	17.6	28.2	6.4	6.1	8.1	SA1777	SA1549	SAR1805	SAS1654	SAV1728	MW1670	htrA	serine protease HtrA	Protein fate	Degradation of proteins, peptides, and glycopeptides	1.61	-2.74	-2.90	-2.16
WAN014G15_at	9.0	8.0	3.4	3.0	3.9	SA2463	SA2244	SAR2545	SAS2347	SAV2455	MW2379		peptidase, M42 family	Protein fate	Degradation of proteins, peptides, and glycopeptides	-1.13	-2.66	-2.96	-2.31
WAN014HGR_at	10.1	5.7	3.8	2.8	3.2	SA1795	SA1566	SAR1823	SAS1671	SAV1745	MW1688		peptidase, M42 family	Protein fate	Degradation of proteins, peptides, and glycopeptides	-1.75	-2.64	-3.61	-3.13
WAN014HH9_at	33.9	35.9	13.5	11.8	10.2	SA1801	SA1572	SAR1836	SAS1677	SAV1751	MW1694	pepV	peptidase, M20/M25/M40 family	Protein fate	Degradation of proteins, peptides, and glycopeptides	1.06	-2.51	-2.87	-3.32
WAN014HKH_at	12.5	15.3	5.1	4.7	4.9	SA0945	SA0803	SAR0904	SAS0812	SAV0942	MW0824	ampA	cytosol aminopeptidase family protein	Protein fate	Degradation of proteins, peptides, and glycopeptides	1.23	-2.42	-2.68	-2.52
WAN014GIC_at	5.4	12.9	2.3	2.3	3.4	SA0595	SA0507	SAR0554	SAS0507	SAV0549	MW0504		peptidase, M20/M25/M40 family	Protein fate	Degradation of proteins, peptides, and glycopeptides	2.40	-2.36	-2.38	-1.56
WAN014GLD_at	3.2	2.1	1.5	1.3	2.1				SAS1878		MW1895		M23/M37 peptidase domain protein protein	Protein fate	Degradation of proteins, peptides, and glycopeptides	-1.53	-2.16	-2.43	-1.52
WAN014G0Z_at	4.3	4.5	2.1	1.9	2.9	SA1897	SA1659	SAR1932	SAS1762	SAV1841	MW1782	prsA	protease maturation protein	Protein fate	Degradation of proteins, peptides, and glycopeptides	1.04	-2.06	-2.33	-1.47
WAN014GC0_at	9.0	6.0	2.4	2.1	3.1	SA2671	SA2442	SAR2728	SAS2535	SAV2649	MW2570	secA2	secA family protein	Protein fate	Protein and peptide secretion and trafficking	-1.48	-3.69	-4.32	-2.92
WAN014A80-seq1	11.4	30.1	3.6	4.0	3.9	SA2676		SAR2734	SAS2540	SAV2654	MW2575	sasA	LPXTG-motif cell wall anchor domain protein	Protein fate	Protein and peptide secretion and trafficking	2.65	-3.12	-2.80	-2.88
WAN014A80-seq2	14.5	31.0	5.3	4.9	5.0	SA2676			SAS2540	SAV2654	MW2575	sasA	LPXTG-motif cell wall anchor domain protein	Protein fate	Protein and peptide secretion and trafficking	2.13	-2.75	-2.96	-2.92
WAN014GC5_at	1.5	2.6	0.6	0.5		SA2674	SA2445		SAS2538	SAV2652	MW2573	asp1	accessory secretory protein Asp1	Protein fate	Protein and peptide secretion and trafficking	1.70	-2.71	-2.82	
WAN014GC3_at	2.9	4.4	1.2		1.5	SA2673	SA2444	SAR2730	SAS2537	SAV2651	MW2572	asp2	accessory secretory protein Asp2	Protein fate	Protein and peptide secretion and trafficking	1.52	-2.43		-2.01
WAN014HVE_at	15.2	11.6	3.1	2.7	3.0	SA2016	SA1836	SAR2116	SAS1935	SAV2029	MW1953	groEL	chaperonin, 60 kDa	Protein fate	Protein folding and stabilization	-1.31	-4.92	-5.64	-5.02
WAN014H89_at	52.2	31.1	14.8	17.6	24.1	SA1637	SA1409	SAR1657	SAS1518	SAV1580	MW1532	dnaK	dnak protein	Protein fate	Protein folding and stabilization	-1.68	-3.53	-2.96	-2.17
WAN014HVI_at	5.7	5.0	1.7	1.8	2.6	SA2017	SA1837	SAR2117	SAS1936	SAV2030	MW1954	groES	chaperonin, 10 kDa	Protein fate	Protein folding and stabilization	-1.15	-3.42	-3.15	-2.18
WAN014HKM_at	18.4	20.5	8.0	7.3	9.6	SA0957	SA0815	SAR0916	SAS0824	SAV0954	MW0836		peptidyl-prolyl cis-trans isomerase, cyclophilin-type	Protein fate	Protein folding and stabilization	1.11	-2.31	-2.52	-1.93
WAN014HR2_at	49.0	45.7	21.1	19.0	17.8	SA1946	SA1704	SAR1978	SAS1810	SAV1888	MW1828	map	methionine aminopeptidase, type I	Protein fate	Protein modification and repair	-1.07	-2.32	-2.57	-2.75
WAN014HD7_at	17.0	20.3	7.4	5.7	7.2	SA0826	SA0716	SAR0815	SAS0726	SAV0761	MW0723	lgt	prolipoprotein diacylglyceryl transferase	Protein fate	Protein modification and repair	1.20	-2.28	-2.99	-2.35
WAN014H5L_at	11.7	8.7	5.3	4.9	3.9	SA1591	SA1363	SAR1610	SAS1471	SAV1533	MW1485		lipoate-protein ligase A	Protein fate	Protein modification and repair	-1.35	-2.23	-2.41	-3.02
WAN014GWR_at	8.7	10.4	4.0	4.0	4.6	SA1397	SA1194	SAR1373	SAS1301	SAV1361	MW1248	msrA	peptide methionine sulfoxide reductase	Protein fate	Protein modification and repair	1.19	-2.20	-2.17	-1.91
WAN014GUP_at	5.1	3.1	0.9	1.0	1.2	SA0413	SA0330						ribosomal-protein-serine acetyltransferase, putative	Protein synthesis	Ribosomal proteins: synthesis and modification	-1.62	-5.77	-5.08	-4.19
WAN014GVM_at	1126.1	800.0	562.5	658.0	695.7	SA1238	SA1067	SAR1200	SAS1158	SAV1224	MW1107	rpmB	ribosomal protein L28	Protein synthesis	Ribosomal proteins: synthesis and modification	-1.41	-2.00	-1.71	-1.62
WAN014HCJ_at	32.5	18.9	7.9	9.1	14.8	SA0815	SA0707	SAR0806	SAS0717	SAV0752	MW0714		ribosomal subunit interface protein	Protein synthesis	Translation factors	-1.72	-4.13	-3.59	-2.19
WAN014HDI_at	62.1	40.3	14.6	15.0	12.7	SA1729	SA1506	SAR1762	SAS1611	SAV1683	MW1626	thrS	threonyl-tRNA synthetase	Protein synthesis	tRNA aminoacylation	-1.54	-4.26	-4.14	-4.90
WAN014FXT_at	66.5	27.5	25.5	23.2	22.7	SA1622	SA1394	SAR1642	SAS1503	SAV1565	MW1517	glyS	glycyl-tRNA synthetase	Protein synthesis	tRNA aminoacylation	-2.42	-2.61	-2.87	-2.93
WAN014I4F_at	5.5	1.1	1.7	1.5	1.9	SA1290	SA1114	SAR1247	SAS1205	SAV1271	MW1154	truB	tRNA pseudouridine 55 synthase	Protein synthesis	tRNA and rRNA base modification	-5.18	-3.19	-3.58	-2.87
WAN014G9T_at	20.0	51.8	5.2	3.6	2.8	SA2635	SA2410	SAR2695	SAS2503	SAV2617	MW2537	nrdD	anaerobic ribonucleoside-triphosphate reductase	Purines, pyrimidines, nucleosides, and nucleotides	2'-Deoxyribonucleotide metabolism	2.59	-3.84	-5.62	-7.11
WAN014GQ3_at	7.2	7.4	2.6	2.9	3.0	SA0024	SA0022					surE	5-nucleotidase	Purines, pyrimidines, nucleosides, and nucleotides	Nucleotide and nucleoside interconversions	1.02	-2.72	-2.45	-2.40
WAN014HPG_at	5.3	5.8	2.6	2.3	2.5	SA1031	SA0881	SAR0994	SAS0957	SAV1025	MW0905		2`,3`-cyclic-nucleotide 2`-phosphodiesterase, putative	Purines, pyrimidines, nucleosides, and nucleotides	Other	1.09	-2.02	-2.36	-2.13
WAN014GVY_at	45.7	110.7	10.5	12.4	15.5	SA0460	SA0375	SAR0408	SAS0366	SAV0390	MW0366	guaB	inosine-5-monophosphate dehydrogenase	Purines, pyrimidines, nucleosides, and nucleotides	Purine ribonucleotide biosynthesis	2.42	-4.35	-3.70	-2.95
WAN014GVZ_at	57.5	105.9	15.4	15.3	17.9	SA0461	SA0376	SAR0409	SAS0367	SAV0391	MW0367	guaA	GMP synthase	Purines, pyrimidines, nucleosides, and nucleotides	Purine ribonucleotide biosynthesis	1.84	-3.72	-3.76	-3.21
WAN014GVU_at	13.4	66.9	2.9	4.2	5.2	SA0458	SA0373	SAR0406	SAS0364	SAV0388	MW0364	xprT	xanthine phosphoribosyltransferase	Purines, pyrimidines, nucleosides, and nucleotides	Salvage of nucleosides and nucleotides	5.00	-4.59	-3.15	-2.59
WAN014I08_at	21.3	14.3	6.7	6.1	7.5	SA2104	SA1914	SAR2200	SAS2015	SAV2112	MW2036	upp	uracil phosphoribosyltransferase	Purines, pyrimidines, nucleosides, and nucleotides	Salvage of nucleosides and nucleotides	-1.49	-3.18	-3.47	-2.86
WAN014G7M_at	86.4	18.6	1.6	1.2	1.5	SA0179	SA0187	SAR0194	SAS0168	SAV0193	MW0167		transcriptional regulator, RpiR family	Regulatory functions	DNA interactions	-4.64	-54.74	-73.86	-58.21
WAN014HU2_at	9.9	3.1	2.5	2.1	2.3	SA0257	SA0261	SAR0269	SAS0248	SAV0271	MW0247		ribose operon repressor, putative	Regulatory functions	DNA interactions	-3.16	-3.90	-4.75	-4.29
WAN014H8E_at	10.7	5.8	3.0	4.5	5.0	SA1639	SA1411	SAR1659	SAS1520	SAV1582	MW1534	hrcA	heat-inducible transcription repressor HrcA	Regulatory functions	DNA interactions	-1.84	-3.51	-2.34	-2.12
WAN014G8M_at	2.0	1.7	0.6	0.8		SA2325	SA2123	SAR2418	SAS2225	SAV2332	MW2253		transcriptional regulator, LysR family	Regulatory functions	DNA interactions	-1.19	-3.37	-2.70	
WAN014H99_at	7.1	5.2	2.3	2.1	2.4	SA0731	SA0627	SAR0683	SAS0637	SAV0672	MW0634		transcriptional regulator, LysR family	Regulatory functions	DNA interactions	-1.35	-3.08	-3.33	-2.96
WAN014HVV_at	3.5	2.1	1.3	1.1	1.7	SA2030	SA1847	SAR2129	SAS1947	SAV2042	MW1966	scrR	sucrose operon repressor	Regulatory functions	DNA interactions	-1.67	-2.81	-3.09	-2.14
WAN014G6C_at	5.4	3.8	2.1	1.9	2.5	SA2189	SA1999	SAR2288	SAS2098	SAV2197	MW2123		transcriptional regulator, Sir2 family	Regulatory functions	DNA interactions	-1.45	-2.66	-2.88	-2.21
WAN014G5J_at	3.4	31.1	1.3	1.5	1.3	SA2137	SA1947	SAR2233	SAS2048	SAV2145	MW2069	czrA	transcriptional regulator	Regulatory functions	DNA interactions	9.09	-2.63	-2.33	-2.62
WAN014H9D_at	4.5	4.5	1.7	1.5	1.9	SA0084	SA0097	SAR0107	SAS0078	SAV0101	MW0077		transcriptional regulator, AraC family	Regulatory functions	DNA interactions	1.00	-2.60	-2.93	-2.42
WAN014HUE_at	1.8	1.1	0.7	0.5	0.7	SA1997	SA1748	SAR2026	SAS1858	SAV1934	MW1875		transcriptional regulator, gntR family	Regulatory functions	DNA interactions	-1.60	-2.54	-3.90	-2.59
WAN014GXY_at	4.8	4.4	1.9	1.7	1.3	SA0120	SA0130	SAR0137	SAS0109	SAV0135	MW0109		transcriptional regulator, GntR family	Regulatory functions	DNA interactions	-1.08	-2.51	-2.80	-3.59
WAN014GRR_at	2.0	1.7	0.8	1.2		SA0980	SA0836	SAR0939	SAS0846	SAV0976	MW0858		transcriptional regulator, LysR family	Regulatory functions	DNA interactions	-1.19	-2.47	-1.66	
WAN014H8L_at	3.8	3.7	1.6	1.4	2.0	SA0716	SA0614	SAR0669	SAS0624	SAV0659	MW0621		DNA-binding response regulator, putative	Regulatory functions	DNA interactions	-1.03	-2.35	-2.65	-1.90
WAN014GWG_at	122.4	34.3	52.3	47.1	48.2	SA1374	SA1174	SAR1349	SAS1279	SAV1339	MW1226	lexA	transcriptional repressor LexA	Regulatory functions	DNA interactions	-3.57	-2.34	-2.60	-2.54
WAN014H44_at	4.4	3.0	1.9	1.7	1.9	SA1565	SA1351	SAR1598	SAS1459	SAV1520	MW1473	ahrC	transcriptional regulator, ArgR family	Regulatory functions	DNA interactions	-1.44	-2.27	-2.65	-2.34
WAN014H8Y_at	1.7	1.8	0.8	1.0	1.3	SA0725	SA0622		SAS0632	SAV0667	MW0629		transcriptional regulator, AraC family	Regulatory functions	DNA interactions	1.06	-2.26	-1.74	-1.29
WAN014HP5_at	24.4	15.1	10.8	11.4	12.2	SA1919	SA1678	SAR1951	SAS1783	SAV1861	MW1801	furB	transcriptional regulator, Fur family	Regulatory functions	DNA interactions	-1.61	-2.25	-2.13	-1.99
WAN014GI2_at	1.3	1.2	0.6	0.9		SA0563	SA0476	SAR0521	SAS0475	SAV0518	MW0473		transcriptional regulator, gntR family	Regulatory functions	DNA interactions	-1.08	-2.25	-1.47	
WAN014GS9_at	26.1	10.0	6.5	5.5	3.3	SA1032	SA0882	SAR0995	SAS0958	SAV1026	MW0906		competence transcription factor, putative	Regulatory functions	Other	-2.61	-4.02	-4.77	-7.96
WAN014GPO_at	55.0	62.2	17.8	20.8	18.5	SA0307	SA0298	SAR0307	SAS0287	SAV0310	MW0287	pfoR	perfringolysin O regulator protein	Regulatory functions	Other	1.13	-3.09	-2.65	-2.97
WAN014A7Z-seq1	10.2	28.8	3.6	3.7	3.6		SA2447	SAR2734	SAS2540	SAV2654	MW2575		serine-threonine rich antigen	Regulatory functions	Other	2.84	-2.83	-2.73	-2.83
WAN014GVO_at	1.4	2.4	0.5	0.4	0.9	SA0096	SA0108	SAR0115	SAS0086	SAV0112	MW0085	sarS	staphylococcal accessory regulator S	Regulatory functions	Other	1.74	-2.75	-3.10	-1.53
WAN014HR3_at	3.4	2.6	1.2	1.3	1.6	SA1060	SA0904	SAR1025	SAS0987	SAV1051	MW0935		transcriptional regulator, MarR family	Regulatory functions	Other	-1.30	-2.75	-2.63	-2.15
WAN014A7Z-seq2	17.0	39.7	6.4	5.7	5.5		SA2447		SAS2540	SAV2654	MW2575		serine-threonine rich antigen	Regulatory functions	Other	2.34	-2.64	-2.99	-3.08
WAN014FTO_at	53.3	31.3	21.7	19.4	22.2	SA2287	SA2089	SAR2379	SAS2185	SAV2295	MW2213	sarR	staphylococcal accessory regulator R	Regulatory functions	Other	-1.70	-2.46	-2.75	-2.40
WAN014FUR_at	16.3	12.0	7.6	6.6	9.2	SA2302	SA2103	SAR2394	SAS2202	SAV2310	MW2230		transcriptional regulator, putative	Regulatory functions	Other	-1.35	-2.15	-2.46	-1.77
WAN014GME_at	2.2	1.1	1.0	1.0	1.0						MW1932		anti repressor	Regulatory functions	Other	-1.93	-2.14	-2.15	-2.28
WAN014IKS_at	21.1	22.9	6.0	7.3	8.2	SA1891	SA1653		SAS1756	SAV1835	MW1775	TRAP	RNAIII-activating Protein TRAP	Regulatory functions	Protein interactions	1.08	-3.52	-2.88	-2.57
WAN014HE2_at	23.3	10.1	9.1	9.1	7.2	SA1739	SA1515	SAR1771	SAS1620	SAV1692	MW1636	phoR	sensory box histidine kinase	Regulatory functions	Protein interactions	-2.30	-2.56	-2.56	-3.24
WAN014HD5_at	8.0	6.7	3.6	2.7	2.8	SA0825	SA0715	SAR0814	SAS0725	SAV0760	MW0722	hprK	HPr kinase/phosphatase	Regulatory functions	Protein interactions	-1.19	-2.18	-2.90	-2.79
WAN014GEZ_at	2.2	4.0	0.6	0.6	0.9	SA2662	SA2433	SAR2719	SAS2526	SAV2640	MW2561	bglG2	transcriptional antiterminator, bglG family	Regulatory functions	RNA interactions	1.80	-3.56	-3.66	-2.33
WAN014GNQ_at	28.1	9.5	3.7	4.9	8.2	SA0766	SA0661	SAR0759	SAS0671	SAV0706	MW0668	saeR	two-component signal transduction protein SaeR	Signal transduction	Two-component systems	-2.96	-7.53	-5.69	-3.42
WAN014GNO_at	23.8	6.2	3.2	3.9	4.9	SA0765	SA0660	SAR0758	SAS0670	SAV0705	MW0667	saeS	sensor histidine kinase SaeS	Signal transduction	Two-component systems	-3.83	-7.48	-6.06	-4.84
WAN014GYE_at	10.7	1.9	1.9	1.4	1.5	SA1451	SA1247	SAR1427	SAS1358	SAV1415	MW1305	arlR	DNA-binding response regulator	Signal transduction	Two-component systems	-5.61	-5.60	-7.57	-7.12
WAN014GKT_at	3.5	5.6	1.0	1.0	0.8	SA0246	SA0251	SAR0258	SAS0238	SAV0261	MW0237	lytR	response regulator	Signal transduction	Two-component systems	1.59	-3.53	-3.48	-4.59

Systematic	Stat.	Log	30'	120'	360'	COL	N315	MRSA	MSSA	Mu50	MW2	GENE NAME	Protein Function	TIGR Main Role	TIGR Sub Role	Fold change vs stationary phase LB	30' in vivo	120' in vivo	360' in vivo
WAN014HE4_at	8.5	5.0	2.4	3.1	3.2	SA1740	SA1516	SAR1772	SAS1621	SAV1693	MW1637	phoP	DNA-binding response regulator	Signal transduction	Two-component systems	-1.72	-3.51	-2.76	-2.61
WAN014GKR_at	10.8	19.7	3.5	4.1	3.5	SA0245	SA0250	SAR0257	SAS0237	SAV0260	MW0236	lytS	sensor histidine kinase	Signal transduction	Two-component systems	1.82	-3.07	-2.64	-3.05
WAN014GKV s at	8.8	8.1	3.3	3.4	3.5	SA0019	SA0017	SAR0018	SAS0018	SAV0018	MW0018	yycF	DNA-binding response regulator	Signal transduction	Two-component systems	-1.09	-2.72	-2.64	-2.52
WAN014HO5_at	6.9	5.5	3.0	2.9	2.8	SA1906	SA1667	SAR1940	SAS1770	SAV1849	MW1790		sensor histidine kinase, putative	Signal transduction	Two-component systems	-1.26	-2.26	-2.42	-2.48
WAN014G9A_at	2.3	2.7	1.0	1.1	1.2	SA2358	SA2151	SAR2447	SAS2252	SAV2361	MW2282		DNA-binding response regulator	Signal transduction	Two-component systems	1.20	-2.19	-2.01	-1.97
WAN014GN3_at	18.9	23.6	8.7	6.8	6.0	SA0020	SA0018	SAR0019	SAS0019	SAV0019	MW0019	yycG	sensory box histidine kinase YycG	Signal transduction	Two-component systems	1.25	-2.18	-2.79	-3.17
WAN014IMX_at	7.2	7.5	3.4	2.7	1.9	SA0717	SA0615		SAS0625	SAV0660	MW0622		sensor histidine kinase	Signal transduction	Two-component systems	1.03	-2.16	-2.68	-3.81
WAN014G4T_at	2.4	2.0	1.1	1.1	1.2	SA2071	SA1883	SAR2167	SAS1984	SAV2079	MW2003	kdpE	DNA-binding response regulator	Signal transduction	Two-component systems	-1.16	-2.13	-2.20	-1.92
WAN014GZV_at	146.1	17.8	66.2	36.2	30.8	SA0540	SA0455	SAR0498	SAS0454	SAV0497	MW0452		endoribonuclease L-PSP, putative	Transcription	Degradation of RNA	-8.22	-2.21	-4.04	-4.75
WAN014G1Y_at	66.3	7.5	30.5	17.9	19.2	SA1941	SA1699	SAR1973	SAS1805	SAV1883	MW1823		ribonuclease BN, putative	Transcription	RNA processing	-8.87	-2.17	-3.71	-3.46
WAN014GBI_at	11.3	19.9	1.5	1.8	2.1	SA2441	SA2227	SAR2528	SAS2330	SAV2438	MW2362		amino acid permease	Transport and binding proteins	Amino acids, peptides and amines	1.76	-7.75	-6.33	-5.36
WAN014GZP_at	29.5	7.5	4.0	3.1	3.7	SA1476	SA1270	SAR1449	SAS1380	SAV1437	MW1326		amino acid permease	Transport and binding proteins	Amino acids, peptides and amines	-3.96	-7.44	-9.65	-8.00
WAN014G0K_at	12.1	36.2	3.3	2.2	1.8	SA2450	SA2234	SAR2535	SAS2337	SAV2445	MW2369	opuCD	amino acid ABC transporter, permease protein	Transport and binding proteins	Amino acids, peptides and amines	3.00	-3.64	-5.55	-6.64
WAN014HDG_at	12.8	1.7	3.9	3.1	3.9	SA1728	SA1505	SAR1761	SAS1610	SAV1681	MW1625	lysP	amino acid permease	Transport and binding proteins	Amino acids, peptides and amines	-7.62	-3.33	-4.15	-3.32
WAN014G0N_at	3.5	12.0	1.1	0.7	1.3	SA2451	SA2235	SAR2536	SAS2338	SAV2446	MW2370	opuCC	amino acid transporter, putative	Transport and binding proteins	Amino acids, peptides and amines	3.42	-3.17	-4.72	-2.71
WAN014G0R_at	2.2	8.9	0.7	0.8	0.8	SA2453	SA2237	SAR2538	SAS2340	SAV2448	MW2372	opuCA	amino acid ABC transporter, ATP-binding protein	Transport and binding proteins	Amino acids, peptides and amines	4.02	-3.00	-2.84	-2.76
WAN014G2C_at	54.4	26.1	25.6	27.1	23.5	SA1963	SA1718	SAR1994	SAS1825	SAV1902	MW1843	putP	proline permease	Transport and binding proteins	Amino acids, peptides and amines	-2.09	-2.12	-2.01	-2.31
WAN014HA1_at	9.0	7.4	3.3	2.6	2.6	SA0750	SA0645	SAR0743	SAS0655	SAV0690	MW0652		anion transporter family protein	Transport and binding proteins	Anions	-1.22	-2.72	-3.50	-3.47
WAN014G6Z_at	99.4	27.2	2.1	2.1	2.8	SA0178	SA0186	SAR0193	SAS0167	SAV0192	MW0166		PTS system component	Transport and binding proteins	Carbohydrates, organic alcohols, and acids	-3.65	-48.11	-46.80	-35.64
WAN01BXAT_at	6.4	6.5	1.1	1.1	1.6				SAS0372				phosphotransferase system enzyme II , glucose-specific, factor IIB	Transport and binding proteins	Carbohydrates, organic alcohols, and acids	1.01	-5.73	-5.80	-3.98
WAN014GYF_at	9.6	85.1	1.8	1.8	1.9	SA0516	SA0432	SAR0473	SAS0431	SAV0474	MW0428	treP	PTS system component	Transport and binding proteins	Carbohydrates, organic alcohols, and acids	8.83	-5.31	-5.21	-5.15
WAN014G5W_at	8.5	1.1	2.1	1.4	2.6	SA2148	SA1962	SAR2246	SAS2059	SAV2158	MW2084	mtlA	PTS system, mannitol-specific component	Transport and binding proteins	Carbohydrates, organic alcohols, and acids	-7.80	-4.06	-5.89	-3.27
WAN014G42_at	6.5	5.7	1.7	2.0	2.4	SA2514	SA2293	SAR2582	SAS2390	SAV2505	MW2423		GntP family permease	Transport and binding proteins	Carbohydrates, organic alcohols, and acids	-1.16	-3.83	-3.34	-2.75
WAN014HD3_at	90.4	37.1	33.1	28.4	27.1	SA0093	SA0106	SAR0113	SAS0084	SAV0110	MW0083	lctP	L-lactate permease	Transport and binding proteins	Carbohydrates, organic alcohols, and acids	-2.44	-2.73	-3.19	-3.33
WAN014FV8_at	7.8	8.4	2.9	1.9	2.6	SA2316	SA2114	SAR2408	SAS2216	SAV2323	MW2244	glvC	PTS system component	Transport and binding proteins	Carbohydrates, organic alcohols, and acids	1.07	-2.70	-4.05	-3.00
WAN014GF7_at	2.5	1.9	1.0	1.2	1.2	SA0194	SA0208	SAR0207	SAS0191	SAV0215	MW0191	malF	maltose ABC transporter, permease protein	Transport and binding proteins	Carbohydrates, organic alcohols, and acids	-1.30	-2.56	-2.07	-2.00
WAN014FV6_at	6.2	23.1	2.7	3.5	4.2	SA0400	SA0318	SAR0326	SAS0306	SAV0329	MW0306	sgaT	SgaT protein, putative	Transport and binding proteins	Carbohydrates, organic alcohols, and acids	3.73	-2.27	-1.79	-1.47
WAN014HSG_at	308.7	195.4	138.3	93.3	90.1	SA1091	SA0934	SAR1056	SAS1018	SAV1083	MW0965	ptsH	phosphocarrier protein HPr	Transport and binding proteins	Carbohydrates, organic alcohols, and acids	-1.58	-2.23	-3.31	-3.42
WAN014GF0_at	6.7	31.3	3.2	5.0	7.6	SA2663	SA2434	SAR2720	SAS2527	SAV2641	MW2562		PTS system, fructose-specific IIABC component	Transport and binding proteins	Carbohydrates, organic alcohols, and acids	4.64	-2.08	-1.34	1.13
WAN014GFL_at	0.8	0.6	0.4		0.9	SA0195	SA0209	SAR0208	SAS0192	SAV0216	MW0192	malG	maltose ABC transporter, permease protein	Transport and binding proteins	Carbohydrates, organic alcohols, and acids	-1.42	-2.06		1.08
WAN014HTD_at	45.3	14.9	6.2	7.2	8.1	SA1979	SA1732	SAR2009	SAS1840	SAV1916	MW1857		sodium-dependent transporter	Transport and binding proteins	Cations	-3.04	-7.34	-6.32	-5.57
WAN014FY3_s_at	6.2	2.8	1.4	1.3	2.0	SA0155	SA0163	SAR0170	SAS0143	SAV0168	MW0143		cation efflux family protein	Transport and binding proteins	Cations	-2.17	-4.27	-4.85	-3.06
WAN014GFI_at	16.2	3.5	3.9	2.8	2.6	SA2721	SA2489	SAR2778	SAS2582	SAV2698	MW2617	ureH	high affinity nickel transporter	Transport and binding proteins	Cations	-4.64	-4.17	-5.83	-6.18
WAN014H74_at	38.4	4.0	10.8	5.6	8.8	SA0685	SA0583	SAR0635	SAS0594	SAV0627	MW0590		Na+/H+ antiporter component	Transport and binding proteins	Cations	-9.59	-3.54	-6.91	-4.37
WAN014IMV_at	15.0	2.5	4.4	3.3	4.9	SA0680	SA0579	SAR0631	SAS0590	SAV0623	MW0586		Na+/H+ antiporter component	Transport and binding proteins	Cations	-5.95	-3.41	-4.52	-3.04
WAN014I1L_at	3.6	38.8	1.1	2.0	3.8	SA0311	SA0303	SAR0311	SAS0291	SAV0314	MW0291		sodium:solute symporter family protein	Transport and binding proteins	Cations	10.79	-3.34	-1.80	1.06
WAN014H76_at	27.2	2.8	8.2	4.2	6.3	SA0686	SA0584		SAS0595	SAV0628	MW0591		Na+/H+ antiporter component	Transport and binding proteins	Cations	-9.73	-3.32	-6.42	-4.29
WAN014GU7_at	26.6	68.3	8.1	6.1	6.6	SA1114	SA0956	SAR1079	SAS1040	SAV1105	MW0988		Mn2+/Fe2+ transporter, NRAMP family	Transport and binding proteins	Cations	2.57	-3.30	-4.36	-4.04
WAN014H6Z_at	13.3	1.9	4.1	2.8	4.9	SA0682	SA0581	SAR0633	SAS0592	SAV0625	MW0588		Na+/H+ antiporter component	Transport and binding proteins	Cations	-6.86	-3.22	-4.77	-2.72
WAN014H72_at	5.7	1.2	1.9	1.2	2.2	SA0684	SA0582	SAR0634	SAS0593	SAV0626	MW0589		Na+/H+ antiporter component	Transport and binding proteins	Cations	-4.75	-3.03	-4.78	-2.65
WAN014IMW_at	5.4	0.9	1.9	1.5	2.8	SA0681	SA0580	SAR0632	SAS0591	SAV0624	MW0587		Na+/H+ antiporter component	Transport and binding proteins	Cations	-5.72	-2.82	-3.68	-1.95
WAN014H79_at	51.3	28.5	19.1	15.4	23.2	SA0687	SA0585	SAR0637	SAS0596	SAV0629	MW0592		Na+/H+ antiporter	Transport and binding proteins	Cations	-1.80	-2.69	-3.33	-2.21
WAN01C52T_at	7.8	11.6	3.1	3.2	3.1		#N/A						molybdate-binding protein	Transport and binding proteins	Cations	1.48	-2.50	-2.41	-2.51
WAN014FTW_at	14.8	5.3	6.1	6.5	7.8	SA2292	SA2094	SAR2385	SAS2191	SAV2301	MW2219		Na+/H+ antiporter	Transport and binding proteins	Cations	-2.80	-2.42	-2.29	-1.89
WAN014IWI_at	3.2	5.3	1.4	1.1	1.6	SA2272	SA2074					modA	molybdenum ABC transporter, molybdenum-binding protein	Transport and binding proteins	Cations	1.66	-2.32	-2.87	-1.99
WAN014GRB_at	52.0	72.7	23.6	26.9	29.4	SA0955	SA0813	SAR0914	SAS0822	SAV0952	MW0834	mnhA	Na+/H+ antiporter, MnhA component	Transport and binding proteins	Cations	1.40	-2.20	-1.93	-1.77
WAN014GR8_at	8.2	10.0	3.8	3.6	3.1	SA0953	SA0811	SAR0912	SAS0820	SAV0950	MW0832	mnhC	Na+/H+ antiporter, MnhC component	Transport and binding proteins	Cations	1.21	-2.18	-2.27	-2.67
WAN014GTM_at	2.3	3.2	1.1	1.2	1.6	SA1084	SA0927	SAR1049	SAS1011	SAV1075	MW0958	cbiQ	cobalt transport family protein	Transport and binding proteins	Cations	1.38	-2.16	-1.84	-1.40
WAN01A4RB_at	10.5	4.1	1.7	1.6	1.8			SAR0170	SAS0143	SAV0168	MW0143	efflux	cation-efflux system membrane protein CzcD	Transport and binding proteins	Cations and iron carrying compounds	-2.58	-6.07	-6.56	-5.86
WAN014G26_at	41.8	53.9	11.8	18.5	18.5	SA1952	SA1709	SAR1984	SAS1815	SAV1893	MW1834		ferritin family protein	Transport and binding proteins	Cations and iron carrying compounds	1.29	-3.54	-2.27	-2.26
WAN014H6D_at	2.3	2.6	0.8	0.8	0.8	SA0666	SA0567	SAR0619	SAS0578	SAV0610	MW0574	fecCD	iron compound ABC transporter, permease protein	Transport and binding proteins	Cations and iron carrying compounds	1.15	-2.93	-2.83	-2.70
WAN014H6A_at	4.5	9.1	2.0	2.4	2.5	SA0665	SA0566	SAR0618	SAS0577	SAV0609	MW0573		iron compound ABC transporter, substrate-binding protein, putative	Transport and binding proteins	Cations and iron carrying compounds	2.01	-2.28	-1.85	-1.82
WAN014HCI_at	2.3	3.8	1.1	1.0	1.3	SA0203	SA0217	SAR0216	SAS0200	SAV0225	MW0200	afuA	iron compound ABC transporter, substrate-binding protein, putative	Transport and binding proteins	Cations and iron carrying compounds	1.69	-2.06	-2.36	-1.79
WAN014GVX_at	27.9	131.8	7.1	10.5	9.4	SA0459	SA0374	SAR0407	SAS0365	SAV0389	MW0365	pbuX	xanthine permease	Transport and binding proteins	Nucleosides, purines and pyrimidines	4.73	-3.91	-2.65	-2.96
WAN014I60_at	99.1	78.1	18.0	38.4	39.5	SA1319	SA1140	SAR1274	SAS1232	SAV1300	MW1182	glpF	glycerol uptake facilitator protein	Transport and binding proteins	Other	-1.27	-5.51	-2.58	-2.51
WAN014GZM_at	8.0	3.5	1.7	1.8	2.8	SA1475	SA1269	SAR1448	SAS1379	SAV1436	MW1325		drug transporter, putative	Transport and binding proteins	Other	-2.30	-4.82	-4.56	-2.91
WAN014GPI_at	3.8	23.7	0.9	1.2	1.8	SA0303	SA0295	SAR0304	SAS0284	SAV0307	MW0284		acid phosphatase5-nucleotidase, lipoprotein e(P4) family	Transport and binding proteins	Other	6.18	-4.12	-3.24	-2.14
WAN014FXI_at	12.4	47.3	3.3	6.0	8.8	SA0407	SA0325	SAR0333	SAS0313	SAV0337	MW0313	glpT	glycerol-3-phosphate transporter	Transport and binding proteins	Other	3.82	-3.81	-2.05	-1.42
WAN014HA5_at	24.8	12.4	9.0	6.5	5.9	SA0754	SA0650					norA	drug transporter	Transport and binding proteins	Other	-2.00	-2.75	-3.79	-4.17
WAN014G2U_at	4.8	4.8	1.9	2.0	2.7	SA0163	SA0172	SAR0179	SAS0153	SAV0178	MW0152		drug transporter, putative	Transport and binding proteins	Other	1.01	-2.57	-2.45	-1.76
WAN014G4R_at	138.0	53.9	5.7	14.1	17.4	SA2521	SA2300	SAR2589	SAS2397	SAV2512	MW2431		transporter	Transport and binding proteins	Unknown substrate	-2.56	-24.21	-9.79	-7.95
WAN014H7R_at	60.3	18.0	9.2	8.6	8.8	SA0700	SA0599	SAR0653	SAS0609	SAV0643	MW0605	abcA	ABC transporter, ATP-binding/permease protein	Transport and binding proteins	Unknown substrate	-3.35	-6.59	-7.01	-6.84
WAN014GLN_at	251.1	249.7	67.4	80.1	45.1	SA0689	SA0588	SAR0642	SAS0598	SAV0632	MW0594	sitB	ABC3 transporter family protein	Transport and binding proteins	Unknown substrate	-1.01	-3.72	-3.13	-5.57
WAN014GL6_at	197.9	166.8	53.2	52.6	32.5	SA0688	SA0587	SAR0641	SAS0597	SAV0631	MW0593	sitA	ABC transporter, substrate-binding protein, putative	Transport and binding proteins	Unknown substrate	-1.19	-3.72	-3.76	-6.09
WAN014GM1_at	120.4	90.1	33.1	40.5	22.8	SA0690	SA0589	SAR0643	SAS0599	SAV0633	MW0595	sitC	ABC transporter, ATP-binding protein	Transport and binding proteins	Unknown substrate	-1.34	-3.63	-2.97	-5.27
WAN014G12_at	23.4	0.9	6.9	3.6	4.8	SA2462	SA2243		SAS2346	SAV2454	MW2378	ybbL	ABC transporter, ATP-binding protein	Transport and binding proteins	Unknown substrate	-26.77	-3.37	-6.46	-4.90
WAN014GPM_at	10.5	1.6	3.2	2.1	3.3	SA0306	SA0297	SAR0306	SAS0286	SAV0309	MW0286		ABC transporter, ATP-binding protein	Transport and binding proteins	Unknown substrate	-6.55	-3.33	-4.90	-3.21
WAN014GPK_at	12.7	2.0	3.8	1.9	3.5	SA0305	SA0296	SAR0305	SAS0285	SAV0308	MW0285		permease	Transport and binding proteins	Unknown substrate	-6.27	-3.31	-6.74	-3.66
WAN014HU4_at	3.6	3.6	1.2	1.3	1.3	SA1994	SA1745	SAR2023	SAS1855	SAV1931	MW1872		ABC transporter, ATP-binding protein	Transport and binding proteins	Unknown substrate	1.02	-2.99	-2.74	-2.73
WAN014HUC_at	3.6	3.0	1.5	1.3	1.8	SA1996	SA1747	SAR2025	SAS1857	SAV1933	MW1874		ABC transporter, ATP-binding protein	Transport and binding proteins	Unknown substrate	-1.20	-2.35	-2.68	-1.96
WAN014FVZ_at	1.6	1.3	0.7	0.8		SA2335	SA2132	SAR2427	SAS2233	SAV2341	MW2261	natA	ABC transporter, ATP-binding protein	Transport and binding proteins	Unknown substrate	-1.24	-2.20	-2.08	
WAN014GT0_x_at	26.8	5.5	2.2	1.9	2.8		SA0901	SAR1022	SAS0984	SAV1048			Glutamyl endopeptidase precursor (Staphylococcal serineproteinase) (V8 proteinase	Unclassified	Role category not yet assigned	-4.88	-12.14	-14.05	-9.59
WAN014HGD x at	7.6	1.3	1.5	1.0	1.3				SAS1664	SAV1738		DSP	smooth muscle caldesmon	Unclassified	Role category not yet assigned	-5.73	-5.14	-7.61	-5.64
WAN014HGB x at	9.1	1.4	1.8	1.8	1.8		SA1559			SAV1738		DSP	smooth muscle caldesmon	Unclassified	Role category not yet assigned	-6.52	-5.08	-5.14	-5.18
WAN014IPA_at	3.9	1.2	1.0	1.0	1.5		SA1750			SAV1937	MW1879		MapN protein	Unclassified	Role category not yet assigned	-3.33	-4.02	-3.76	-2.64
WAN014GGA_at	3.2	1.4	0.8	0.7	1.4		#N/A							Unclassified	Role category not yet assigned	-2.31	-3.86	-4.38	-2.23
WAN014GG2_at	8.1	3.9	2.2	1.7	2.4		#N/A							Unclassified	Role category not yet assigned	-2.07	-3.67	-4.65	-3.40
WAN014GFT_at	2.5	1.2	0.7	0.4			#N/A							Unclassified	Role category not yet assigned	-2.06	-3.32	-6.52	
WAN014ILU_x_at	2.2		0.7	0.8	1.1			SAR0401a					intergenic	Unclassified	Role category not yet assigned		-3.19	-2.93	-1.99
WAN014GPY_at	5.4	1.5	1.9	1.6	1.8		#N/A						antisense	Unclassified	Role category not yet assigned	-3.73	-2.89	-3.44	-2.94
WAN014ILI_x_at	4.4	3.0	1.5	1.8	2.3		#N/A						antisense	Unclassified	Role category not yet assigned	-1.46	-2.89	-2.39	-1.86
WAN01BYHK_at	2.3	1.1	0.9	0.9	0.7		#N/A						antisense	Unclassified	Role category not yet assigned	-2.17	-2.64	-2.71	-3.23
WAN014GPS_at	7.1	3.5	2.9	2.5	3.1		#N/A						antisense	Unclassified	Role category not yet assigned	-2.02	-2.48	-2.90	-2.31
WAN014IXG_at	1.1		0.4						SAS1909		MW1926		phi PVL orf 39-like protein	Unclassified	Role category not yet assigned		-2.45		
WAN014GSF_at	1.6	1.0	0.7	0.6			#N/A						intergenic	Unclassified	Role category not yet assigned	-1.63	-2.41	-2.81	
WAN014I24_at	2.4	3.3	1.0	0.9	1.1		SA0556	SAR0607		SAV0599			Staphylococcus aureus paralogous family TIGR01218	Unclassified	Role category not yet assigned	1.38	-2.38	-2.71	-2.19
WAN014GQF_at	31.7	11.2	13.3	16.0	24.3		#N/A						antisense	Unclassified	Role category not yet assigned	-2.82	-2.38	-1.98	-1.30
WAN014H67_at	23.9	21.7	10.1	8.5	10.0		#N/A						antisense	Unclassified	Role category not yet assigned	-1.10	-2.37	-2.82	-2.38
WAN01BU6J_at	4.8	2.9	2.1	2.0	3.0		#N/A						intergenic orf	Unclassified	Role category not yet assigned	-1.64	-2.29	-2.41	-1.61
WAN014GIJ_at	2.9	4.2	1.3	1.3	1.8		SA1753	SAR2033	SAS1864	SAV1941	MW1883		Bacteriophage phiN315	Unclassified	Role category not yet assigned	1.44	-2.20	-2.33	-1.65
WAN014GAI_at	19.9	22.6	9.3	10.2	10.8		#N/A						Intergenic	Unclassified	Role category not yet assigned	1.13	-2.15	-1.96	-1.84
WAN014HP0_at	6.2	3.6	3.0	1.9	2.2		#N/A							Unclassified	Role category not yet assigned	-1.73	-2.09	-3.23	-2.78
WAN01A738_x_at	1.7	0.8	0.8	0.8	1.4		#N/A							Unclassified	Role category not yet assigned	-2.14	-2.07	-2.27	-1.26
WAN01C4UG_at	1.3	1.4	0.6	0.8	1.4		SAV197			SAV1978			PVL orf 51-like protein	Unclassified	Role category not yet assigned	1.03	-2.06	-1.69	1.06
WAN014H6X_at	2.1	0.9	1.0	0.9	1.7		#N/A						antisense	Unclassified	Role category not yet assigned	-2.41	-2.03	-2.39	-1.22
AP001553-cds65 a	1.9		1.0				#N/A							Unclassified	Role category not yet assigned		-2.01		
WAN014G8A_at	1.9	2.9	0.9	0.8	0.6		SA2273			SAV2485			Staphylococcus tandem lipoproteins subfamily	Unclassified	Role category not yet assigned	1.55	-2.00	-2.32	-3.28
WAN014IKR_at	1.2	1.1	0.6				SA0093			SAV0097			Staphylococcus tandem lipoproteins subfamily	Unclassified	Role category not yet assigned	-1.08	-1.98		
WAN014GE2_at	32.4	3.7	3.6	2.0	3.8	SA2597	SA2367	SAR2661	SAS2467	SAV2581	MW2501		hydrolase, alpha/beta hydrolase fold family	Unknown function	Enzymes of unknown specificity	-8.71	-8.88	-16.09	-8.46
WAN014GBQ_at	67.4	13.9	10.9	9.1	8.9	SA2459	SA2240	SAR2541	SAS2343	SAV2451	MW2375		carboxylesterase family protein	Unknown function	Enzymes of unknown specificity	-4.85	-6.17	-7.39	-7.59
WAN014HAO_at	7.2	2.1	1.5	0.8	1.0	SA0763	SA0658	SAR0756	SAS0668	SAV0703	MW0665		oxidoreductase, aldo/keto reductase family	Unknown function	Enzymes of unknown specificity	-3.41	-4.91	-9.25	-7.06

Systematic	Stat.	Log	30'	120'	360'	COL	N315	MRSA	MSSA	Mu50	MW2	GENE NAME	Protein Function	TIGR Main Role	TIGR Sub Role	Fold change vs stationary phase			
																LB	30' in vivo	120' in vivo	360' in vivo
WAN014HNH_at	81.4	15.3	16.9	17.4	35.3	SA0111	SA0122	SAR0129	SAS0101	SAV0126	MW0100	butA	oxidoreductase, short-chain dehydrogenase/reductase family	Unknown function	Enzymes of unknown specificity	-5.33	-4.82	-4.67	-2.31
WAN014G3G_at	16.8	8.3	3.9	4.3	4.0	SA2021	SA1841	SAR2121	SAS1940	SAV2034	MW1958		carbon-nitrogen hydrolase family protein	Unknown function	Enzymes of unknown specificity	-2.02	-4.28	-3.92	-4.25
WAN014H3A_at	7.2	11.3	1.9	1.7	2.3	SA0602	SA0513	SAR0560	SAS0513	SAV0555	MW0510	gph	hydrolase, haloacid dehalogenase-like family	Unknown function	Enzymes of unknown specificity	1.56	-3.71	-4.32	-3.21
WAN014H1J_at	5.2	4.8	1.5	1.5	2.4	SA0569	SA0482	SAR0527	SAS0481	SAV0524	MW0479		ATP:guanido phosphotransferase family protein	Unknown function	Enzymes of unknown specificity	-1.09	-3.50	-3.38	-2.19
WAN014GU6_at	6.5	1.2	1.9	1.3	1.8	SA0399	SA0317	SAR0325	SAS0305	SAV0328	MW0305		oxidoreductase, putative	Unknown function	Enzymes of unknown specificity	-5.42	-3.46	-5.03	-3.62
WAN014H6E_at	2.7	2.8	0.8	0.9		SA0667	SA0568	SAR0620	SAS0579	SAV0611	MW0575		hydrolase, putative	Unknown function	Enzymes of unknown specificity	1.03	-3.45	-2.90	
WAN014HQJ_at	2.2	2.1	0.7	0.6		SA1048	SA0893	SAR1014	SAS0976	SAV1040			acetyltransferase, GNAT family	Unknown function	Enzymes of unknown specificity	-1.07	-3.29	-3.86	
WAN014H5O_at	6.0	6.8	2.0	1.9	1.6	SA0655	SA0557	SAR0608	SAS0568	SAV0600	MW0563		oxidoreductase, aldo/keto reductase 2 family	Unknown function	Enzymes of unknown specificity	1.14	-3.01	-3.05	-3.70
WAN014H3E_at	4.5	5.0	1.5	1.5	1.5	SA0606	SA0517	SAR0564	SAS0517	SAV0559	MW0514		hydrolase, haloacid dehalogenase-like family	Unknown function	Enzymes of unknown specificity	1.10	-2.97	-2.95	-3.05
WAN014H9O_at	3.6	0.8	1.2	1.0	1.0	SA0739	SA0634	SAR0732	SAS0644	SAV0679	MW0641		acetyltransferase, GNAT family	Unknown function	Enzymes of unknown specificity	-4.39	-2.95	-3.72	-3.49
WAN014FU5_at	14.0	3.5	4.9	3.1	4.1	SA2293	SA2095	SAR2386	SAS2192	SAV2302	MW2220		NAD/NADP octopine/nopaline dehydrogenase family protein	Unknown function	Enzymes of unknown specificity	-3.99	-2.84	-4.58	-3.44
WAN014H6I_at	2.5	2.7	0.9	0.8	0.9	SA0668	SA0569	SAR0621	SAS0580	SAV0612	MW0576		hydrolase, alpha/beta fold family	Unknown function	Enzymes of unknown specificity	1.05	-2.78	-3.11	-2.76
WAN014G75_at	6.9	2.9	2.5	1.7	2.4	SA2575	SA2347	SAR2641	SAS2446	SAV2560	MW2481		aminotransferase, class I	Unknown function	Enzymes of unknown specificity	-2.37	-2.78	-4.03	-2.92
WAN014H38_at	4.2	5.5	1.5	1.3	1.9	SA1545	SA1333	SAR1578	SAS1442	SAV1502	MW1456		oxidoreductase, short-chain dehydrogenase/reductase family	Unknown function	Enzymes of unknown specificity	1.30	-2.75	-3.20	-2.26
WAN014GVG_at	13.3	3.7	4.9	4.8	4.3	SA1189	SA1019	SAR1152	SAS1110	SAV1176	MW1059		acetyltransferase, GNAT family	Unknown function	Enzymes of unknown specificity	-3.57	-2.74	-2.76	-3.12
WAN014GG6_at	1.7	1.4	0.7	0.8		SA0197	SA0211	SAR0210	SAS0194	SAV0218	MW0194		oxidoreductase, Gfo/Idh/MocA family	Unknown function	Enzymes of unknown specificity	-1.20	-2.62	-2.23	
WAN014GCQ_at	30.1	18.1	11.8	6.0	7.4	SA2532	SA2309	SAR2601	SAS2406	SAV2521	MW2441		acetyltransferase, GNAT family	Unknown function	Enzymes of unknown specificity	-1.66	-2.55	-5.02	-4.08
WAN014G6L_at	6.6	7.4	2.7	3.0	2.4	SA2245	SA2052	SAR2340	SAS2146	SAV2255	MW2174		acetyltransferase, GNAT family	Unknown function	Enzymes of unknown specificity	1.11	-2.47	-2.18	-2.70
WAN014HD9_at	11.2	10.5	4.7	3.9	3.7	SA0827	SA0717	SAR0816	SAS0727	SAV0762	MW0724		acetyltransferase, putative	Unknown function	Enzymes of unknown specificity	-1.07	-2.40	-2.86	-3.05
WAN01BQMM_at	23.8	6.0	10.1	6.6	7.0	SA2368		SAR2460					acetyltransferase, GNAT family	Unknown function	Enzymes of unknown specificity	-3.94	-2.35	-3.60	-3.38
WAN014FUU_at	2.9	1.8	1.3	1.1	1.3	SA2303	SA2104	SAR2395	SAS2203	SAV2311	MW2231		inositol monophosphatase family protein	Unknown function	Enzymes of unknown specificity	-1.60	-2.27	-2.61	-2.27
WAN014G2Z_at	8.0	6.5	3.8	3.0	3.5	SA2488	SA2266						oxidoreductase, short-chain dehydrogenase/reductase family	Unknown function	Enzymes of unknown specificity	-1.24	-2.13	-2.70	-2.27
WAN014H7J_at	19.2	18.1	9.2	7.2	8.1	SA1625	SA1397	SAR1645	SAS1506	SAV1568	MW1520	cdd	cytidine and deoxycytidylate deaminase family protein	Unknown function	Enzymes of unknown specificity	-1.06	-2.09	-2.69	-2.38
WAN014HJT_at	6.7	6.0	3.3	2.4	2.3	SA1835	SA1606	SAR1868	SAS1709	SAV1788	MW1726		oxidoreductase, aldo/keto reductase family	Unknown function	Enzymes of unknown specificity	-1.13	-2.05	-2.84	-2.92
WAN014H6P_at	8.1		4.0	2.6	4.1	SA0671	SA0572	SAR0624	SAS0583	SAV0615	MW0579		hydrolase, alpha/beta fold family	Unknown function	Enzymes of unknown specificity		-2.02	-3.14	-1.98
WAN014HYD_at	3.2	2.3	1.6	1.3	1.9	SA1188	SA1018	SAR1151	SAS1109	SAV1175	MW1058		hydrolase, haloacid dehalogenase-like family	Unknown function	Enzymes of unknown specificity	-1.37	-1.99	-2.46	-1.65
WAN014IQZ_at	40.6	13.5	4.2	3.7	5.3	SA2660	SA2431		SAS2524	SAV2638	MW2559	isaB	immunodominant antigen B	Unknown function	General	-3.02	-9.55	-10.91	-7.70
WAN014G2S_at	16.3	0.8	2.8	2.0	3.0	SA2484	SA2262	SAR2561	SAS2365	SAV2474	MW2398		alkylhydroperoxidase, AhpD family	Unknown function	General	-20.07	-5.83	-8.22	-5.45
WAN014GPZ_at	3.4	6.0	0.6	0.5	1.2	SA0313	SA0305	SAR0313	SAS0293	SAV0316	MW0293		ROK family protein	Unknown function	General	1.77	-5.27	-6.32	-2.90
WAN014HQ2_at	110.8	14.3	21.9	14.5	16.1	SA1933	SA1692	SAR1965	SAS1797	SAV1875	MW1815		ThiJ/PfpI family protein	Unknown function	General	-7.73	-5.06	-7.64	-6.88
WAN014IV3_at	6.1	1.8	1.5	1.4	1.4	SA0410	SA0328		SAS0316	SAV0340	MW0316		FMN reductase-related protein	Unknown function	General	-3.42	-4.10	-4.34	-4.24
WAN014GUG_at	3.5	1.0	1.0	1.0	1.3	SA0408	SA0326	SAR0334	SAS0314	SAV0338	MW0314		glyoxalase family protein	Unknown function	General	-3.68	-3.65	-3.41	-2.77
WAN014H3T_at	105.8	42.6	29.5	20.3	30.1	SA0618	SA0529	SAR0575	SAS0529	SAV0571	MW0526		SIS domain protein	Unknown function	General	-2.48	-3.59	-5.21	-3.52
WAN014G8E_at	4.7	4.4	1.4	1.1	1.5	SA2602	SA2372						chromosome assembly protein homolog, putative	Unknown function	General	-1.06	-3.33	-4.29	-3.06
WAN014HIG_at	4.1	1.3	1.4	0.9	1.2	SA0921	SA0780	SAR0882	SAS0790	SAV0919	MW0802		hemolysin, putative	Unknown function	General	-3.13	-2.93	-4.64	-3.44
WAN014HJH_at	3.0	4.3	1.1	1.2	1.1	SA0931	SA0790	SAR0891	SAS0799	SAV0929	MW0811	pho2	p-nitrophenyl phosphatase	Unknown function	General	1.42	-2.63	-2.47	-2.76
WAN014H7S_at	6.7	4.1	2.7	2.3	2.6	SA1628	SA1400	SAR1648	SAS1509	SAV1571	MW1523	phoH	PhoH family protein	Unknown function	General	-1.63	-2.46	-2.91	-2.54
WAN014GF4_at	53.6	44.3	22.5	17.5	19.9	SA2666	SA2437	SAR2723	SAS2530	SAV2644	MW2565		N-acetylmuramoyl-L-alanine amidase domain protein	Unknown function	General	-1.21	-2.39	-3.07	-2.69
WAN014G0U_at	2.2	1.6	0.9	1.1	1.3	SA1894	SA1656	SAR1929	SAS1759	SAV1838	MW1778		HIT family protein	Unknown function	General	-1.32	-2.38	-2.04	-1.62
WAN014H5S_at	2.2	3.7	0.9	1.0	1.1	SA0657	SA0559	SAR0610	SAS0570	SAV0602	MW0565		protease synthase and sporulation negative regulatory protein pai 1, putative	Unknown function	General	1.66	-2.36	-2.14	-1.95
WAN014H6J_at	2.0	1.6	0.9	0.8	0.8	SA1607	SA1380	SAR1627	SAS1488	SAV1550	MW1502		5-formyltetrahydrofolate cyclo-ligase family protein	Unknown function	General	-1.27	-2.29	-2.43	-2.63
WAN014GZ2_at	11.6	10.5	5.1	3.9	4.3	SA1460	SA1258	SAR1438	SAS1368	SAV1425	MW1315	degV	degV family protein	Unknown function	General	-1.11	-2.28	-2.96	-2.69
WAN014H5W_at	9.4	15.0	4.2	3.5	3.8	SA0658	SA0560	SAR0611	SAS0571	SAV0603	MW0566		HD domain protein	Unknown function	General	1.59	-2.25	-2.71	-2.45
WAN014GOS_at	12.2	13.0	5.6	3.7	4.8	SA0022	SA0020	SAR0021	SAS0021	SAV0021	MW0021	yycI	yycI protein	Unknown function	General	1.06	-2.19	-3.33	-2.55
WAN014GZZ_at	106.8	15.8	51.1	25.2	23.0	SA0541	SA0456	SAR0499	SAS0455	SAV0498	MW0453	spoVG	stage V sporulation protein G	Unknown function	General	-6.75	-2.09	-4.23	-4.65
WAN014HLJ_at	116.7	113.4	57.9	50.8	48.7	SA0977	SA0833	SAR0935	SAS0842	SAV0972	MW0854		mrp protein homolog	Unknown function	General	-1.03	-2.02	-2.30	-2.40
